# Copper catalyzed photoredox synthesis of α-keto esters, quinoxaline, and naphthoquinone: controlled oxidation of terminal alkynes to glyoxals†
†Electronic supplementary information (ESI) available. CCDC 1584501, 1584500 and 1847226. For ESI and crystallographic data in CIF or other electronic format see DOI: 10.1039/c8sc03447h


**DOI:** 10.1039/c8sc03447h

**Published:** 2018-08-29

**Authors:** Deb Kumar Das, V. Kishore Kumar Pampana, Kuo Chu Hwang

**Affiliations:** a Department of Chemistry , National Tsing Hua University , Hsinchu , Taiwan , Republic of China . Email: kchwang@mx.nthu.edu.tw ; Fax: +886 35711082

## Abstract

Herein, we report a facile visible light induced copper catalyzed controlled oxidation of terminal C≡C alkynes to α-keto esters and quinoxalines *via* formation of phenylglyoxals as stable intermediates, under mild conditions by using molecular O_2_ as a sustainable oxidant. The current copper catalysed photoredox method is simple, highly functional group compatible with a broad range of electron rich and electron poor aromatic alkynes as well as aliphatic alcohols (1°, 2° and 3° alcohols), providing an efficient route for the preparation of α-keto esters (43 examples), quinoxaline and naphthoquinone with higher yields than those in the literature reported thermal processes. Furthermore, the synthetic utility of the products has been demonstrated in the synthesis of two biologically active molecules, an *E. coli* DHPS inhibitor and CFTR activator, using the current photoredox process. In addition, we applied this methodology to the one-pot synthesis of a heterocyclic compound (quinoxaline, an FLT3 inhibitor) by trapping the intermediate phenylglyoxal with *O*-phenylenediamine. The intermediate phenylglyoxal can also be isolated and further reacted with an internal alkyne to form naphthoquinone. This process can be readily scaled up to the gram scale.

## Abstract 2

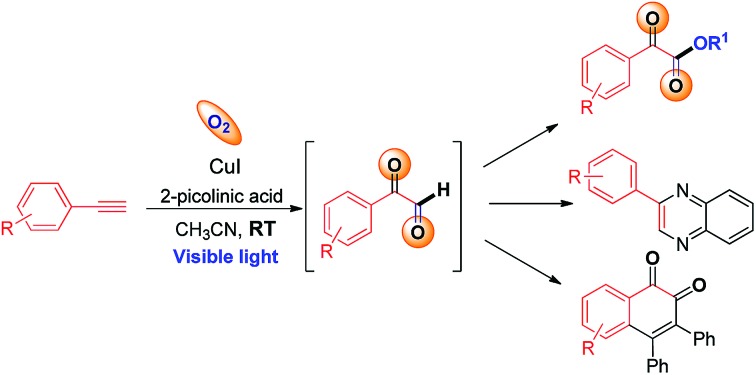
Controlled oxidation of the terminal C≡C triple bond using O_2_ (1 atm) as an oxidant and reagent.

## Introduction

**Scheme 1:**
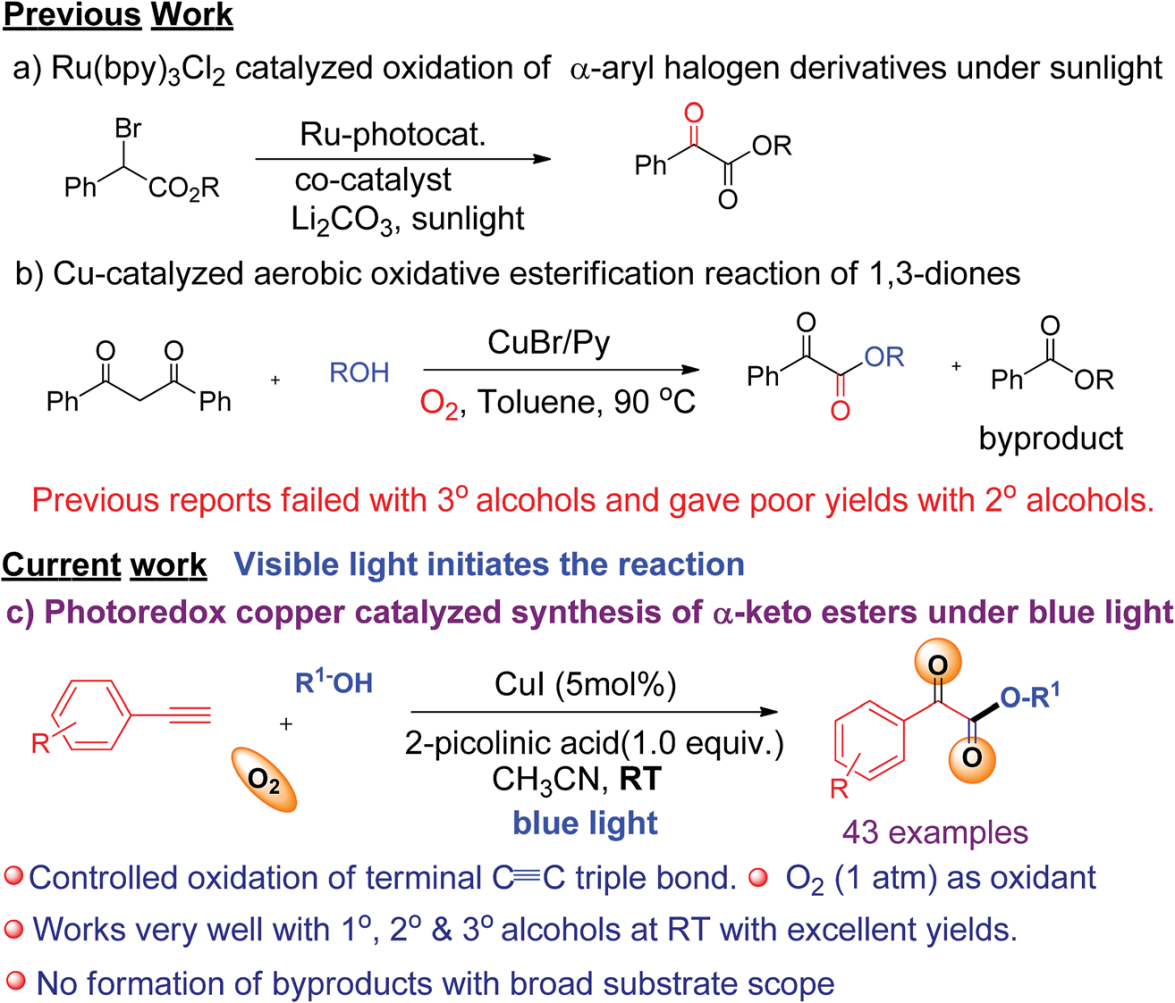
Different synthetic approaches toward α-keto esters.

Photoredox catalysis has been proven to be a powerful tool for the construction of new chemical bonds, and has attracted attention from researchers all around the world.^1^ Photoredox copper-based complexes have been shown to be an inexpensive, potent catalytic system for various organic transformations.^2^ In recent years, the direct introduction of two vicinal functional groups into terminal alkynes *via* activation of the C≡C triple bond has become a very attractive process to achieve valuable synthons, bioactive natural products, and their synthetic analogues.^3^,^4^ In particular, catalyzed oxidation of C≡C triple bonds by transition metal complexes in the presence of molecular O_2_ plays an important role in the chemical industry.^5^ However, it remains very challenging to avoid over-oxidation of C≡C triple bonds to generate over-oxidized products.^6^ Our group has recently reported various visible light-mediated copper(i)-catalysed cross-coupling and C–H annulation reactions.^7^ It has been demonstrated that copper(i) phenylacetylide is the key photocatalyst involved in these visible light induced coupling reactions.^7^ It was shown that photo-irradiation of copper(i) phenylacetylide in the presence of molecular oxygen can generate Cu(ii)-phenylacetylide and a superoxide radical anion *via* the single electron transfer (SET) process.7*d* The generated superoxide radical anion coordinates to a copper ligand complex, and is responsible for controlled oxidation of the C≡C triple bond of a terminal alkyne.7*d* Similarly, we envisaged that a terminal alkyne can be transformed into valuable α-keto esters *via* controlled oxidation. α-Keto ester analogues are considered to be valuable precursors and intermediates for various pharmaceuticals and bioactive molecules.^8^ Due to their vast potential,^8^ many research groups have put significant efforts into the synthesis of these compounds in recent years.^9^ Recently, Jiao *et al.* reported the photoredox catalyzed synthesis of α-keto esters *via* oxidation of α-aryl halo derivatives using an expensive ruthenium catalyst under sunlight irradiation (Scheme 1a).10*a* Thereafter, the same group described the aerobic oxidative esterification reaction of 1,3-diones *via* C–C bond cleavage at high temperatures (Scheme 1b)10*b* that resulted in the formation of unwanted esters as byproducts. Later on, Song *et al.* demonstrated the oxidative esterification of acetophenones at high temperatures.^11^ Despite significant progress, common major limitations of literature strategies include the use of expensive catalysts,9a,d,f,^11^ pre-synthesized starting substrates,9b,^11^ the need for additives or bases,9d–g,10a,^11^ the requirement of stoichiometric amounts of oxidants,9*a* formation of ester byproducts,10*b* harsh reaction conditions,9b,c,e–g,10b,^11^ and poor or no yield of products when 2° or 3° alcohols were used.9a–e,10b,^11^ Therefore, there is a strong need to develop an efficient modality for the construction of α-keto esters that can conquer the above-mentioned limitations. In this communication, we report a visible-light-induced copper catalyzed synthesis of α-keto esters from the reaction of a variety of alkynes and aliphatic alcohols under mild conditions using O_2_ as an oxidant (Scheme 1c).

The significance of the present work includes the following: (a) this is the first example of oxidation of terminal alkynes to α-keto esters under visible light at room temperature under mild conditions; (b) low toxic, inexpensive CuI was used as a catalyst and abundant O_2_ as an oxidant, (c) controlled oxidation of the C≡C triple bond to phenylglyoxals, and thus no formation of ester or homo-coupling byproducts, and (d) a broad substrate scope and compatibility with a wide range of aromatic alkynes and 1°, 2°, or 3° alcohols. To the best of our knowledge, the use of terminal alkynes as a key starting material for the synthesis of α-keto esters under visible light is yet to be reported.

## Results and discussion

When a mixture of phenyl acetylene (**1a**) (0.5 mmol), MeOH (**2a**) (2 mL), copper iodide (CuI, 5 mol%), and 2-picolinic acid (1.0 equiv.) as a ligand in CH_3_CN (4 mL) in the presence of molecular O_2_ was irradiated under blue LEDs at room temperature for 12 h, it furnished the desired α-keto ester (**3a**) with a yield of 86% (Table 1, entry 1). When CuI was replaced by other CuX catalysts (X = Cl, Br), the desired product, **3a**, was not formed (Table 1, entry 2). The halide anion effect was attributed to the larger size and polarizability and better leaving ability of iodide ions in organic solvents as compared to other halide anions, which facilitates easy formation of copper complexes for this reaction. Removal of the copper catalyst or ligand failed to produce **3a** (Table 1, entries 3 & 4). When the amount of ligand loading was decreased to 5 or 10 mol%, the conversion of phenyl acetylene to the desired α-keto ester (**3a**) was low and either the reaction failed or gave trace amounts of the desired product (Table 1, entries 5 & 6). Reaction with 50 mol% of 2-picolinic acid as a ligand provided product **3a** in 71% yield (Table 1, entry 7), whereas increasing the amount of ligand to 2.0 equivalents gave an α-keto ester in 85% yield (Table 1, entry 8). The yield was similar when 1.0 equivalent of ligand was used (Table 1, entry 1). Increasing or decreasing the amount of ligand failed to increase the yield of the desired product; thus it can be concluded that the optimal amount of the 2-picolinic acid ligand is 1.0 equiv. Replacing the ligand with di-picolinic acid does not affect the yield of **3a** (Table 1, entry 9), whereas in the case of 2-amino pyridine as a ligand, we observed a complete inhibition of **3a** (Table 1, entry 10). The yield of **3a** remains the same in neat MeOH, but tends to decrease in THF and toluene (Table 1, entries 11–13). Reactions under ambient white light irradiation produced **3a** with 82% yield (Table 1, entry 15). Removal of either O_2_ or light leads to no product formation, indicating their crucial roles in the current protocol (Table 1, entries 16 & 17).

**Table 1:** Optimization of reaction conditions^*a*^

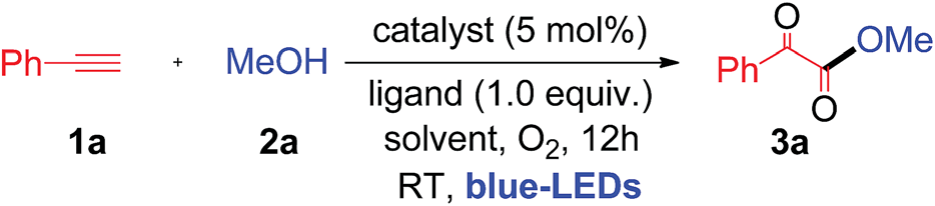				
Entry	Cu [catalyst]	Ligand	Solvent	Yield^*b*^ %
1	CuI	2-Picolinic acid	CH_3_CN	86
2^*c*^	Other copper salts	2-Picolinic acid	CH_3_CN	n.r
3	None	2-Picolinic acid	CH_3_CN	n.r
4	CuI	None	CH_3_CN	0
5^*d*^	CuI	2-Picolinic acid	CH_3_CN	0
6^*e*^	CuI	2-Picolinic acid	CH_3_CN	<5
7^*f*^	CuI	2-Picolinic acid	CH_3_CN	71
8^*g*^	CuI	2-Picolinic acid	CH_3_CN	85
9	CuI	Dipicolinic acid	CH_3_CN	82
10	CuI	2-Aminopyridine	CH_3_CN	0
**11** ^*h*^	**CuI**	**2-Picolinic acid**	**None**	**86**
12	CuI	2-Picolinic acid	THF	65
13	CuI	2-Picolinic acid	Toluene	36
14^*i*^	CuI	2-Picolinic acid	MeOH	76
15^*j*^	CuI	2-Picolinic acid	MeOH	82
16^*k*^	CuI	2-Picolinic acid	MeOH	n.r
17^*l*^	CuI	2-Picolinic acid	MeOH	n.r

^*a*^Reaction conditions: 1a (0.50 mmol), MeOH (2 mL), CH_3_CN (4 mL), ligand (0.5 mmol) and catalyst (0.05 mmol); the reaction mixture was irradiated with blue LEDs (40 mW cm^–2^ at 460 nm) at RT for 12 h under O_2_ (1 atm).

^*b*^Isolated yields.

^*c*^Other copper salts such as CuX (X = Cl, Br).

^*d*^5 mol% of 2-picolinic acid was used.

^*e*^10 mol% of 2-picolinic acid was used.

^*f*^50 mol% of 2-picolinic acid was used.

^*g*^2.0 equiv. of 2-picolinic acid were used.

^*h*^MeOH (4 mL) was used both as the reactant and solvent.

^*i*^Under 1 atm. air.

^*j*^Under ambient white light irradiation (12 h, 8 mW cm^–2^ at 460 nm).

^*k*^At room temperature and in the dark.

^*l*^Under a N_2_ atmosphere. n.r. = no reaction.

Having established the optimal reaction conditions, we then investigated the scope and applicability of this reaction using different 1°, 2° and 3° alcohols as substrates for the synthesis of substituted α-keto esters (Table 2). The reactions were performed with various primary alcohols like ethanol (**2b**), *n*-propanol (**2c**), *n*-butanol (**2d**) and 2-methylpropan-1-ol (**2e**), and the desired product (**3b–e**) was obtained in good yields at room temperature (Table 2). The current photochemical process also works well for primary alcohols like 2-methoxyethanol (**2f**) and benzyl alcohol (**2g**) providing α-keto esters (**3f** and **3g**) in good to excellent yields under similar reaction conditions (Table 2).

**Table 2:** Substrate scope of alcohols^*a*^

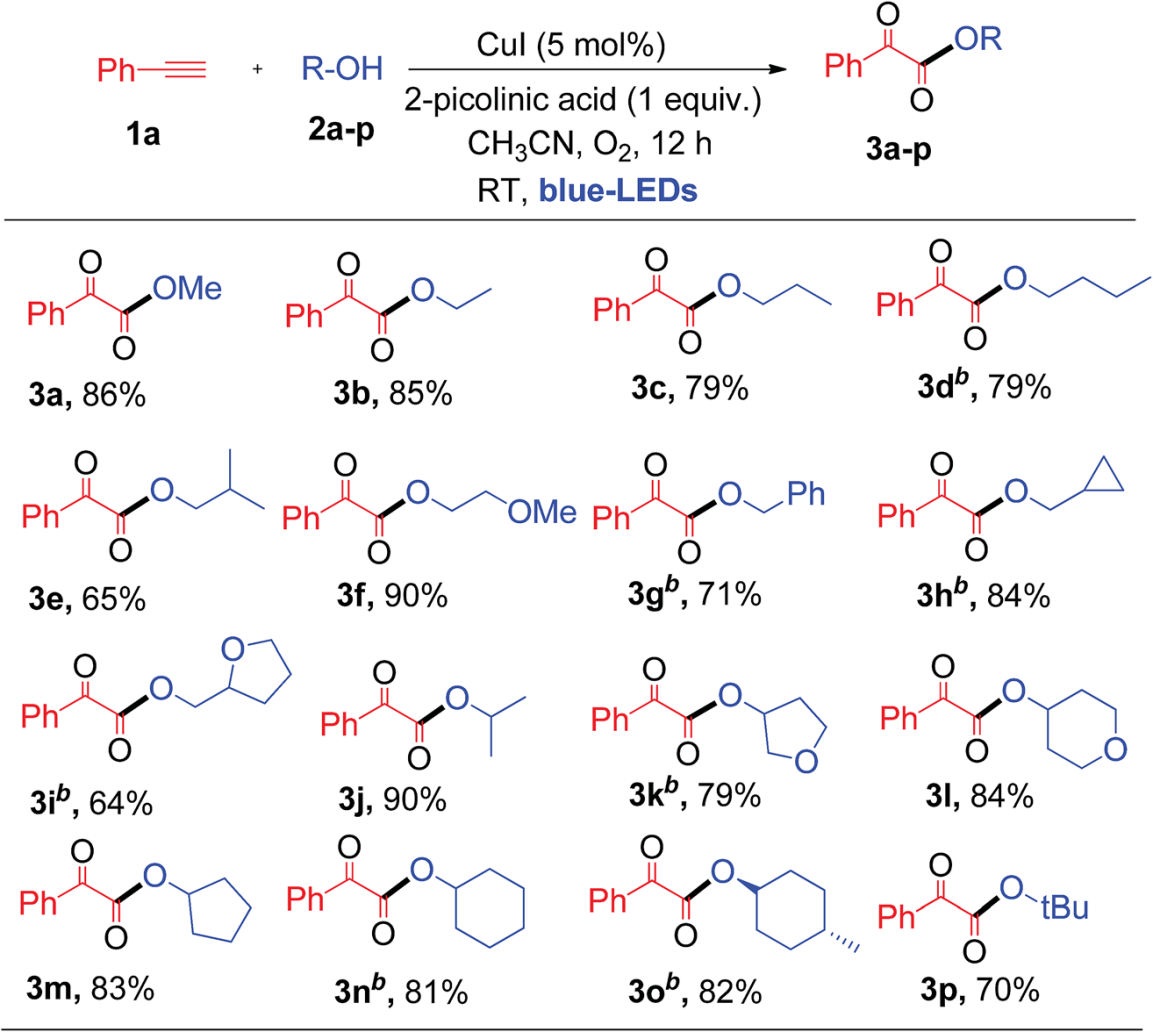

^*a*^Standard conditions.

^*b*^2 mmol of ROH in 4 mL of CH_3_CN, isolated yields.

Interestingly, cyclopropanemethanol (**2h**) and tetrahydrofurfuryl alcohol (**2i**) reacted well with **1a** to produce **3h** and **3i** in 84% and 68% yields, respectively, without cyclic ring opening. Next, **1a** reacts with 2° alcohols (**2j–2l**) smoothly to afford the corresponding α-keto esters (**3j–3l**) in good yields. Slightly strained or labile alcohols (**2h**, **2i**, **2k**, and **2l**) worked well in this protocol, without producing any cleavage products, which is not possible using the earlier thermal processes. Besides, **1a** reacts with alicyclic 2° alcohols (**2m–2o**) to afford the desired products (**3m–3o**) in good yields (Table 2). Unfortunately, this protocol does not work for aromatic alcohols, such as phenol, which was attributed to the fact that phenol is oxidized to *p*-benzoquinones in the presence of copper and O_2_.7a,^12^ The reaction of **1a** with tertiary butanol (**2p**) provided α-keto ester **3p** in 70% yield (Table 2). It is worth noting that the transformation of terminal alkynes to α-keto esters using tertiary alcohols has no precedent literature reports. Unfortunately, this protocol does not work for aliphatic amines. Both primary and secondary amines, such as *n*-propyl amine and piperidine, were used as nucleophiles for the present system, but no α-ketoamide product was observed. Next, a competitive reaction of phenyl acetylene (**1a**) with equal moles of 1°, 2° and 3° alcohols, such as MeOH (**2a**), isopropanol (**2j**) and tertiary butanol (**2p**), under standard conditions was surveyed, which afforded α-keto ester **3a** as a major product in 73% yield derived from the 1° alcohol, *i.e.*, MeOH. Product **3j** derived from the 2° alcohol was formed in trace quantities without any α-keto ester **3p** resulting from tertiary butanol. For nucleophilic attack on the glyoxal aldehyde, the 3° alcohol is expected to be better than the 2° alcohol and 1° alcohol. This observed result clearly indicates that steric hindrance plays a more important role than the electronic factor, which leads to a predominance of the primary alcohol in the coupling reaction.

Next, the substrate scope of aryl alkynes was examined with different aliphatic alcohols under the standard conditions (Table 3). The electron neutral and halo-(Cl, F, and I) substituted phenyl acetylenes readily react with aliphatic alcohols to afford the corresponding α-keto esters (**4b–4j**) with good to excellent yields as depicted in Table 3. Aryl alkynes with strong electron withdrawing and donating (CF_3_, CN, nitro, acetyl, ester, sulfone, and methoxy) groups showed excellent tolerance in the current photoredox protocol to give the corresponding α-keto esters (**4k–4t**) in good yields (Table 3). Copper-catalyzed aerobic oxidative coupling reactions involving electron rich substituted terminal alkynes suffer from homo-coupling byproducts.7*d* However, in the current process no homo-coupling product was observed. Notably, the present photoredox process works well for the reaction of 1,3-dialkynes to generate 1,3-α-diketo ester products **4u** and **4v** in good yields when using methanol as the solvent. The synthesis of 1,3-diketo esters is either difficult8b,^13^ or not achievable by the previously reported thermal processes. However, in contrast, it was easily achieved with the current photoredox process.

**Table 3:** Substrate scope of aryl terminal alkynes^*a*^

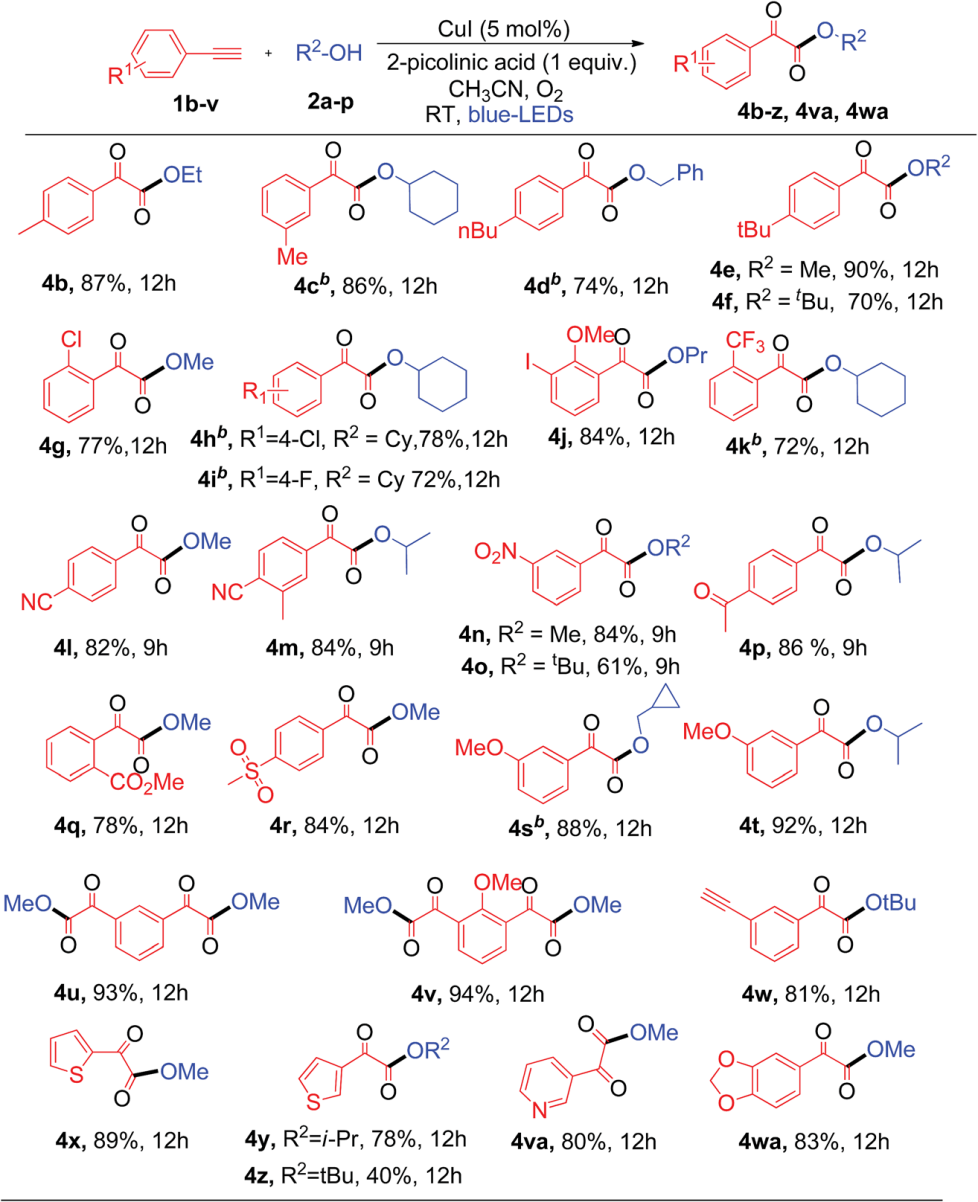

^*a*^Standard conditions.

^*b*^2 mmol of R–OH in 4 mL of CH_3_CN.

Note that when tertiary butanol was coupled with 1,3-dialkyne, only mono α-keto ester **4w** was obtained in 81% yield, where the absence of the di-substituted α-keto ester might be due to the steric hindrance effect from the bulky tertiary butyl group in the S_N_2 reaction. Notably heterocyclic alkynes 2-ethynylthiophenes, 3-ethynylthiophenes and 3-ethynylpyridine, which are usually sensitive to oxidative conditions, also effectively react with 1°, 2° and 3° alcohols to generate the desired α-keto esters (**4x–4z** and **4va**) in moderate to good yields. However, heterocyclic alkynes ethynyl indole and ethynyl pyrimidine failed to give the desired α-keto esters under the current protocol. This protocol was successful in producing α-keto ester **4wa** in 83% yield when heteroaryl alkyne 5-ethynyl-1,3-benzodioxole was used under similar conditions. Unfortunately, aliphatic terminal alkynes did not work for this protocol and failed to produce the corresponding α-keto esters as products. The reason for the failure of the aliphatic alkynes is most probably due to the lower acidity of aliphatic terminal alkynes as compared to aromatic ones, thus making the step of formation of copper phenylacetylide from aliphatic alkynes slower than that from aromatic alkynes.

**Scheme 2:**
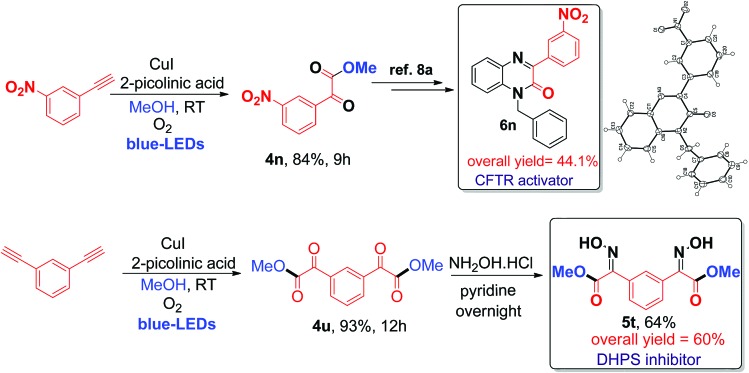
Synthesis of biologically active phenylquinoxalinone **6n** (CFTR activator) and bis oxime ester **5t** (DHPS inhibitor).

Finally, the application of the current visible light-initiated Cu(i)-catalyzed strategy was demonstrated for the expedient synthesis of compounds with biological activity, such as 1-benzyl-3-(3-nitrophenyl)quinoxalin-2(1*H*)-one **6n** (a CFTR activator)8*a* and bis oxime ester **5t** (an *E. coli* DHPS inhibitor).8*c* Preparation of phenylquinoxalinone **6n** could be carried out in 3 steps in 44% overall yield (Scheme 2), which is greener and better than the literature reported method (4 steps with an overall yield of 32%) (Schemes S1 and S2, ESI†).8*a*

Next, the preparation of bis oxime ester **5t** was carried out in 2 steps in 60% overall yield (Scheme 2), which is also better and greener than the literature reported process (4 steps, 25% overall yield) using pre-synthesized starting substrates.8*c* In addition, the current process can be readily scaled up to a gram scale (1.029 g of 3-nitro phenyl acetylene); 1.16 g of **4n** was obtained (79% yield) after irradiation with blue LEDs for 12 h at room temperature (ESI) and we have further evaluated and compared the green chemistry metrics (Fig. S5 and S6, ESI†).7*d* The structures of **4n** and **6n** were confirmed by single-crystal X-ray diffraction (Fig. S7 and S8, ESI†). In addition, compounds **4u**, **4v**, and **4x** can be used as precursors for synthesizing biologically active molecules (Scheme S7, ESI†).

**Scheme 3:**
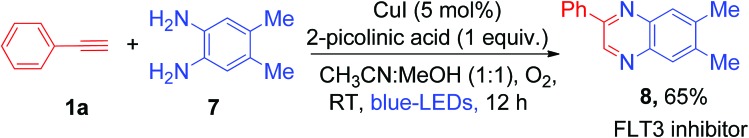
One-pot synthesis of 2-phenyl quinoxaline (an FLT3 inhibitor) by *in situ* trapping of phenylglyoxal using commercially available substrate 1,2-phenylenediamine.

Synthesis of quinoxaline *via* double condensation of 1,2-phenylenediamines with phenylglyoxals in the presence of a catalyst is a well-established concept in organic synthesis.14*a* Hashmi *et al.* reported gold and silver bi-metal co-catalyzed synthesis of quinoxaline derivatives from terminal alkynes which involved the oxidation of phenyl acetylene to phenylglyoxals using external oxidant pyridine N-oxide (4 equiv.)14*b* (Scheme S6, ESI†). Recently, it was shown that quinoxaline derivatives can be synthesized by copper-catalyzed oxidative reaction of phenylglyoxal with *o*-phenylenediamines.14*c* Inspired by the above literature reports, we hypothesized that controlled oxidation of phenyl acetylene will generate phenylglyoxal as a possible intermediate in the present copper catalyzed photoredox process. Thus, trapping of the phenylglyoxal intermediate with 1,2-phenylenediamines may lead to one-pot synthesis of pharmacologically active 2-phenyl quinoxaline by using a cheaper Cu catalyst and abundant molecular O_2_ as an oxidant. So, under the same reaction conditions, we added 1.0 equiv. of 4,5-dimethylbenzene-1,2-diamine (**7**) to the reaction solution and irradiated it with visible light for 12 h at room temperature (Scheme 3). Not surprisingly, we obtained the corresponding 6,7-dimethyl-2-phenylquinoxaline (**8**), which is a biologically active FLT3 inhibitor,^15^ in 65% yield as a product in this unprecedented photoredox copper catalyzed one-pot process (Scheme 3). We did not observe the formation of 3-phenylquinoxalin-2-ol as a product in the current photoredox method, which was previously reported as a key product under strong basic conditions.7*g* The difference in the formation of products was attributed to the differences in the reaction conditions and thus different reaction mechanisms (mechanistic comparison, Scheme S7, ESI†).

**Scheme 4:**
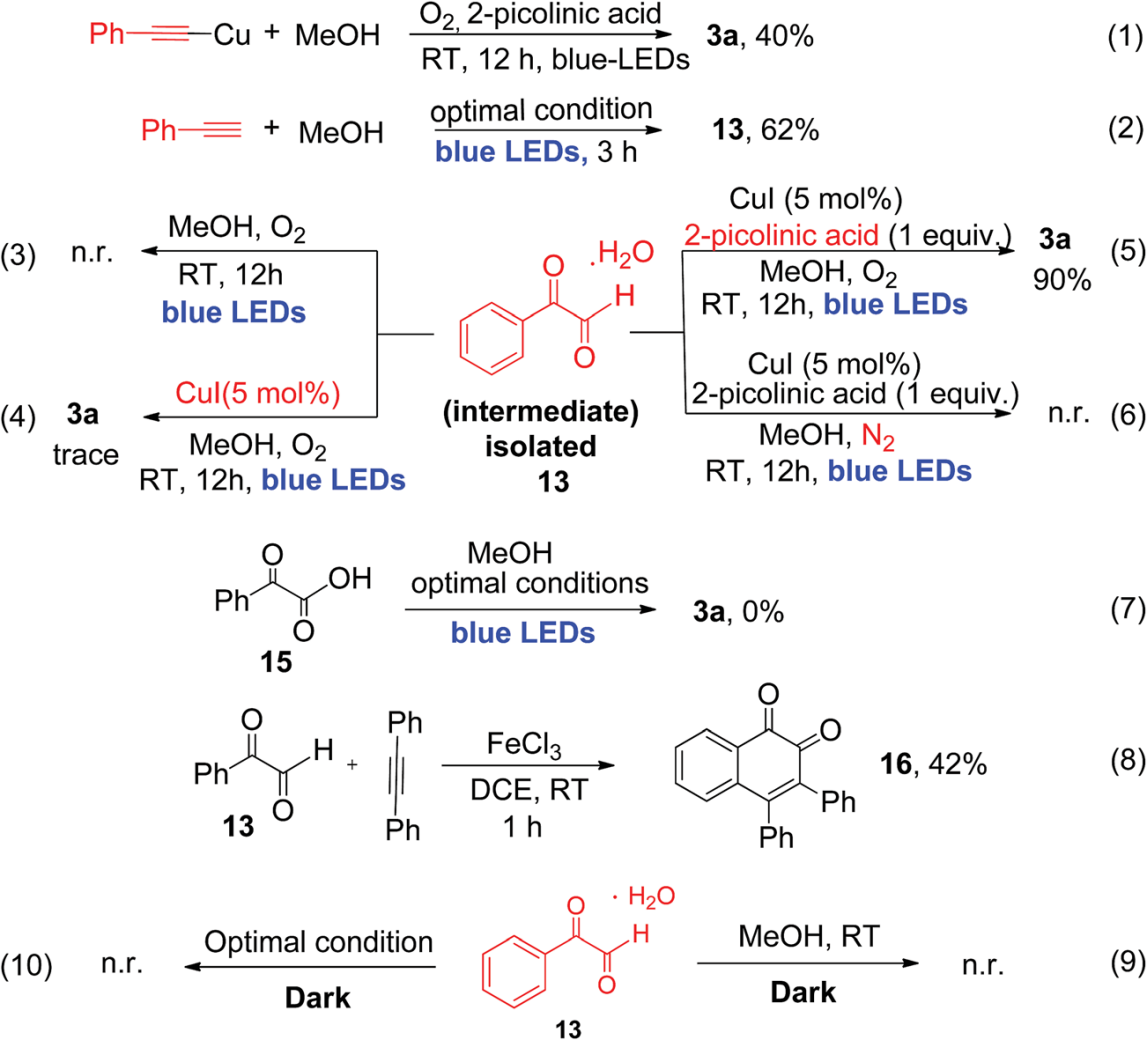
Mechanistic control studies.

To provide detailed insights regarding the reaction mechanism, we carried out several control experiments, as shown in Scheme 4. First, pre-synthesized copper(i)-phenylacetylide **1a′** was reacted with MeOH, in the absence of CuI under similar reaction conditions, which produced the desired α-keto ester (**3a**) with 40% yield after 12 h of irradiation (eqn (1), Scheme 4).

The reduced yield can be attributed to the fact that the isolated Cu(i)-phenylacetylide powder exists in highly aggregated forms.7d,^16^ This result implies that the *in situ*-generated Cu(i)-phenylacetylide is most probably the key light-absorbing photocatalyst involved in this oxidative coupling reaction. Next, we performed a short-time reaction of 3 h, under the optimal conditions, and we were delighted to isolate phenylglyoxal **13** as a stable intermediate in 62% yield (eqn (2), Scheme 4). Phenylglyoxals are important precursors in organic synthesis, and can be used to construct various biologically active heterocyclic compounds.14b,^17^ In the literature, very few methods are available for the synthesis of glyoxal derivatives.14*b* The most common method for the synthesis of phenylgloxal involves SeO_2_ mediated oxidation of substituted methyl ketones under harsh reaction conditions.^18^ Recently, photoredox oxidation of brominated acetophenones to phenylglyoxal was reported using an expensive ruthenium photocatalyst.^19^ That method, however, cannot use commercially available phenylacetylene as the starting substrate. In contrast, the synthesis of phenylglyoxal was easily achieved in a short time in our current study under mild reaction conditions using inexpensive copper to catalyze the photoredox process and commercially available aryl alkynes as starting substrates. After the isolation of phenylglyoxal, we conducted some key control experiments with **13** for better understanding of the reaction mechanism. First, the reaction of phenylglyoxal with MeOH was carried out in the presence of light and O_2_, but in the absence of the CuI catalyst, which led to no formation of α-keto ester **3a** (eqn (3), Scheme 4). When phenylglyoxal reacted with the solvent MeOH in the presence of 5 mol% CuI catalyst, light and O_2_, but in the absence of the 2-picolinic acid ligand, only a trace amount of **3a** was formed (eqn (4), Scheme 4). When the control reaction was performed in the presence of the CuI catalyst, 2-picolinic acid, O_2_, and blue light irradiation, **3a** was produced in 90% yield (eqn (5), Scheme 4). If the same reaction was carried out in a N_2_ atmosphere, no formation of **3a** was observed (eqn (6), Scheme 4). From the above control experiments (eqn (3)–(6), Scheme 4), it is very clear that the CuI catalyst, 2-picolinic acid ligand, O_2_, and blue light irradiation all are very crucial factors for the formation of the α-keto ester product **3a**.

Selective oxidation of terminal alkynes to glyoxal, free from the subsequent over-oxidation to glyoxalic acid, is a very challenging reaction in synthetic chemistry.^6^ In our current protocol, selective oxidation of phenyl acetylene to phenyl glyoxal was achieved successfully and no phenyl glyoxalic acid resulting from over-oxidation was observed. Thus, when phenyl glyoxalic acid **15** was reacted with MeOH under the same conditions, we did not observe product **3a**, which clearly suggests that over-oxidation of glyoxal to glyoxalic acid^20^ did not occur under the current reaction conditions (eqn (7), Scheme 4). 2-Picolinic acid plays a crucial role in avoiding the formation of the homocoupling product from copper phenylacetylide (which is a common side product in a reaction involving terminal alkynes in the presence of a copper catalyst) and it directs the system to activate terminal C≡C bonds *via* controlled oxidation to phenylglyoxal. It is documented in the literature that nitrogen containing ligands can reduce the formation of polymeric byproducts and Glaser alkyne–alkyne homocoupling products.21*a* Hence the optimal amount of ligand is found to be 1.0 equivalent due to the above-mentioned facts. Formation of the polymeric form of Cu(ii) bis-picolinate (single crystal X-ray, Fig. S9, ESI†) might be one of the reasons for the decrease in the yield when the reaction was carried out with 5 and 10 mol% of 2-picolinic acid as a ligand. Also due to the amphoteric nature of 2-picolinic acid,21*b* it can help maintain the acidic pH of the reaction mixture, thus avoiding the over-oxidation of phenyl glyoxal to glyoxalic acid. Therefore, an excess amount of 2-picolinic acid (*i.e.*, 1 equiv.) ligand, instead of a catalytic amount, is required to achieve the optimal product yield. Next, phenylglyoxal **13**, isolated from the current photoredox process, could readily react with an internal alkyne for the synthesis of 1,2-naphthoquinone **16**, *via* oxidative annulation reaction^22^ (eqn (8), Scheme 4). Furthermore, reaction of phenylglyoxal **13** with MeOH was carried out under O_2_ in the absence of light, *i.e.*, under dark conditions, which leads to no formation of α-keto ester **3a** (eqn (9) and (10), Scheme 4). This result clearly demonstrates that light irradiation is required for the transformation of phenyl glyoxal to the α-keto ester product. Most probably, the transformation of phenyl glyoxal to the α-keto ester product requires the help of the copper superoxide radical, which cannot be generated in the absence of light irradiation. The superoxide radical anion was generated under visible light irradiation of Cu(i)-phenylacetylide, and is responsible for controlled aerobic oxidation of phenyl glyoxal to α-ketoesters.

**Scheme 5:**
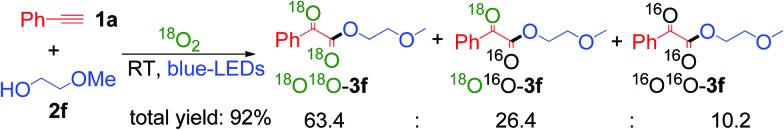
Isotopic labelling experiment.

Finally, an isotopic labeling experiment was carried out in the presence of ^18^O_2_ (98%), instead of ^16^O_2_ air, under the standard conditions (Scheme 5). ^18^O labeled α-keto esters **3f** were obtained, with a ratio of ^18^O^18^O-**3f** : ^18^O^16^O-**3f**: ^16^O^16^O-**3f** = 63.4 : 26.4 : 10.2 (Scheme S9 ESI†). These results unambiguously indicate that the oxygen atoms in the α-keto ester products mainly originate from molecular O_2_. The ^18^O^16^O-**3f** product was most probably formed *via* a partial exchange with the moisture in air or during the silica gel column purification process.^19^ It should be noted that the compounds containing 1,2-diketo groups are active, and the oxygen of carbonyl can be exchanged *via* hemiketal with the oxygen of water in air.5b,^23^

**Scheme 6:**
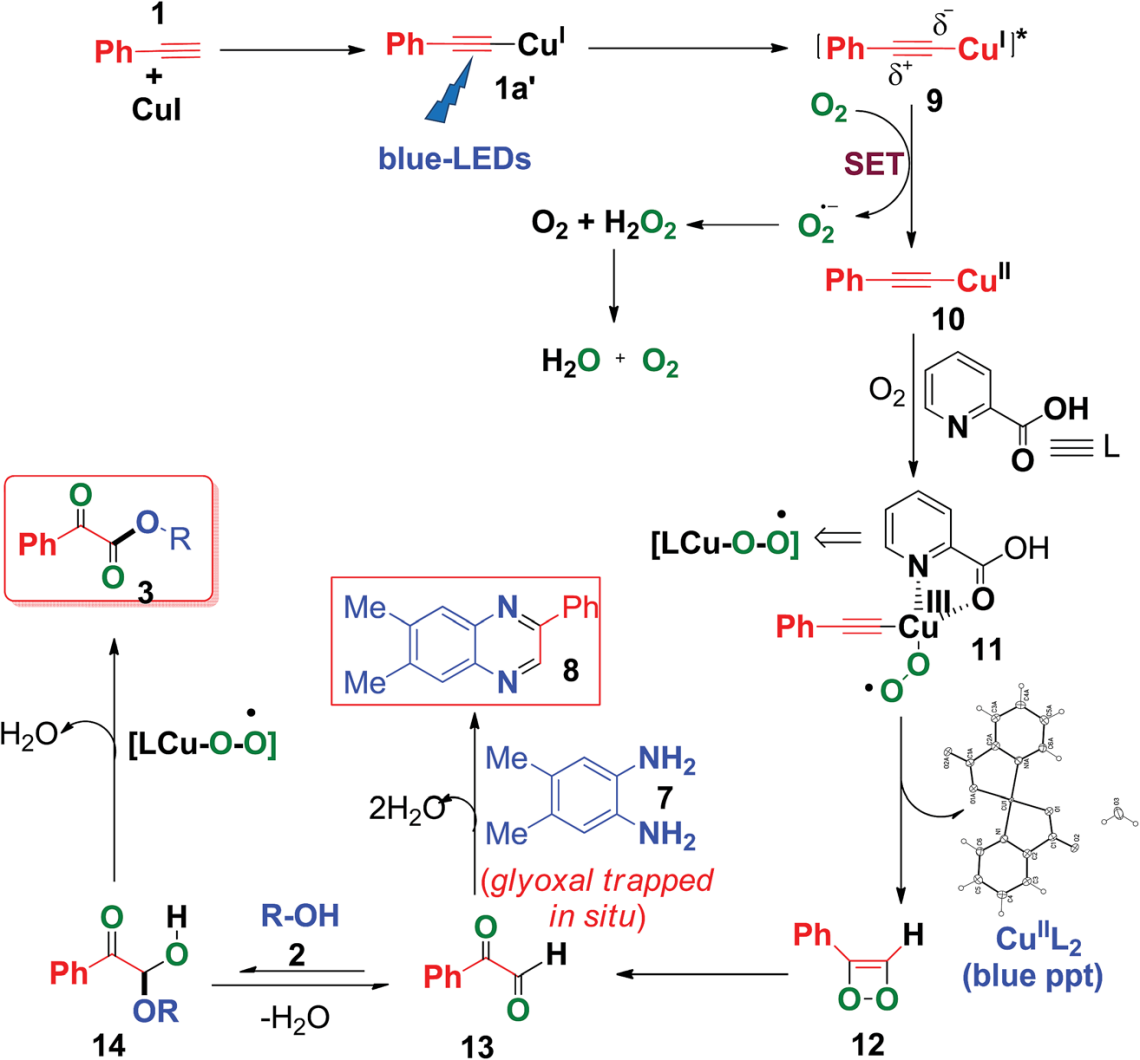
Plausible mechanism for the formation of α-keto esters.

Based on the above control experiments and our previous studies,7c,d a plausible mechanism was proposed and is shown in Scheme 6. Photoexcitation of *in situ*-generated Cu(i)-phenylacetylide (**1a′**) (UV-visible spectrum, Fig. S6, ESI†) generates a long lived (*τ* = 15.9 μs) triplet excited state Cu(i)-phenylacetylide (**9**)7c,d with partial charge separation occurring *via* ligand to metal charge transfer (LMCT).7c,d Thus the photoexcited Cu(i)-phenylacetylide then donates an electron to molecular O_2_ (*i.e.*, a SET process) to generate a superoxide radical anion (O_2_˙^–^) and an electron deficient Cu(ii)-phenylacetylide (**10**),7c,d which was confirmed by EPR measurements by using 5,5-dimethyl-1-pyrrolineN-oxide (DMPO) as a selective superoxide spin trapping reagent (Fig. S2, ESI†). Next, coordination of 2-picolinic acid (L) to Cu(ii)-phenylacetylide and subsequent reaction to molecular O_2_ results in the formation of copper(iii)-superoxo complex **11**.7c,^24^ Isomerization rearrangement of the resulting Cu(iii)-peroxo complex (**11**)occurs with concurrent formation of a C–O bond to form the intermediate (**12**).^23^ Subsequent O–O bond cleavage of the intermediate (**12**) produces 2-oxo-2-phenylacetaldehyde (**13**) and Cu^II^(pic)_2_ was eliminated as a blue ppt (Fig. S1 and S9, ESI†).^25^ Furthermore, a nucleophilic attack on **13** by alcohol **2** on the electron deficient carbonyl group affords hemiacetal intermediate **14**,9c,^11^ which further undergoes copper catalysed aerobic oxidation^26^ to produce α-keto esters (**3**). When 4,5-dimethylbenzene-1,2-diamine (**7**) was present in the reaction mixture, it trapped the *in situ*-generated phenylglyoxal **13***via* intermolecular double condensation reaction to produce 6,7-dimethyl-2-phenylquinoxaline (**8**) in a one-pot manner, as shown in Scheme 6.

In the presence of 4,5-dimethylbenzene-1,2-diamine, the formation of α-keto esters was suppressed, due to the fact that *o*-phenylene diamine acts as a better nucleophile (N is less electronegative than O) to phenylglyoxal than alcohol (**2**), thus favouring the formation of 2-phenyl quinoxaline (**8**), instead of the formation of hemiacetal (**14**).

## Conclusion

In summary, we have developed an unprecedented visible light induced copper catalyzed process for the controlled aerobic oxidation of the terminal C≡C triple bond to phenylglyoxal at room temperature, followed by esterification, for the synthesis of α-keto esters that evades the need for a base, an expensive catalyst, strong oxidants, elevated temperatures and other harsh reaction conditions. The reaction proceeds easily with excellent functional group tolerance towards the electron donating and withdrawing terminal alkynes. Moreover, it is compatible with 1°, 2°, and 3° alcohols and slightly strained or labile alcohols, which is not possible or difficult in thermal processes. The utility of this protocol has also been successfully applied for the synthesis of two biologically active molecules, *i.e.*, 1-benzyl-3-(3-nitrophenyl) quinoxalin-2(1*H*)-one (a CFTR activator) and bis oxime ester (an *E. coli* DHPS inhibitor) on a gram scale with fewer steps and higher total yields than those in the literature reported processes. We have also demonstrated the one-pot synthesis of a pharmacologically active heterocyclic compound, *i.e.*, 2-phenyl quinoxaline (an FLT3 inhibitor) *via* an unprecedented photoredox copper catalyzed process, as well as the synthesis of naphthoquinone using phenylglyoxal isolated from the current photoredox process.

## Conflicts of interest

There are no conflicts to declare.

## Supplementary Material

Supplementary information

Crystal structure data
