# Supplementary material for: Copper catalyzed photoredox synthesis of α-keto esters, quinoxaline, and naphthoquinone: controlled oxidation of terminal alkynes to glyoxals
Source: Chem Sci. 2018 Aug 29;9(37):7318–26. doi: 10.1039/c8sc03447h (PMC6167948; doi:10.1039/c8sc03447h)

Electronic Supplementary Material (ESI) for Chemical Science.
This journal is © The Royal Society of Chemistry 2018

Supporting Information

Copper Catalyzed Photoredox Synthesis of α-Keto Esters, Quinoxaline, Naphthoquinone: Controlled Oxidation of Terminal Alkynes to Glyoxals

Deb Kumar Das,‡ V. Kishore Kumar Pampana‡ and Kuo Chu Hwang*

‡These authors contributed equally.

Department of Chemistry, National Tsing Hua University, Hsinchu, Taiwan, R. O. C.
E-mail: kchwang@mx.nthu.edu.tw

Table of contents

Experimental section	S2
General Procedure for the formation of *α*-keto esters	S2-S3
Competition reaction of phenyl acetylene with 1°, 2° and 3° alcohols	S3-S4
Experimental procedures for preparation of biological active compounds **6n** and **5t**	S4-S7
Synthesis of methyl 2-(3-nitrophenyl)-2-oxoacetate (**4n**) in a gram scale	S7
Evaluagtion of Green metrics of the current photochemical process	S8
Evaluation of Green metrics of the reported thermal process	S9-10
Medicinal applications of *α*-keto esters **4u, 4v, 4x**	S11
Mechanistic pathways comparison for the formation of 3-phenyl quinaxoline-2-ol (previous work) and 2-phenyl quinaxoline (current work)	S12
Preparation of copper(I) phenylacetylide	S13
EPR spectra	S13-S16
Excitation/emission spectra of Cu(I)-phenylacetylide	S17
$^{18}O_2$-labeling experiments	S17-S18
References	S19
^{1}H NMR, ^{13}C NMR and HRMS data	S20-S39
^{1}H NMR and ^{13}C NMR spectra	S38-S85
ORTEP diagram and X-Ray Data of compound **4n**	S86
ORTEP diagram and X-Ray Data of compound **6n**	S93
ORTEP diagram of compound bis picolinate Cu(II) complex	S102

Experimental section

General: All reactions were conducted in oven-dried glasswares. All reactions were conducted using a blue light-emitting diode (LED) array (30 LEDs, power density: 40 mW/cm^2 at 460 nm)

as the visible-light source under an oxygen (O_2, 1 atm) atmosphere. All solvents were dried according to known methods and distilled prior to use. Starting materials were commercially available (Sigma-Aldrich or Alfa-Aesar or TCI chemicals) and used as received. ^{1}H NMR and ^{13}C NMR spectra were recorded at 400 and 600 MHz using deuterated $CDCl_3$ or $CDCl_3$-DMSO-d_6 mixture. Chemical shifts (δ) were reported as parts per million (ppm) and the following abbreviations were used to identify the multiplicities: s= singlet, d= doublet, t= triplet, q= quartet, m= multiplet, b= broad, and all combinations thereof can be explained by their integral parts. Unless otherwise specified, the proton/carbon signal of these 2 solvent peaks (at δ 7.24 or 2.50 and δ 77.00 or 39.51 ppm, respectively) was used as the internal reference. EPR spectra were recorded using a Bruker ESP-300E instrument.

General procedure for the formation of α-keto esters

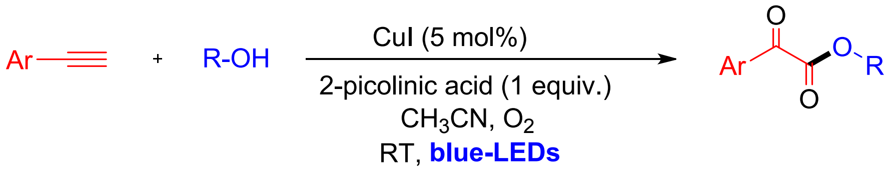

A dry test tube (20 mL) containing 5 mol% CuI (5 mg) and 0.5 mmol of 2-piconilic acid (61 mg), was added 2 mL of dry CH_3CN, aliphatic alcohol (2 mL) and terminal acetylene (0.50 mmol) *via* syringe. For low boiling aliphatic alcohols, such as MeOH, EtOH, propanol, isopropanol, n-butanol and tertiaty butanol (4 mL), was used as both reactant and solvent. The reaction mixture was then irradiated with blue LEDs (40 mW/cm^2 at 460 nm) under an oxygen atmosphere (1 atm.) at room temperature (25-28 ^{o}C) until completion of the reaction (monitored by TLC). The reaction mixture was diluted with 40 % ethyl acetate in hexane and stirred for 10 min. The mixture was filtered through celite and silica gel pads, and washed with ethyl acetate. The filtrate was concentrated and the residue was purified by column chromatography on silica gel to collect the α-keto ester as product.

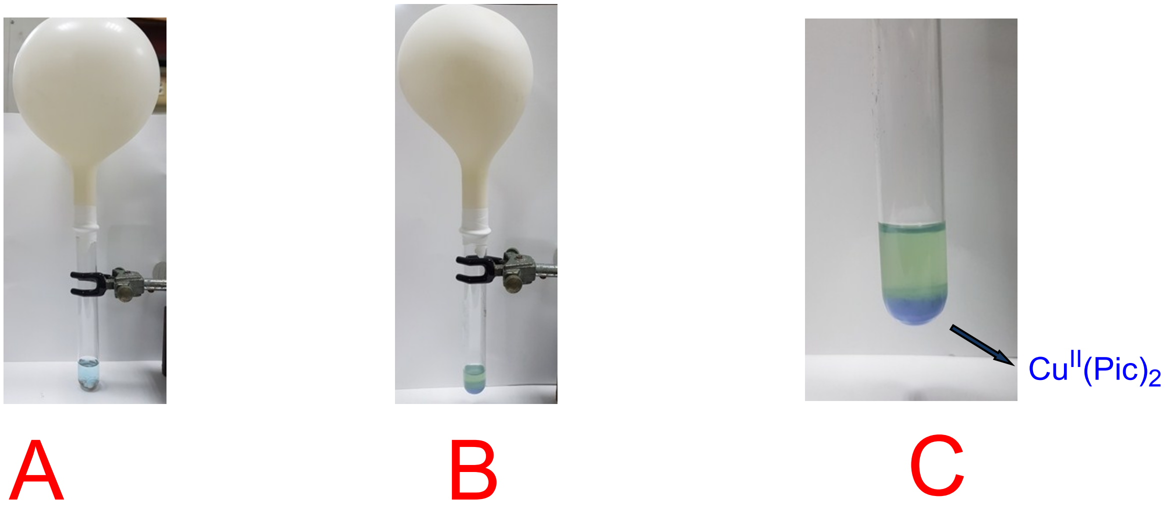

Figure S1. Optical pictures of reaction mixture before irradiation (**A**) and after irradiation (**B & C**).

Competitive reaction of phenyl acetylene with 1º, 2º and 3º alcohols

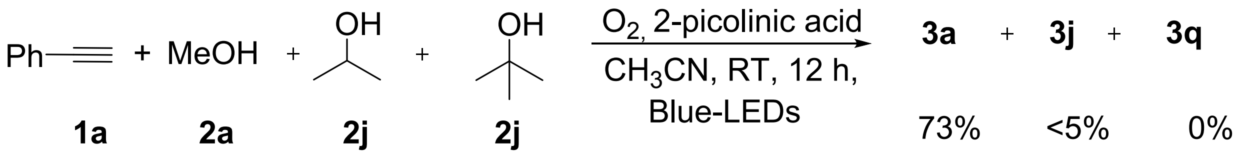

Procedure of competitive reaction: A dry test tube (20 mL) containing 5 mol% CuI (9.5 mg) and 1 mmol of 2-piconilic acid (123 mg), was added 4 mL of dry CH_3CN, MeOH (1.0 mmol, 40 µL), isopropanol (1 mmol, 76 µL) and tertiaty butanol (1 mmol, 96 µL) and phenyl acetylene (1.0 mmol) *via* syringe. The reaction mixture was then irradiated with blue LEDs (40 mW/cm^2 at 460 nm) under an oxygen atmosphere (1 atm.) at room temperature (25-28 ºC) until completion of the reaction (monitored by TLC). The reaction mixture was diluted with 40 % ethyl acetate in hexane and stirred for 10 min. The mixture was filtered through celite and silica gel pads, and washed with ethyl acetate. The filtrate was concentrated and the residue was purified by column chromatography on silica gel to collect the α-keto ester **3a** as major product in 73% yield derived from 1º alcohol i.e., MeOH. Product **3j** derived from 2º alcohol was formed in trace quantity, however we did not observe α-keto ester **3p** resulting from tertiary butanol.

Experimental procedure for the synthesis of biologically active compounds:

Preparation of Methyl 2-(3-nitrophenyl)-2-oxoacetate (4n):

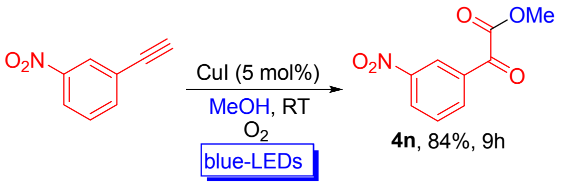

A dry test tube (20 mL) containing 5 mol% CuI (5 mg) and 0.5 mmol of 2-piconilic acid (61 mg), was added 4 mL of dry CH_3OH, and 3-nitrophenylacetylene (75 mg, 0.50 mmol) *via* syringe. The reaction mixture was then irradiated with blue LEDs (40 mW/cm^2 at 460 nm) under an oxygen atmosphere at room temperature (25-28 ^{o}C) until completion of the reaction (monitored by TLC). The reaction mixture was diluted with 40 % ethyl acetate in hexane and stirred for 10 min. The mixture was filtered through celite and silica gel pads, and washed with ethyl acetate. The filtrate was concentrated and the residue was purified by column chromatography on silica gel (eluent: petroleum ether/ethyl acetate = 4 : 1) to afford 88 mg of **4n** (84%) as solid product.

Preparation of 1-benzyl-3-(3-nitrophenyl)quinoxalin-2(1H)-one (6n):[s1]

The above collected solid (**4n**) (84 mg, 0.4 mmol) was mixed with o-phenylenediamine (34 mg, 0.4 mmol) in toluene (8 mL) and heated at 70 °C overnight. The precipitate that formed was collected by filtration, as yellow solid. The yield of product **5n** after filtration was found to be 75% (80 mg, 0.3 mmol) and used in the next step without purification. Compound **5n** (0.3 mmol) was dissolved in DMF (10 mL), benzyl bromide (0.45 mmol) and K_2CO_3 (0.6 mmol) were added, and the mixture was stirred overnight. The solution was diluted with water and extracted with ethyl acetate. The organic layer was washed with water three times. The organic layer was washed with brine and dried over magnesium sulfate. The final product **6n** obtained in 70% yield after solvent evaporation was purified by column chromatography on silica gel (eluent: petroleum ether/ethyl acetate = 5 : 1).

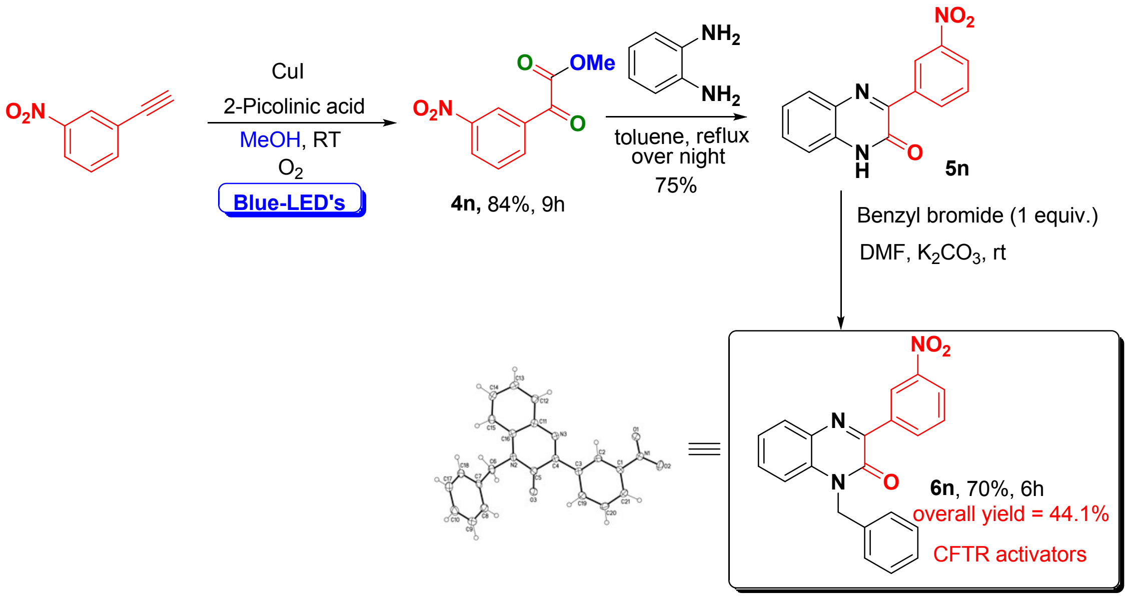

O_2N
CuI
2-Picolinic acid
MeOH, RT
O_2
Blue-LED's
O
OMe
O_2N
O
4n, 84%, 9h
NH_2
NH_2
toluene, reflux
over night
75%
NO_2
N
N
H
O
5n
Benzyl bromide (1 equiv.)
DMF, K_2CO_3, rt
NO_2
N
N
O
6n, 70%, 6h
overall yield = 44.1%
CFTR activators

Scheme S1. Synthetic comparison of the CFTR activator **(6n)** with literature[s1] and the current method.

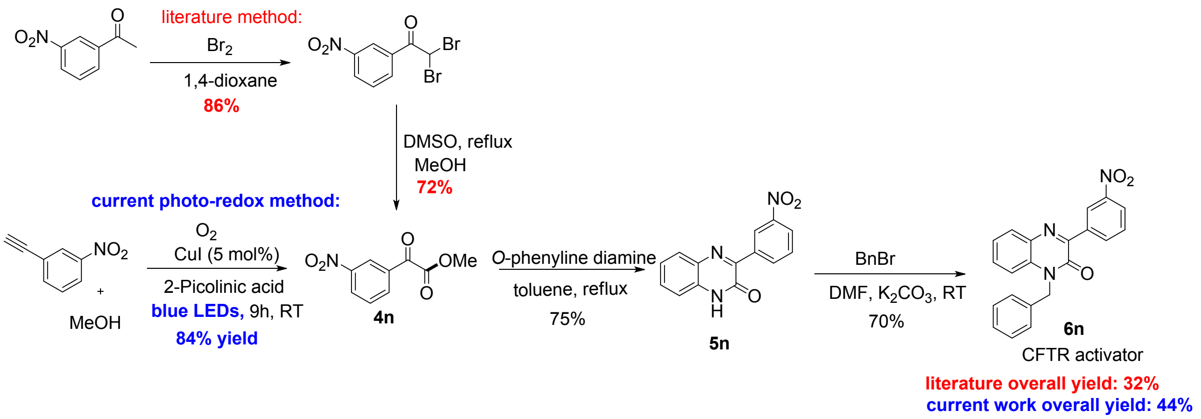

Preparation of dimethyl 2,2'-(1,3-phenylene)bis(2-oxoacetate)(4u): A dry test tube (20 mL) containing 5 mol% CuI (5 mg) and 0.5 mmol of 2-piconilic acid (61 mg), was added 4 mL of dry CH_3OH, and 1,3-diethynylbenzene (63 mg, 0.50 mmol) via syringe. The reaction mixture was then irradiated with blue LEDs (40 mW/cm^2 at 460 nm) under an oxygen atmosphere at room temperature (25-28 ^oC) until completion of the reaction (monitored by TLC). The reaction mixture was diluted with 40 % ethyl acetate in hexane and stirred for 10 min. The mixture was filtered through celite and silica gel pads, and washed with ethyl acetate. The filtrate was concentrated and the residue was purified by column chromatography on silica gel (eluent: petroleum ether/ethyl acetate = 5 : 1) to afford 116 mg of **4t** (93%) as a colourless liquid.

Preparation of dimethyl 2,2'-(1,3-phenylene)bis(2-(hydroxyimino)acetate) (5t);[s2] To a solution of **4t** (116 mg, 0.46 mmol) in dry MeOH (15 mL) was added hydroxylamine hydrochloride (69 mg, 1 mmol) and pyridine (2 drops). The reaction was stirred under nitrogen at room temperature overnight. Then the mixture was concentrated in vacuo and re-suspended between HCl solution (1 M, 10 mL) and EtOAc (15 mL). The organic phase was separated and further washed with HCl solution (1 M, 1 x 10 mL), then dried ($MgSO_4$), filtered and concentrated in vacuo to give **4t** as an off-white solid (82 mg, 64% yield).

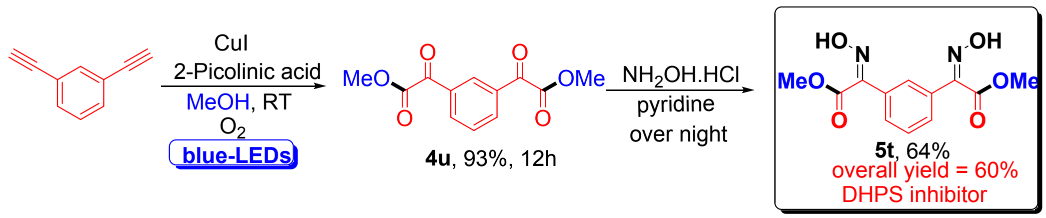

Scheme S2. Synthetic comparison of DHPS inhibitor (**5t**) with literature[s2] and current photo-redox method.

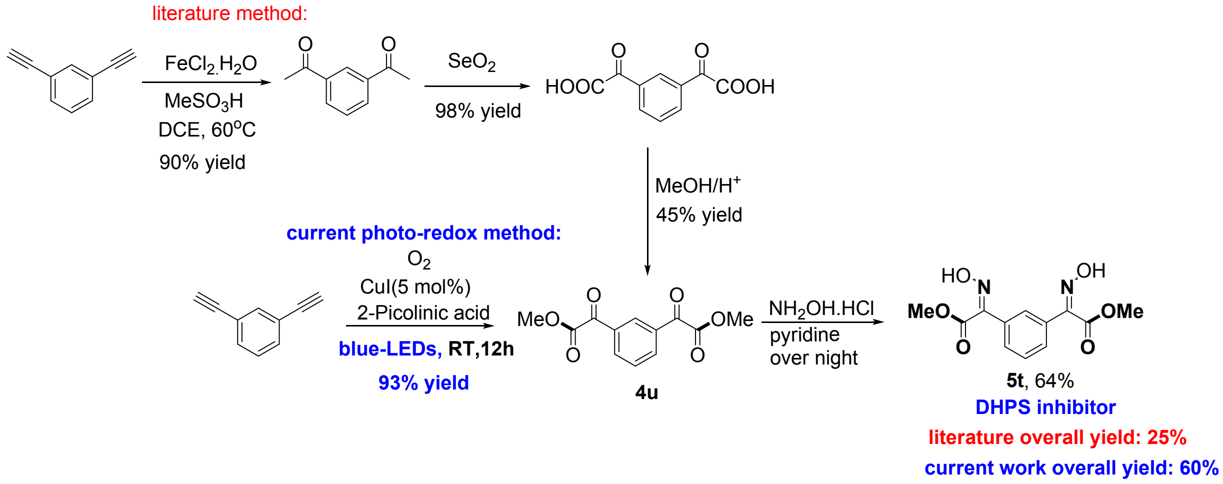

*Synthesis of Methyl 2-(3-nitrophenyl)-2-oxoacetate (**4n**) in gram scale:* To a flame-dried round bottom flask (100 mL) containing 5 mol% CuI and 7.0 mmol of 2-piconilic acid (861 mg), was added 56 mL of dry CH_3OH, and 3-nitrophenylacetylene (1.03 g, 7.0 mmol) *via* syringe. The reaction mixture was then irradiated with blue LEDs (40 mW/cm^2 at 460 nm) under an oxygen atmosphere at room temperature (25-28 °C) until completion of the reaction (monitored by TLC). After the reaction was complete, methanol was removed under reduced pressure and the reaction mixture was diluted with 40 % ethyl acetate in hexane and stirred for 10 min. The mixture was filtered through celite and silica gel pads, and washed with ethyl acetate. The filtrate was concentrated and the residue was purified by column chromatography on silica gel (eluent: petroleum ether/ethyl acetate = 4 : 1) to afford 1.16 mg of **4n** (79%) as solid product.

Evaluation of Green metrics of the current photochemical process

Scheme S3. Current photochemical process for the preparation of α-ketoester (**4n**)

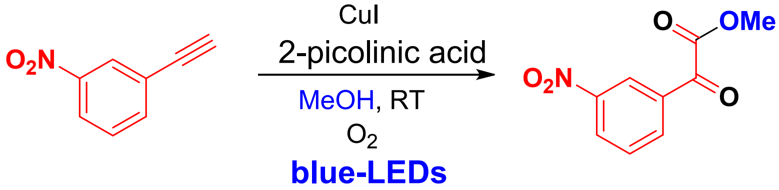

Reactant 1	1-ethynyl-3-nitrobenzene	1.029 g	7.0 mmol	F.W = 147.02
Reactant 2	MeOH	0.224 g	7.0 mmol	F.W = 32.02
Ligand	2-picolinic acid	0.861 g	7.0 mmol	F.W = 123.02
solvent	MeOH (56 mL) (d = 0.792 g/mL	44.128 g		
Auxiliary	------	------	-----	------
Recycled solvent	MeOH (40 mL)	31.6 g		
Product	Methyl 2-(3-nitrophenyl)-2-oxoacetate	1.156 g	5.53 mmol	F.W = 209.03

Product yield = 79%

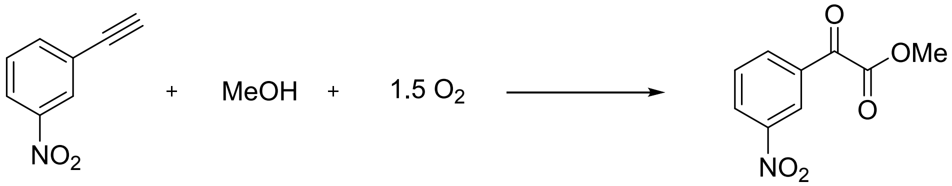

Atom economy defined as "how much of the reactants remain in the final desired product"

$$\text{Atom economy (AE)} = \frac{\text{Molecular mass of desired product}}{\text{Molecular mass of all reactants}} \text{ X } 100$$

$$\text{Atom efficiency} = \frac{79\% \text{ x } 92\%}{100} = 72.7\%$$

$$\text{Atom economy} = \frac{209}{147 + 32 + 48} = 92\%$$

$$\text{E-factor} = \frac{\text{Amount of waste}}{\text{Amount of product}}$$

$$\text{E-factor} = \frac{1.029 + 0.224 + 0.861 + 44.128 - 1.156 - 31.6}{1.156} = 11.66 \text{ Kg waste per Kg product}$$

Reaction mass efficiency defined as "the percentage of the mass of the reactant that remain in the product."

$$\text{Reaction mass efficiency (RME)} = \frac{\text{Molecular mass of desired product}}{\text{Molecular mass of all reactants}} \text{ X } 100$$

$$\text{Reaction mass efficiency (RME)} = \frac{1.156}{1.029 + 0.224} \text{ X } 100 = 92.2\%$$

$$\text{Carbon efficiency} = \frac{9}{8 + 1} \text{ X } 100 = 100\%$$

Evaluation of Green metrics of the reported thermal process

Scheme S4. Reported thermal procedure for preparation of α-keto ester (**4n**).

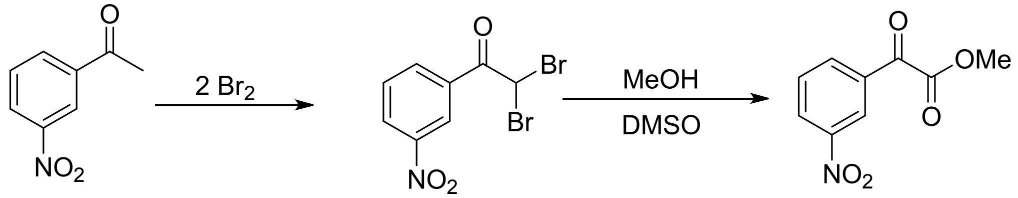

Reactant 1	1-(3-nitrophenyl)ethan-1-one	1.15 g	7.0 mmol	F.W = 165.04
Reactant 2	Br_2	2.48 g	15.75 mmol	F.W = 157.84
solvent	Dioxane (87.5 mL) (d = 1.03 g/mL	90 g		
Auxiliary	------	------	-----	------
Product	2,2-dibromo-1-(3-nitrophenyl)ethan-1-one	1.931 g	6.02 mmol	F.W = 320.86

Product yield = 86%

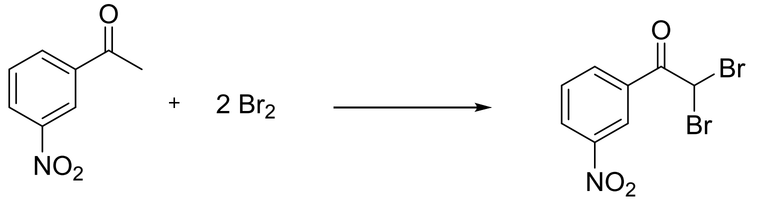

$$\text{E-factor} = \frac{1.15 + 2.48 + 90 - 1.931}{1.931} = 47.48 \text{ Kg waste per Kg product}$$

$$\text{Atom economy} = \frac{320.86}{165.02 + 2 \times 157.84} \times 100 = 67\%$$

$$\text{Atom efficiency} = \frac{86\% \times 67\%}{100} = 57.62\%\%$$

$$\text{Carbon efficiency} = \frac{8}{8} \text{ X } 100 = 100\%$$

$$\text{Reaction mass efficiency (RME)} = \frac{1.931}{1.15 + 2.48} \text{ X } 100 = 53.2\%$$

Reactant 1	2,2-dibromo-1-(3-nitrophenyl)ethan-1-one	2.24 g	7.0 mmol	F.W = 320.86
Reactant 2	MeOH	0.372 g	11.62 mmol	F.W = 32
Reactant 3	DMSO (116.2 mL) (d = 1.1 g/mL	127.82 g	---	F.W = 78.13
Auxiliary	------	------	-----	------
Product	methyl 2-(3-nitrophenyl)-2-oxoacetate	1.053 g	5.04 mmol	F.W = 209.03

Product yield = 72%

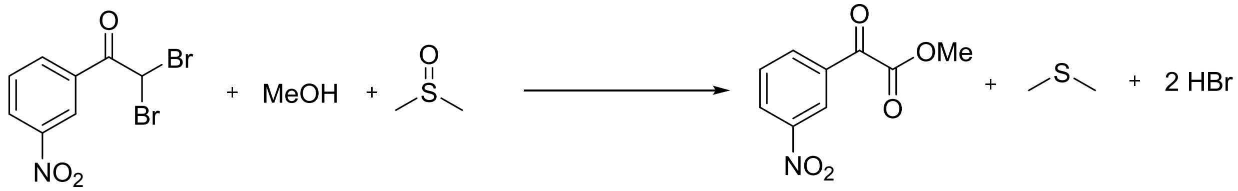

$$\text{E-factor} = \frac{2.24 + 0.372 + 127.82 - 1.053}{1.053} = 122.9 \text{ Kg waste per Kg product}$$

$$\text{Atom economy} = \frac{209.03}{320 + 32 + 78} \times 100 = 48.6\%$$

$$\text{Atom efficiency} = \frac{72\% \times 48.6\%}{100} = 35\%\%$$

$$\text{Carbon efficiency} = \frac{9}{8 + 1} \text{ X } 100 = 100\%$$

Scheme S5. Medicinal applications of α-keto esters **4u, 4v, 4x:**

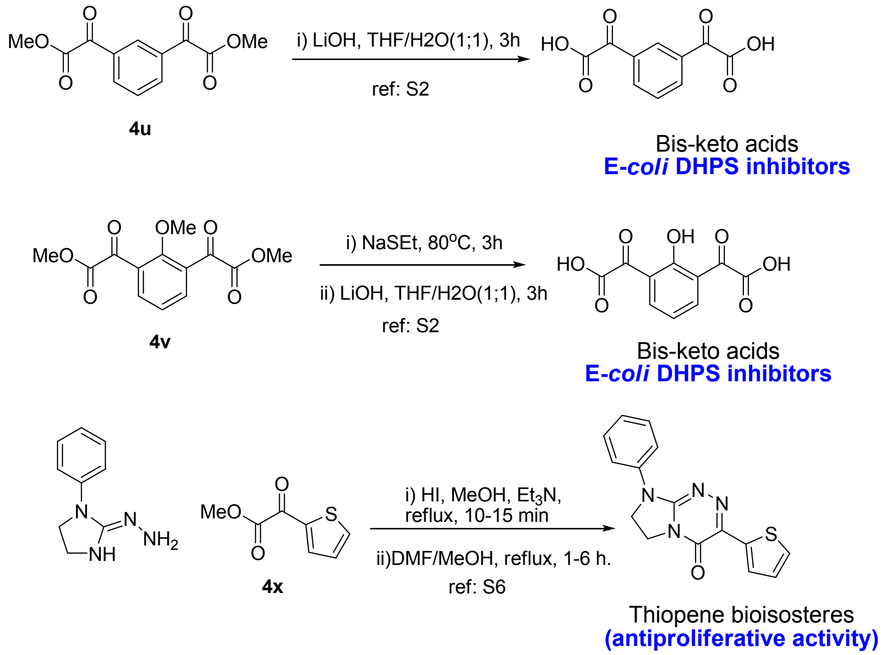

General procedure for the formation of 6,7-dimethyl-2-phenylquinoxaline ***(8).***

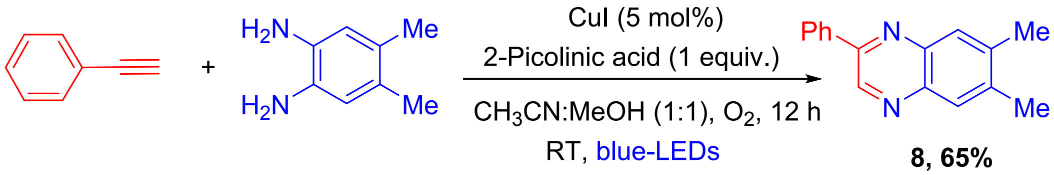

A dry test tube (20 mL) containing 5 mol% CuI (5 mg) and 0.5 mmol of 2-piconilic acid (61 mg), was added 2 mL of dry CH_3CN, MeOH (2 mL) and terminal acetylene (0.50 mmol) via syringe, then 4,5-dimethylbenzene-1,2-diamine (0.50mmol) was added to the reaction mixture which was irradiated with blue LEDs (40 mW/cm^2 at 460 nm) under an oxygen atmosphere (1 atm.) at room temperature (25-28 ^{o}C) until completion of the reaction (monitored by TLC). The reaction mixture was diluted with 40 % ethyl acetate in hexane and stirred for 10 min. The mixture was filtered through celite and silica gel pads, and washed with ethyl acetate. The filtrate was concentrated and the residue was purified by column chromatography on silica gel to afford 67 mg of 6,7-dimethyl-2-phenylquinoxaline **8** (65%) as yellow solid product.

Scheme S6. Previous literature thermal method for synthesis of 2-phenyl quinaxoline (**8**)[S8a].

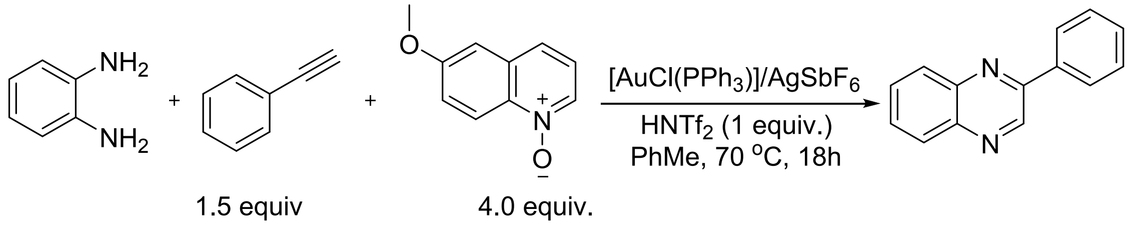

- Requires expensive gold and silver catalysts
- Need of strong external oxidant pyridine N-oxide
- Requires excess amount of external oxidant (4 equiv.) and acid additive
- Harsh reaction condition and longer reaction time.

Scheme S7. Comparison of mechanistic pathways for the formation of 3-phenyl quinaxoline-2-ol (previous work)[s7] and 2-phenyl quinaxoline (current work).

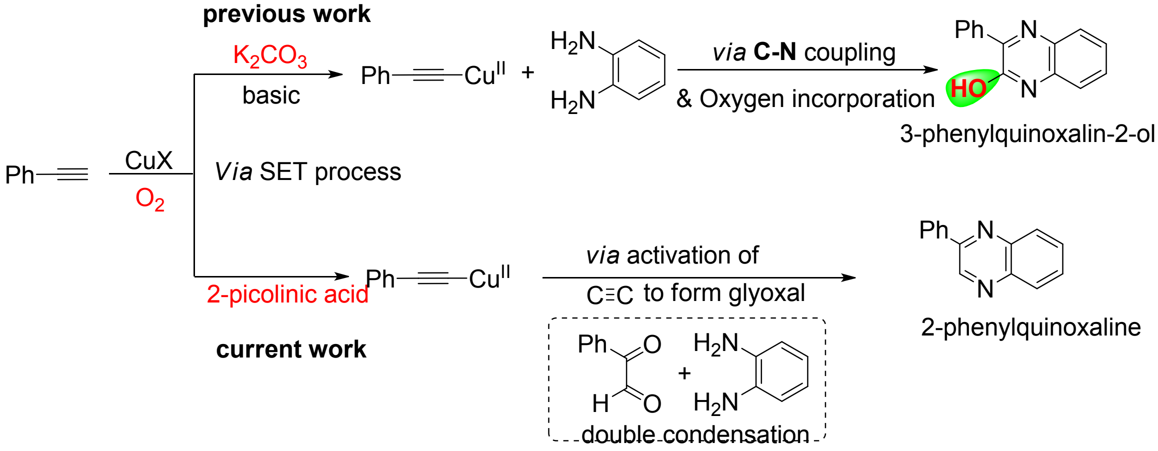

Plausible mechanism for 3-phenyl quinaxoline-2-ol proceeds via C-N coupling under basic condition and in the absence of 2-picolinic acid ligand, whereas formation of 2-phenyl quinaxoline is going through visible light assisted control oxidation of phenylacetylene to phenylglyoxal in the presence of 2-picolinic acid ligand, which later double condenses with diamine.

Scheme S8. Plausible mechanism for formation of 3-phenyl quinaxoline-2-ol.[7g]

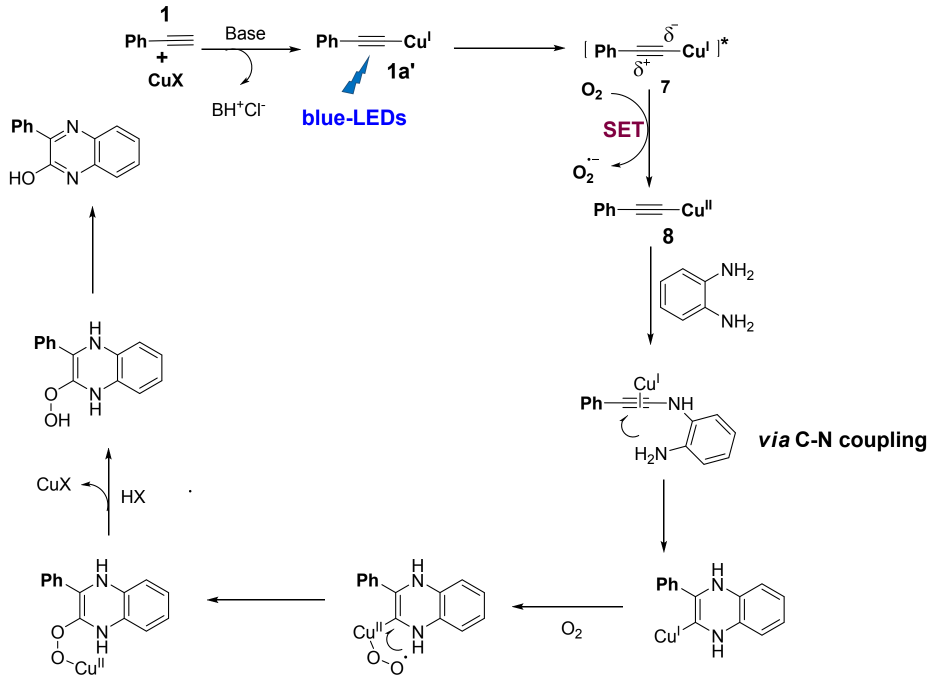

Preparation of copper(I) phenylacetylide:[s3] CuI (1.0 g, 5.0 mmol) was dissolved in ammonium hydroxide to form a blue solution. While stirring, phenylacetylene (0.5 g, 5.1 mmol in 50 mL ethanol) was added dropwise to the solution. The system was allowed to stand for 15 min to form a yellow precipitate suspension. The precipitate was filtered out and washed with water, ethanol, and diethyl ether, three times each. The solid was vacuum-dried, and 0.65 g (75% yield) of a bright yellow solid was obtained. The spectroscopic data for the yellow solid are shown below: FT-IR (KBr, cm^{-1})[s4:] 1929 (C≡C), 1596, 1568; UV-Vis: λ_{abs}= 476 nm.

EPR measurements: EPR spectra were recorded at room temperature on a Bruker ESP-300E (X band, 9.8 GHz) with parameters setting as shown below: receiver gain= 30 n; receiver phase= 0 deg; receiver harmonic= 1; field modulation frequency=100000 Hz; microwave frequency [Hz]= 9.660469 e^{+09}; field modulation amplitude [T]= 0.00016; receiver time constant [S] = 0.32768; microwave power= 0.015 W; receiver offset [%FS]= 0; DMPO (5-,5-dimethyl-1-pyrroline N-oxide) was employed as a radical trap for trapping of the superoxide radical anion.

The reaction under a standard condition phenyl acetylene (**1a**), MeOH (**2a**), CuI, 2-picolinic acid, 1 atm. O_2 in CH_3CN was irradiated with blue LEDs for 20 min in the presence of DMPO in an EPR chamber while recording the EPR spectra. The EPR signals shown in Figure **S1** is corresponding to DMPO-OO(H). Next, under the standard condition copper(I) phenylacetylide

(**1a'**) (1 atm. O_2) was irradiated under blue LEDs for 20 min in the presence of EPR trapping reagent DMPO. The EPR signals shown in Figure **S3** is corresponding to DMPO-OO(H) which shows that superoxide anion radical was formed in the reaction solution. No superoxide EPR signal was observed from the reaction solution under the standard condition in the absence of CuI or O_2 (Figures S2 & S4). These results indicate that copper(I) phenylacetylide undergoes single electron transfer to O_2, and generates superoxide free radical upon blue LEDs irradiation.[s5]

EPR spectra of the **reaction mixture after blue LEDs irradiation**

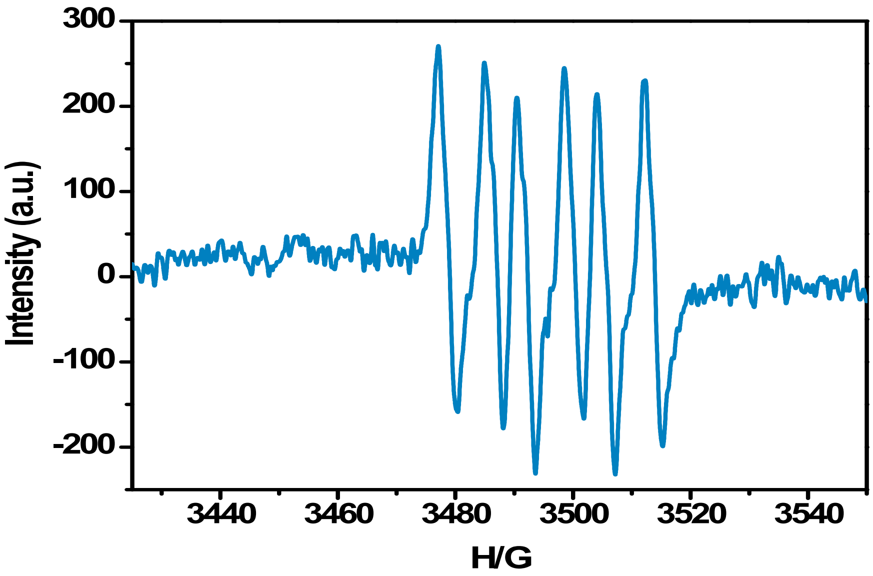

Figure S2: EPR spectra of the reaction mixture: phenyl acetylene (**1a**) (0.1 mmol), MeOH (**2a**) (1 mL), 5 mol% CuI and 2-picolinic acid (0.1 mmol), in 1 mL of CH_3CN 1 atm. O_2, 0.5 mL of this reaction solution was taken out into a small vial, followed by the addition of 0.01 mL of DMPO (5 x 10^{-2} M). The mixture was irradiated with blue LEDs at room temperature under an oxygen atmosphere (1 atm.) for 20 minutes. The reaction mixture was then analysed by EPR spectra. The classical 6 EPR peaks are originated from the DMPO-OO· radical species.

EPR spectra of the reaction mixture **without CuI**

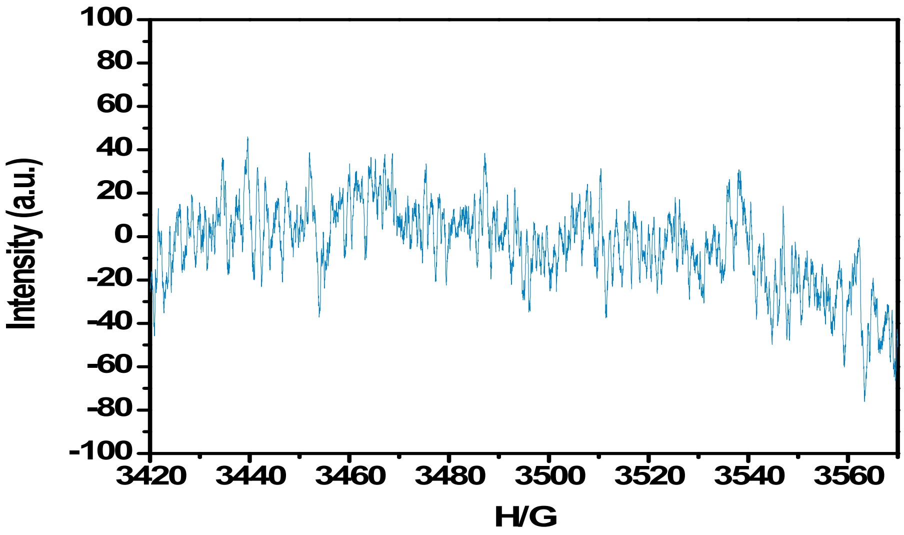

Figure S3: EPR spectra of the reaction mixture: phenyl acetylene (**1a**) (0.1 mmol), MeOH (**2a**) (1 mL) and 2-picolinic acid (0.1 mmol), in 1 mL of CH_3CN 1 atm. $O_{2,}$ 0.5 mL of this reaction solution was taken out into a small vial, followed by the addition of 0.01 mL of DMPO (5 x 10^{-2} M). The mixture was irradiated with blue LEDs at room temperature under an oxygen atmosphere (1 atm.) for 20 minutes (*in the absence of CuI*). The reaction mixtures were analysed by EPR spectra. No signals were detected.

EPR spectra of copper (I) phenylacetylide (1a') with O_2 under blue-LEDs

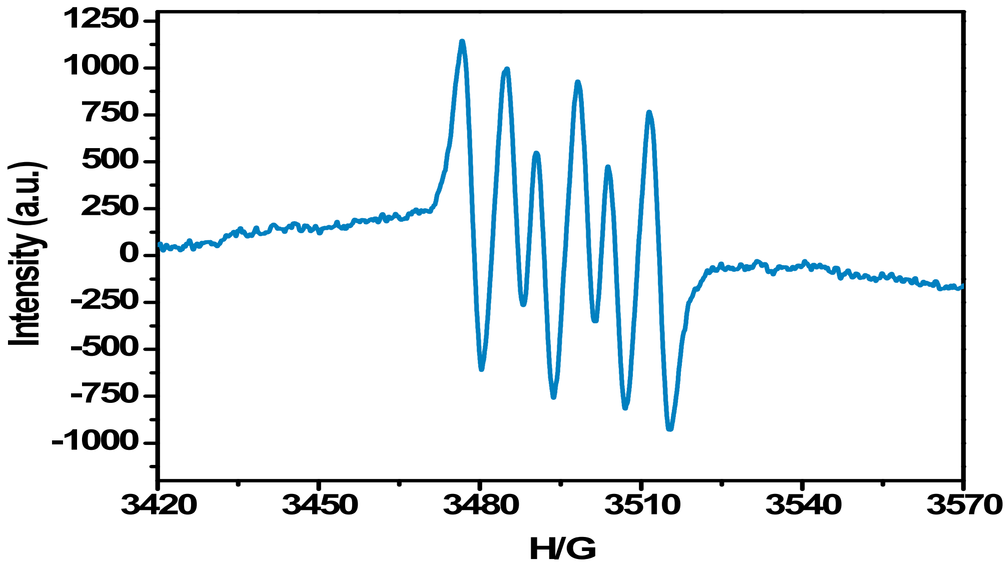

Figure S4: EPR spectra of the reaction mixture: 10 mg of copper (I) phenylacetylide in 7 mL of CH_3CN under. 0.5 mL of this reaction solution was taken out into a small vial, followed by the addition of 0.01 mL of DMPO (5 x 10^{-2} M). The mixture was irradiated with blue LEDs at room temperature under an oxygen atmosphere for 20 minutes. The reaction mixtures were analysed by EPR spectra. There are 6 classical peaks, which are corresponding to the signals (DMPO-OO(H)). There are classical 6 peaks, the signals corresponding to the DMPO-OO· radical species.

EPR spectra of the reaction mixture without $\mathbf{O_2}$ (under N_2) at RT

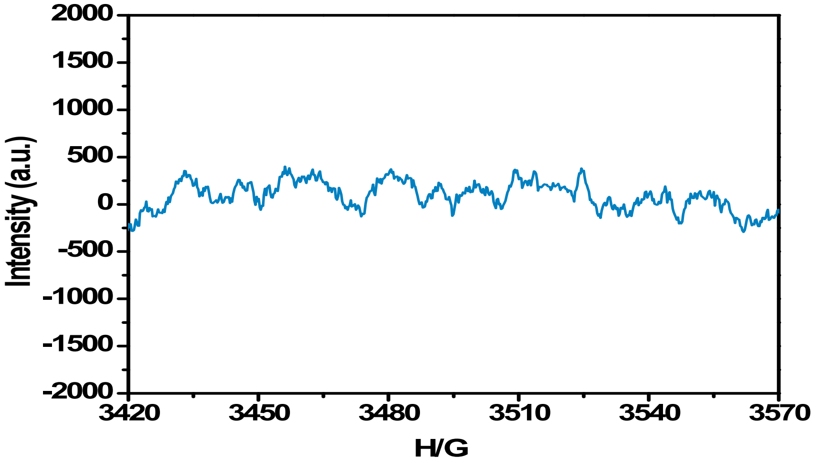

Figure S5: EPR spectra of the reaction mixture: phenylacetylene (**1a**) (0.11 mmol), and 5 mol% of CuI in CH_3CN purged with N_2 (without O_2). 0.5 mL of this reaction solution was taken out into a small vial, followed by the addition of 0.01 mL of DMPO (5 x 10^{-2} M). The mixture was irradiated with blue LEDs at room temperature under nitrogen atmosphere (1 atm.) for 20 minutes (in the absence of O_2). The reaction mixture was analysed by EPR spectra. No signals were detected.

Excitation and emission spectra of copper(I) phenylacetylide:

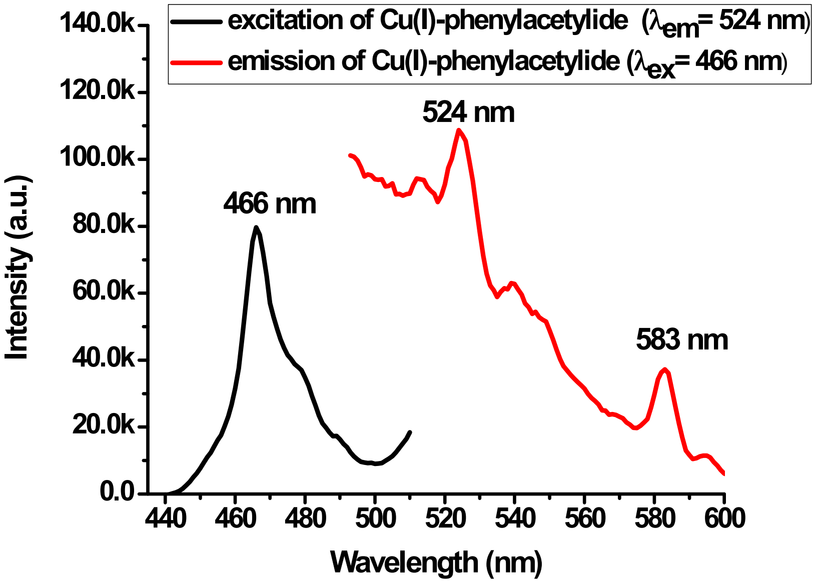

Figure S6: Excitation and emission spectra of in-situ generated copper(I) phenylacetylide in CH_3CN solvent.

***$^{18}O_2$ labeling experiments*:** We have performed an $^{18}O_2$-labeling experiment under the standard condition (98% purity of $^{18}O_2$ gas, instead of $^{16}O_2$ air, was filled in the reaction system). From ESI mass, the final product **3f** was determined to contain an ^{18}O labeled α-keto ester **3f**, 63.4% exclusively, indicating that the oxygen atom in the α-keto ester originated from molecular O_2. The $^{18}O^{16}O$-**3f** product is most probably formed *via* a partial ^{18}O-$H_2{}^{16}O$ exchange in air/moisture or during the silica gel column purification. It should be noted that the 1,2-diketo containing analogues are very active, and the oxygen of carbonyl can be exchanged *via* hemiketal with the oxygen of water in air.

Scheme S9. $^{18}O_2$ isotope labelling experiments.

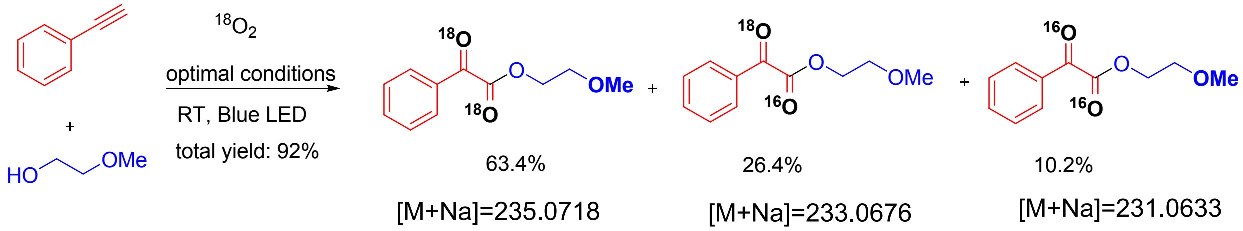

Display Report

Analysis Info

Analysis Name	D:\Data\NCTU SERVICE\Data\2017\20170804\ES-O18-crude_GB6_01_14985.d	Acquisition Date	8/4/2017 11:47:42 AM
Method	Small molecule.m	Operator	NCTU
Sample Name	ES-O18-crude	Instrument	impact HD 1819696.00164
Comment			

Acquisition Parameter

Source Type	ESI	Ion Polarity	Positive	Set Nebulizer	1.0 Bar
Focus	Active	Set Capillary	4500 V	Set Dry Heater	200 °C
Scan Begin	50 m/z	Set End Plate Offset	-500 V	Set Dry Gas	6.0 l/min
Scan End	1500 m/z	Set Charging Voltage	2000 V	Set Divert Valve	Waste
		Set Corona	0 nA	Set APCI Heater	0 °C

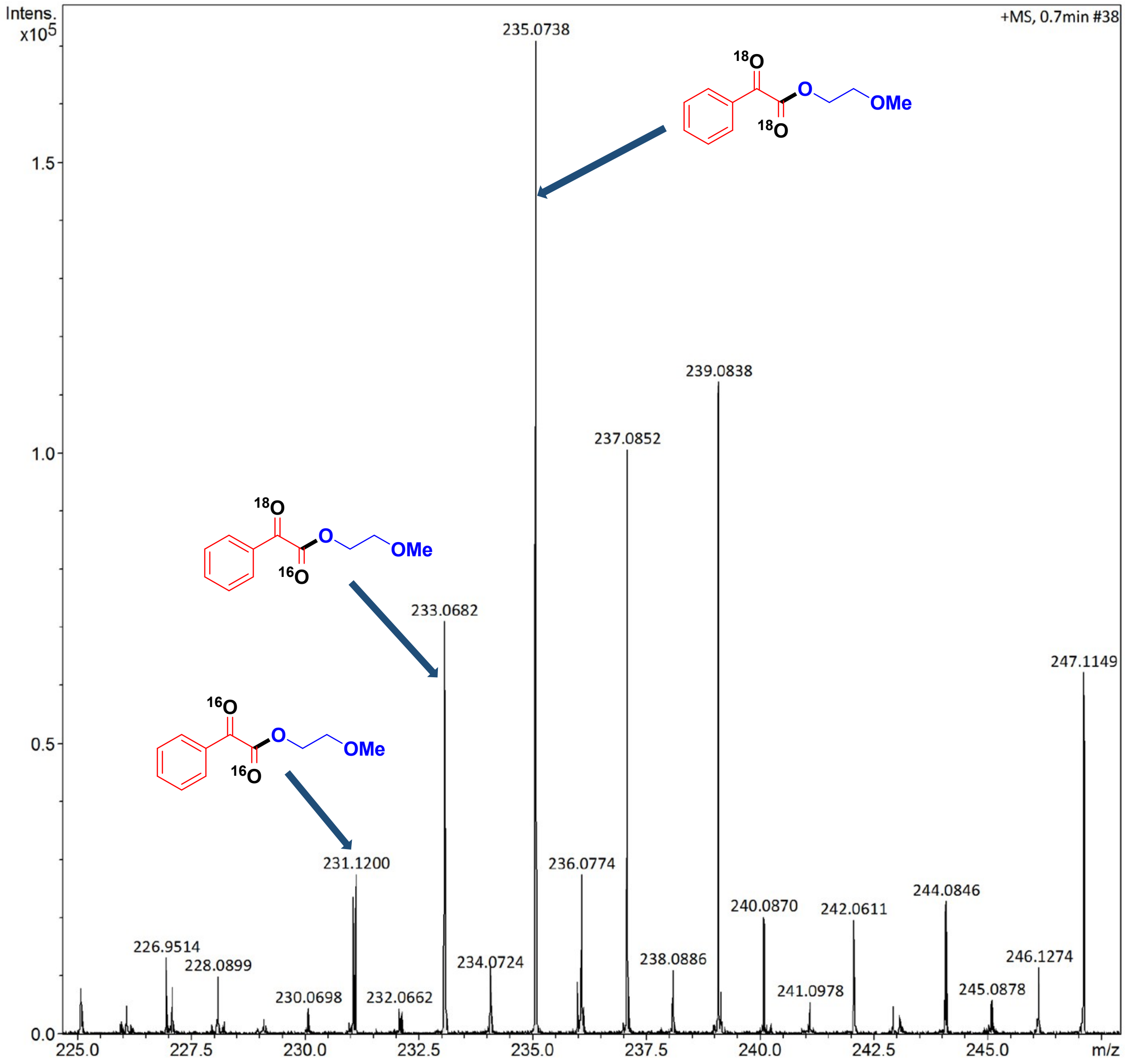

Supporting References:

S1. J.-H. Son, J. S. Zhu, P.-W. Phuan, O. Cil, A. P. Teuthorn, C. K. Ku, S. Lee, A. S. Verkman and M. J. Kurth, *J. Med. Chem*., 2017, **60**, 2401.

S2. (a) B. A. Boughton, L. Hor, J. A. Gerrard and C. A. Hutton, *Bioorg. Med. Chem*., 2012, **20**, 2419. (b) B. A. Boughton, R. C. J. Dobson, J. A. Gerrard and C. A. Hutton, *Bioorg. Med. Chem. Lett.*, 2008, **18,** 460.

S3. W. Shi, Y. Luo, X. Luo, L. Chao, H. Zhang, J. Wang and A. Lei, *J. Am. Chem. Soc.,* 2008, **130**, 14713.

S4. Y. Okamoto and S. K. Kundu, *J. Phys. Chem.,* 1973, **77**, 2677.

S5. A. Sagadevan, A. Ragupathi, C.-C. Lin, J. R. Hwu and K. C. Hwang, *Green Chem.,* 2015, **17**, 1113.

S6. M. Sztanke, J. Rzymowska and K. Sztanke, *Bioorg. Med. Chem.*, 2015, **23**, 3448.

S7. A. Sagadevan, A. Ragupathi and K.C. Hwang, *Photochem. Photobiol. Sci*., 2013, **12**, 2110.

S8. (a) S. Shi, T. Wang, W. Yang, M. Rudolph and A. S. K. Hashmi, *Chem. Eur. J*., 2013, **19**, 6576 (b) M. Antoine, M. Gerlach, E. Günther, T. Schuster, M. Czech, I. Seipelt and P. Marchand, *Synthesis*, 2012, **69**.

S9. C.-H. Hung, P. Gandeepan, L.-C. Cheng, L.-Y. Chen, M.-J. Cheng and C.-H. Cheng, *J. Am. Chem. Soc*., 2017, **139**, 17015.

Methyl 2-oxo-2-phenylacetate (3a)

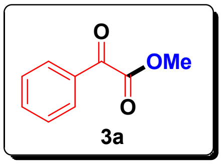

Colourless liquid; **^{1}H NMR** (600 MHz, $CDCl_3$): δ 7.99 (d, *J* = 6.0 Hz, 2 H), 7.63 (t, *J*= 6.0 Hz, 1 H), 7.48 (t, *J*= 6.0 Hz, 2 H), 3.95 (s, 3 H); **^{13}C NMR** (150 MHz, $CDCl_3$): δ 186.0, 164.0, 134.9, 132.4, 130.0, 128.8 and 52.7; IR (KBr): 2923, 1741, 1686, 1451, 1213, 1177 cm^{-1}; HRMS: calcd for $C_9H_9O_3$ (M+H): 165.0552, found: 165.0546.

Ethyl 2-oxo-2-phenylacetate (3b)

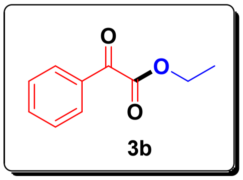

Colourless liquid; **^{1}H NMR** (600 MHz, $CDCl_3$): δ 7.98 (d, *J* = 6.0 Hz, 2 H), 7.63 (t, *J*= 6.0 Hz, 1 H), 7.48 (t, *J*= 6.0 Hz, 2 H), 4.42 (q, *J* = 6.0 Hz, 2 H), 1.39 (t, *J* = 6.0 Hz, 3 H); **^{13}C NMR** (150 MHz, $CDCl_3$): δ 186.4, 163.8, 134.9,132.4, 130.0, 128.9, 62.3 and 14.0; IR (KBr): 2957, 2927, 1728, 1691, 1597, 1451, 1203 cm^{-1}; HRMS: calcd for $C_{10}H_{11}O_3$ (M+H): 179.0708, found: 179.0707.

Propyl 2-oxo-2-phenylacetate (3c)

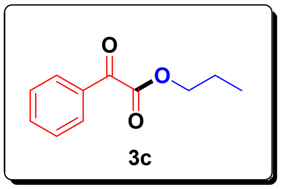

Colourless liquid; **^{1}H NMR** (600 MHz, $CDCl_3$): δ 7.97 (d, *J* = 6.0 Hz, 2 H), 7.62 (t, *J*= 12.0 Hz, 1 H), 7.48 (t, *J*= 6.0 Hz, 2 H), 4.32 (t, *J*= 12.0 Hz, 2 H), 1.79 (q, *J* = 6.0 Hz, 2 H), 0.98 (t, *J* = 6.0 Hz, 3 H); **^{13}C NMR** (150 MHz, $CDCl_3$): δ 186.4, 163.9, 134.8,132.4, 129.9, 128.8, 67.6, 21.8 and 10.2; IR (KBr): 2971, 1736, 1690, 1597, 1451, 1323, 1170, 990 cm^{-1}; HRMS: calcd for $C_{11}H_{12}O_3$ (M+H): 193.0865, found: 193.0857.

Butyl 2-oxo-2-phenylacetate (3d)

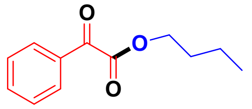

3d

Colourless liquid; **^{1}H NMR** (600 MHz, $CDCl_3$): δ 7.98 (d, *J* = 12.0 Hz, 2 H), 7.62 (t, *J*= 6.0 Hz, 1 H), 7.48 (t, *J*= 6.0 Hz, 2 H), 4.36 (t, *J*= 12.0 Hz, 2 H), 1.76-1.71 (m, 2 H), 1.45-1.39 (m,2 H), 0.93 (t, *J* = 12.0 Hz, 3 H); **^{13}C NMR** (150 MHz, $CDCl_3$): δ 186.4, 163.9, 134.8,132.4, 129.9, 128.8, 66.0, 30.4, 18.9 and 13.5; IR (KBr): 2962, 2875, 1738, 1691, 1597, 1200, 1176 cm^{-1}; HRMS: calcd for $C_{12}H_{14}O_3$ (M+H): 207.1021, found: 207.1013.

Isobutyl 2-oxo-2-phenylacetate (3e)

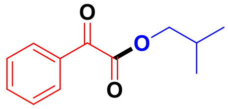

3e

Colourless liquid; **^{1}H NMR** (600 MHz, $CDCl_3$): δ 7.97 (d, *J* = 6.0 Hz, 2 H), 7.62 (t, *J*= 6.0 Hz, 1 H), 7.47 (t, *J*= 6.0 Hz, 2 H), 4.15 (d, *J*= 6.0 Hz, 2 H), 2.07-2.03 (m, 2 H), 0.97 (d, *J* = 6.0 Hz, 6 H); **^{13}C NMR** (150 MHz, $CDCl_3$): δ 186.4, 164.0, 134.8,132.4, 129.8, 128.8, 72.2, 27.6 and 18.8; IR (KBr): 2966, 1737, 1691, 1597, 1451, 1323, 1176, 1003 cm^{-1}; ESI-MS: calcd for $C_{12}H_{14}O_3$ (M+Na): 229.0841, found: 229.0835.

2-Methoxyethyl 2-oxo-2-phenylacetate (3f)

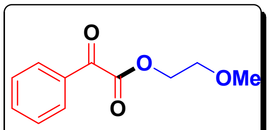

3f

Colourless liquid; **^{1}H NMR** (600 MHz, $CDCl_3$): δ 8.00 (d, *J* = 6.0 Hz, 2 H), 7.63 (t, *J*= 6.0 Hz, 1 H), 7.49 (t, *J*= 6.0 Hz, 2 H), 4.51 (t, *J* = 6.0 Hz, 2 H), 3.69 (t, *J* = 6.0 Hz, 2 H), 3.38 (s, 3 H); **^{13}C NMR** (150 MHz, $CDCl_3$): δ 186.1, 163.7, 134.9,132.3, 130.0, 128.8, 69.8, 64.7 and 58.7; IR (KBr): 2927, 1740, 1688, 1596, 1451, 1177, 1129, 1023 cm^{-1}; ESI-MS: calcd for $C_{11}H_{12}O_4$ (M+Na): 231.0633, found: 231.0628

Benzyl 2-oxo-2-phenylacetate (3g)

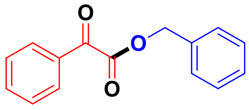

3g

Colourless liquid; **^{1}H NMR** (600 MHz, $CDCl_3$): δ 7.96 (d, *J* = 12.0 Hz, 2 H), 7.62 (t, *J*= 6.0 Hz, 1 H), 7.48-7.42 (m, 2 H), 7.39 (t, *J* = 6.0 Hz, 2 H), 7.36 (t, *J* = 6.0 Hz, 3 H), 5.40 (s, 2 H); **^{13}C NMR** (150 MHz, $CDCl_3$): δ 186.0, 163.6, 134.9,134.5, 132.4, 129.9, 128.8, 128.7, 128.5 and 67.7; IR (KBr): 1736, 1687, 1596, 1196, 1174 cm^{-1}; ESI-MS: calcd for $C_{15}H_{12}O_3$ (M+Na): 263.0679, found: 263.0677.

Cyclopropylmethyl 2-oxo-2-phenylacetate (3h)

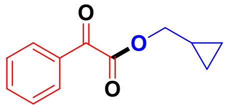

3h

Colourless liquid; **^{1}H NMR** (600 MHz, $CDCl_3$): δ 7.99 (d, *J* = 6.0 Hz, 2 H), 7.64 (t, *J*= 6.0 Hz, 1 H), 7.47 (t, *J*= 6.0 Hz, 2 H), 4.21 (d, *J*= 6.0 Hz, 2 H), 1.27-1.22 (m, 1 H), 0.64-0.61 (m, 1 H), 0.38-0.35 (m, 2 H); **^{13}C NMR** (150 MHz, $CDCl_3$): δ 186.5, 164.0, 134.8,132.4, 130.0, 128.8, 71.1, 9.7 and 3.6; IR (KBr): 2964, 1732, 1688, 1597, 1451, 1200, 984 cm^{-1}; HRMS: calcd for $C_{12}H_{13}O_3$ (M+H): 205.0865, found: 208.0859. (Product **3h** contains a trace amount of cyclopropanemethanol (**2h**) and phenyl glyoxal (**13**) as impurity which is inseparable by coloumn chromatography)

(Tetrahydrofuran-2-yl)methyl 2-oxo-2-phenylacetate (3i)

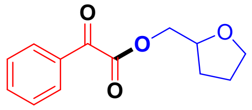

3i

Colourless liquid; **^{1}H NMR** (600 MHz, $CDCl_3$): δ 8.01 (d, *J* = 12.0 Hz, 2 H), 7.63 (t, *J*= 6.0 Hz, 1 H), 7.49 (t, *J*= 12.0 Hz, 2 H), 4.42-4.40 (m, 2 H), 4.39-4.33 (m, 1 H), 4.23 (t, *J*= 6.0 Hz, 1 H), 3.89-3.86 (m, 1 H), 3.81-3.78 (m, 1 H), 2.03-2.01 (m, 1 H), 1.93-1.90 (m, 2 H), 1.71-1.68 (m, 1

H); ^{13}C NMR (150 MHz, $CDCl_3$): δ 186.1, 163.8, 134.9,132.5, 130.1, 128.9, 76.0, 68.5, 67.5, 28.0 and 25.7; IR (KBr): 2927, 1738, 1688, 1450, 1200, 1176, 1069 cm^{-1}; HRMS: calcd for $C_{13}H_{14}O_4$ (M+H):235.0970, found:235.0965.

Isopropyl 2-oxo-2-phenylacetate (3j)

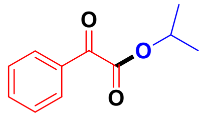

3j

Colourless liquid; ^{1}**H NMR** (600 MHz, $CDCl_3$): δ 7.96 (d, J = 12.0 Hz, 2 H), 7.60 (t, J= 6.0 Hz, 1 H), 7.47 (t, J= 6.0 Hz 2 H), 5.32-5.25 (m, 1 H), 1.37 (d, J = 6.0 Hz, 6 H); ^{13}**C NMR** (150 MHz, $CDCl_3$): δ 186.6, 163.5, 134.7, 132.4, 129.8, 128.7, 70.5 and 21.6; IR (KBr): 2984, 1732, 1689, 1597, 1451, 1323, 1170 cm^{-1}; HRMS: calcd for $C_{11}H_{13}O_3$ (M+H):193.0865, found:193.0859.

Tetrahydrofuran-3-yl 2-oxo-2-phenylacetate (3k)

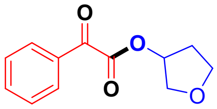

3k

Colourless liquid; ^{1}**H NMR** (600 MHz, $CDCl_3$): δ 7.97 (d, J = 6.0 Hz, 2 H), 7.63 (t, J = 6.0 Hz, 1 H), 7.50 (t, J = 6.0 Hz, 2 H), 5.58-5.55 (m, 1 H), 4.04-4.01(m, 1H), 3.98-3.87 (m, 3 H), 2.31-2.25 (m, 1 H), 2.17-2.14 (m, 1 H); ^{13}**C NMR** (150 MHz, $CDCl_3$): δ 185.8, 163.6, 135.0, 132.3, 129.9, 128.9, 77.0, 72.8, 66.7 and 32.7; IR (KBr): 2923, 1734, 1694, 1584, 1174 cm^{-1}; HRMS: calcd for $C_{12}H_{13S}O_4$ (M+H): 221.0814, found:221.0808.

Tetrahydro-2H-pyran-4-yl 2-oxo-2-phenylacetate (3l)

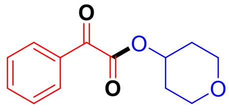

3l

Colourless liquid; ^{1}**H NMR** (600 MHz, $CDCl_3$): δ 7.96 (d, J = 12.0 Hz, 2 H), 7.63 (t, J= 6.0 Hz, 1 H), 7.48 (t, J= 6.0 Hz, 2 H), 5.26-5.22 (m, 1 H), 3.94-3.91 (m, 2 H), 3.58-3.54 (m, 2H), 2.05-

2.02 (m, 2H), 1.85-1.82 (m, 2H); **^{13}CNMR** (150 MHz, $CDCl_3$): δ 186.2, 163.3, 134.9, 132.2, 129.8, 128.9, 71.6, 65.0, 31.4; IR (KBr): 2957, 1743, 1664, 1593, 1443, 1200 cm^{-1}; ESI-MS: calcd for $C_{13}H_{14}NaO_4$ (M+Na):257.0790, found:257.0784.

Cyclopentyl 2-oxo-2-phenylacetate (3m)

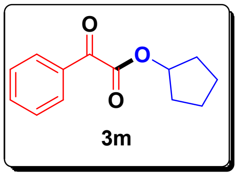

Colourless liquid; **^{1}H NMR** (600 MHz, $CDCl_3$): δ 7.96 (d, J = 6.0 Hz, 2 H), 7.61 (t, J= 6.0 Hz, 1 H), 7.47 (t, J= 6.0 Hz 2 H), 5.45-5.44 (m, 1 H), 1.97-1.94 (m, 2 H), 1.86-1.83 (m, 2 H), 1.76-1.73 (m, 2 H), 1.63-1.60 (m, 2 H); **^{13}C NMR** (150 MHz, $CDCl_3$): δ 186.6, 163.9, 134.7, 132.5, 129.9, 128.8, 79.5, 32.6 and 23.6; IR (KBr): 2965, 1731, 1689, 1597, 1450, 1204, 990 cm^{-1}; ESI-MS: calcd for $C_{13}H_{15}O_3$ (M+H):219.1021, found:219.1016.

Cyclohexyl 2-oxo-2-phenylacetate (3n)

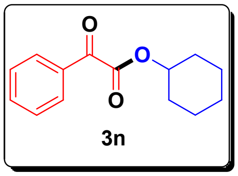

Colourless liquid; **^{1}H NMR** (600 MHz, $CDCl_3$): δ 7.63 (d, J = 6.0 Hz, 2 H), 7.61 (t, J= 6.0 Hz, 1 H), 7.46 (t, J= 6.0 Hz 2 H), 5.09-5.04 (m, 1 H), 1.98-1.73 (m, 2 H), 1.60-1.58 (m, 2 H), 1.56-1.53 (m, 2 H), 1.43-1.39 (m, 2 H), 1.38-1.36 (m, 2 H), 1.29-1.22 (m, 2 H); **^{13}C NMR** (150 MHz, $CDCl_3$): δ 186.7, 163.6, 134.7, 132.4, 129.8, 128.8, 75.3, 31.3, 25.0 and 23.5; IR (KBr): 2938, 1731, 1690, 1451, 1203, 1176, 989 cm^{-1}; HRMS: calcd for $C_{14}H_{17}O_3$ (M+H): 233.1178, found: 233.1172.

(1r,4r)-4-methylcyclohexyl 2-oxo-2-phenylacetate (3o)

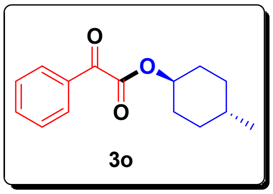

Colourless liquid; **^{1}H NMR** (600 MHz, $CDCl_3$): δ 7.97 (d, *J* = 12.0 Hz, 2 H), 7.62 (t, *J* = 6.0 Hz, 1 H), 7.48 (t, *J* = 6.0 Hz 2 H), 4.99-4.95 (m, 1 H), 2.10-2.09 (m, 2 H), 1.78-1.76(m, 2 H), 1.53-1.48(m, 2 H), 1.41-1.05(m, 3H) and 0.90 (d, *J* = 6.0 Hz, 3 H) ; **^{13}C NMR** (150 MHz, $CDCl_3$): δ 186.7, 163.6, 134.7, 132.5, 129.9, 128.8, 76.1, 32.8, 31.5, 31.4, and 21.7; IR (KBr): 2949, 1731, 1691, 1203, 1177 cm^{-1}; ESI-MS: calcd for $C_{15}H_{18}O_3$ (M+H): 247.1334, found: 247.1356. (Product **3o** contains trace amount of trans-4-methylcyclohexanol (**2o**) and phenylglyoxal (**13**) as impurity which is inseparable by coloumn chromatography)

Tert-butyl 2-oxo-2-phenylacetate (3p)

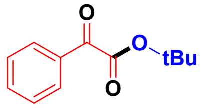

3p

Colourless liquid; **^{1}H NMR** (600 MHz, $CDCl_3$): δ 7.96 (d, *J* = 12.0 Hz, 2 H), 7.62 (t, *J*= 6.0 Hz, 1 H), 7.48 (t, *J*= 6.0 Hz 2 H), 1.61(m, 9 H); **^{13}C NMR** (150 MHz, $CDCl_3$): δ 186.8, 163.7, 134.6, 132.5, 129.8, 128.8, 84.7, and 28.0; IR (KBr): 2923, 2850, 1730,1691, 1597, 1214 cm^{-1}; HRMS: calcd for $C_{12}H_{15}O_3$ (M+H): 207.1021, found: 207.1016.

Ethyl 2-oxo-2-p-tolylacetate (4b)

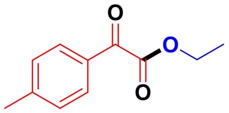

4b

Colourless liquid; **^{1}H NMR** (600 MHz, $CDCl_3$): δ 7.88 (d, *J* = 12.0 Hz, 2 H), 7.28 (d, *J* = 12.0 Hz, 2 H), 4.42 (q, *J* = 6.0 Hz, 2 H), 2.40 (s, 3H) 1.38 (t, *J* = 6.0 Hz, 3 H); **^{13}C NMR** (150 MHz, $CDCl_3$): δ 186.0, 163.9, 146.1, 130.0, 129.9, 129.5, 62.1, 21.8 and 14.0; IR (KBr): 2924, 1736, 1691, 1597, 1451, 1323, 1176, 1003 cm^{-1}; HRMS: calcd for $C_{11}H_{13}O_3$ (M+H): 193.0865, found: 193.0859.

Cyclohexyl 2-oxo-2-m-tolylacetate (4c)

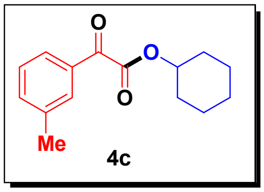

Colourless liquid; **^{1}H NMR** (600 MHz, $CDCl_3$): δ 7.76 (t, J = 6.0 Hz, 2 H), 7.43 (d, J = 6.0 Hz, 1 H),7.36 (t, J = 6.0 Hz, 1 H), 5.09-5.04 (m, 1 H), 2.38 (s, 3H), 1.98-1.95 (m, 2 H), 1.77-1.74 (m, 2 H), 1.58-1.53 (m, 2 H), 1.43-1.40 (m, 2 H), 1.39-1.36 (m, 2 H), 1.30-1.26 (m, 2 H); **^{13}C NMR** (150 MHz, $CDCl_3$): δ 186.0, 163.7, 138.7, 135.5, 132.5, 130.1, 128.6, 127.2, 75.2, 31.3, 25.1, 23.5 and 21.2; IR (KBr): 2936, 1732, 1688, 1232, 1158, 1035 cm^{-1}; HRMS: calcd for $C_{15}H_{19}O_3$ (M+H):247.1134, found:247.1145.

Benzyl 2-(4-butylphenyl)-2-oxoacetate (4d)

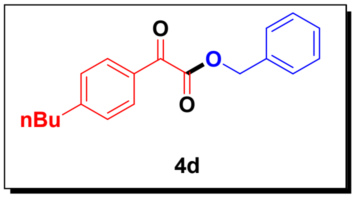

Colourless liquid; **^{1}H NMR** (600 MHz, $CDCl_3$): δ 7.88 (d, J = 6.0 Hz, 2 H), 7.44 (d, J= 6.0 Hz, 2 H), 7.39-7.34 (m, 3 H), 7.28 (d, J = 12.0 Hz, 2 H), 5.39 (s, 2 H), 2.67 (t, J = 6.0 Hz, 2 H), 1.64-1.61 (m, 2 H), 1.60-1.57 (m, 2 H), 1.36 (t, J = 6.0 Hz, 3 H); **^{13}C NMR** (150 MHz, $CDCl_3$): δ 185.6, 171.4, 163.8, 151.1, 134.5, 130.1, 128.9, 128.7, 128.6, 128.5, 67.5, 35.8, 32.9, 22.2 and 13.7; IR (KBr): 2929, 1735, 1686, 1605, 1288, 1170, 995 cm^{-1}; HRMS: calcd for $C_{19}H_{21}O_3$ (M+H): 247.1491, found: 247.1484.

Methyl 2-(4-tert-butylphenyl)-2-oxoacetate (4e)

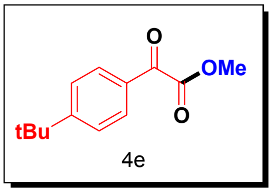

Colourless liquid; **^{1}H NMR** (600 MHz, $CDCl_3$): δ 7.93 (d, J = 6.0 Hz, 2 H), 7.50 (d, J= 6.0 Hz, 2 H), 5.39 (s, 2 H), 3.94 (s, 3 H), 1.61 (s, 9 H); **^{13}C NMR** (150 MHz, $CDCl_3$): δ 185.6, 164.1,

159.1, 130.0, 129.8, 125.8, 52.6, 35.3, and 30.9; IR (KBr): 2964, 1742, 1683, 1603, 1411, 1216, 1176, 1109 cm^{-1}; ESI-MS: calcd for $C_{13}H_{16}NaO_3$ (M+Na):243.0977, found:243.0992.

Tert-butyl 2-(4-tert-butylphenyl)-2-oxoacetate (4f)

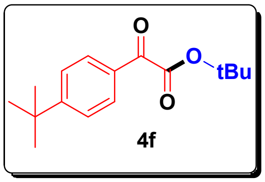

Colourless liquid; **^{1}H NMR** (600 MHz, $CDCl_3$): δ 7.89 (d, J = 6.0 Hz, 2 H), 7.50 (d, J= 6.0 Hz, 2 H), 1.61(m, 9 H), 1.32 (m, 9 H); **^{13}C NMR** (150 MHz, $CDCl_3$): δ 186.5, 163.9, 158.7, 130.5, 129.9, 125.8, 84.5, 35.3, 30.9 and 28.1; IR (KBr): 2964, 1729, 1684, 1604, 1460, 1369, 1220, 1153, 1108, 987 cm^{-1}; HRMS: calcd for $C_{16}H_{23}O_3$ (M+H):263.1647, found:263.1642.

Methyl 2-(2-chlorophenyl)-2-oxoacetate (4g)

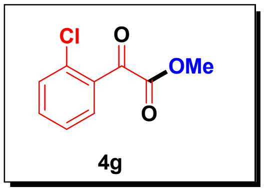

Colourless liquid; **^{1}H NMR** (600 MHz, $CDCl_3$): δ 7.74 (d, J = 6.0 Hz, 1 H), 7.52 (t, J= 6.0 Hz, 1 H), 7.43-7.37 (m, 2 H), 3.93 (s, 3 H); **^{13}C NMR** (150 MHz, $CDCl_3$): δ 186.1, 163.4, 134.3, 133.2, 131.5, 130.5, 127.2 and 53.2; IR (KBr): 2924, 1734, 1686, 1594, 1203 cm^{-1}; HRMS: calcd for $C_9H_7ClO_3$ (M+H): 199.0162, found: 199.0156.

Cyclohexyl 2-(4-chlorophenyl)-2-oxoacetate (4h)

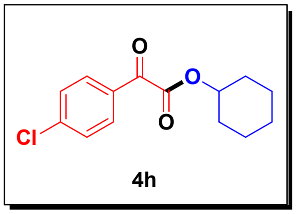

Colourless liquid; **^{1}H NMR** (600 MHz, $CDCl_3$): δ 7.94 (d, J = 12.0 Hz, 2 H), 7.47 (d, J = 12.0 Hz, 2 H), 5.07-5.04 (m, 1 H), 1.98-1.95 (m, 2 H), 1.79-1.74 (m, 2 H), 1.60-1.54 (m, 2 H), 1.44-1.37 (m, 2 H), 1.31-1.25 (m, 2 H); **^{13}C NMR** (150 MHz, $CDCl_3$): δ 185.3, 163.0, 141.4, 131.3,

131.0, 129.2, 75.7, 31.4, 25.1, 23.6; IR (KBr): 2921, 2850, 1727, 1688, 1645, 1588, 1469, 1201, 991 cm^{-1}; HRMS: calcd for $C_{14}H_{16}ClO_3$ (M+H): 267.0788, found: 267.0782.

Cyclohexyl 2-(4-fluorophenyl)-2-oxoacetate (4i)

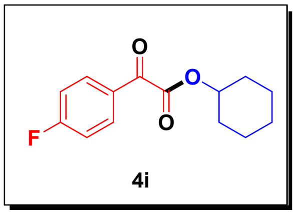

Colourless liquid; **^{1}H NMR** (600 MHz, $CDCl_3$): δ 8.02 (d, J = 6.0 Hz, 2 H), 7.14 (t, J = 6.0 Hz, 2 H), 5.07-5.02 (m, 1 H), 1.97-1.95 (m, 2 H), 1.76-1.73 (m, 2 H), 1.57-1.53 (m, 2 H), 1.43-1.36 (m, 2 H), 1.31-1.24 (m, 2 H); **^{13}C NMR** (150 MHz, $CDCl_3$): δ 184.9, 167.5, 165.8, 163.1, 132.8, 132.7, 129.0, 116.2, 116.0, 75.5, 31.3, 25.0, 23.5; IR (KBr): 2939, 2861, 1727, 1688, 1599, 1200, 1155 cm^{-1}; HRMS: calcd for $C_{14}H_{16}FO_3$ (M+H):251.1083 found:251.1078.

Propyl 2-(3-iodo-2-methoxyphenyl)-2-oxoacetate (4j)

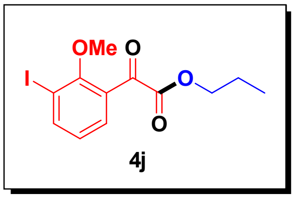

Colourless liquid; **^{1}H NMR** (600 MHz, $CDCl_3$): δ 8.03 (d, J = 6.0 Hz, 1 H), 7.80 (d, J= 6.0 Hz, 1 H), 6.99 (t, J= 6.0 Hz, 1 H), 4.27 (t, J= 6.0 Hz, 2 H), 3.80 (s, 3 H),1.76-1.73 (m, 2 H), 0.96 (t, J = 6.0 Hz, 3 H); **^{13}C NMR** (150 MHz, $CDCl_3$): δ 186.6, 164.4, 160.9, 146.0, 130.9,128.9, 126.4, 91.6, 67.8, 63.5, 21.7 and 10.2; IR (KBr): 2968, 1736, 1685, 1581, 1118 cm^{-1}; ESI-MS: calcd for $C_{12}H_{13}INaO_4$ (M+Na): 370.9756, found: 370.9751.

Cyclohexyl 2-oxo-2-(2-(trifluoromethyl)phenyl)acetate (4k)

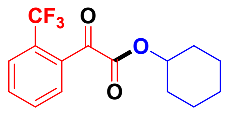

4k

Colourless liquid; **¹H NMR** (600 MHz, $CDCl_3$): δ 7.72 (d, *J* = 6.0 Hz, 1 H), 7.63 (t, *J* = 6.0 Hz, 2 H), 7.55 (d, *J* = 6.0 Hz, 1 H), 4.97-4.93 (m, 1 H), 1.91-1.89 (m, 2 H), 1.73-1.69 (m, 2 H), 1.55-1.49 (m, 2 H), 1.39-1.32 (m, 2 H), 1.27-1.21 (m, 2 H); **¹³C NMR** (150 MHz, $CDCl_3$): δ 187.1, 160.6, 134.5, 131.8, 129.5, 128.5, 128.3, 126.8, 126.7, 124.3, 122.4, 76.1, 31.0, 25.0, 23.5; IR (KBr): 2942, 2863, 1727, 1582, 1316, 1597, 1204 cm^{-1}; HRMS: calcd for $C_{15}H_{16}F_3O_3$ (M+H): 301.1052, found: 301.1046.

Methyl 2-(4-cyanophenyl)-2-oxoacetate (4l)

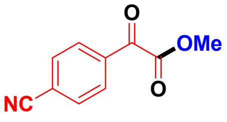

4l

Colourless liquid; **¹H NMR** (600 MHz, $CDCl_3$): δ 8.14 (d, *J* = 12.0 Hz, 2 H), 7.79 (d, *J* = 6.0 Hz, 2 H), 3.97 (s, 3 H); **¹³C NMR** (150 MHz, $CDCl_3$): δ 183.9, 162.5, 135.4, 132.5, 130.4, 117.9, 117.4, 53.1; IR (KBr): 2965, 2235, 1738, 1688, 1606, 1408, 1325, 1205, 1005 cm^{-1}; ESI-MS: calcd for $C_{10}H_8NO_3$ (M+H): 190.0504, found:190.0577.

Isopropyl 2-(4-cyano-3-methylphenyl)-2-oxoacetate (4m)

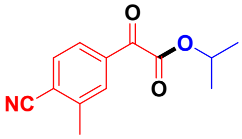

4m

Colourless liquid; **¹H NMR** (600 MHz, $CDCl_3$): δ 7.93 (s, 1 H), 7.86 (d, *J* = 6.0 Hz, 1 H), 7.72 (d, *J*= 12.0 Hz, 1 H), 5.31-5.28 (m, 1 H), 2.60 (s, 3 H),1.39 (d, *J* = 6.0 Hz, 6 H); **¹³C NMR** (150 MHz, $CDCl_3$): δ 185.1, 162.3, 142.7, 135.4, 132.8, 131.1, 127.4, 118.2, 116.8, 71.2, 21.6 and

20.5; IR (KBr): 2922, 2227, 1739, 1695, 1289 cm^{-1}; HRMS: calcd for $C_{13}H_{14}NO_3$ (M+H):232.0974, found:232.0968.

Methyl 2-(3-nitrophenyl)-2-oxoacetate (4n)

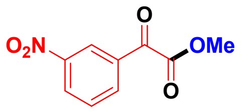

4n

Pale Yellow solid; ^{1}**H NMR** (600 MHz, $CDCl_3$): δ 8.48 (s, 1 H), 8.47 (d, J = 6.0 Hz, 1 H), 8.38 (d, J= 6.0 Hz, 1 H), 7.72 (t, J = 12.0 Hz, 1 H), 3.99 (s, 3 H); ^{13}**C NMR** (150 MHz, $CDCl_3$): δ 183.0, 162.3, 148.4, 135.4, 133.8, 130.1, 128.8, 124.9 and 53.2; IR (KBr): 2932, 1737, 1690, 1597, 1343, 1206 cm^{-1}; ESI-MS: calcd for $C_9H_7NaNO_5$ (M+Na): 232.0222, found: 232.0256.

Tert-butyl 2-(3-nitrophenyl)-2-oxoacetate (4o)

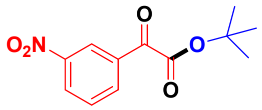

4o

Pale Yellow solid; ^{1}**H NMR** (600 MHz, $CDCl_3$): δ 8.83 (s, 1 H), 8.47 (d, J = 6.0 Hz, 1 H), 8.32 (d, J= 6.0 Hz, 1 H), 7.71 (t, J = 6.0 Hz, 1 H), 1.63 (s, 9 H); ^{13}**C NMR** (150 MHz, $CDCl_3$): δ 184.1, 161.9, 148.4, 135.4, 135.2, 134.0, 128.6, 124.9, 110.7, 85.8, and 28.0; IR (KBr): 2982, 1727, 1699, 1614, 1535, 1350, 1212, 1153 cm^{-1}; ESI-MS: calcd for $C_{12}H_{13}NaNO_5$ (M+Na): 274.0691, found:274.0686.

Isopropyl 2-(4-acetylphenyl)-2-oxoacetate (4p)

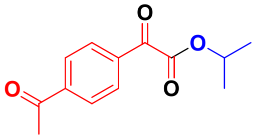

4p

Colourless liquid; **^{1}H NMR** (600 MHz, $CDCl_3$): δ 8.08 (d, J = 12.0 Hz, 2 H), 8.02 (d, J= 12.0 Hz, 2 H), 5.34-5.27 (m, 1 H), 2.63 (s, 3 H), 1.40 (d, J = 6.0 Hz, 6 H); **^{13}C NMR** (150 MHz, $CDCl_3$): δ 197.2, 185.7, 162.8, 141.2, 135.6, 130.1,128.5, 71.0, 26.9 and 21.6; IR (KBr): 2927, 2856, 1732,1683, 1259 cm^{-1}; ESI-MS: calcd for $C_{13}H_{14}NaO_4$ (M+Na):257.0790, found:257.0784.

Methyl 2-(2-methoxy-2-oxoacetyl)benzoate (4q)

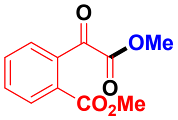

4q

Colourless liquid; **^{1}H NMR** (600 MHz, $CDCl_3$): δ 7.99 (d, J = 12.0 Hz, 1 H), 7.64 (t, J= 12.0 Hz, 1 H), 7.59 (t, J= 6.0 Hz, 1 H), 7.51 (d, J = 6.0 Hz, 1 H), 3.85 (s, 3 H), 3.84 (s, 3 H); **^{13}C NMR** (150 MHz, $CDCl_3$): δ 187.2, 166.6, 161.2, 138.5, 133.0,131.4, 129.6, 129.5, 128.9, 52.9, 52.6; IR (KBr): 2924, 1756, 1602, 1467, 1209, 1089, 1013 cm^{-1}; HRMS: calcd for $C_{11}H_{11}O_5$ (M+H):223.0606, found:223.0601.

Methyl 2-(4-(methylsulfonyl)phenyl)-2-oxoacetate (4r)

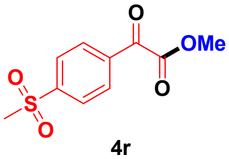

4r

Colourless liquid; **^{1}H NMR** (600 MHz, $CDCl_3$): δ 8.21 (d, J = 6.0 Hz, 2 H), 8.05 (d, J= 6.0 Hz, 2 H), 3.97 (s, 3 H), 3.06 (s, 3 H); **^{13}C NMR** (150 MHz, $CDCl_3$): δ 184.1, 162.6, 145.5, 136.3, 130.9, 127.8, 53.2, 44.1; IR (KBr): 2926, 2853, 1743, 1691, 1404, 11551 cm^{-1}; ESI-MS: calcd for $C_{10}H_{10}O_5S$ (M+Na): 265.0147, found: 265.0156.

Cyclopropylmethyl 2-(3-methoxyphenyl)-2-oxoacetate (4s)

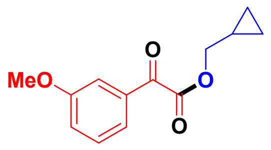

4s

Colourless liquid; **^{1}H NMR** (600 MHz, $CDCl_3$): δ 7.55 (d, J = 6.0 Hz, 1 H), 7.50 (s, 1 H), 7.38 (t, J= 6.0 Hz, 1 H), 7.16 (d, J = 12.0 Hz, 1 H), 4.20 (d, J = 6.0 Hz, 2 H), 3.83 (s, 3 H), 1.27-1.23 (m, 1 H), 0.64-0.62 (m, 2 H), 0.38-0.36 (m, 2 H); **^{13}C NMR** (150 MHz, $CDCl_3$): δ 186.3, 164.0, 159.8, 133.6, 129.8, 123.0, 121.8, 113.1, 71.1, 55.4, 9.72, 3.60; IR (KBr): 2956, 1735, 1686, 1597, 1250 cm^{-1}; ESI-MS: calcd for $C_{13}H_{14}O_4$ (M+Na): 257.0790, found:257.0766.

Isopropyl 2-(3-methoxyphenyl)-2-oxoacetate (4t)

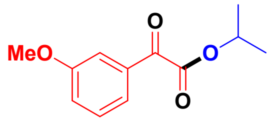

4t

Colourless liquid; **^{1}H NMR** (600 MHz, $CDCl_3$): δ 7.51 (d, J = 6.0 Hz, 1 H), 7.47 (d, J = 6.0 Hz, 1 H), 7.37 (t, J= 12.0 Hz, 1 H), 7.15 (d, J= 12.0 Hz, 1 H), 5.31-5.25 (m, 1 H), 3.81 (s, 3 H),1.37 (d, J = 6.0 Hz, 6 H); **^{13}C NMR** (150 MHz, $CDCl_3$): δ 186.5, 163.5, 159.8, 133.6, 129.8, 122.9, 121.6, 113.1, 70.6, 55.4 and 21.6; IR (KBr): 2984, 1732, 1689, 1598, 1487, 1254 cm^{-1}; HRMS: calcd for $C_{12}H_{15}O_4$ (M+H):223.0970, found:223.0965.

Dimethyl 2,2'-(1,3-phenylene)bis(2-oxoacetate) (4u)

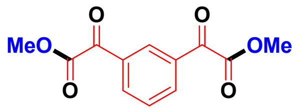

4u

Colourless liquid; **^{1}H NMR** (600 MHz, $CDCl_3$): δ 8.68 (s, 1 H), 8.30 (d, J = 6.0 Hz, 2 H), 7.66 (t, J = 6.0 Hz, 1 H), 3.98 (s, 6 H); **^{13}C NMR** (150 MHz, $CDCl_3$): δ 184.2, 162.9, 135.6, 133.1, 131.7, 129.6, 53.1; IR (KBr): 2924, 1728, 1693, 1603, 1441, 1305, 1204 cm^{-1}; ESI-MS: calcd for $C_{12}H_{11}O_6$ (M+H): 251.0556, found: 251.0550.

Dimethyl 2,2'-(2-methoxy-1,3-phenylene)bis(2-oxoacetate) (4v)

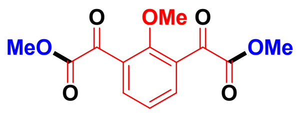

4v

Colourless liquid; ^{1}**H NMR** (600 MHz, $CDCl_3$): δ 8.03 (d, *J* = 6.0 Hz, 2 H), 7.37 (t, *J* = 6.0 Hz, 1 H), 3.90 (s, 6 H), 3.75 (s, 3 H); ^{13}**C NMR** (150 MHz, $CDCl_3$): δ 185.3, 164.0, 162.1, 136.8, 128.3, 124.7, 66.0, 52.9; IR (KBr): 2923, 2845, 1749, 1686, 1592, 1221 cm^{-1}; HRMS: calcd for $C_{13}H_{13}O_7$ (M+H): 281.0661, found: 281.0656.

Tert-butyl 2-(3-ethynylphenyl)-2-oxoacetate (4w)

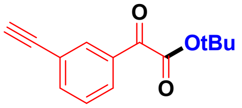

4w

Colourless liquid; ^{1}**H NMR** (600 MHz, $CDCl_3$): δ 8.06 (d, *J* = 6.0 Hz, 1 H), 7.93 (d, *J* = 6.0 Hz, 1 H), 7.72 (d, *J* = 6.0 Hz, 1 H), 7.47 (t, *J* = 6.0 Hz, 1 H) 3.13 (s, 1 H), 1.61 (s, 9 H); ^{13}**C NMR** (150 MHz, $CDCl_3$): δ 185.8, 163.0, 137.7, 133.4, 132.7, 129.9, 128.9, 123.1, 85.1, 82.0, 78.7, 28.0; IR (KBr): 3271, 2923, 1726, 1678, 1221 cm^{-1}; ESI-MS: calcd for $C_{14}H_{14}O_3$ (M+Na): 231.1021, found: 231.1016.

Methyl 2-oxo-2-(thiophen-2-yl)acetate (4x)

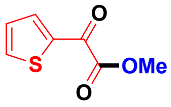

4x

Colourless liquid; ^{1}**H NMR** (600 MHz, $CDCl_3$): δ 8.13 (d, *J* = 6.0 Hz, 1 H), 7.80 (d, *J*= 6.0 Hz, 1 H), 7.18 (t, *J* = 6.0 Hz, 1 H), 3.94 (s, 3 H); ^{13}**C NMR** (150 MHz, $CDCl_3$): δ 175.9, 162.0, 139.0, 137.6, 137.3, 128.6, 53.1; IR (KBr): 2926, 1732, 1684, 1597, 1221 cm^{-1}; HRMS: calcd for $C_7H_7O_3S$ (M+H): 171.0116, found: 171.0117.

Isopropyl 2-oxo-2-(thiophen-3-yl)acetate (4y)

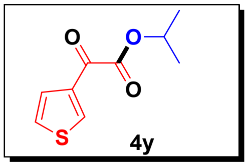

Colourless liquid; **^{1}H NMR** (600 MHz, $CDCl_3$): δ 8.48 (d, J = 6.0 Hz, 1 H), 7.66 (d, J= 6.0 Hz, 1 H), 7.34 (d, J = 6.0 Hz, 1 H), 5.26-5.22 (m, 1 H), 1.39 (d, J = 6.0 Hz, 6 H; **^{13}C NMR** (150 MHz, $CDCl_3$): δ 178.6, 162.1, 137.4, 127.8, 126.6, 70.7, 21.6; IR (KBr): 2923, 2851, 1726, 1673, 1220 cm^{-1}; HRMS: calcd for $C_9H_{11}O_3S$ (M+H): 199.0429, found: 199.0423.

Tert-butyl 2-oxo-2-(thiophen-3-yl)acetate (4z)

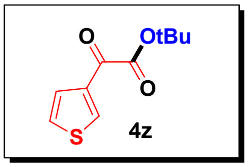

Colourless liquid; **^{1}H NMR** (600 MHz, $CDCl_3$): δ 8.41 (d, J = 6.0 Hz, 1 H), 7.63 (d, J= 6.0 Hz, 1 H), 7.33 (d, J = 6.0 Hz, 1 H), 1.59 (s, 9 H); **^{13}C NMR** (150 MHz, $CDCl_3$): δ 179.3, 162.2, 136.9, 127.7, 126.5, 84.4, 27.9; IR (KBr): 2949, 1707, 1606, 1285, 1187, 1115 cm^{-1}; HRMS: calcd for $C_{10}H_{13}O_3S$ (M+H): 213.0585, found: 213.0568.

Methyl 2-oxo-2-(pyridin-3-yl)acetate (4va)

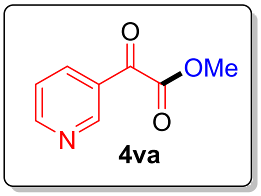

Pale yellow solid; **^{1}H NMR** (600 MHz, $CDCl_3$): δ 9.23 (s, 1 H), 8.83 (d, J= 6.0 Hz, 1 H), 8.34 (m, 1 H), 7.46 (d, J= 6.0 Hz, 1 H), 3.97 (s, 3 H) ; **^{13}C NMR** (150 MHz, $CDCl_3$): δ 184.1, 162.4, 154.8, 151.5, 137.2, 128.4, 123.7, 53.1; IR (neat): 2961, 1736, 1687, 1596,1451, 1195 cm^{-1}; HRMS: calcd for $C_8H_9NO_3$ (M+H): 166.0498, found: 166.0499.

Methyl 2-(benzo[d][1,3]dioxol-5-yl)-2-oxoacetate (4wa)

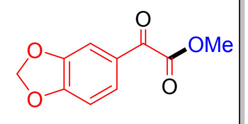

4wa

Pale yellow liquid; **^{1}H NMR** (600 MHz, $CDCl_3$): δ 7.61 (d, *J*= 6.0 Hz, 1 H), 7.46 (s, *J*= 6.0 Hz, 1 H), 6.87 (d, *J*= 6.0 Hz, 1 H), 6.06 (s, 2 H), 3.93 (s, 3 H); **^{13}C NMR** (150 MHz, $CDCl_3$): δ 184.1, 164.1, 153.6, 148.5, 127.9, 127.1, 108.7, 108.3, 102.2, 52.7, 29.6; IR (neat): 2924, 1737, 1677, 1450, 1244, 1203, 1105 cm^{-1}; HRMS: calcd for $C_{10}H_8O_5$ (M+H): 231.0269, found: 231.0264.

Dimethyl 2,2'-(1,3-phenylene)bis(2-(hydroxyimino)acetate) (5t)

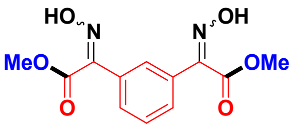

5t

white solid; **^{1}H NMR** (600 MHz, $CDCl_3$): δ 8.86 (bs, 1 H), 7.78 (s, 1 H), 7.56 (t, *J* = 6.0 Hz, 1 H), 7.40 (t, *J* = 6.0 Hz, 1 H), 3.94 (s, 6 H); **^{13}C NMR** (150 MHz, $CDCl_3$): δ 163.6, 150.6, 131.0, 129.2, 128.3, 124.5, 52.6; IR (neat): 2971, 2833, 1748, 1691, 1158 cm^{-1}; ESI-MS: calcd for $C_{12}H_{12}N_2O_6$ (M+Na): 303.0593, found:303.0584.

1-benzyl-3-(3-nitrophenyl)quinoxalin-2(1H)-one (6n)

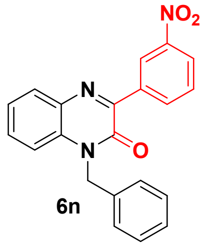

6n

Yellow solid; **^{1}H NMR** (600 MHz, $CDCl_3$): δ 9.32 (s, 1 H), 8.82 (m, 1H), 8.29 (m, 1 H), 7.95 (m, 1H), 7.62 (t, *J* = 6.0 Hz, 1 H),7.48 (t, *J* = 6.0 Hz, 1 H), 7.35-7.23 (m, 6H), 5.63 (s, 2H); **^{13}C NMR** (150 MHz, $CDCl_3$): δ 154.4, 150.9, 148.1, 137.3, 135.4, 134.9, 132.9, 132.8, 131.2, 130.8, 128.9, 127.7, 126.8, 124.7, 124.6, 124.0, 114.5, 46.1; IR (neat): 2971, 1726, 1678, 1513, 1343, 1158 cm^{-1}; ESI-MS: calcd for $C_{21}H_{16}N_3O_3$ (M+H):358.1192, found: 358.1186.

6,7-Dimethyl-2-phenylquinoxaline (8)

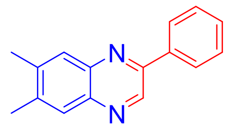

8

Yellow solid; ^{1}**H NMR** (600 MHz, $CDCl_3$): δ 9.20 (s, 1 H), 8.15 (d, J = 6.0 Hz, 2H), 8.89 (s, 1 H), 7.84 (s, 1H), 7.54-7.52 (m, 2 H),7.49-7.47 (m, 1 H), 2.49 (s, 6 H); ^{13}**C NMR** (150 MHz, $CDCl_3$): δ 151.0, 142.4, 141.2, 140.8, 140.5, 137.1, 129.8, 129.0, 128.6, 128.1, 127.3, 20.4, 20.3; IR (neat): 3454, 3062, 2926, 1612, 1572, 1129 cm^{-1}; EI-MS: calcd for $C_{16}H_{14}N_2$: 234.1157, found: 234.1155.

Phenyl glyoxal monohydrate (13)[S8]

(Phenylglyoxal exists in hydrated form at room temperature.)[S8]

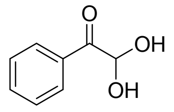

13

White solid; ^{1}**H NMR** (600 MHz, DMSO): δ 8.06 (d, J = 6.0 Hz, 2 H), 7.63 (t, J = 6.0 Hz, 1H), 7.50 (t, J = 6.0 Hz, 2 H), 6.73 (d, J = 6.0 Hz, 2H), 5.67 (t, J = 6.0 Hz, 1 H); ^{13}**C NMR** (150 MHz, $CDCl_3$): δ 196.1, 133.6, 133.2, 129.3, 128.4, 89.1. IR (neat): 3322, 1694, 1595, 1443, 1225, 1112 cm^{-1} (data are consistent with those reported in literature ref. S8).

3,4-diphenylnaphthalene-1,2-dione (16) [S9]

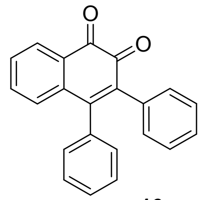

16

Red solid; ^{1}**H NMR** (600 MHz, $CDCl_3$): δ 8.20 (d, J = 6.0 Hz, 1 H), 7.52-7.46 (m, 2 H), 7.29-7.25 (m, 3 H), 7.13-7.08 (m, 5 H), 7.04 (d, J = 12.0 Hz, 1 H) 6.95-6.94 (m, 2 H); ^{13}**C NMR** (150 MHz, $CDCl_3$): δ 180.6, 179.1, 152.6, 138.5, 136.8, 135.6, 135.4, 133.4, 131.1, 130.3, 130.2, 130.1, 129.1, 128.2, 128.1, 127.5, 127.4 ; IR (KBr, cm^{-1}): 1658, 1581, 1342, 1272. HRMS: calcd

for $C_{22}H_{14}O_2$: 310.0994, found 310.0995. (data are consistent with those reported in literature ref. S9)

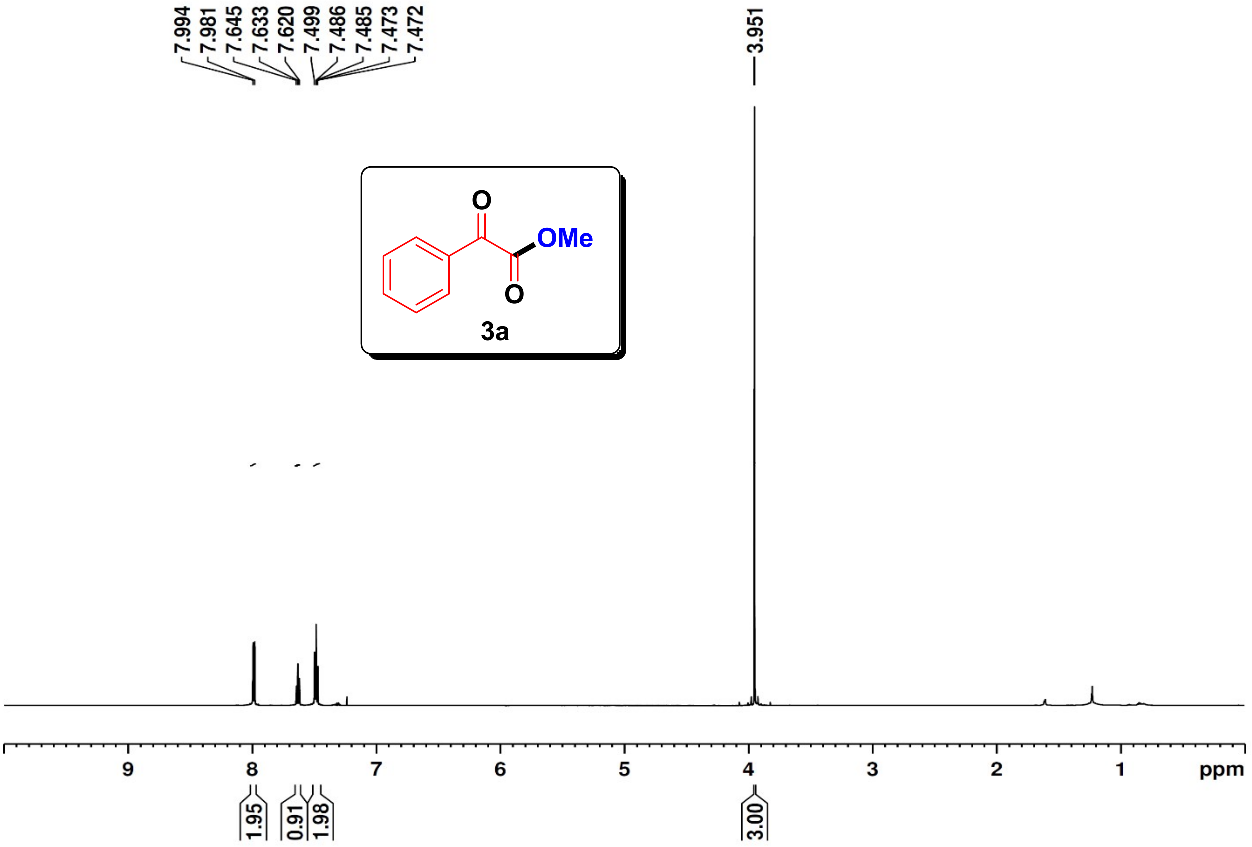
7.994
7.981
7.645
7.633
7.620
7.499
7.486
7.485
7.473
7.472
3.951
O
OMe
O
3a
9
8
7
6
5
4
3
2
1
ppm
1.95
0.91
1.98
3.00

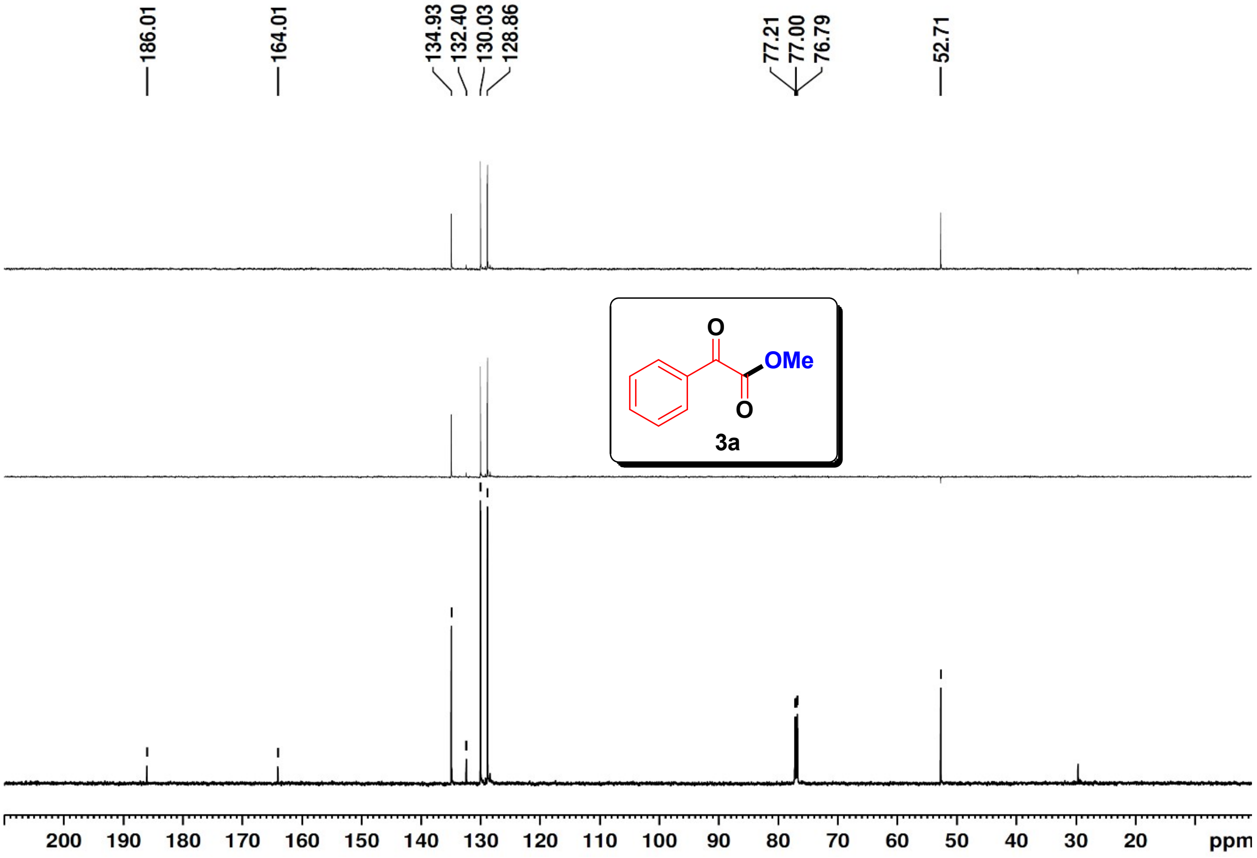
186.01
164.01
134.93
132.40
130.03
128.86
77.21
77.00
76.79
52.71
O
OMe
O
3a
200
190
180
170
160
150
140
130
120
110
100
90
80
70
60
50
40
30
20
ppm

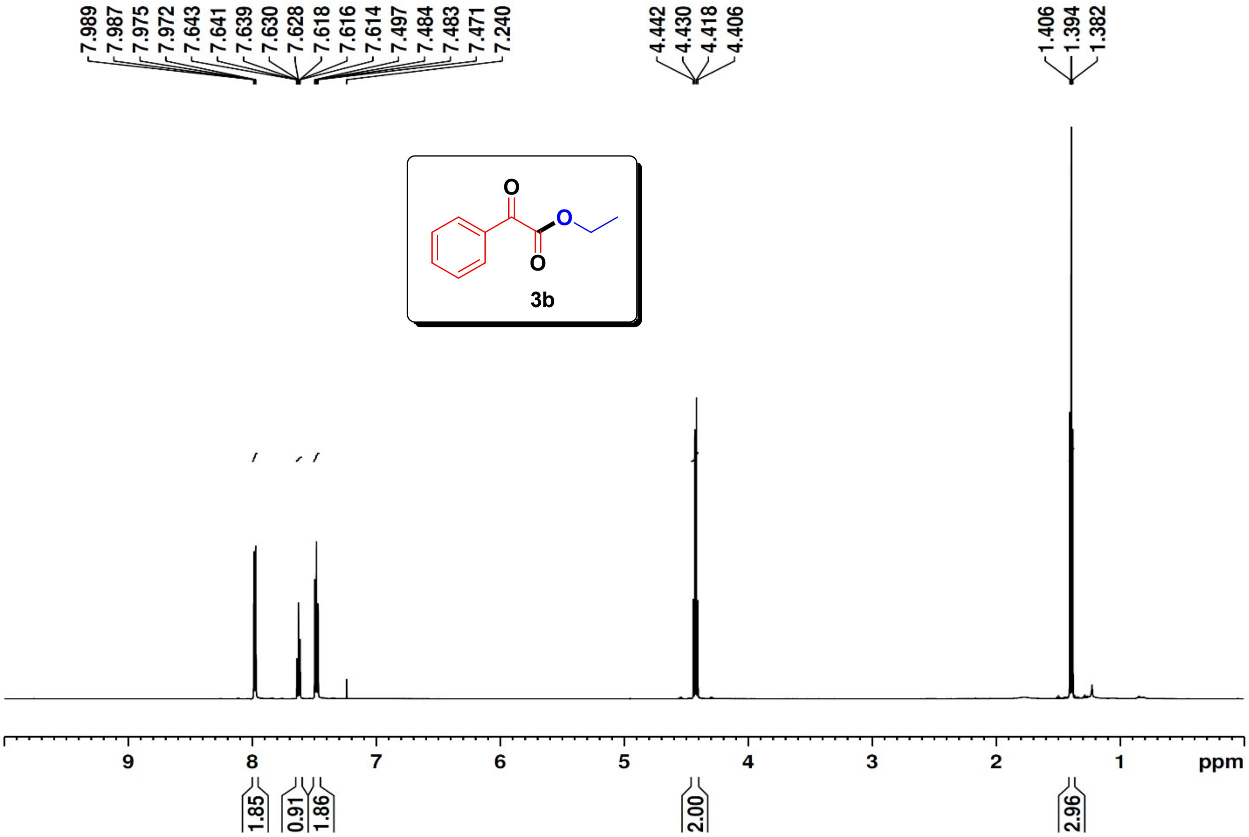
7.989 7.987 7.975 7.972 7.643 7.641 7.639 7.630 7.628 7.618 7.616 7.614 7.497 7.484 7.483 7.471 7.240
4.442 4.430 4.418 4.406
1.406 1.394 1.382
3b
1.85 0.91 1.86 2.00 2.96
ppm

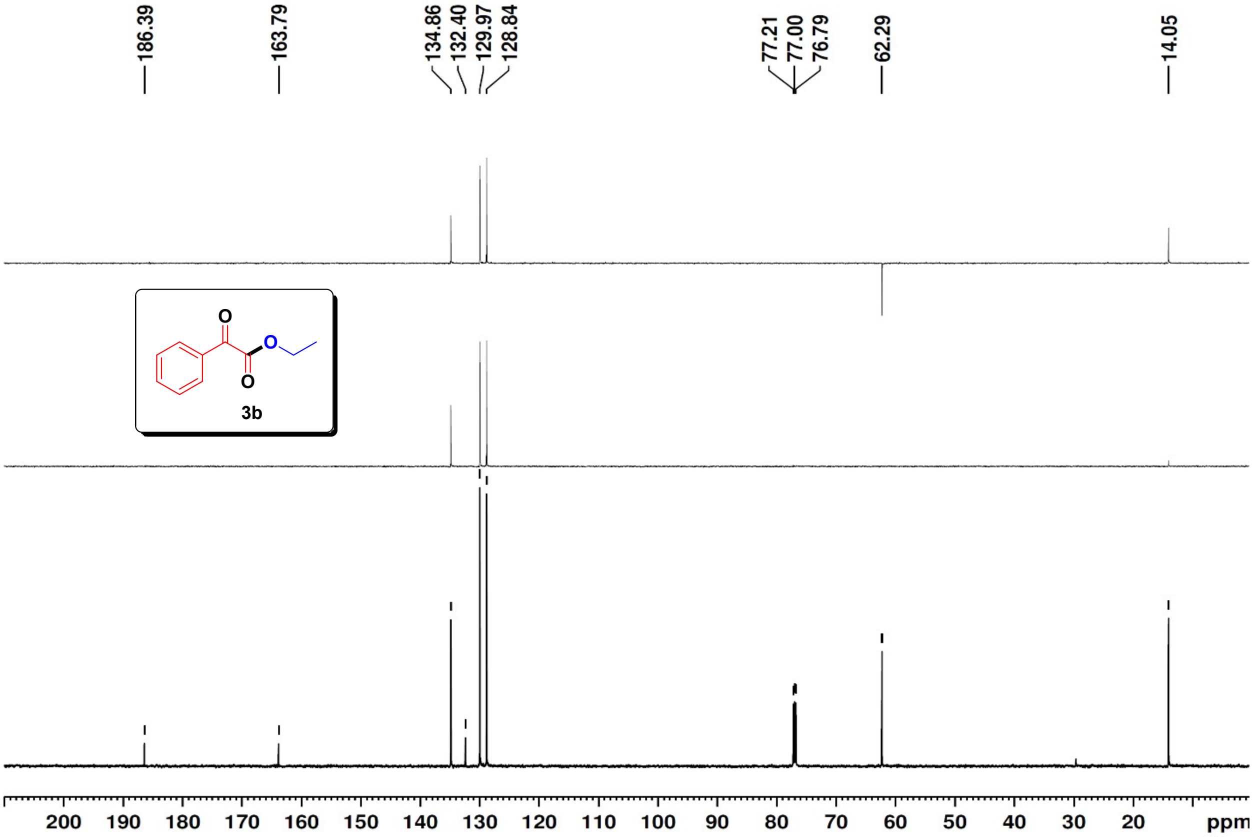
186.39
163.79
134.86 132.40 129.97 128.84
77.21 77.00 76.79
62.29
14.05
3b
ppm

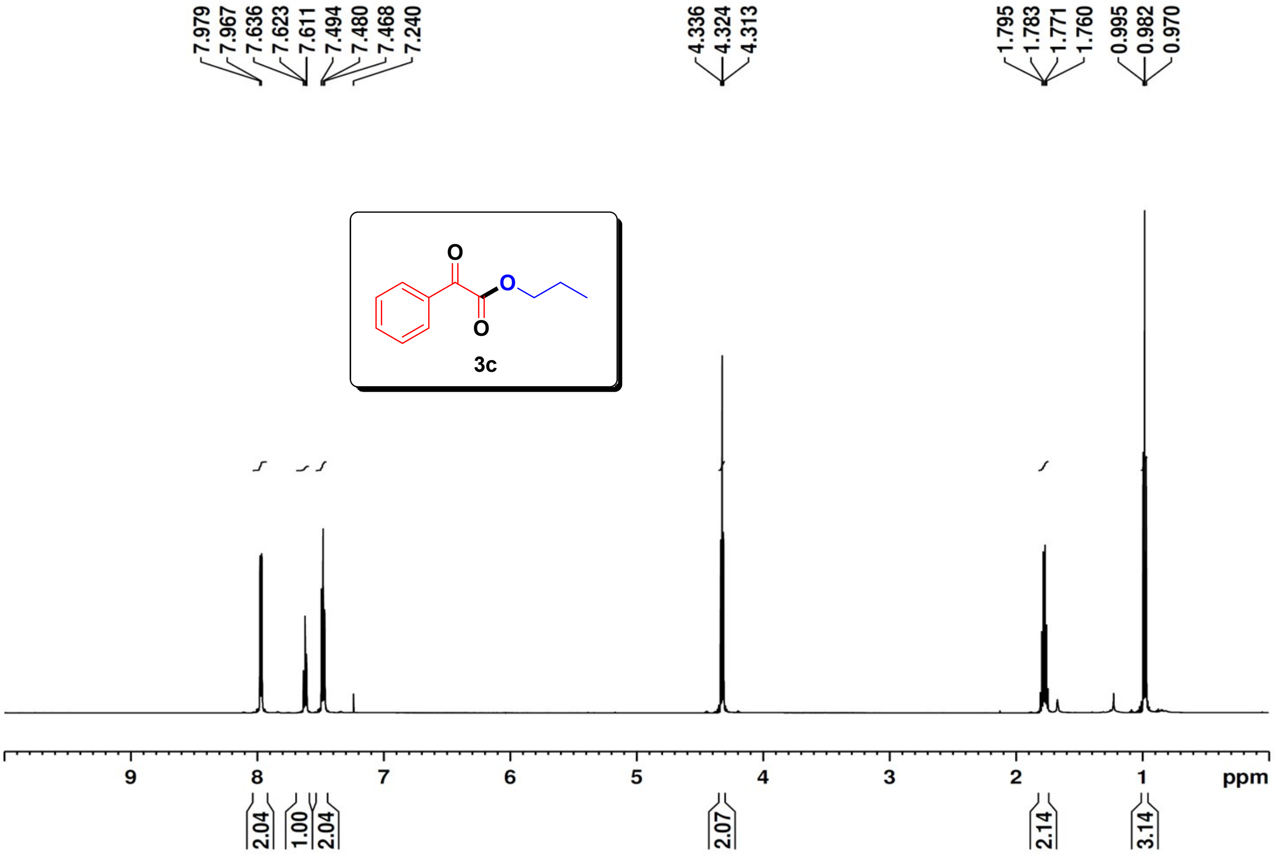
7.979
7.967
7.636
7.623
7.611
7.494
7.480
7.468
7.240
4.336
4.324
4.313
1.795
1.783
1.771
1.760
0.995
0.982
0.970
O
O
O
3c
9
8
7
6
5
4
3
2
1
ppm
2.04
1.00
2.04
2.07
2.14
3.14

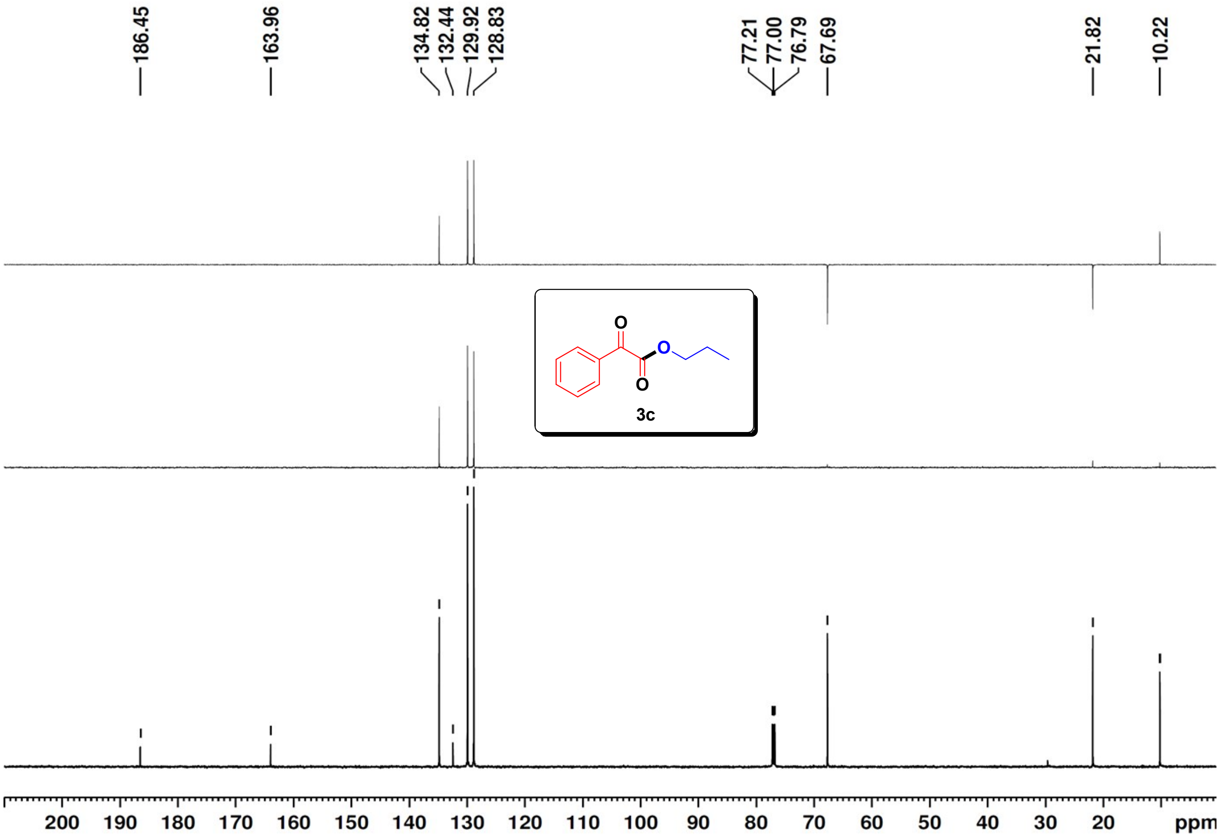
186.45
163.96
134.82
132.44
129.92
128.83
77.21
77.00
76.79
67.69
21.82
10.22
O
O
O
3c
200
190
180
170
160
150
140
130
120
110
100
90
80
70
60
50
40
30
20
ppm

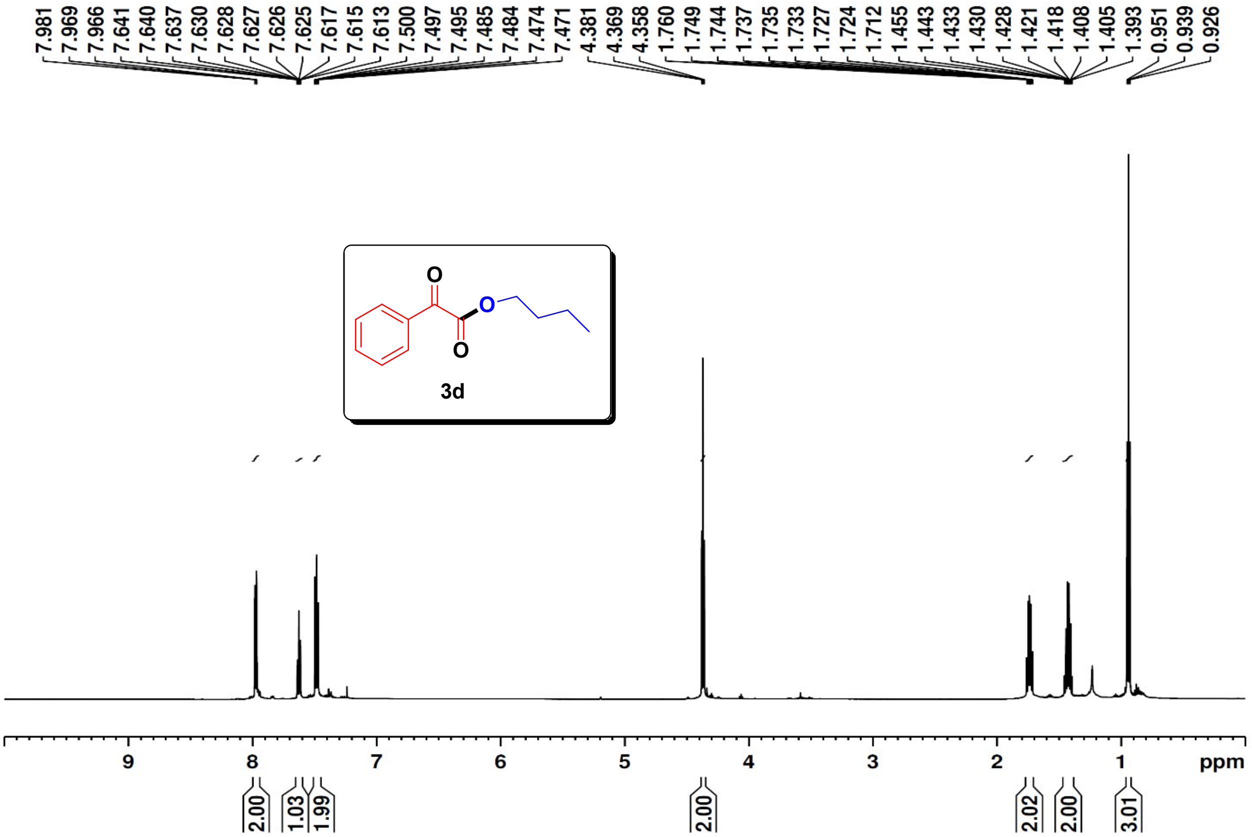
7.981 7.969 7.966 7.641 7.640 7.637 7.630 7.628 7.627 7.626 7.625 7.617 7.615 7.613 7.500 7.497 7.495 7.485 7.484 7.474 7.471 4.381 4.369 4.358 1.760 1.749 1.744 1.737 1.735 1.733 1.727 1.724 1.712 1.455 1.443 1.433 1.430 1.428 1.421 1.418 1.408 1.405 1.393 0.951 0.939 0.926
O
O
O
3d
9
8
7
6
5
4
3
2
1
ppm
2.00
1.03
1.99
2.00
2.02
2.00
3.01

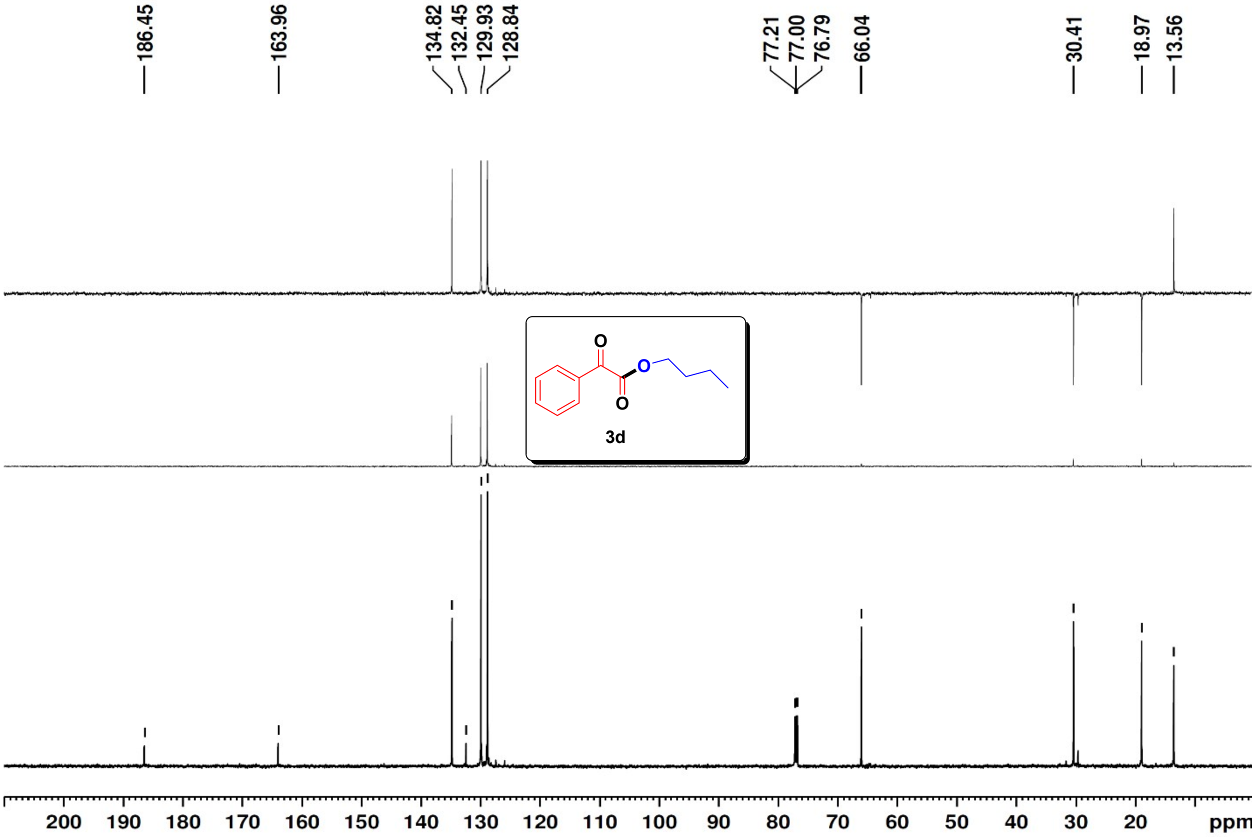
186.45
163.96
134.82 132.45 129.93 128.84
77.21 77.00 76.79
66.04
30.41
18.97
13.56
O
O
O
3d
200
190
180
170
160
150
140
130
120
110
100
90
80
70
60
50
40
30
20
ppm

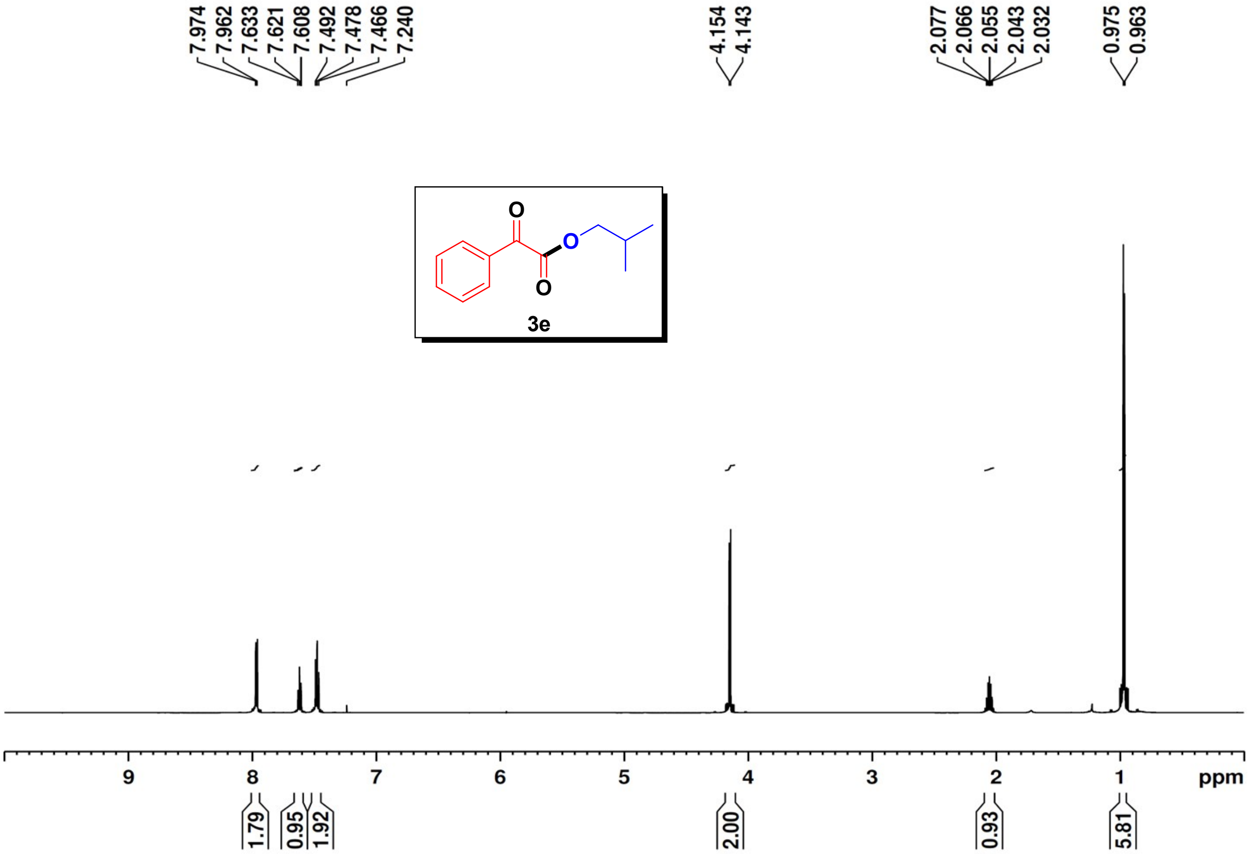
7.974
7.962
7.633
7.621
7.608
7.492
7.478
7.466
7.240
4.154
4.143
2.077
2.066
2.055
2.043
2.032
0.975
0.963
O
O
O
3e
9
8
7
6
5
4
3
2
1
ppm
1.79
0.95
1.92
2.00
0.93
5.81

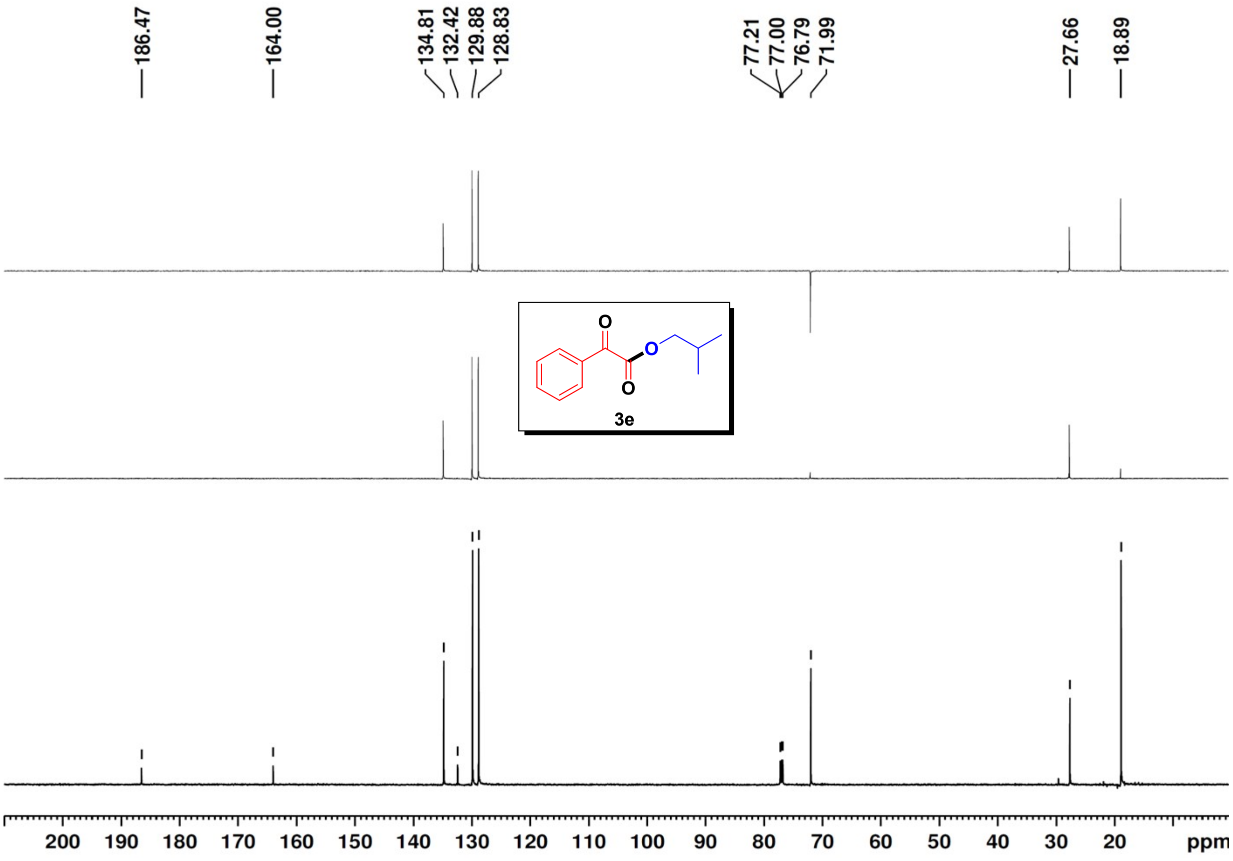
186.47
164.00
134.81
132.42
129.88
128.83
77.21
77.00
76.79
71.99
27.66
18.89
O
O
O
3e
200
190
180
170
160
150
140
130
120
110
100
90
80
70
60
50
40
30
20
ppm

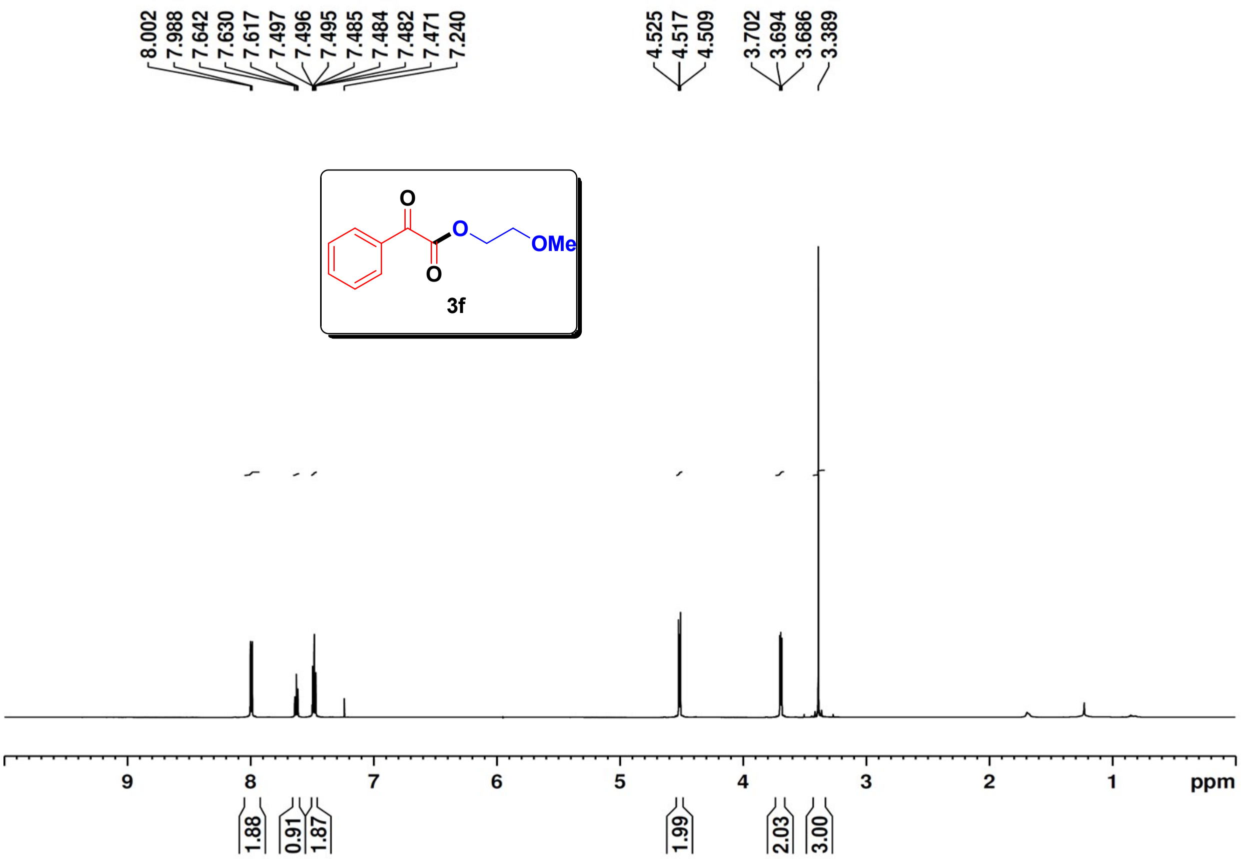
8.002
7.988
7.642
7.630
7.617
7.497
7.496
7.495
7.485
7.484
7.482
7.471
7.240
4.525
4.517
4.509
3.702
3.694
3.686
3.389
O
O
O
OMe
3f
9
8
7
6
5
4
3
2
1
ppm
1.88
0.91
1.87
1.99
2.03
3.00

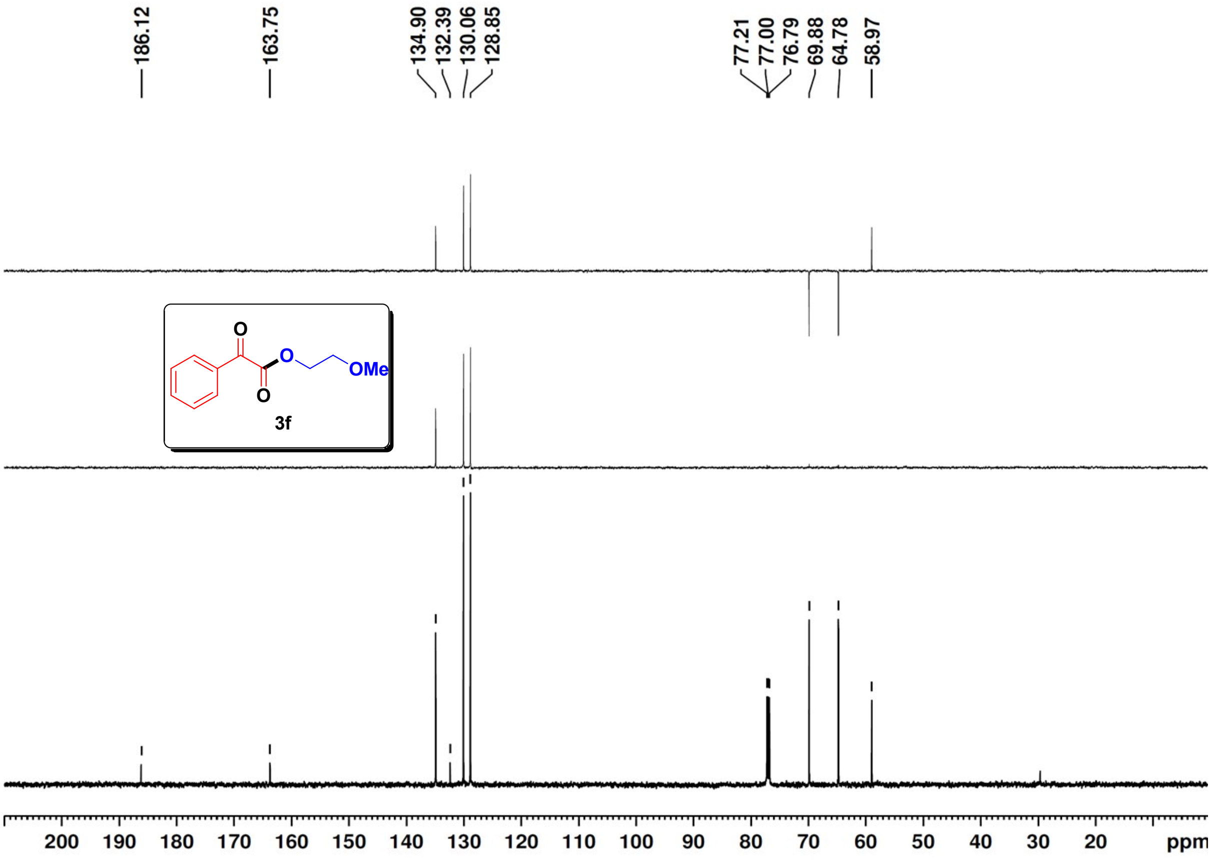
186.12
163.75
134.90
132.39
130.06
128.85
77.21
77.00
76.79
69.88
64.78
58.97
O
O
O
OMe
3f
200
190
180
170
160
150
140
130
120
110
100
90
80
70
60
50
40
30
20
ppm

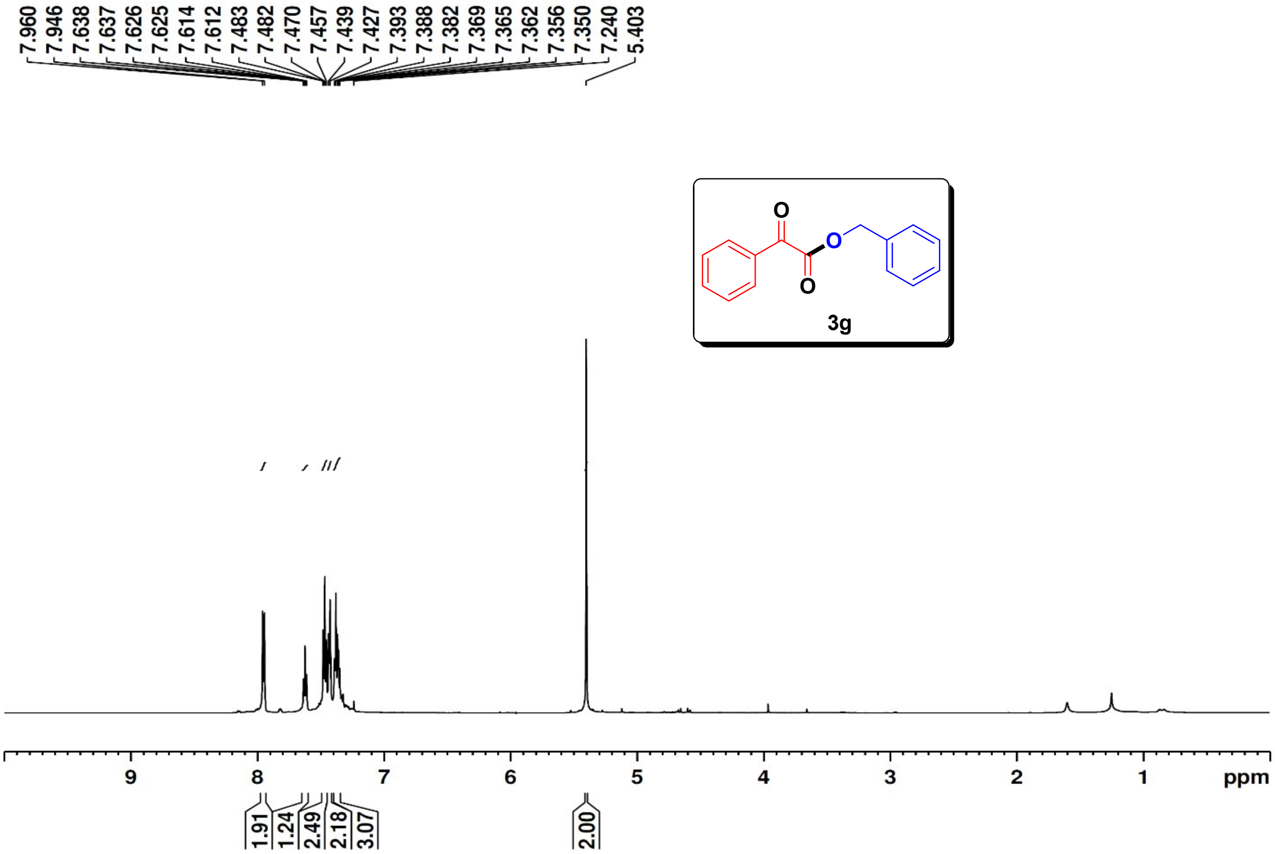
7.960
7.946
7.638
7.637
7.626
7.625
7.614
7.612
7.483
7.482
7.470
7.457
7.439
7.427
7.393
7.388
7.382
7.369
7.365
7.362
7.356
7.350
7.240
5.403
O
O
O
3g
9
8
7
6
5
4
3
2
1
ppm
1.91
1.24
2.49
2.18
3.07
2.00

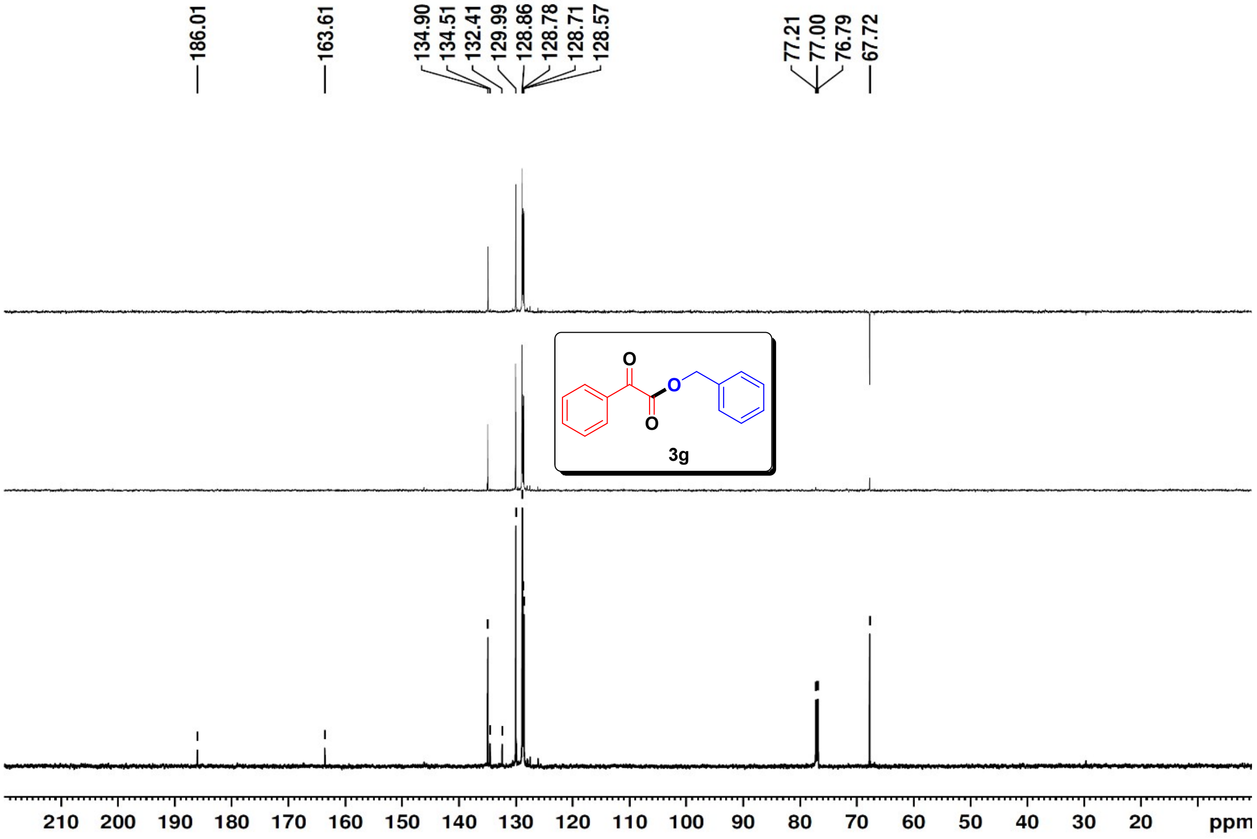
186.01
163.61
134.90
134.51
132.41
129.99
128.86
128.78
128.71
128.57
77.21
77.00
76.79
67.72
O
O
O
3g
210
200
190
180
170
160
150
140
130
120
110
100
90
80
70
60
50
40
30
20
ppm

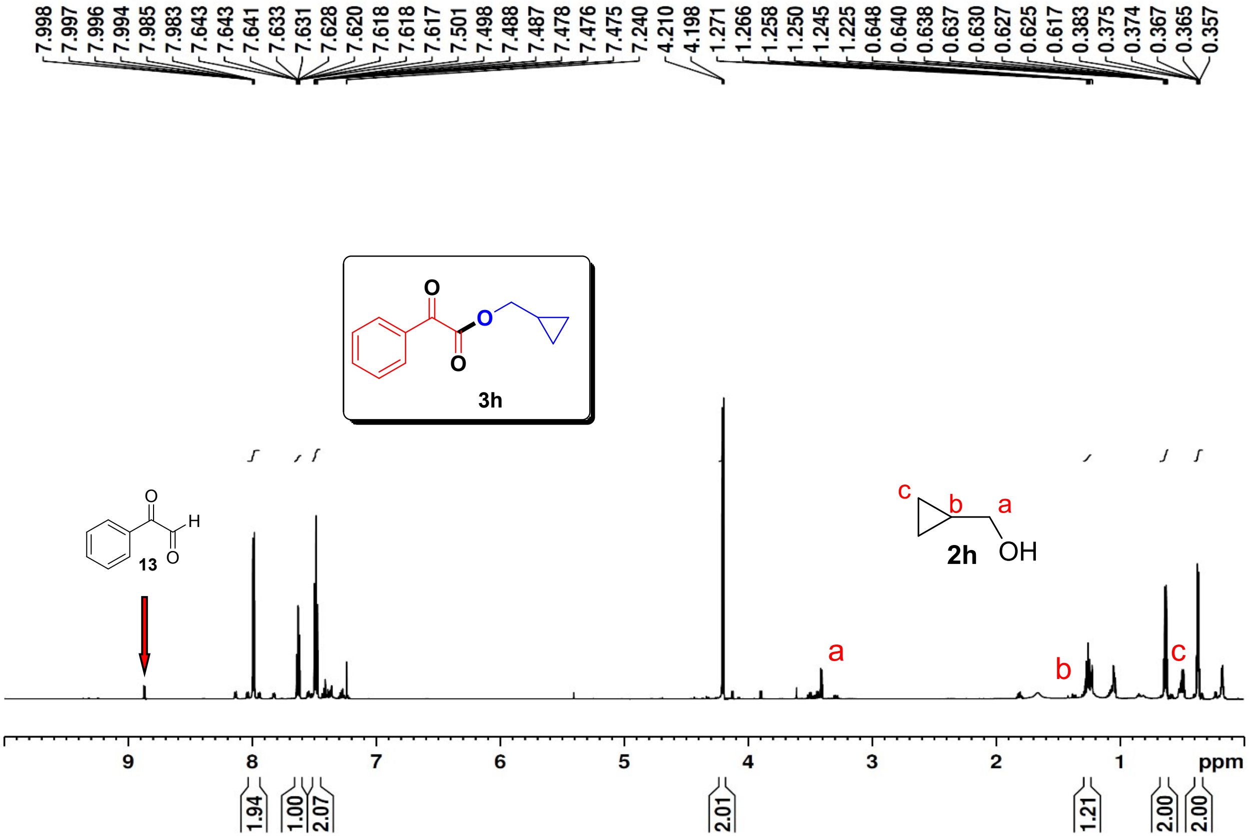
7.998
7.997
7.996
7.994
7.985
7.983
7.643
7.643
7.641
7.633
7.631
7.628
7.620
7.618
7.618
7.617
7.501
7.498
7.488
7.487
7.478
7.476
7.475
7.240
4.210
4.198
1.271
1.266
1.258
1.250
1.245
1.225
0.648
0.640
0.638
0.637
0.630
0.627
0.625
0.617
0.383
0.375
0.374
0.367
0.365
0.357
3h
13
c
b
a
2h
OH
a
b
c
9
8
7
6
5
4
3
2
1
ppm
1.94
1.00
2.07
2.01
1.21
2.00
2.00

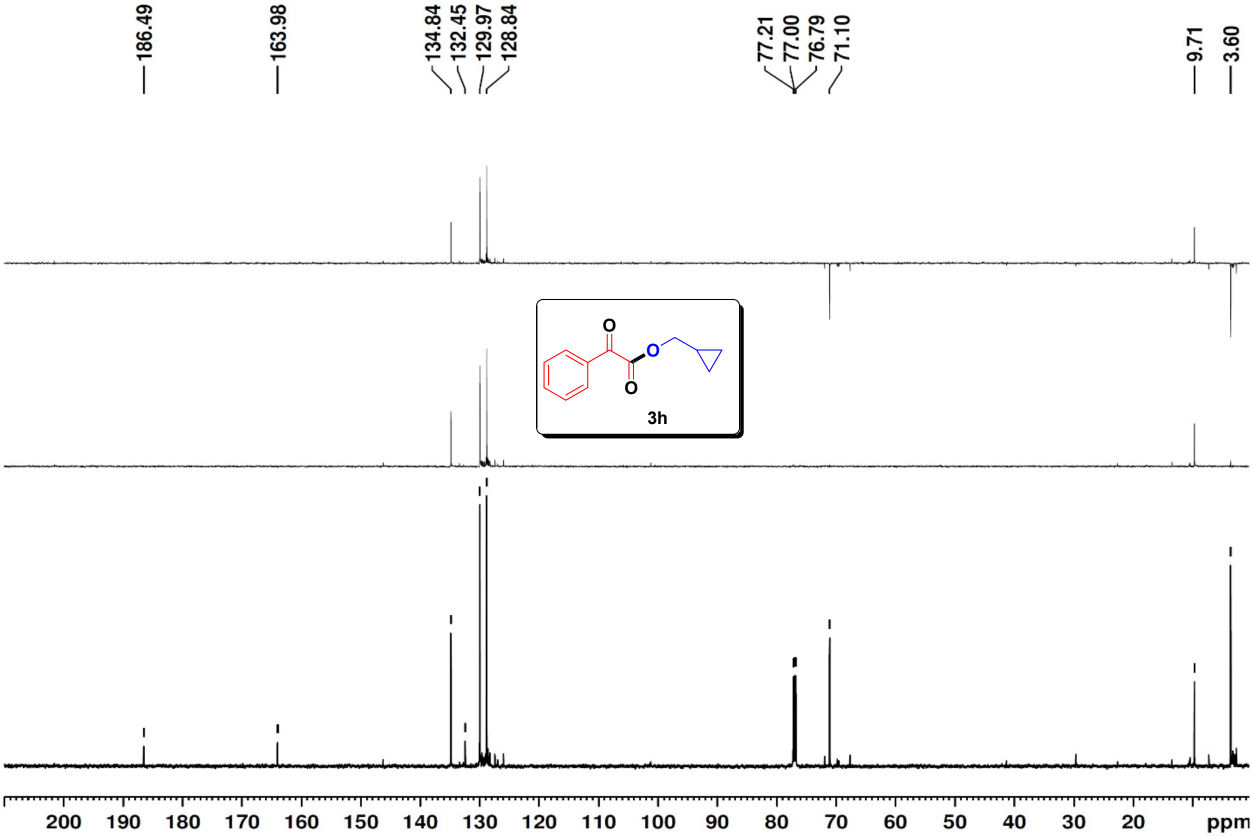
186.49
163.98
134.84
132.45
129.97
128.84
77.21
77.00
76.79
71.10
9.71
3.60
3h
200
190
180
170
160
150
140
130
120
110
100
90
80
70
60
50
40
30
20
ppm

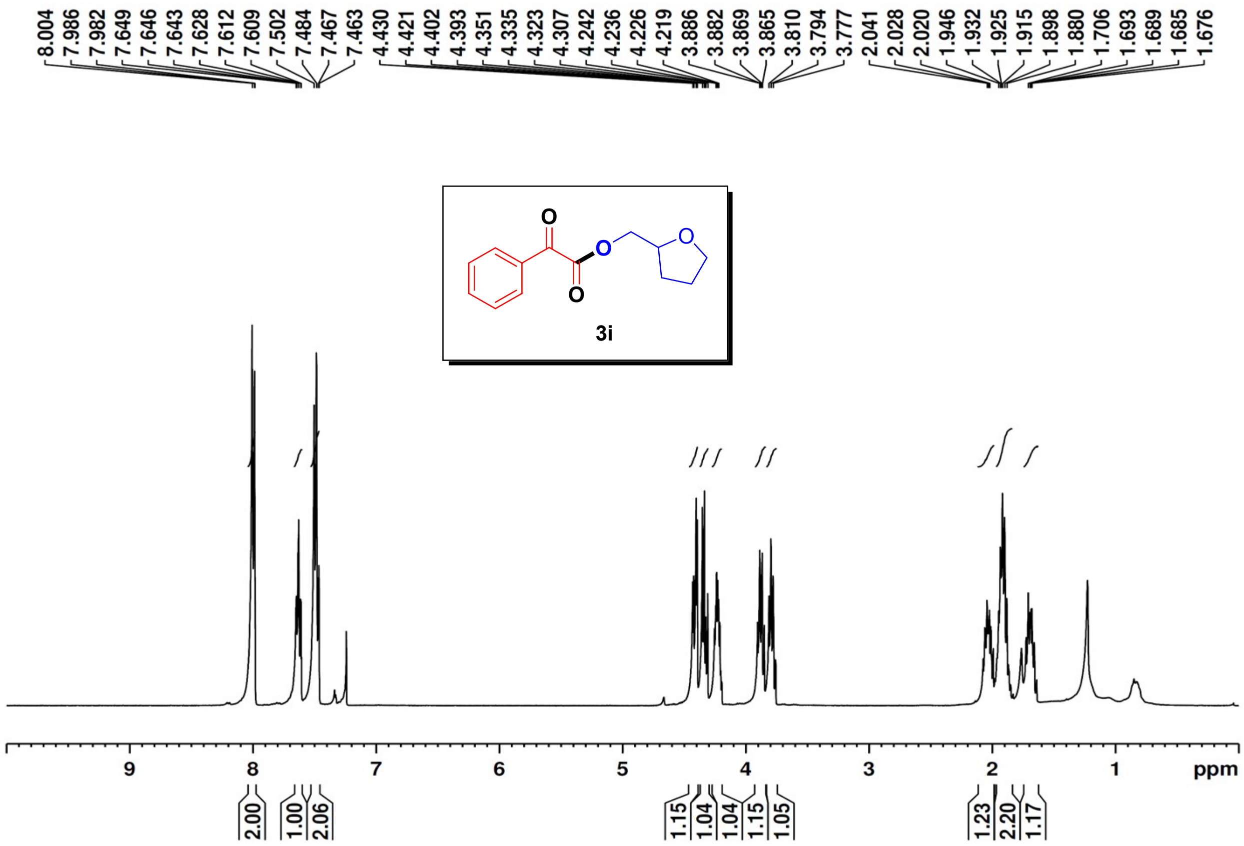
8.004
7.986
7.982
7.649
7.646
7.643
7.628
7.612
7.609
7.502
7.484
7.467
7.463
4.430
4.421
4.402
4.393
4.351
4.335
4.323
4.307
4.242
4.236
4.226
4.219
3.886
3.882
3.869
3.865
3.810
3.794
3.777
2.041
2.028
2.020
1.946
1.932
1.925
1.915
1.898
1.880
1.706
1.693
1.689
1.685
1.676
O
O
O
O
3i
9 8 7 6 5 4 3 2 1 ppm
2.00
1.00
2.06
1.15
1.04
1.04
1.15
1.05
1.23
2.20
1.17

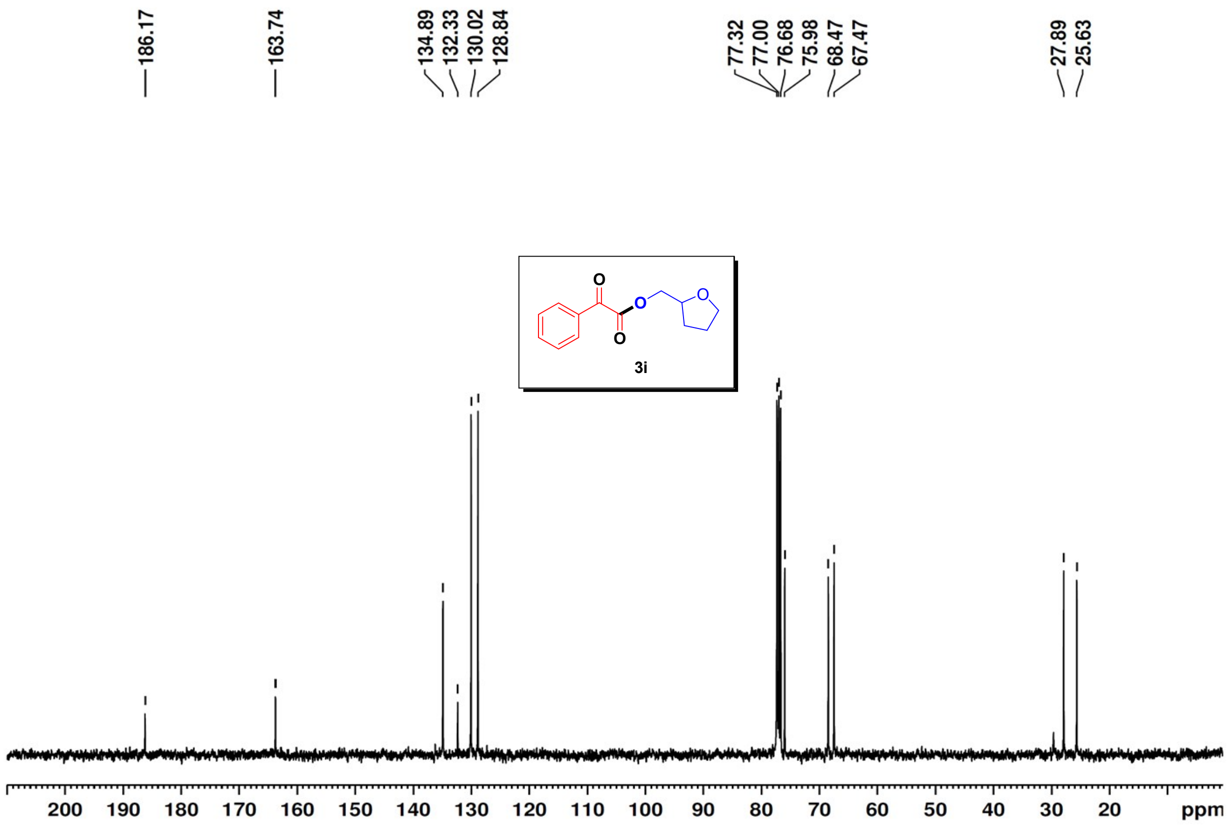
186.17
163.74
134.89
132.33
130.02
128.84
77.32
77.00
76.68
75.98
68.47
67.47
27.89
25.63
O
O
O
O
3i
200 190 180 170 160 150 140 130 120 110 100 90 80 70 60 50 40 30 20 ppm

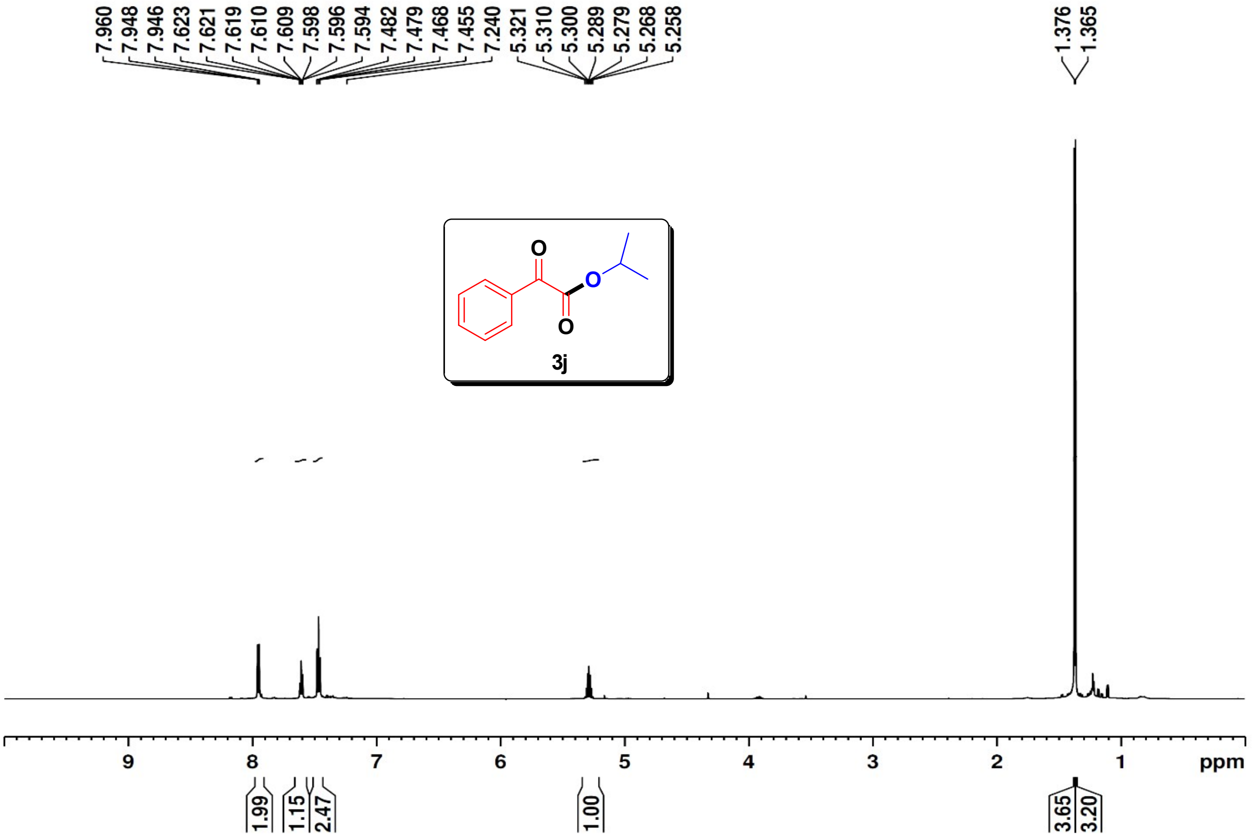

7.960
7.948
7.946
7.623
7.621
7.619
7.610
7.609
7.598
7.596
7.594
7.482
7.479
7.468
7.455
7.240
5.321
5.310
5.300
5.289
5.279
5.268
5.258
1.376
1.365
O
O
O
3j
9
8
7
6
5
4
3
2
1
ppm
1.99
1.15
2.47
1.00
3.65
3.20

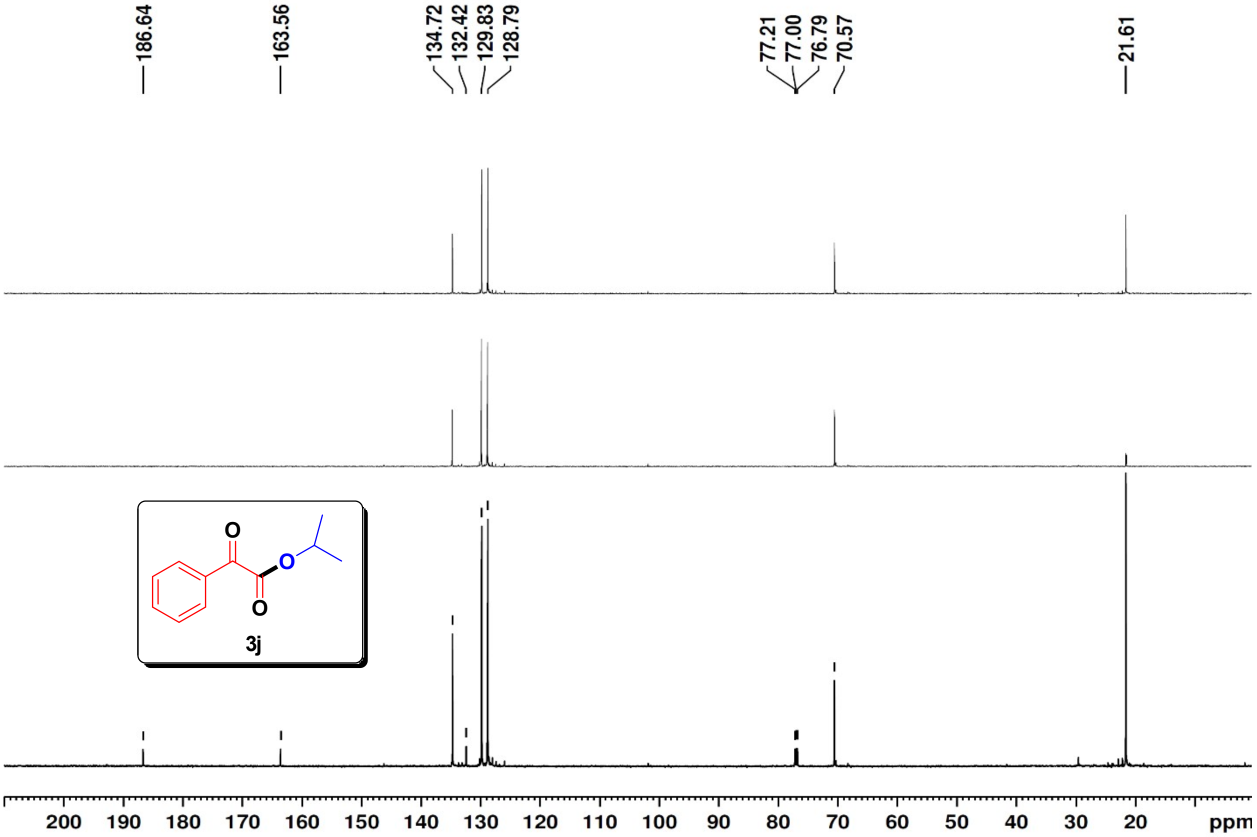

186.64
163.56
134.72
132.42
129.83
128.79
77.21
77.00
76.79
70.57
21.61
O
O
O
3j
200
190
180
170
160
150
140
130
120
110
100
90
80
70
60
50
40
30
20
ppm

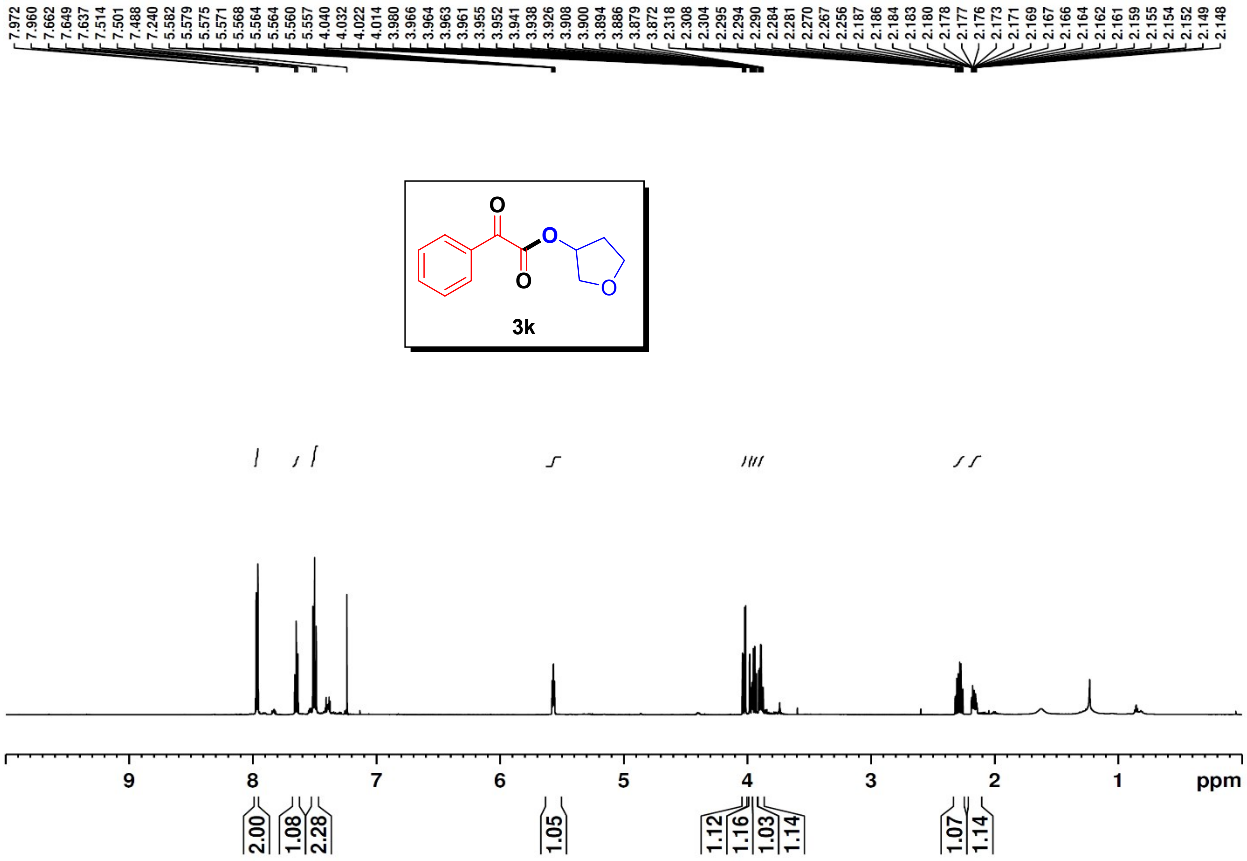
7.972
7.960
7.662
7.649
7.637
7.514
7.501
7.488
7.240
5.582
5.579
5.575
5.571
5.568
5.564
5.564
5.560
5.557
4.040
4.032
4.022
4.014
3.980
3.966
3.964
3.963
3.961
3.955
3.952
3.941
3.938
3.926
3.908
3.900
3.894
3.886
3.879
3.872
2.318
2.308
2.304
2.295
2.294
2.290
2.284
2.281
2.270
2.267
2.256
2.187
2.186
2.184
2.183
2.180
2.178
2.177
2.176
2.173
2.171
2.169
2.167
2.166
2.164
2.162
2.161
2.159
2.155
2.154
2.152
2.149
2.148
O
O
O
O
3k
9
8
7
6
5
4
3
2
1
ppm
2.00
1.08
2.28
1.05
1.12
1.16
1.03
1.14
1.07
1.14

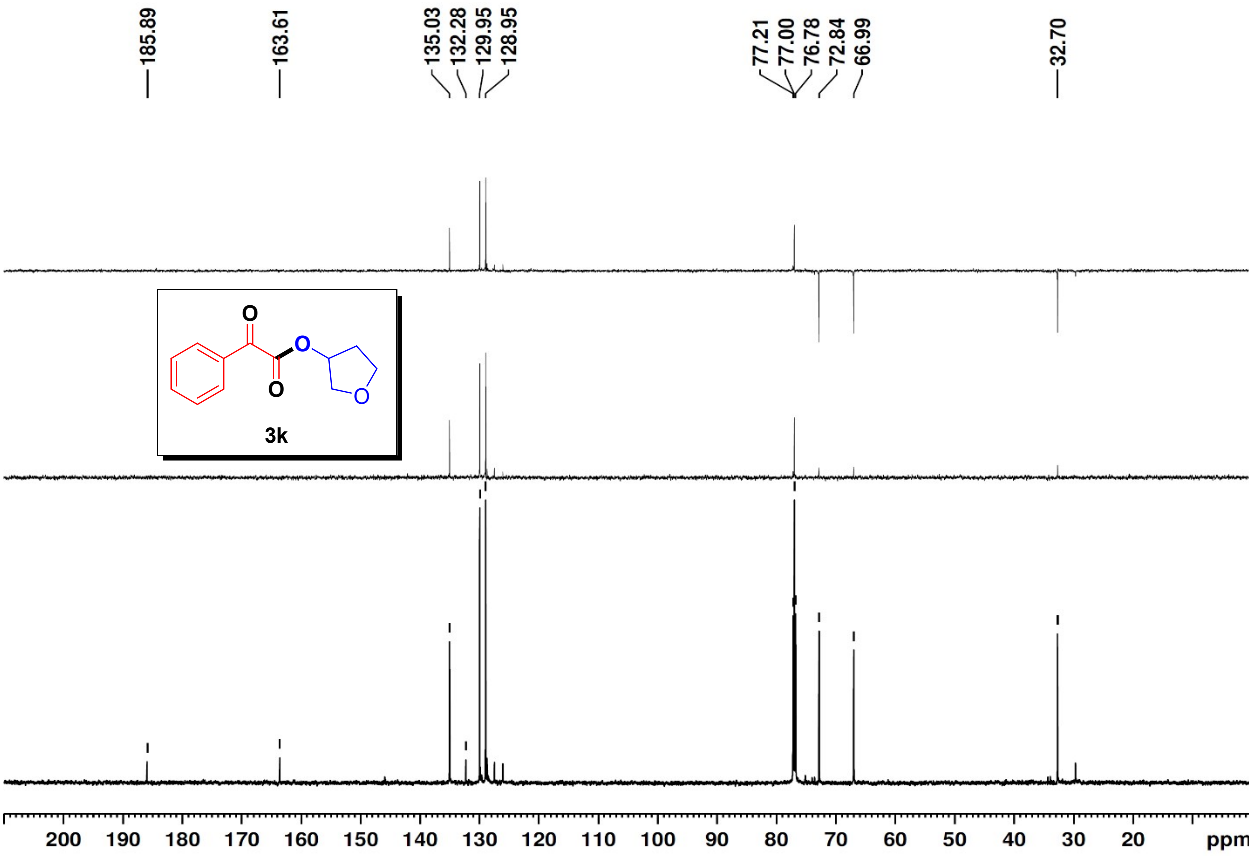
185.89
163.61
135.03
132.28
129.95
128.95
77.21
77.00
76.78
72.84
66.99
32.70
O
O
O
O
3k
200
190
180
170
160
150
140
130
120
110
100
90
80
70
60
50
40
30
20
ppm

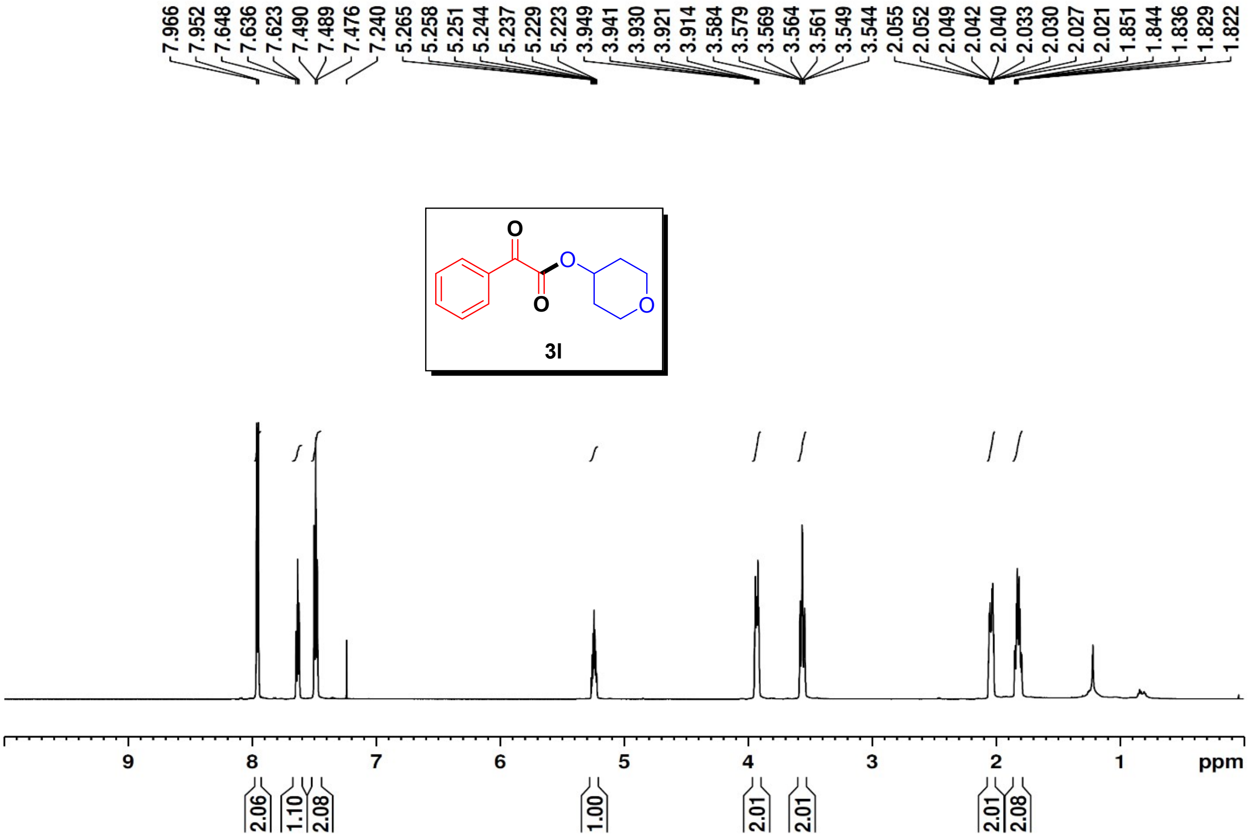
7.966
7.952
7.648
7.636
7.623
7.490
7.489
7.476
7.240
5.265
5.258
5.251
5.244
5.237
5.229
5.223
3.949
3.941
3.930
3.921
3.914
3.584
3.579
3.569
3.564
3.561
3.549
3.544
2.055
2.052
2.049
2.042
2.040
2.033
2.030
2.027
2.021
1.851
1.844
1.836
1.829
1.822
3l
9
8
7
6
5
4
3
2
1
ppm
2.06
1.10
2.08
1.00
2.01
2.01
2.01
2.08

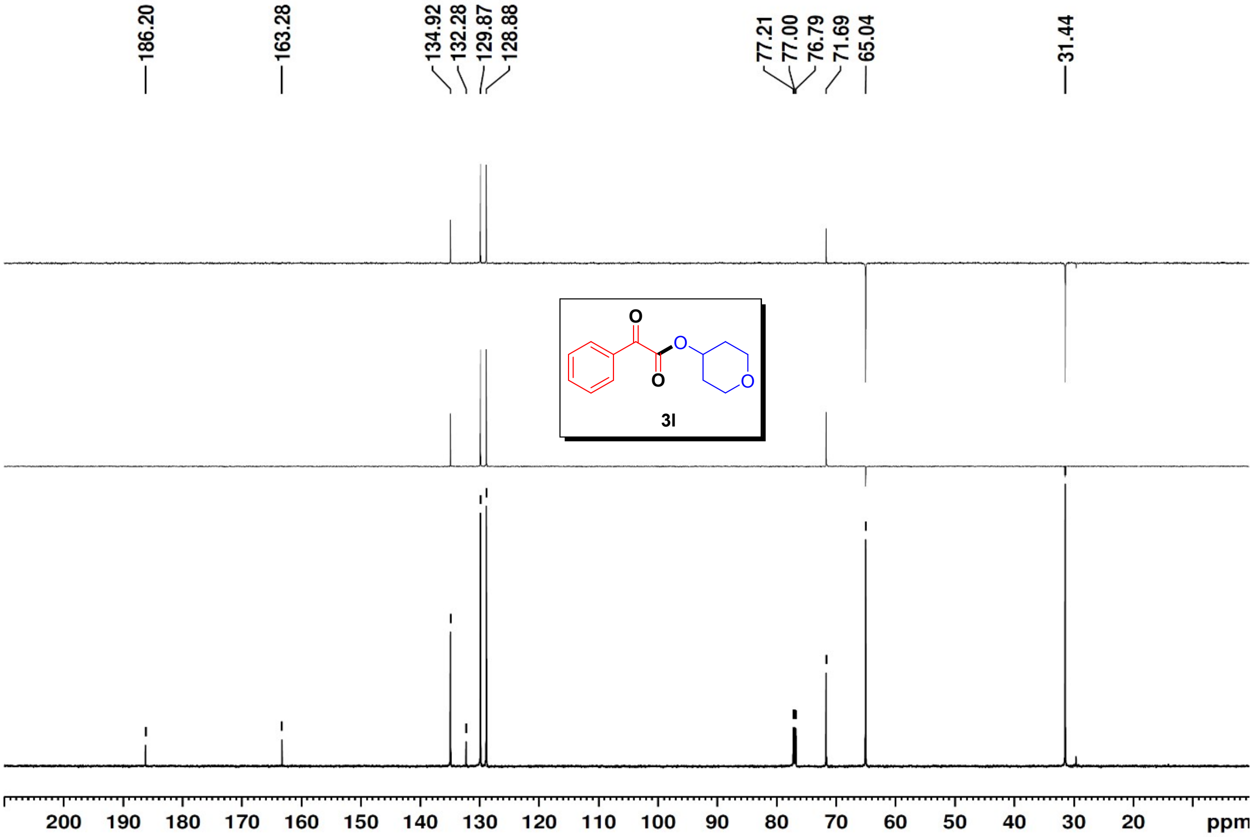
186.20
163.28
134.92
132.28
129.87
128.88
77.21
77.00
76.79
71.69
65.04
31.44
3l
200
190
180
170
160
150
140
130
120
110
100
90
80
70
60
50
40
30
20
ppm

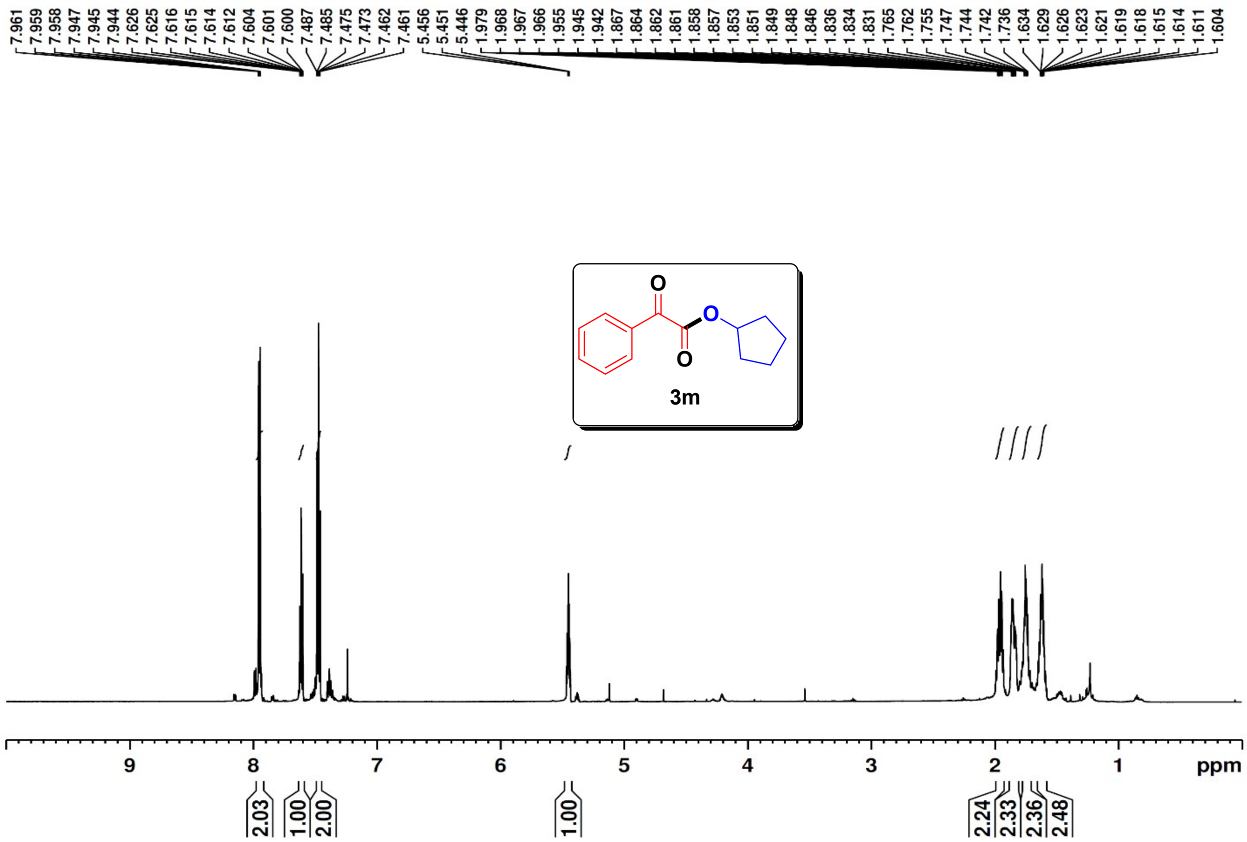
7.961
7.959
7.958
7.947
7.945
7.944
7.626
7.625
7.616
7.615
7.614
7.612
7.604
7.601
7.600
7.487
7.485
7.475
7.473
7.462
7.461
5.456
5.451
5.446
1.979
1.968
1.967
1.966
1.955
1.945
1.942
1.867
1.864
1.862
1.861
1.858
1.857
1.853
1.851
1.849
1.848
1.846
1.836
1.834
1.831
1.765
1.762
1.755
1.747
1.744
1.742
1.736
1.634
1.629
1.626
1.623
1.621
1.619
1.618
1.615
1.614
1.611
1.604
O
O
O
3m
9
8
7
6
5
4
3
2
1
ppm
2.03
1.00
2.00
1.00
2.24
2.33
2.36
2.48

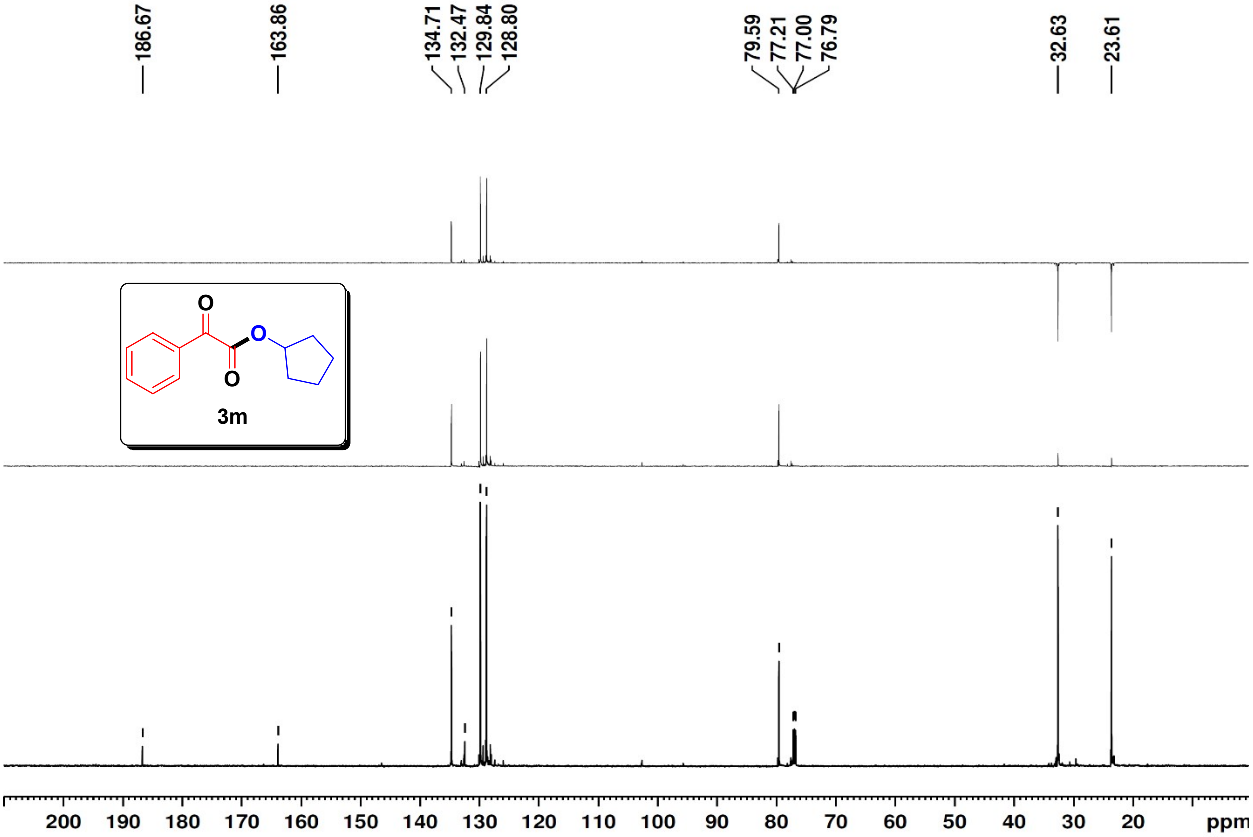
186.67
163.86
134.71
132.47
129.84
128.80
79.59
77.21
77.00
76.79
32.63
23.61
O
O
O
3m
200
190
180
170
160
150
140
130
120
110
100
90
80
70
60
50
40
30
20
ppm

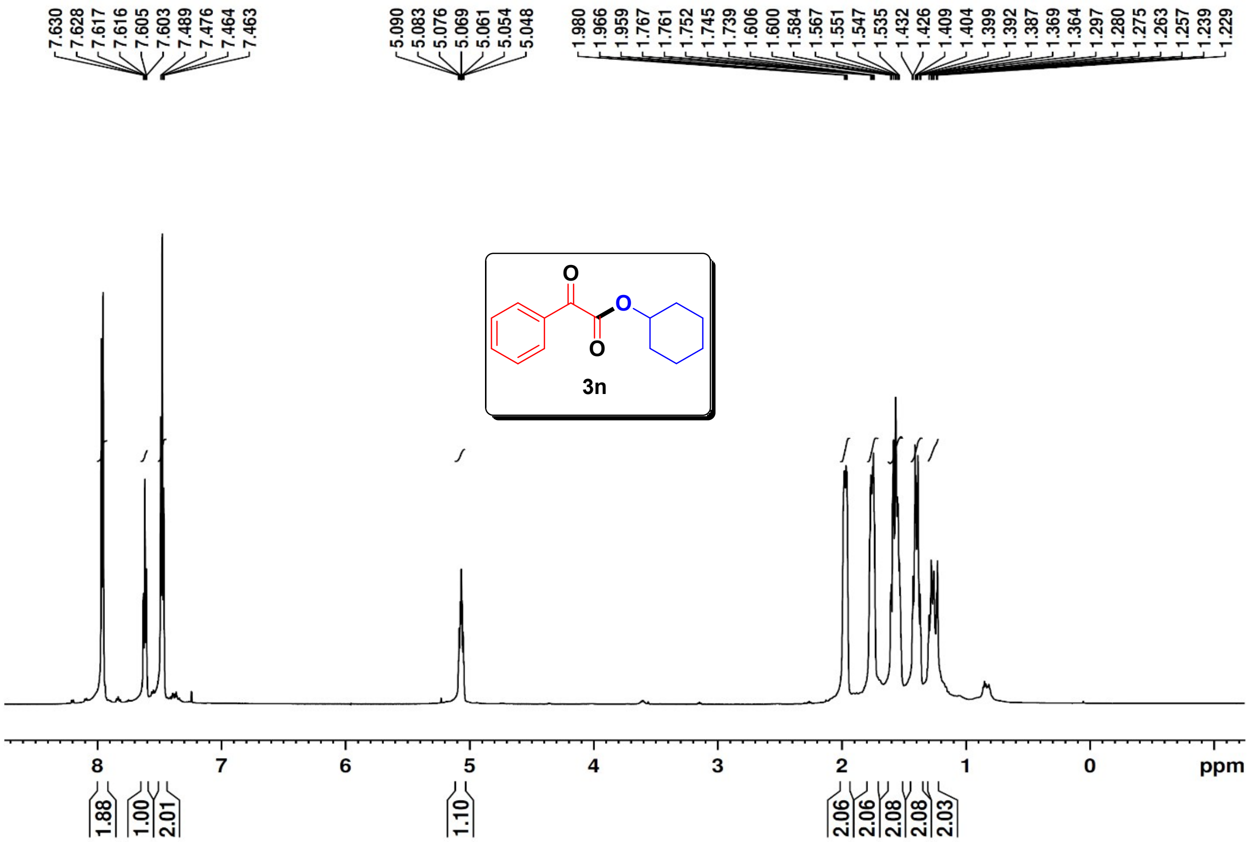

7.630
7.628
7.617
7.616
7.605
7.603
7.489
7.476
7.464
7.463
5.090
5.083
5.076
5.069
5.061
5.054
5.048
1.980
1.966
1.959
1.767
1.761
1.752
1.745
1.739
1.606
1.600
1.584
1.567
1.551
1.547
1.535
1.432
1.426
1.409
1.404
1.399
1.392
1.387
1.369
1.364
1.297
1.280
1.275
1.263
1.257
1.239
1.229
3n
1.88
1.00
2.01
1.10
2.06
2.06
2.08
2.08
2.03
ppm

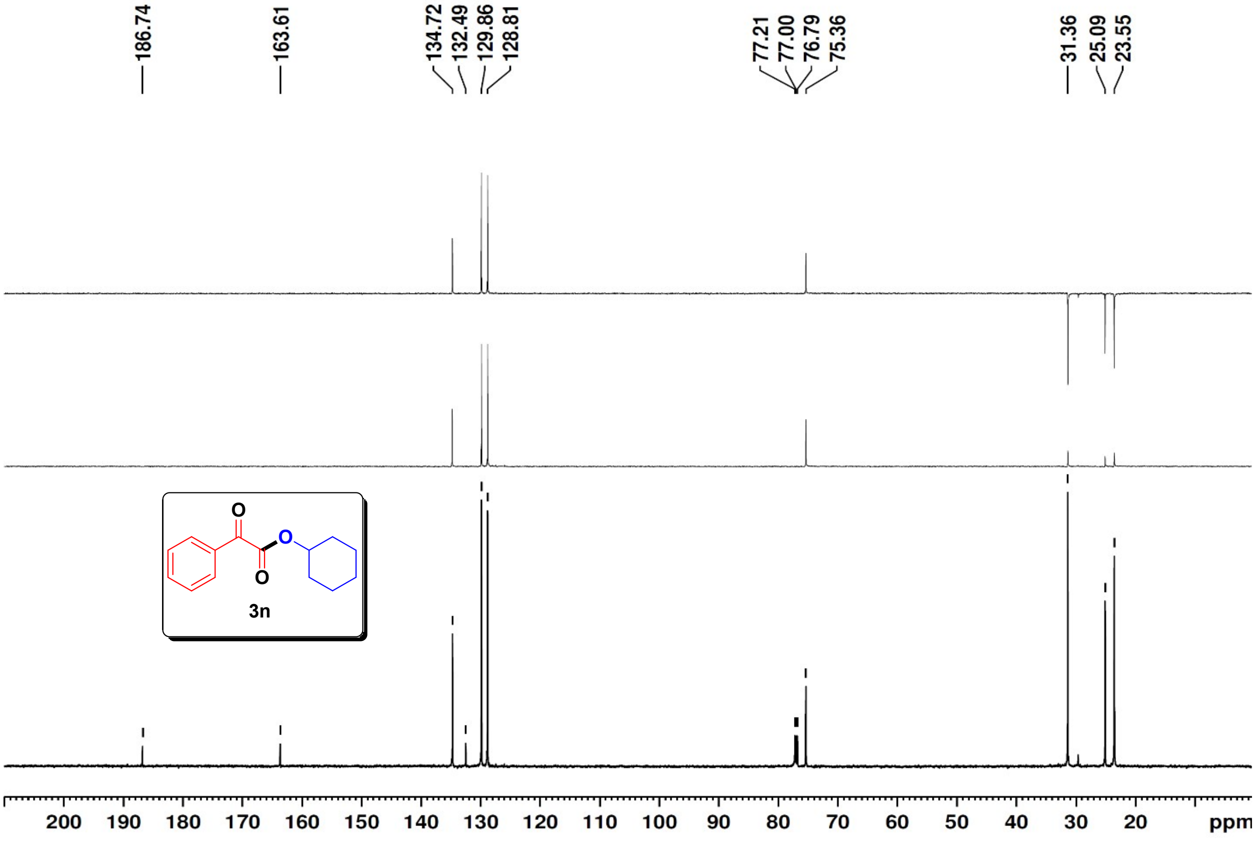

186.74
163.61
134.72
132.49
129.86
128.81
77.21
77.00
76.79
75.36
31.36
25.09
23.55
3n
ppm

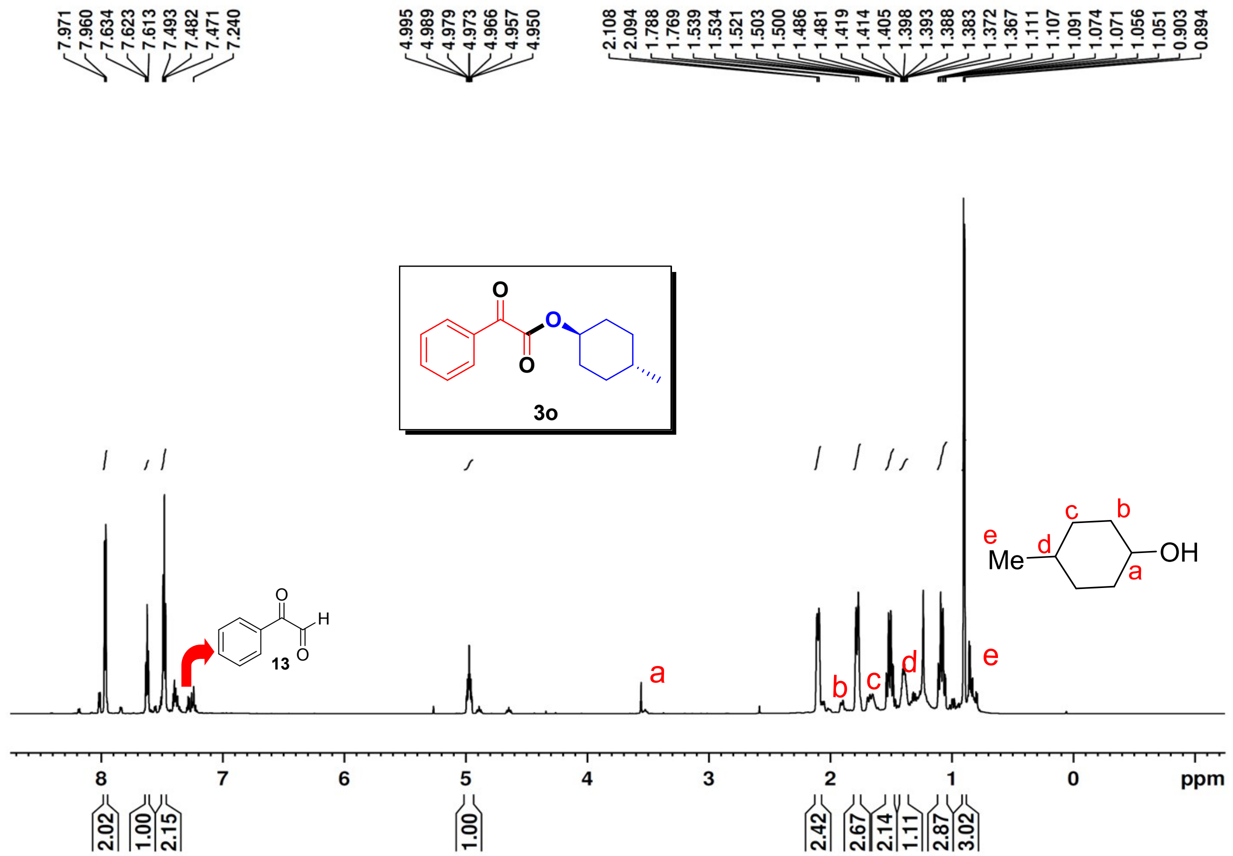

7.971
7.960
7.634
7.623
7.613
7.493
7.482
7.471
7.240
4.995
4.989
4.979
4.973
4.966
4.957
4.950
2.108
2.094
1.788
1.769
1.539
1.534
1.521
1.503
1.500
1.486
1.481
1.419
1.414
1.405
1.398
1.393
1.388
1.383
1.372
1.367
1.111
1.107
1.091
1.074
1.071
1.056
1.051
0.903
0.894
3o
13
a
b
c
d
e
Me
OH
ppm
2.02
1.00
2.15
1.00
2.42
2.67
2.14
1.11
2.87
3.02

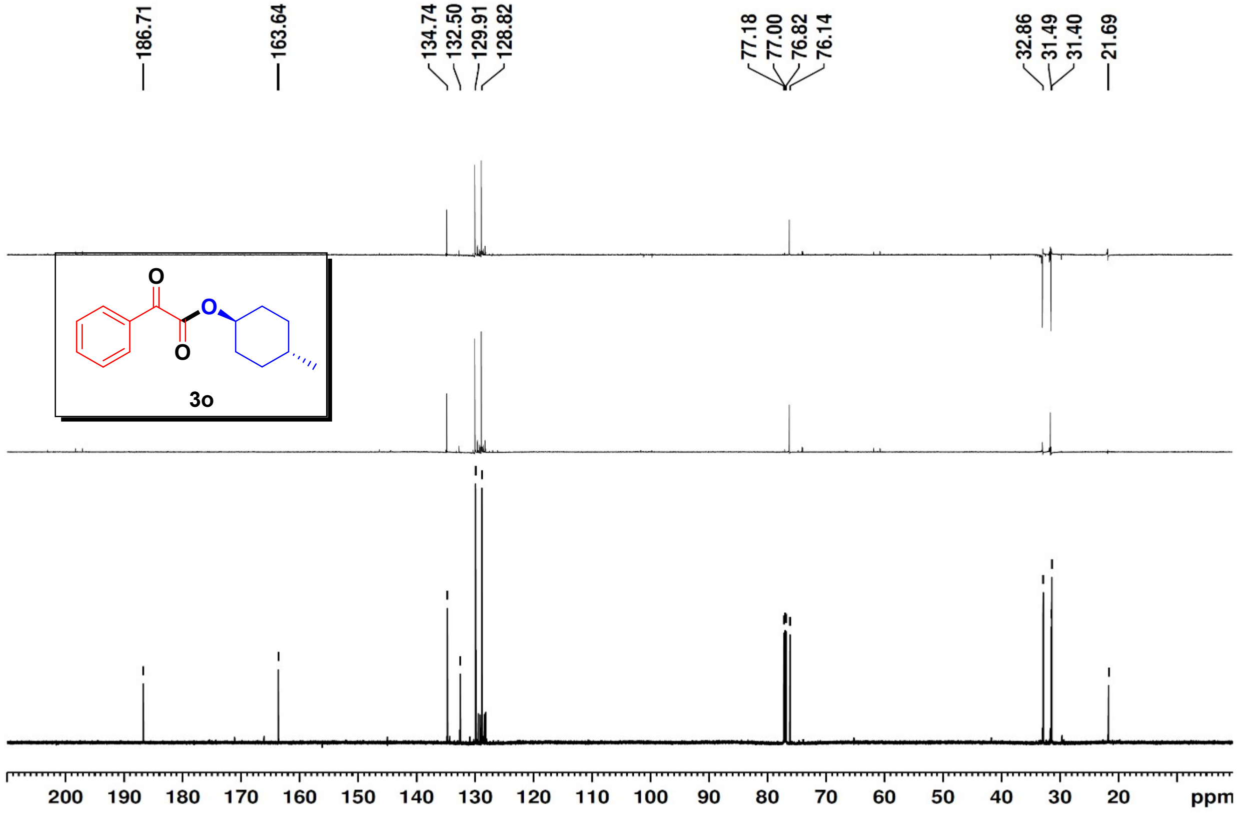

186.71
163.64
134.74
132.50
129.91
128.82
77.18
77.00
76.82
76.14
32.86
31.49
31.40
21.69
3o
ppm

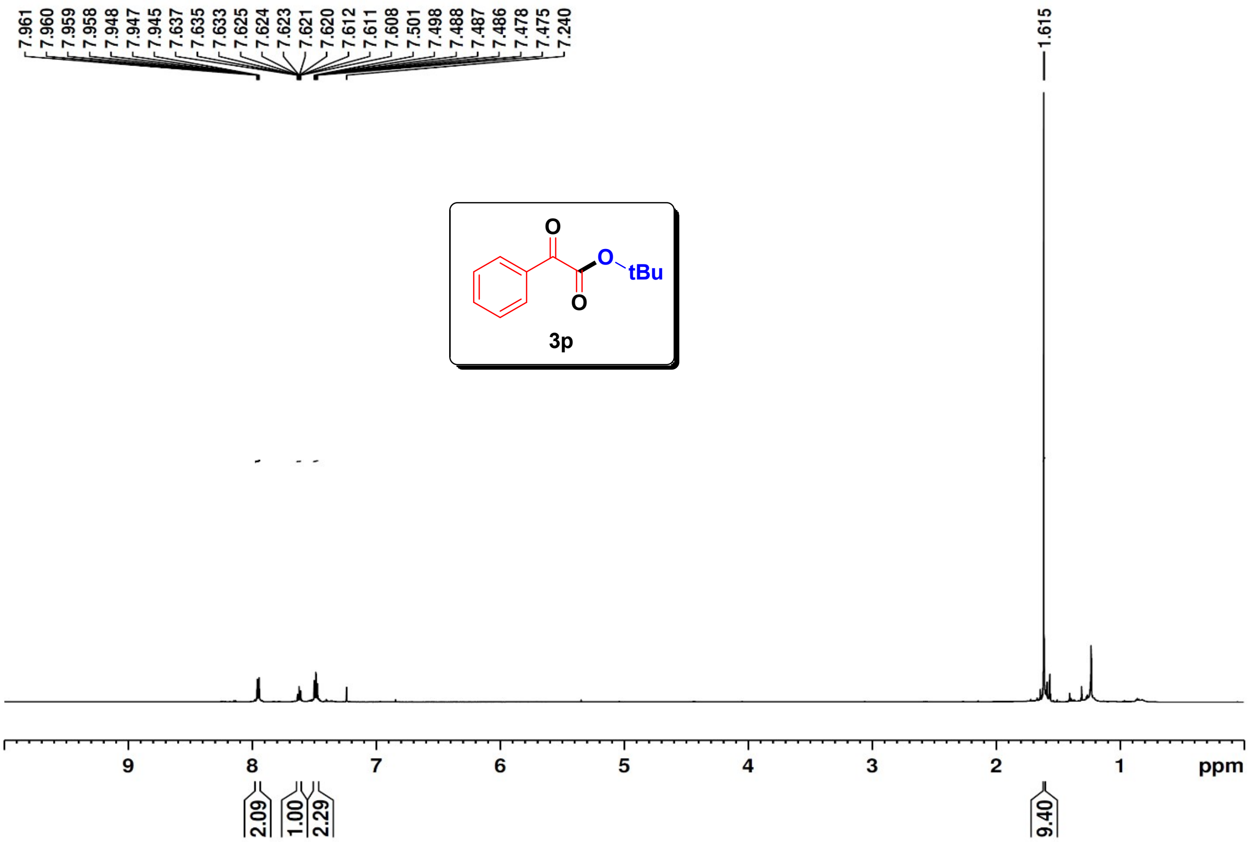
7.961
7.960
7.959
7.958
7.948
7.947
7.945
7.637
7.635
7.633
7.625
7.624
7.623
7.621
7.620
7.612
7.611
7.608
7.501
7.498
7.488
7.487
7.486
7.478
7.475
7.240
1.615
O
O
tBu
O
3p
9
8
7
6
5
4
3
2
1
ppm
2.09
1.00
2.29
9.40

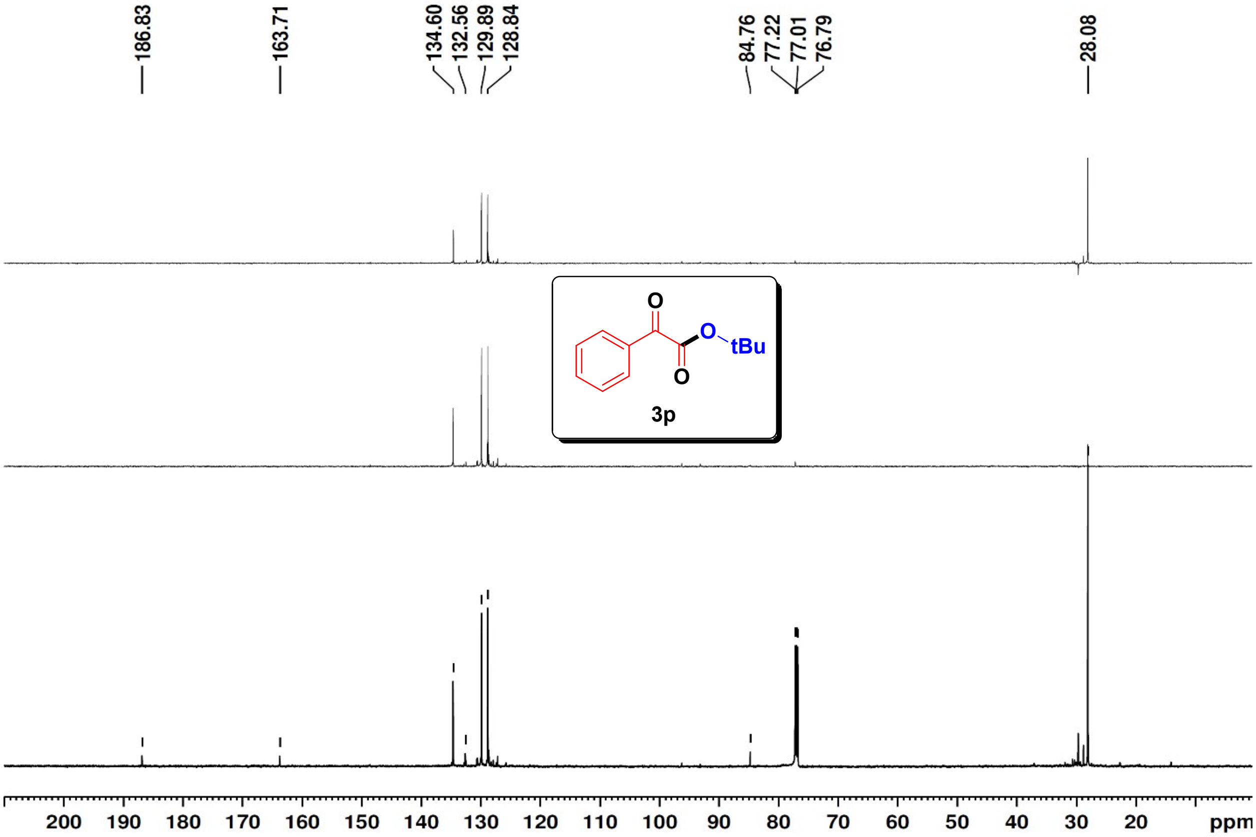
186.83
163.71
134.60
132.56
129.89
128.84
84.76
77.22
77.01
76.79
28.08
O
O
tBu
O
3p
200
190
180
170
160
150
140
130
120
110
100
90
80
70
60
50
40
30
20
ppm

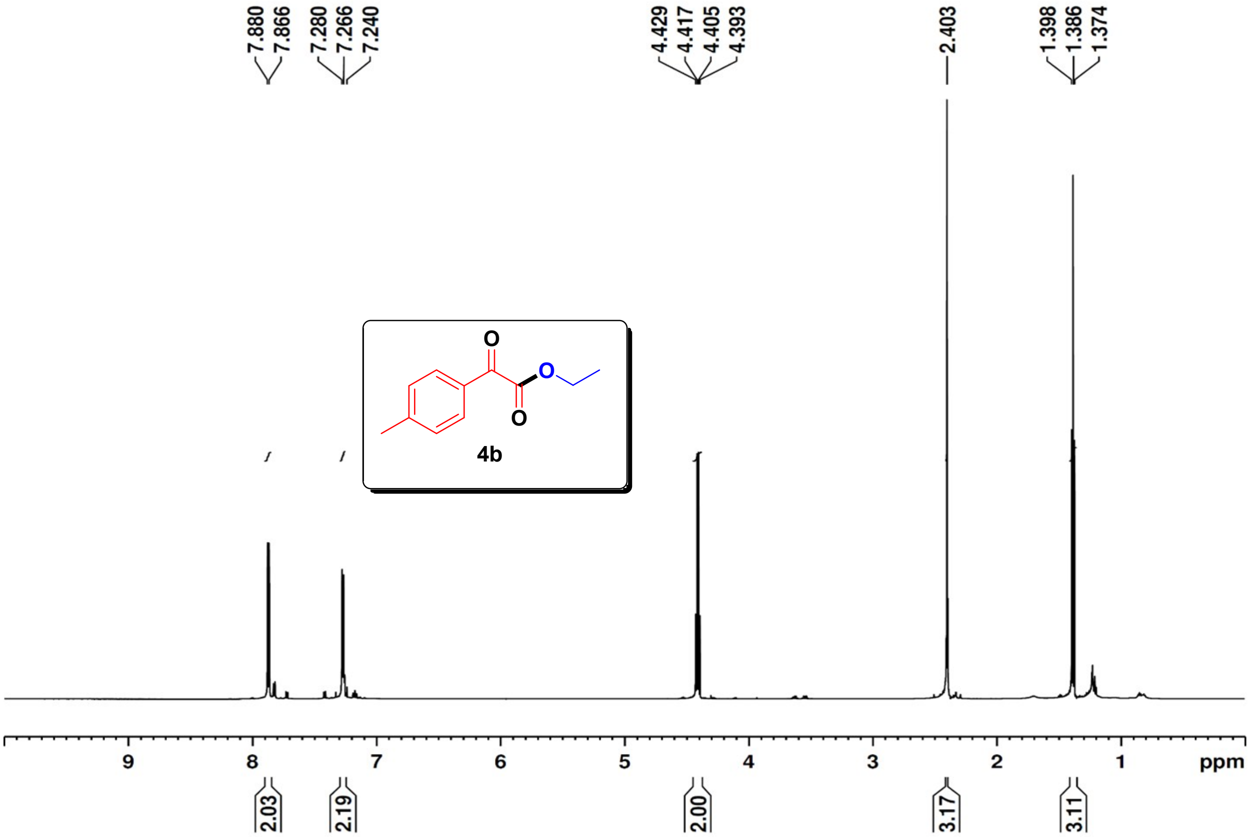
7.880
7.866
7.280
7.266
7.240
4.429
4.417
4.405
4.393
2.403
1.398
1.386
1.374
O
O
O
4b
9
8
7
6
5
4
3
2
1
ppm
2.03
2.19
2.00
3.17
3.11

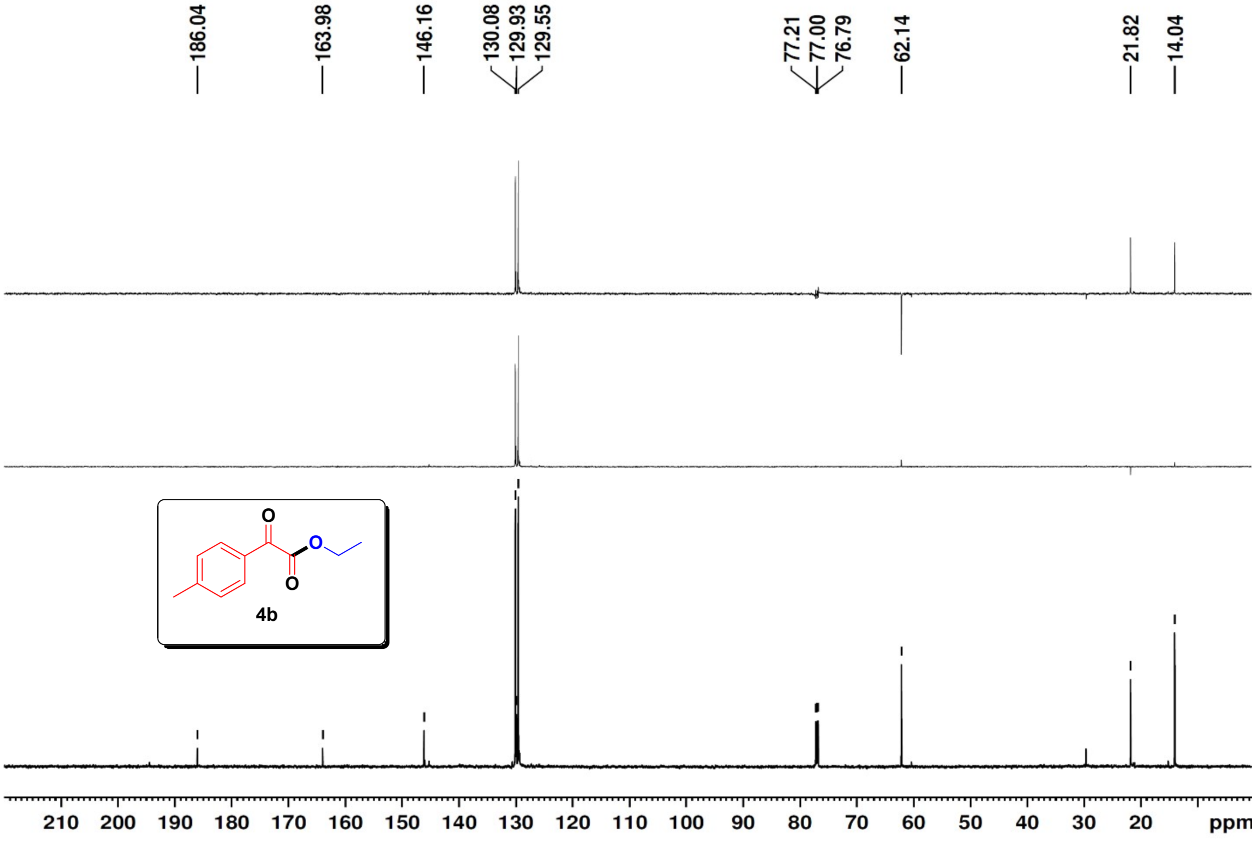
186.04
163.98
146.16
130.08
129.93
129.55
77.21
77.00
76.79
62.14
21.82
14.04
O
O
O
4b
210
200
190
180
170
160
150
140
130
120
110
100
90
80
70
60
50
40
30
20
ppm

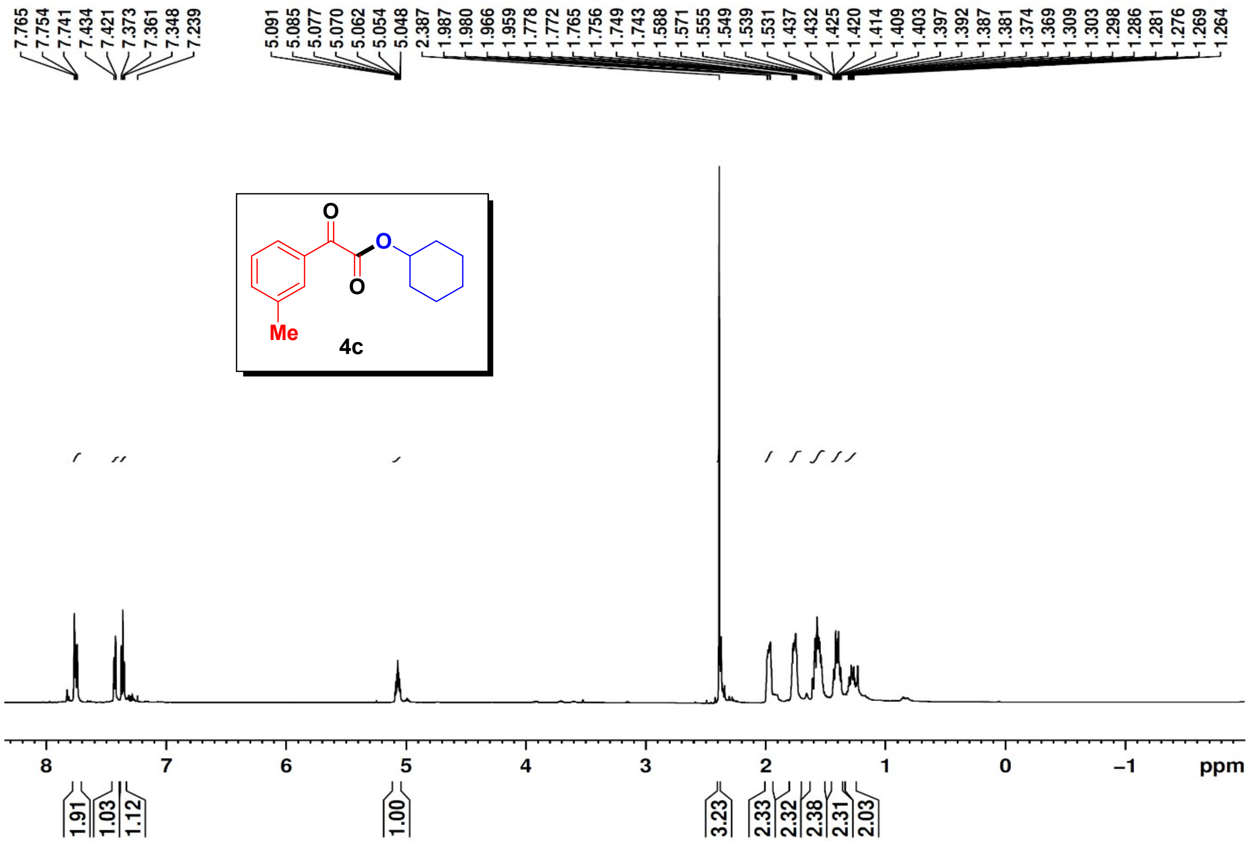

7.765
7.754
7.741
7.434
7.421
7.373
7.361
7.348
7.239
5.091
5.085
5.077
5.070
5.062
5.054
5.048
2.387
1.987
1.980
1.966
1.959
1.778
1.772
1.765
1.756
1.749
1.743
1.588
1.571
1.555
1.549
1.539
1.531
1.437
1.432
1.425
1.420
1.414
1.409
1.403
1.397
1.392
1.387
1.381
1.374
1.369
1.309
1.303
1.298
1.286
1.281
1.276
1.269
1.264
O
O
O
Me
4c
ppm
1.91
1.03
1.12
1.00
3.23
2.33
2.32
2.38
2.31
2.03

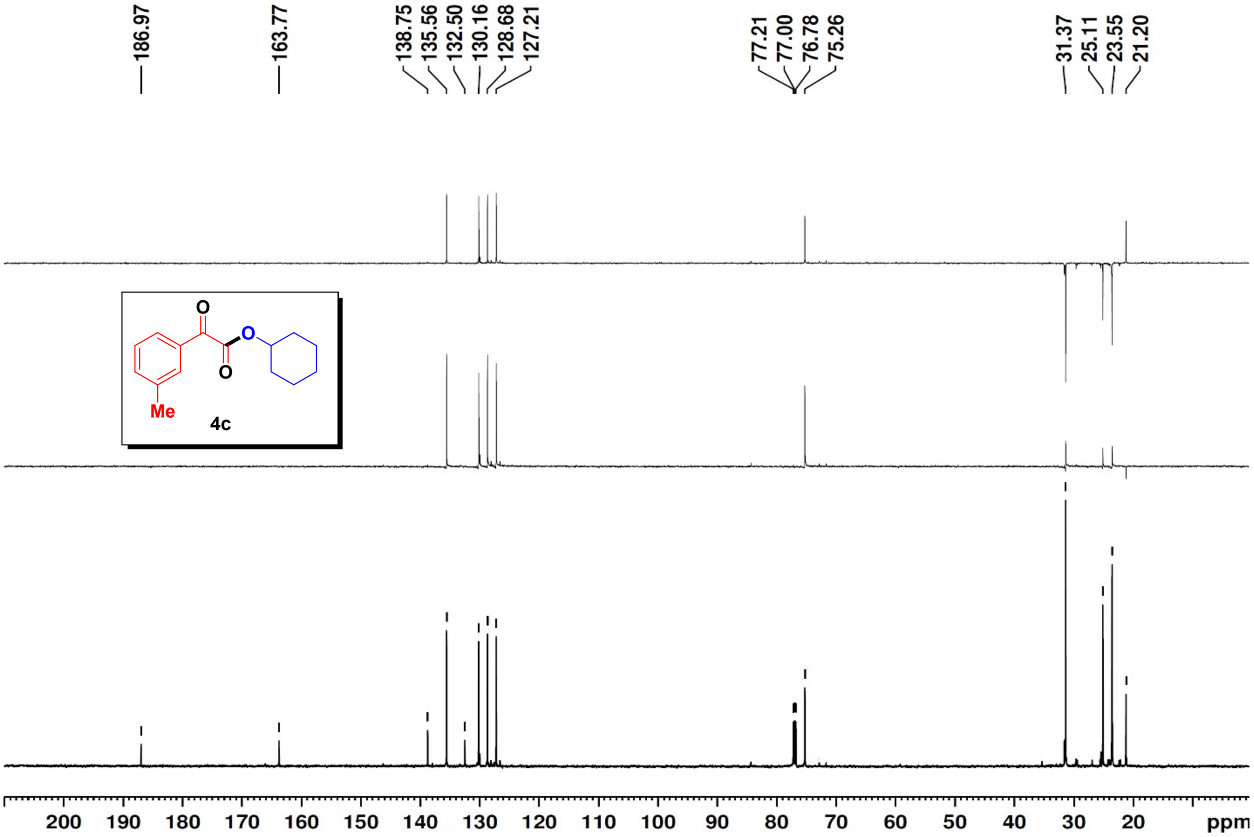

186.97
163.77
138.75
135.56
132.50
130.16
128.68
127.21
77.21
77.00
76.78
75.26
31.37
25.11
23.55
21.20
O
O
O
Me
4c
ppm

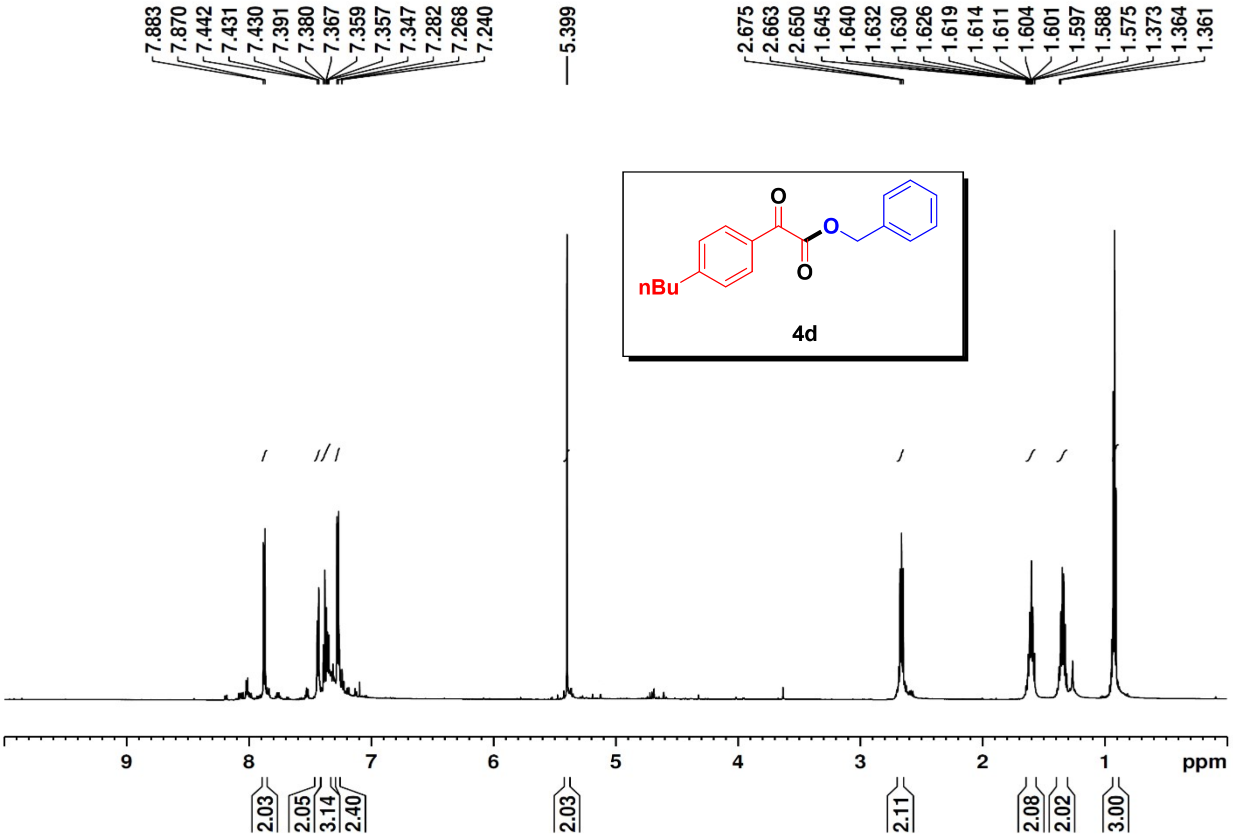
7.883
7.870
7.442
7.431
7.430
7.391
7.380
7.367
7.359
7.357
7.347
7.282
7.268
7.240
5.399
2.675
2.663
2.650
1.645
1.640
1.632
1.630
1.626
1.619
1.614
1.611
1.604
1.601
1.597
1.588
1.575
1.373
1.364
1.361
nBu
4d
9
8
7
6
5
4
3
2
1
ppm
2.03
2.05
3.14
2.40
2.03
2.11
2.08
2.02
3.00

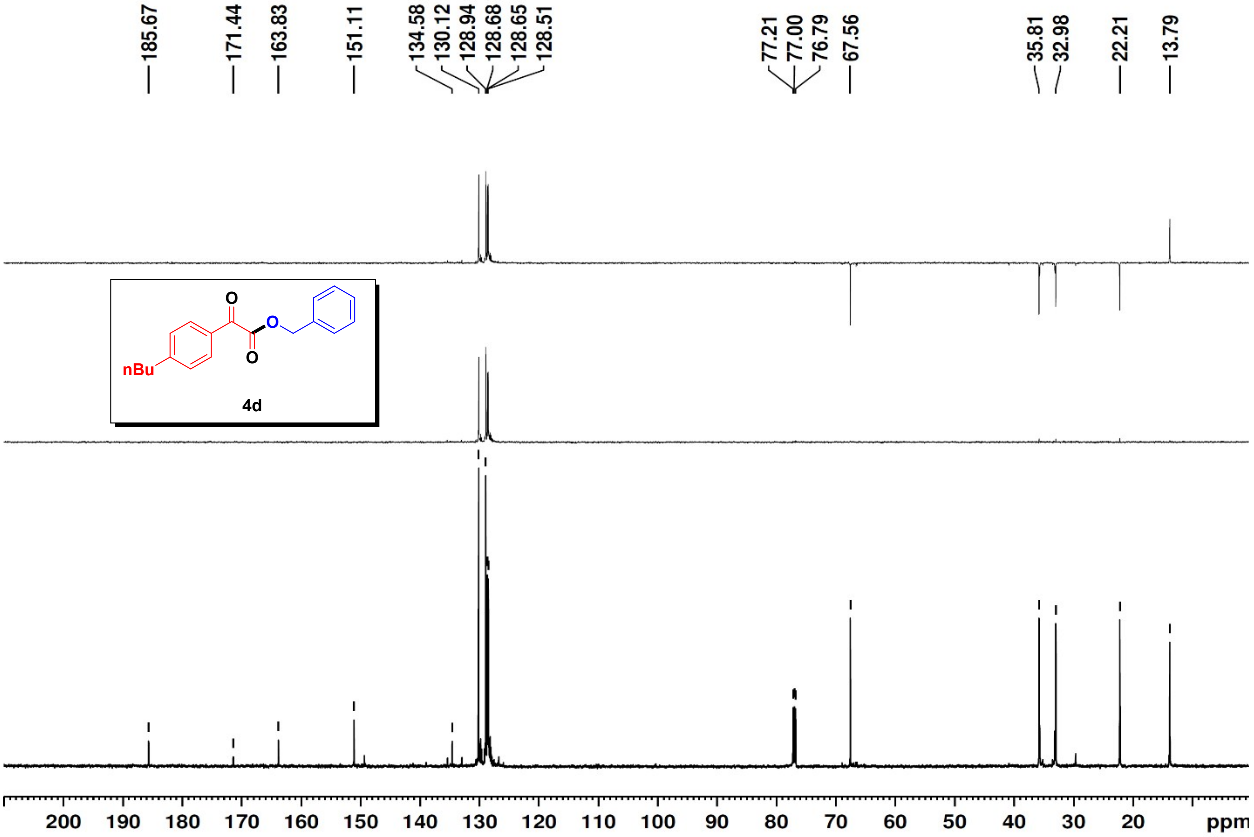
185.67
171.44
163.83
151.11
134.58
130.12
128.94
128.68
128.65
128.51
77.21
77.00
76.79
67.56
35.81
32.98
22.21
13.79
nBu
4d
200
190
180
170
160
150
140
130
120
110
100
90
80
70
60
50
40
30
20
ppm

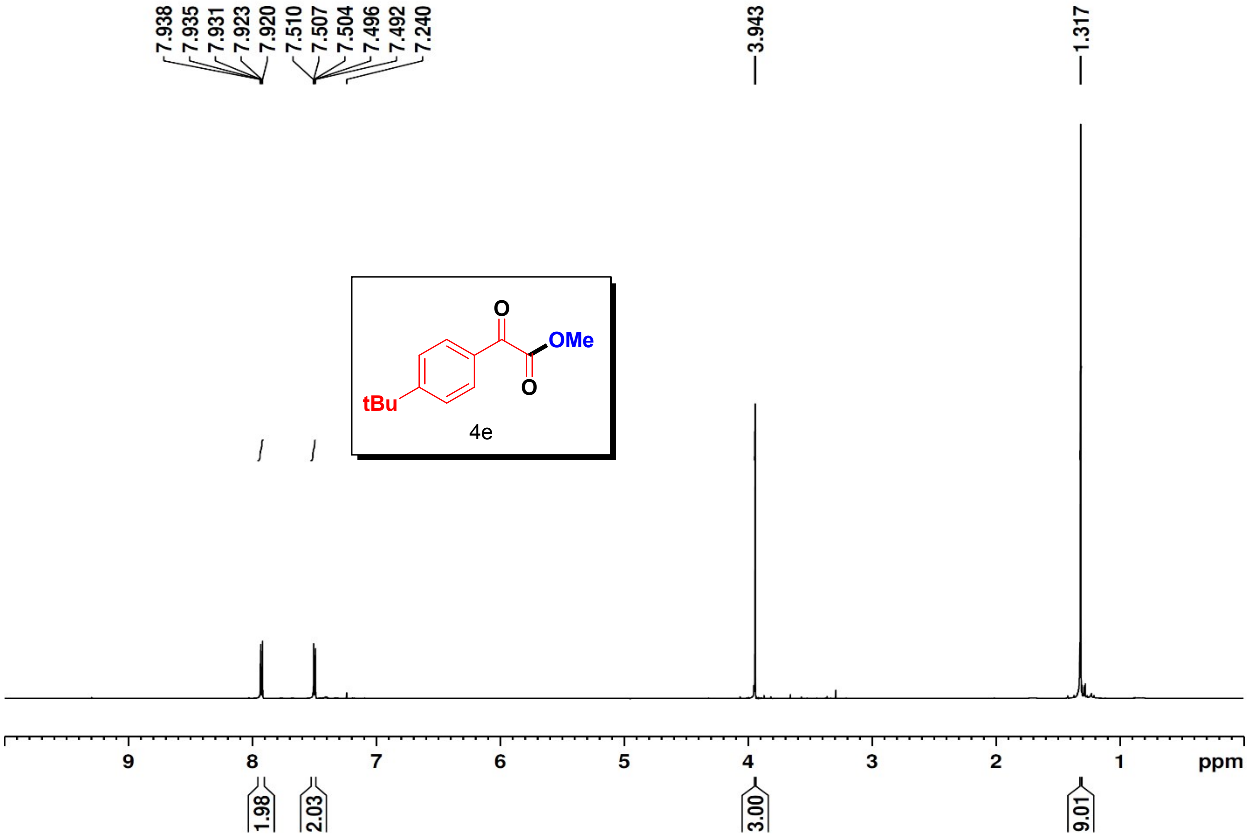
7.938
7.935
7.931
7.923
7.920
7.510
7.507
7.504
7.496
7.492
7.240
3.943
1.317
tBu
O
OMe
O
4e
9
8
7
6
5
4
3
2
1
ppm
1.98
2.03
3.00
9.01

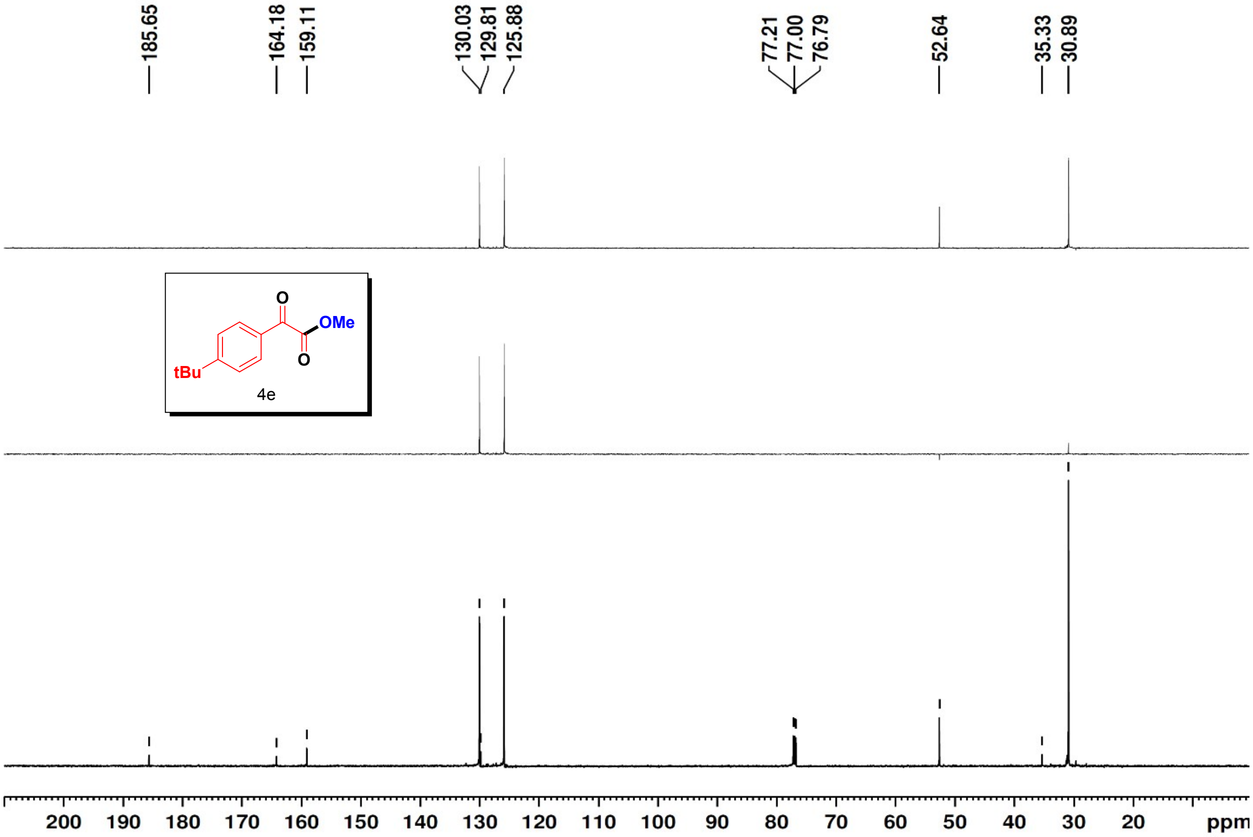
185.65
164.18
159.11
130.03
129.81
125.88
77.21
77.00
76.79
52.64
35.33
30.89
tBu
O
OMe
O
4e
200
190
180
170
160
150
140
130
120
110
100
90
80
70
60
50
40
30
20
ppm

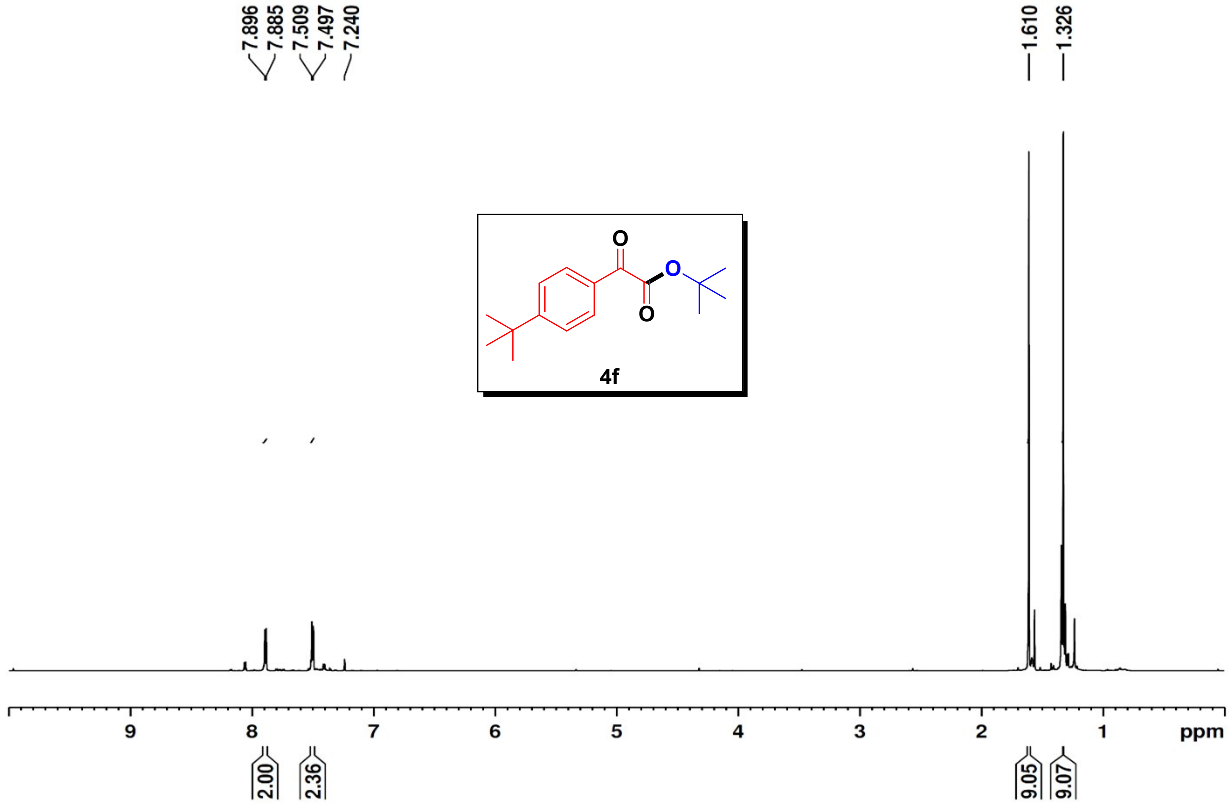
7.896
7.885
7.509
7.497
7.240
1.610
1.326
4f
9 8 7 6 5 4 3 2 1 ppm
2.00
2.36
9.05
9.07

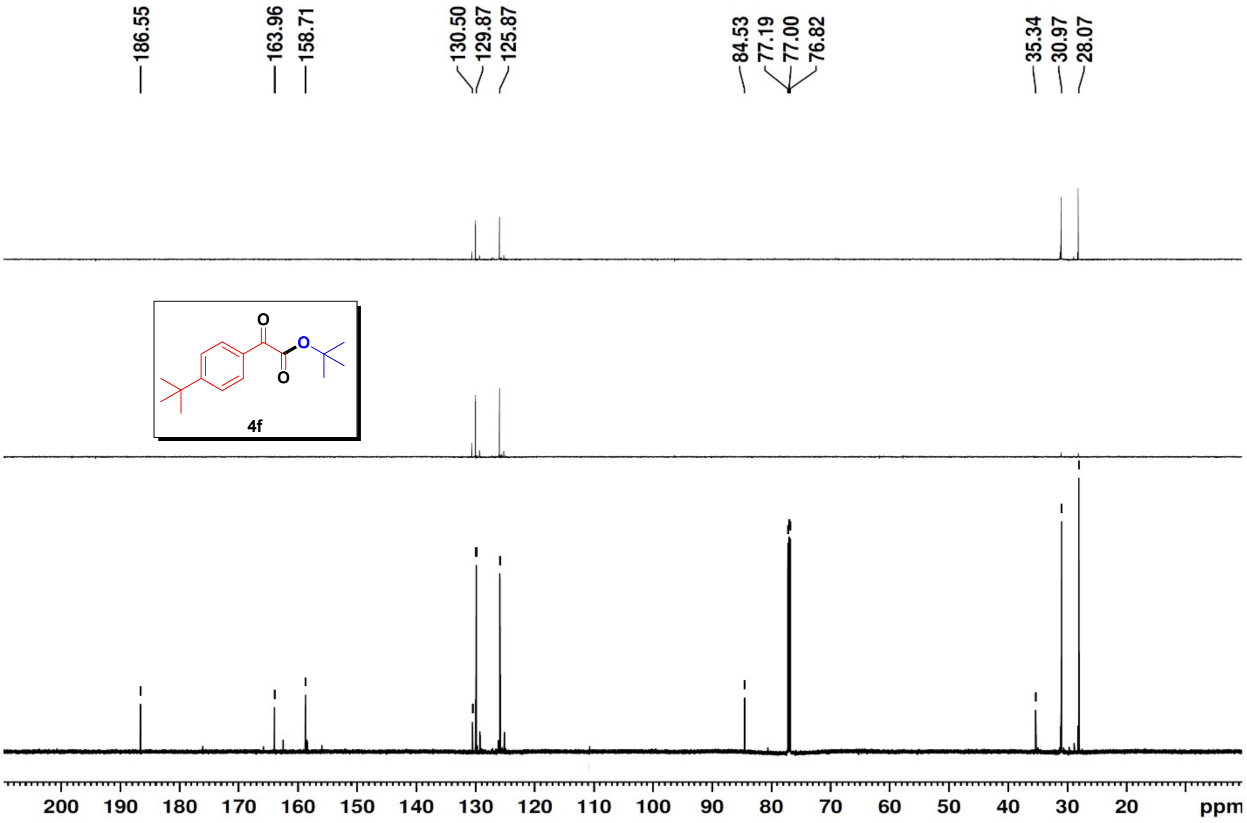
186.55
163.96
158.71
130.50
129.87
125.87
84.53
77.19
77.00
76.82
35.34
30.97
28.07
4f
200 190 180 170 160 150 140 130 120 110 100 90 80 70 60 50 40 30 20 ppm

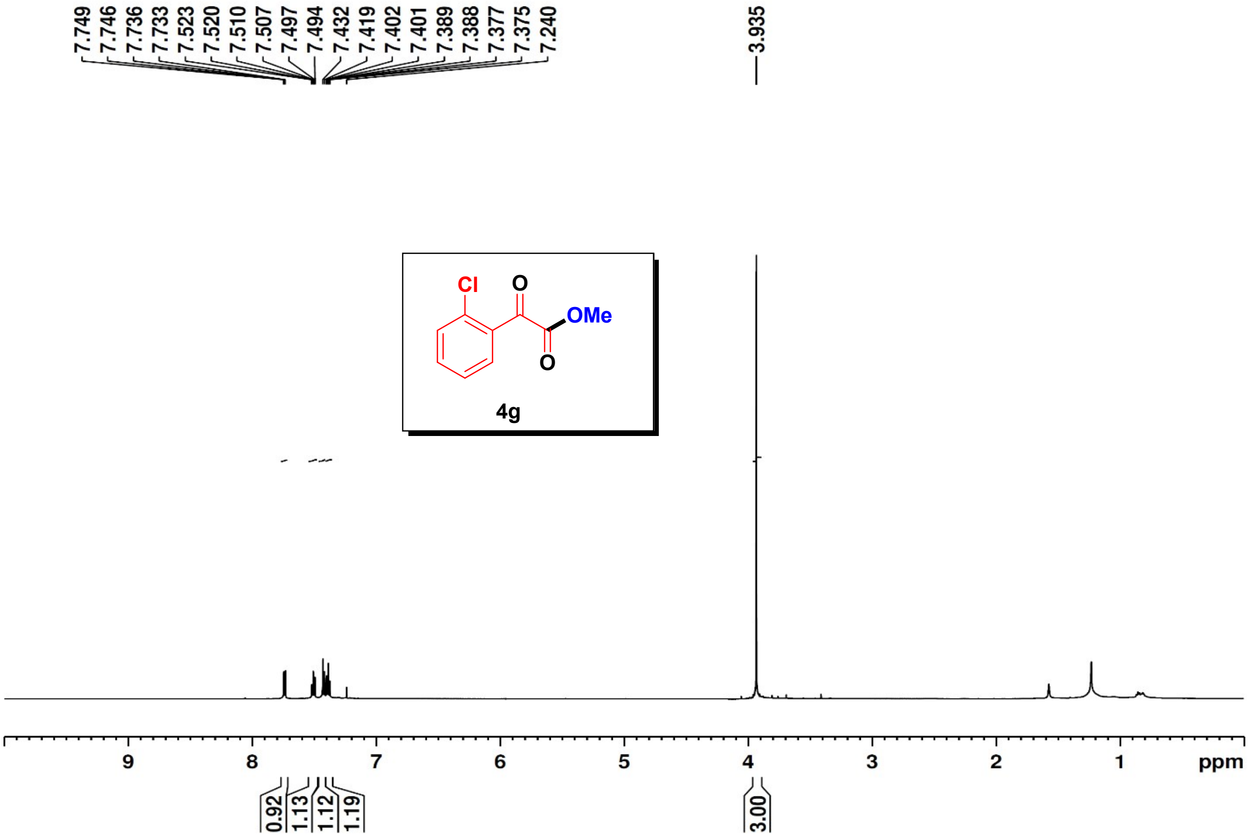
7.749
7.746
7.736
7.733
7.523
7.520
7.510
7.507
7.497
7.494
7.432
7.419
7.402
7.401
7.389
7.388
7.377
7.375
7.240
3.935
Cl
O
OMe
O
4g
9
8
7
6
5
4
3
2
1
ppm
0.92
1.13
1.12
1.19
3.00

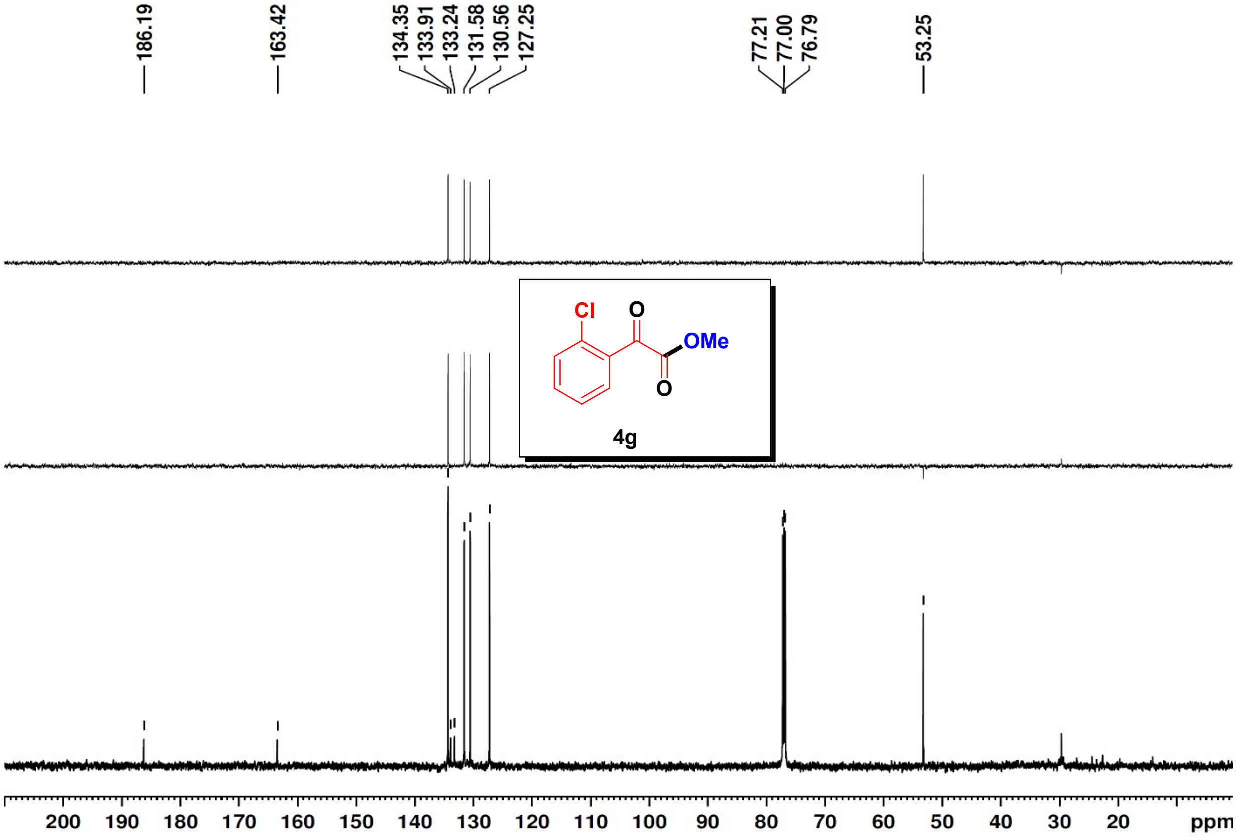
186.19
163.42
134.35
133.91
133.24
131.58
130.56
127.25
77.21
77.00
76.79
53.25
Cl
O
OMe
O
4g
200
190
180
170
160
150
140
130
120
110
100
90
80
70
60
50
40
30
20
ppm

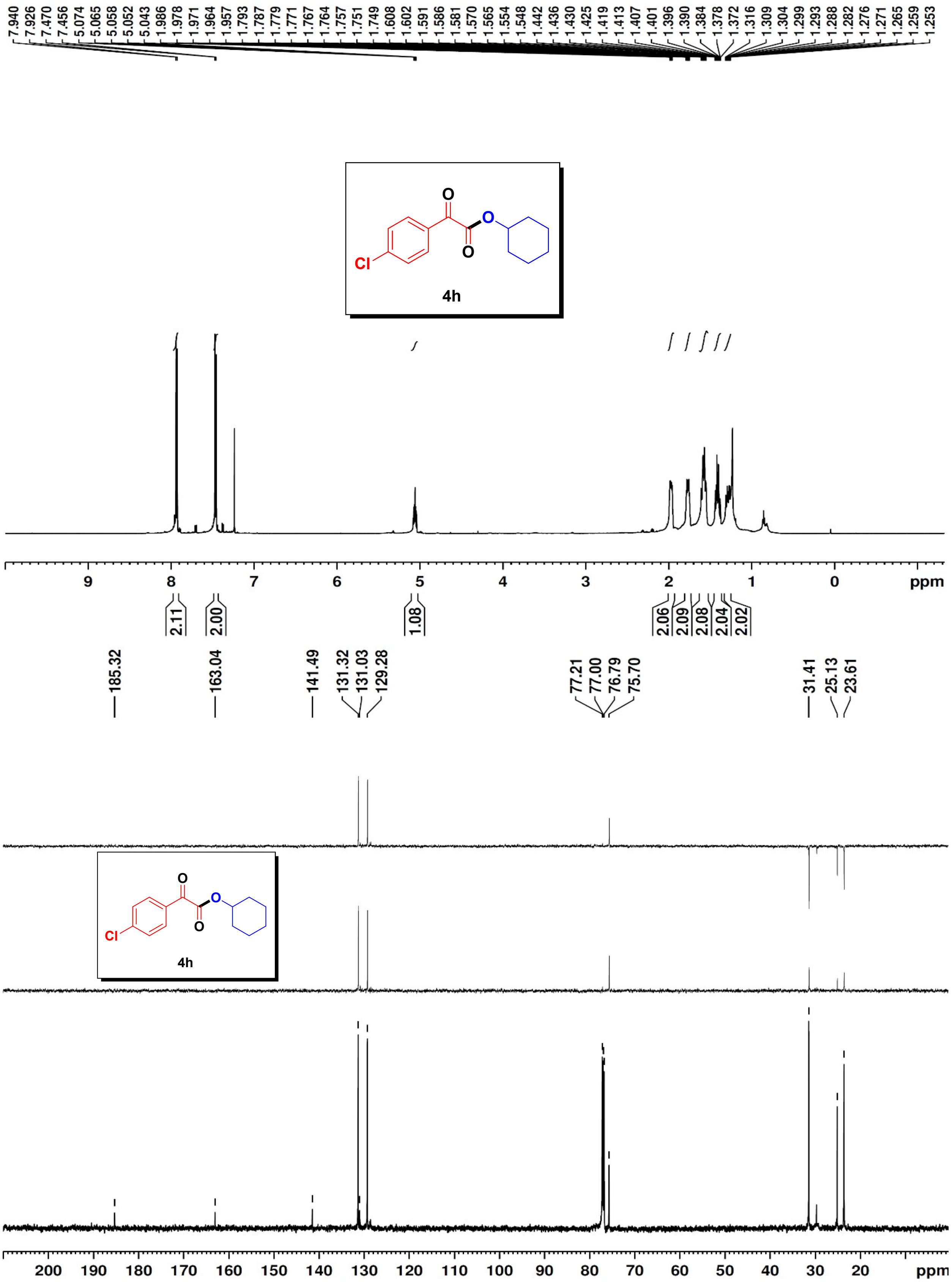

7.940
7.926
7.470
7.456
5.074
5.065
5.058
5.052
5.043
1.986
1.978
1.971
1.964
1.957
1.793
1.787
1.779
1.771
1.767
1.764
1.757
1.751
1.749
1.608
1.602
1.591
1.586
1.581
1.570
1.565
1.554
1.548
1.442
1.436
1.430
1.425
1.419
1.413
1.407
1.401
1.396
1.390
1.384
1.378
1.372
1.316
1.309
1.304
1.299
1.293
1.288
1.282
1.276
1.271
1.265
1.259
1.253
O
O
O
Cl
4h
2.11
2.00
1.08
2.06
2.09
2.08
2.04
2.02
ppm
185.32
163.04
141.49
131.32
131.03
129.28
77.21
77.00
76.79
75.70
31.41
25.13
23.61
200
190
180
170
160
150
140
130
120
110
100
90
80
70
60
50
40
30
20
ppm

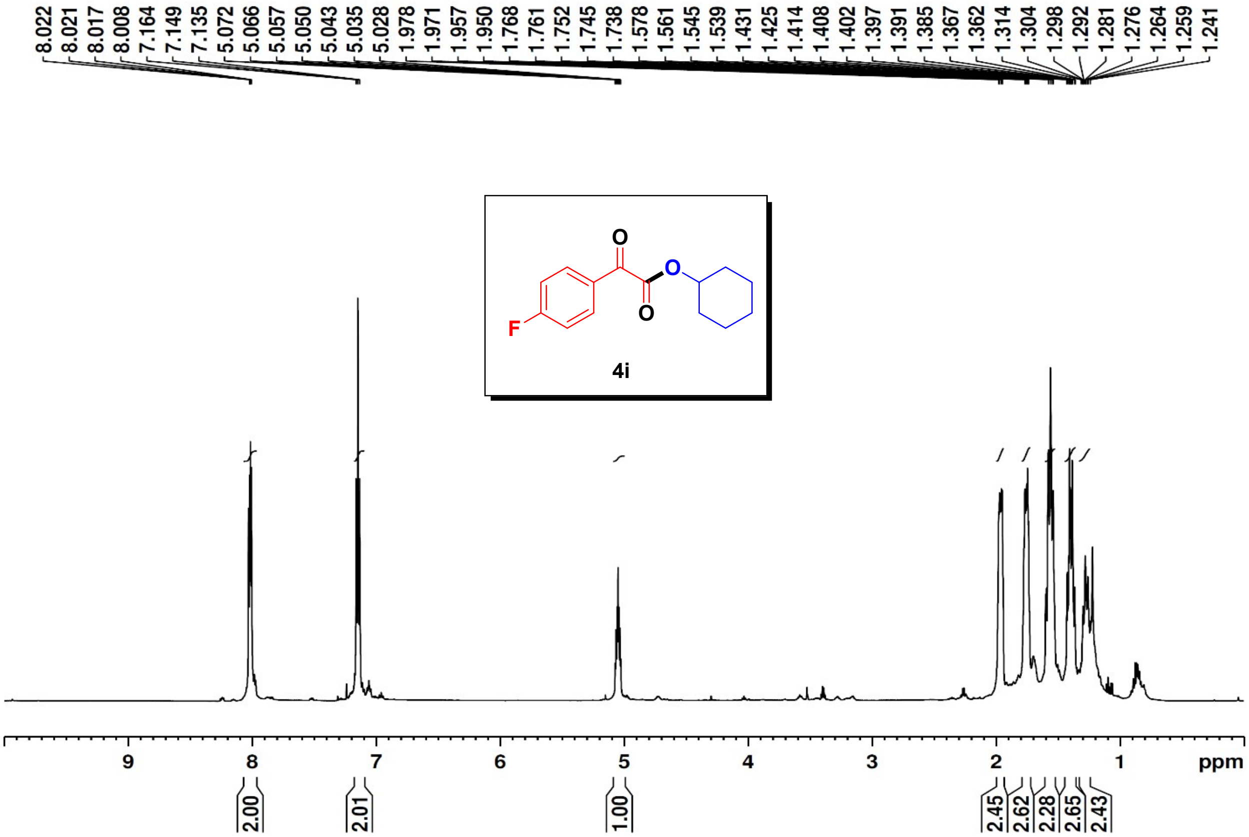
8.022
8.021
8.017
8.008
7.164
7.149
7.135
5.072
5.066
5.057
5.050
5.043
5.035
5.028
1.978
1.971
1.957
1.950
1.768
1.761
1.752
1.745
1.738
1.578
1.561
1.545
1.539
1.431
1.425
1.414
1.408
1.402
1.397
1.391
1.385
1.367
1.362
1.314
1.304
1.298
1.292
1.281
1.276
1.264
1.259
1.241
O
O
O
F
4i
9
8
7
6
5
4
3
2
1
ppm
2.00
2.01
1.00
2.45
2.62
2.28
2.65
2.43

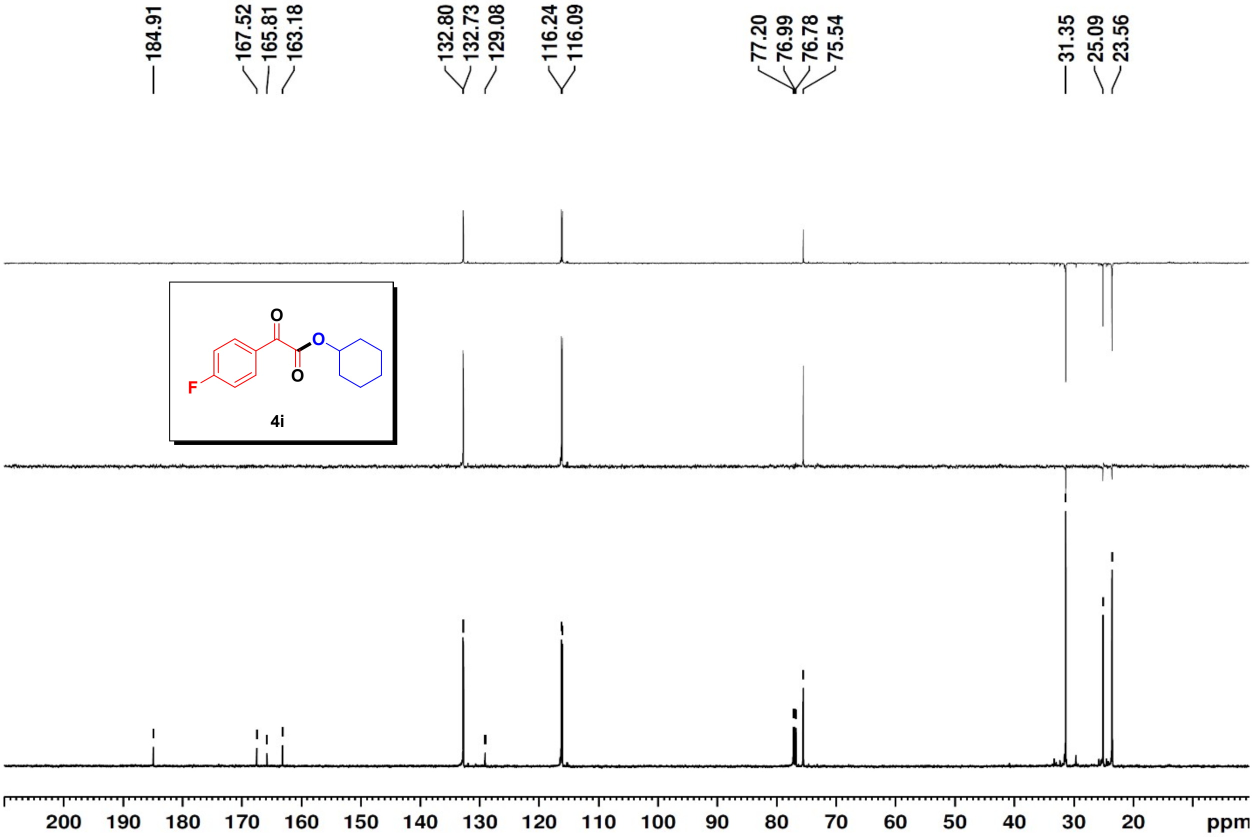
184.91
167.52
165.81
163.18
132.80
132.73
129.08
116.24
116.09
77.20
76.99
76.78
75.54
31.35
25.09
23.56
O
O
O
F
4i
200
190
180
170
160
150
140
130
120
110
100
90
80
70
60
50
40
30
20
ppm

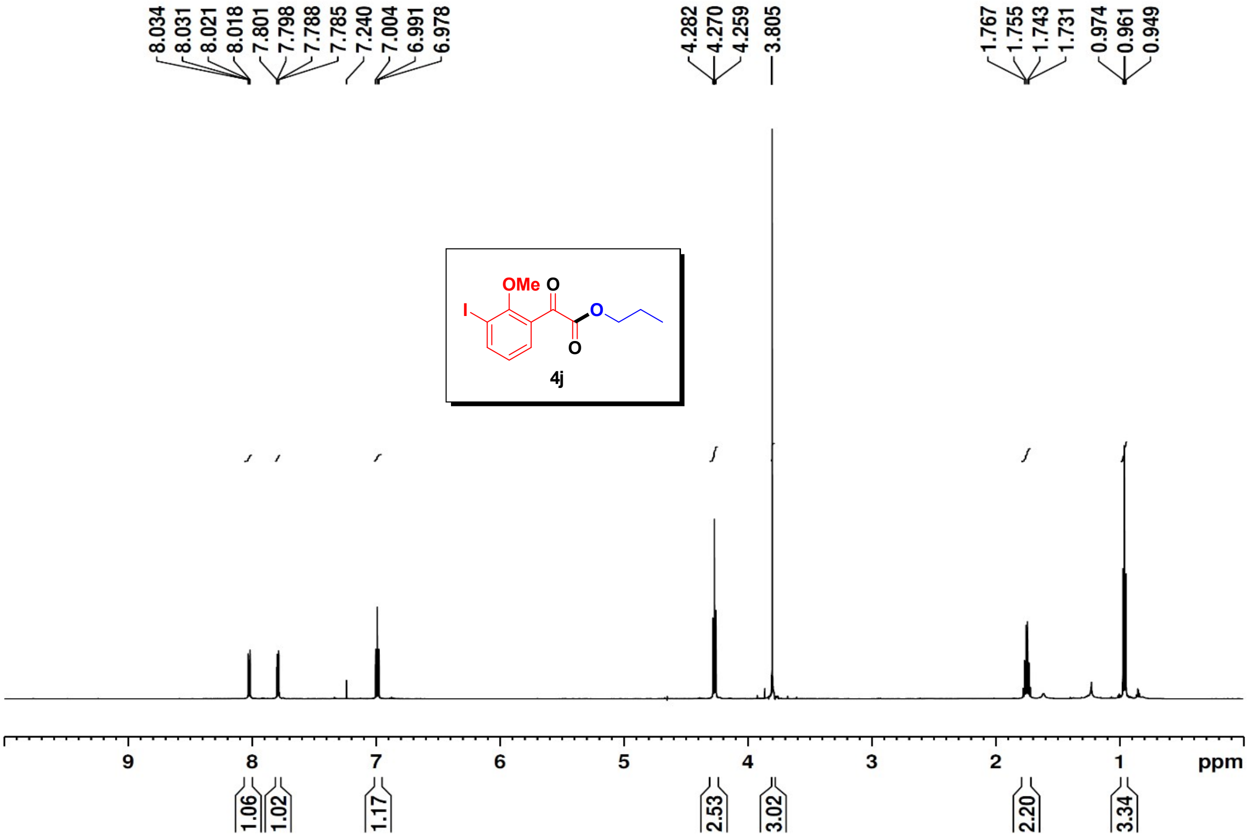

8.034
8.031
8.021
8.018
7.801
7.798
7.788
7.785
7.240
7.004
6.991
6.978
4.282
4.270
4.259
3.805
1.767
1.755
1.743
1.731
0.974
0.961
0.949
OMe
O
I
O
O
4j
9
8
7
6
5
4
3
2
1
ppm
1.06
1.02
1.17
2.53
3.02
2.20
3.34

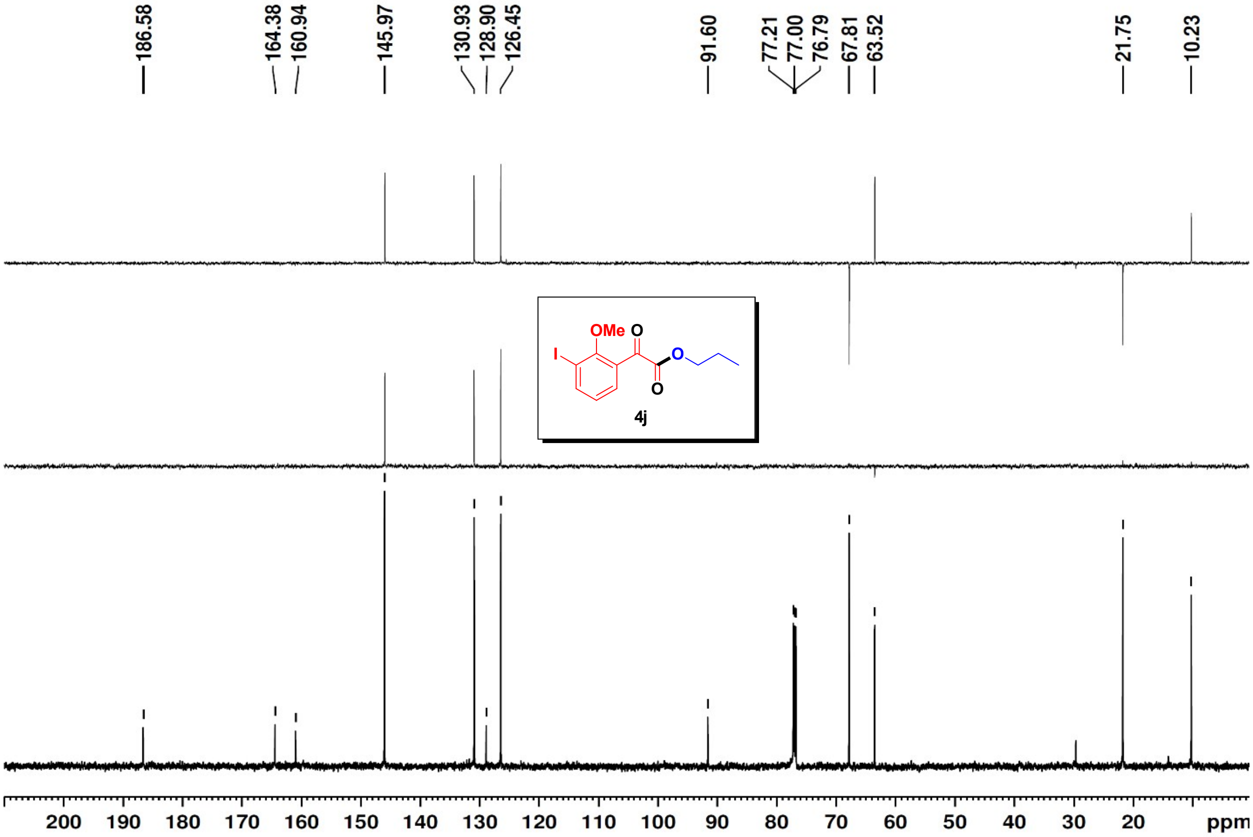

186.58
164.38
160.94
145.97
130.93
128.90
126.45
91.60
77.21
77.00
76.79
67.81
63.52
21.75
10.23
OMe
O
I
O
O
4j
200
190
180
170
160
150
140
130
120
110
100
90
80
70
60
50
40
30
20
ppm

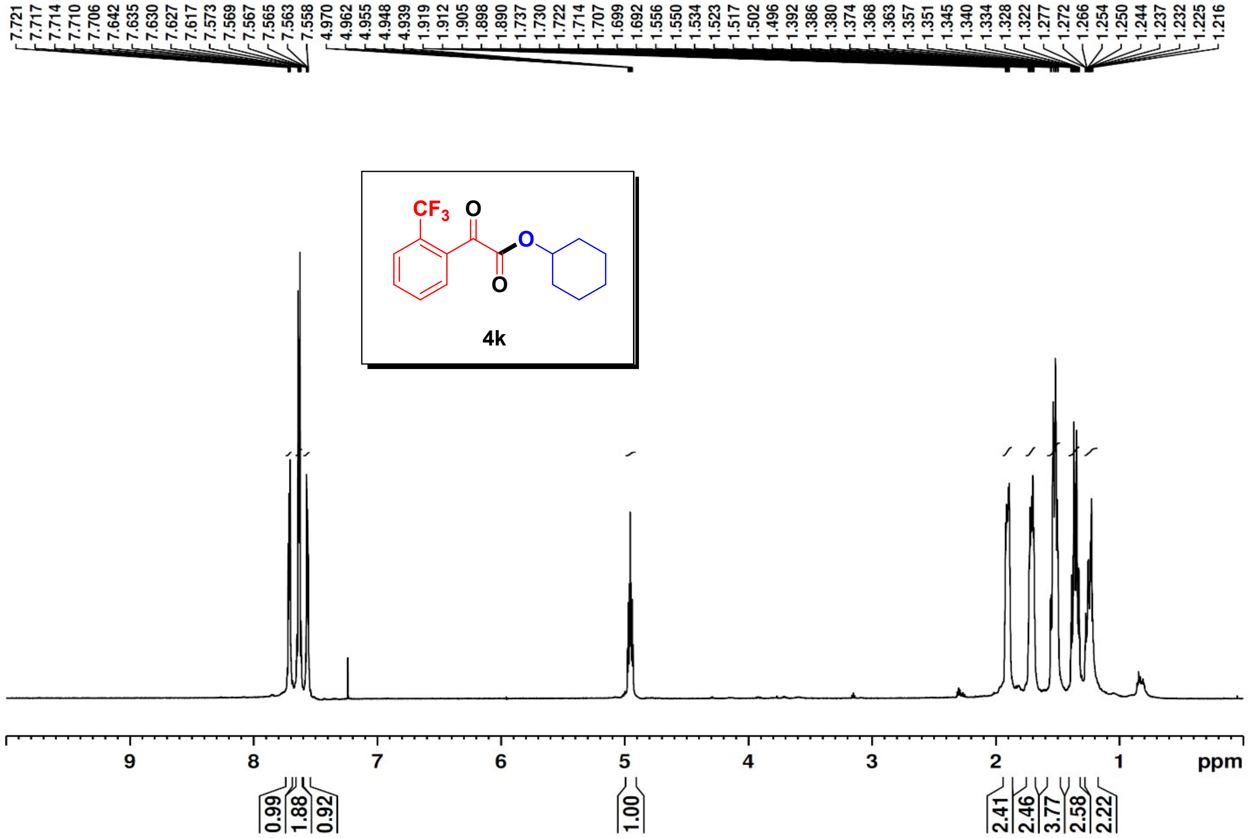
7.721
7.717
7.714
7.710
7.706
7.642
7.635
7.630
7.627
7.617
7.573
7.569
7.567
7.565
7.563
7.558
4.970
4.962
4.955
4.948
4.939
1.919
1.912
1.905
1.898
1.890
1.737
1.730
1.722
1.714
1.707
1.699
1.692
1.556
1.550
1.534
1.523
1.517
1.502
1.496
1.392
1.386
1.380
1.374
1.368
1.363
1.357
1.351
1.345
1.340
1.334
1.328
1.322
1.277
1.272
1.266
1.254
1.250
1.244
1.237
1.232
1.225
1.216
CF3
O
O
O
4k
9
8
7
6
5
4
3
2
1
ppm
0.99
1.88
0.92
1.00
2.41
2.46
3.77
2.58
2.22

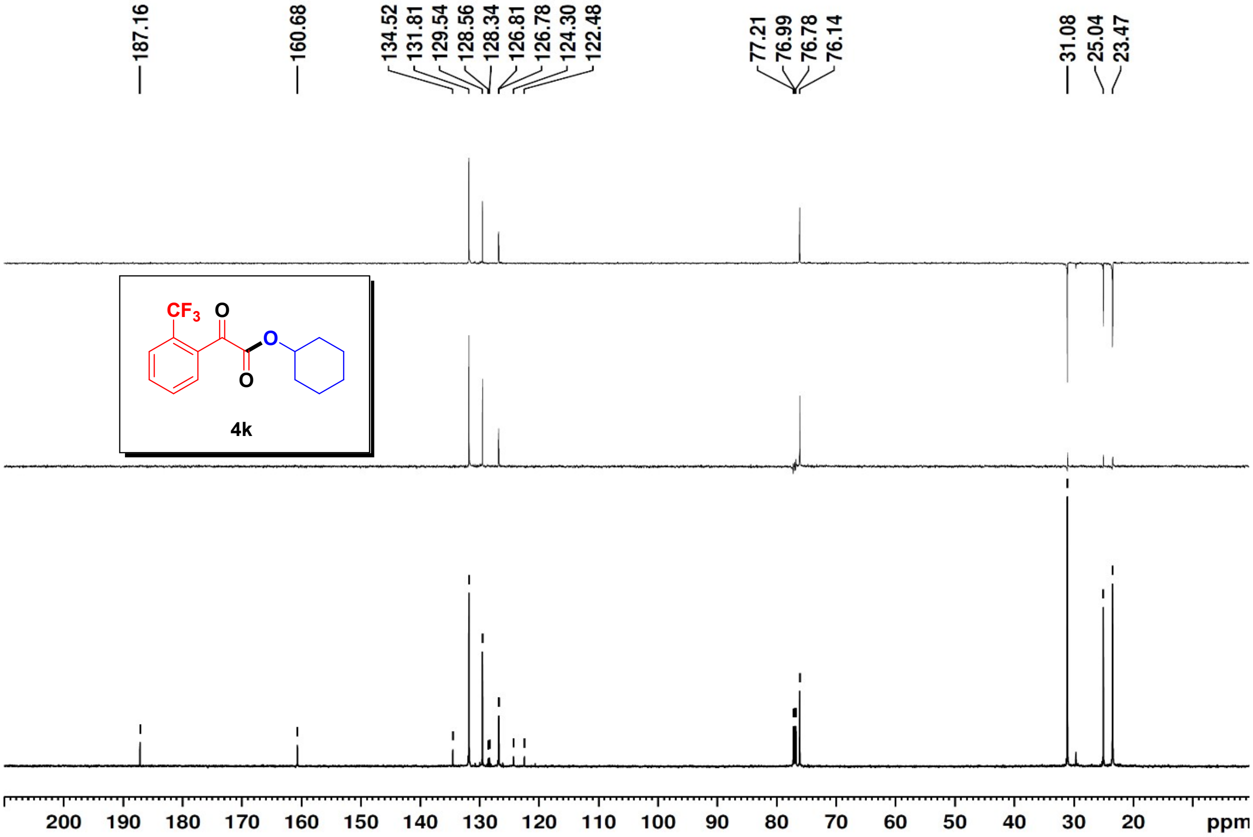
187.16
160.68
134.52
131.81
129.54
128.56
128.34
126.81
126.78
124.30
122.48
77.21
76.99
76.78
76.14
31.08
25.04
23.47
CF3
O
O
O
4k
200
190
180
170
160
150
140
130
120
110
100
90
80
70
60
50
40
30
20
ppm

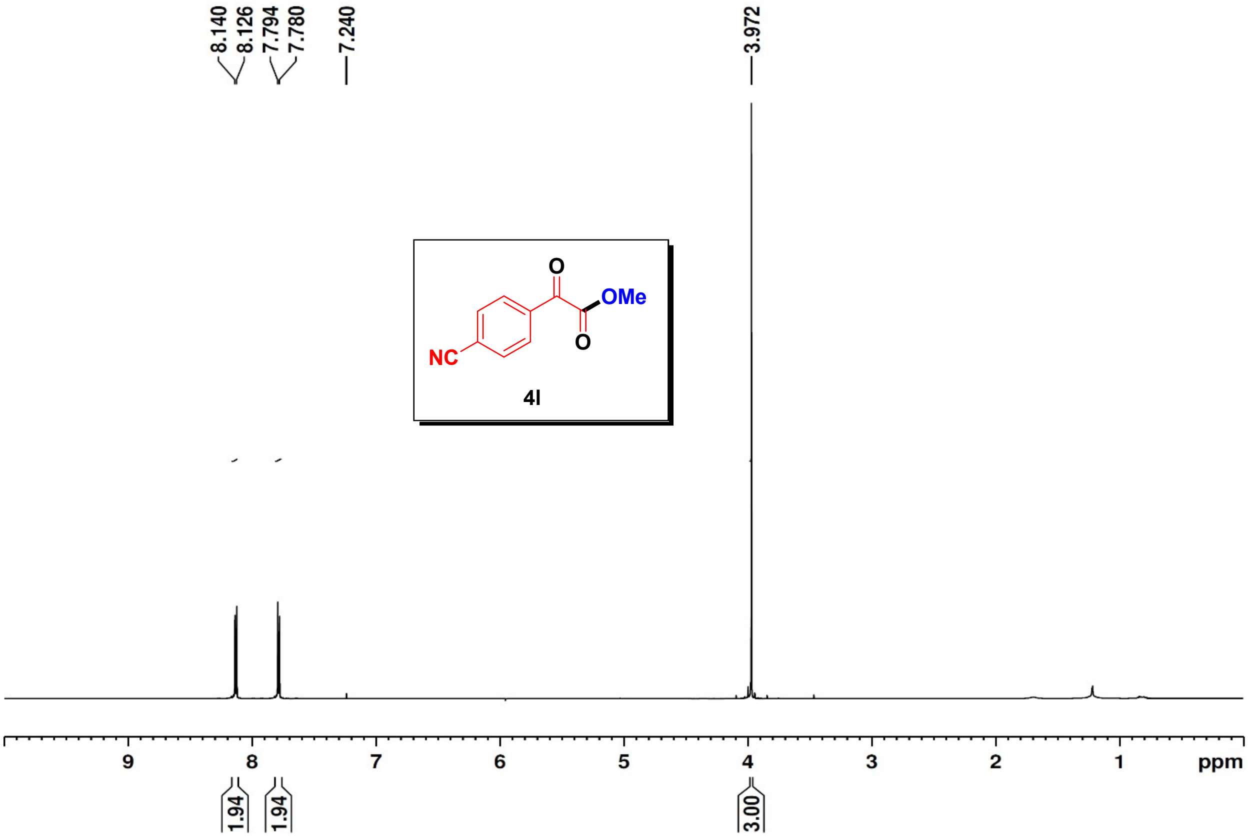
8.140
8.126
7.794
7.780
7.240
3.972
O
OMe
NC
O
4l
9
8
7
6
5
4
3
2
1
ppm
1.94
1.94
3.00

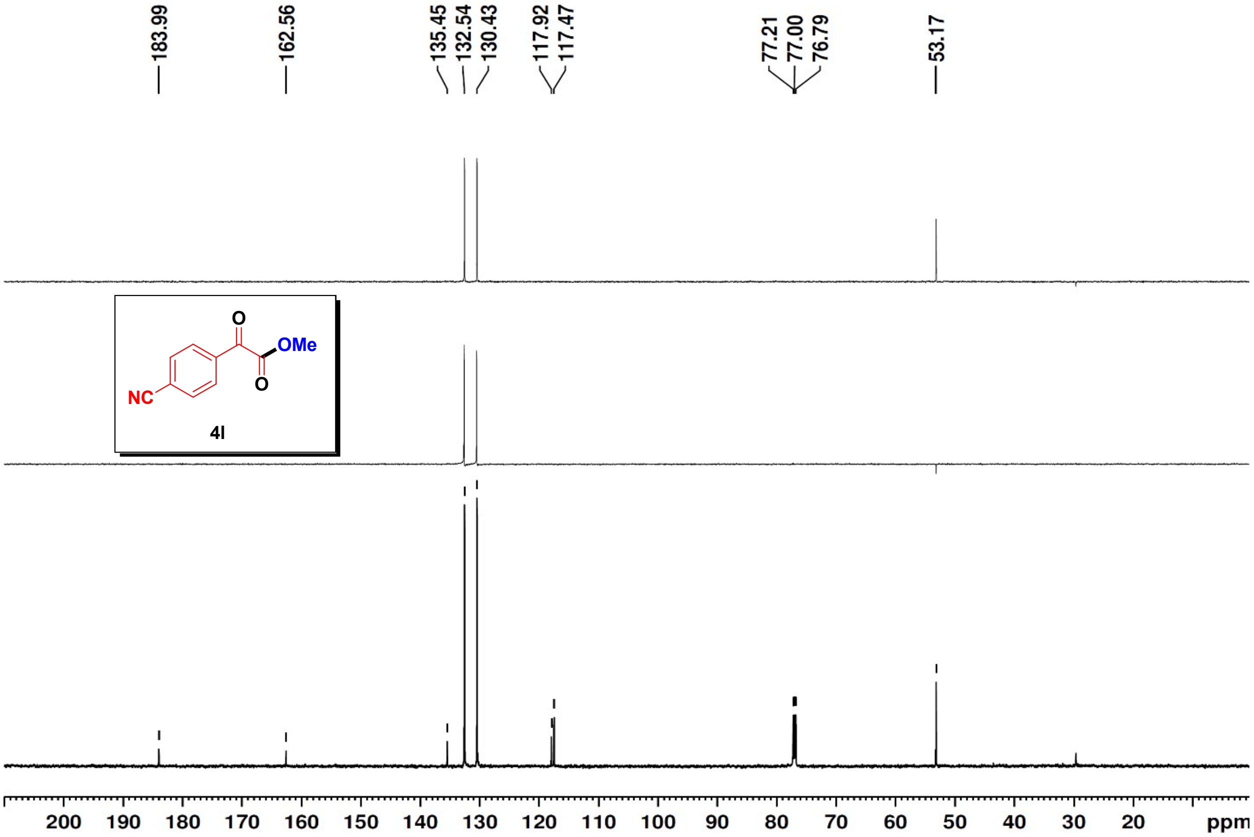
183.99
162.56
135.45
132.54
130.43
117.92
117.47
77.21
77.00
76.79
53.17
O
OMe
NC
O
4l
200
190
180
170
160
150
140
130
120
110
100
90
80
70
60
50
40
30
20
ppm

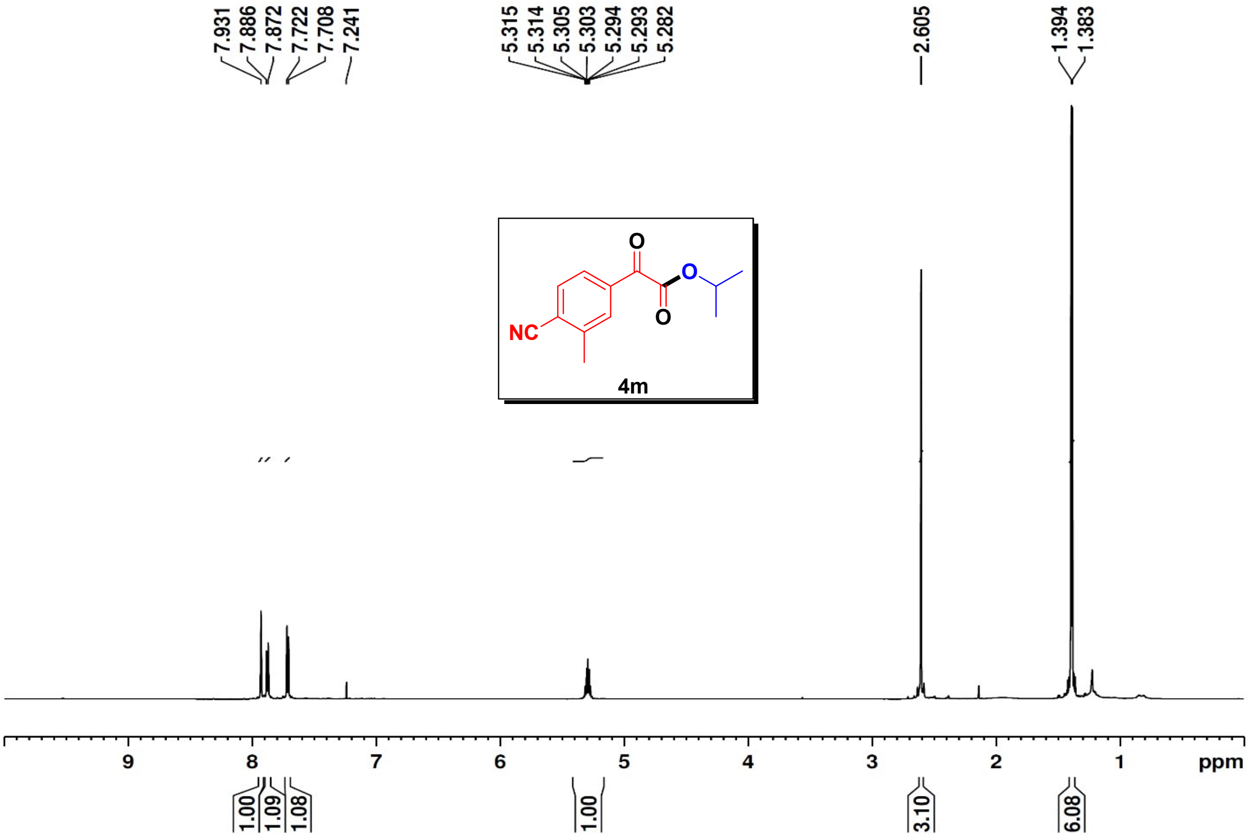

7.931
7.886
7.872
7.722
7.708
7.241
5.315
5.314
5.305
5.303
5.294
5.293
5.282
2.605
1.394
1.383
NC
O
O
O
4m
9
8
7
6
5
4
3
2
1
ppm
1.00
1.09
1.08
1.00
3.10
6.08

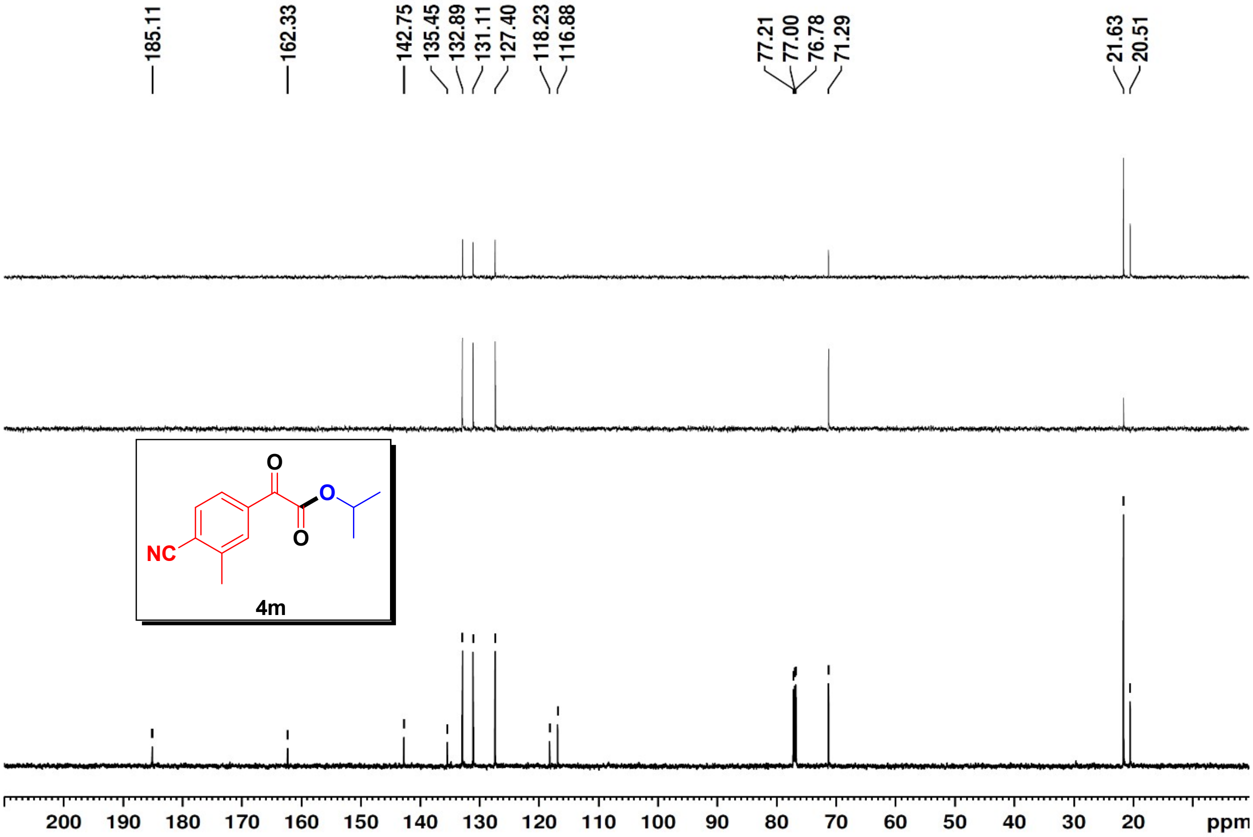

185.11
162.33
142.75
135.45
132.89
131.11
127.40
118.23
116.88
77.21
77.00
76.78
71.29
21.63
20.51
NC
O
O
O
4m
200
190
180
170
160
150
140
130
120
110
100
90
80
70
60
50
40
30
20
ppm

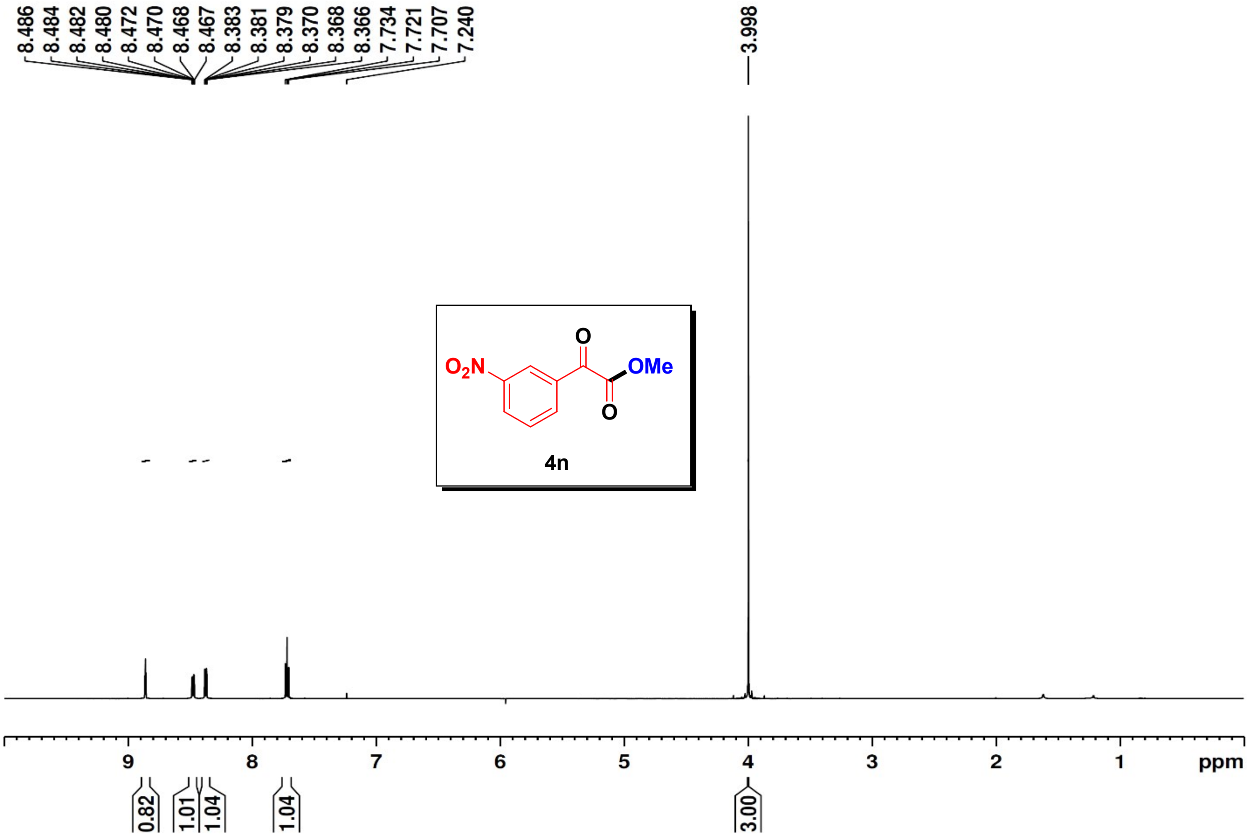

8.486
8.484
8.482
8.480
8.472
8.470
8.468
8.467
8.383
8.381
8.379
8.370
8.368
8.366
7.734
7.721
7.707
7.240
3.998
O
O_2N
OMe
O
4n
9
8
7
6
5
4
3
2
1
ppm
0.82
1.01
1.04
1.04
3.00

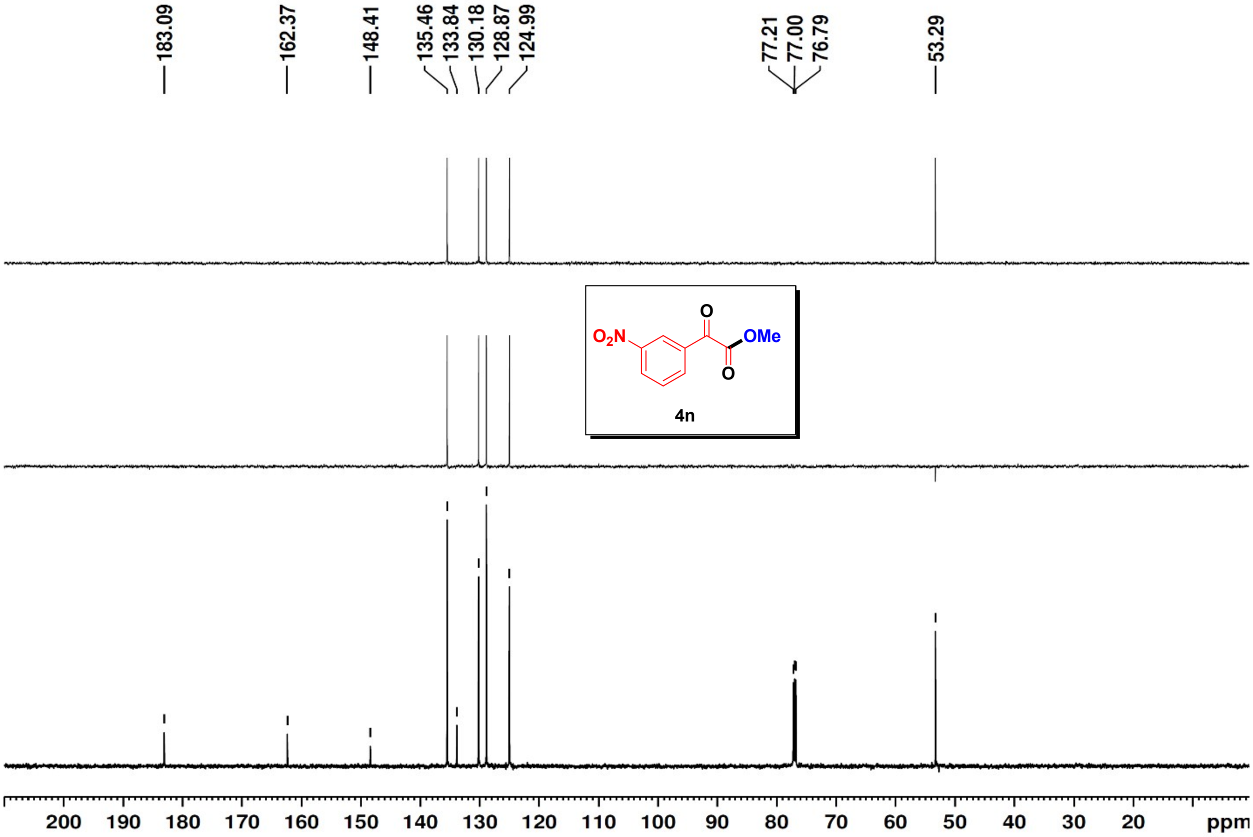

183.09
162.37
148.41
135.46
133.84
130.18
128.87
124.99
77.21
77.00
76.79
53.29
O
O_2N
OMe
O
4n
200
190
180
170
160
150
140
130
120
110
100
90
80
70
60
50
40
30
20
ppm

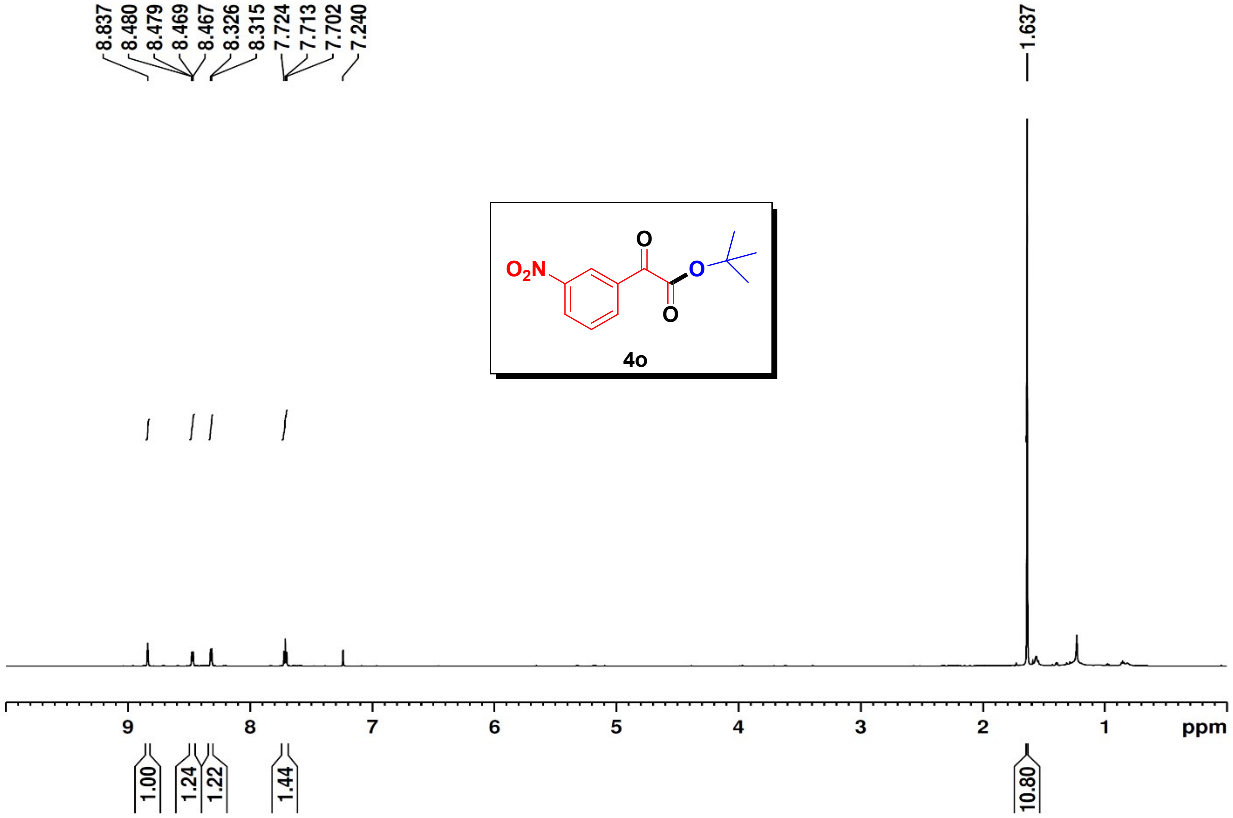
8.837
8.480
8.479
8.469
8.467
8.326
8.315
7.724
7.713
7.702
7.240
1.637
O_2N
O
O
O
4o
1.00
1.24
1.22
1.44
10.80
9
8
7
6
5
4
3
2
1
ppm

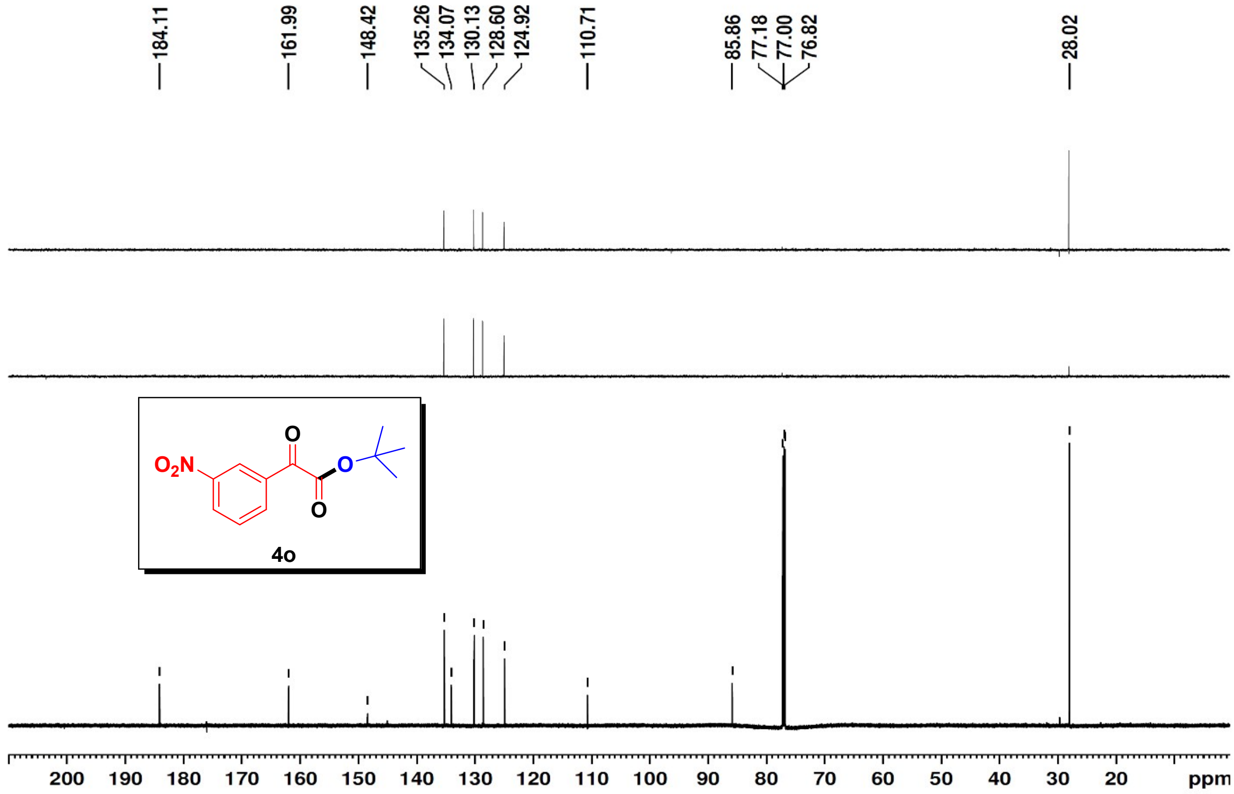
184.11
161.99
148.42
135.26
134.07
130.13
128.60
124.92
110.71
85.86
77.18
77.00
76.82
28.02
O_2N
O
O
O
4o
200
190
180
170
160
150
140
130
120
110
100
90
80
70
60
50
40
30
20
ppm

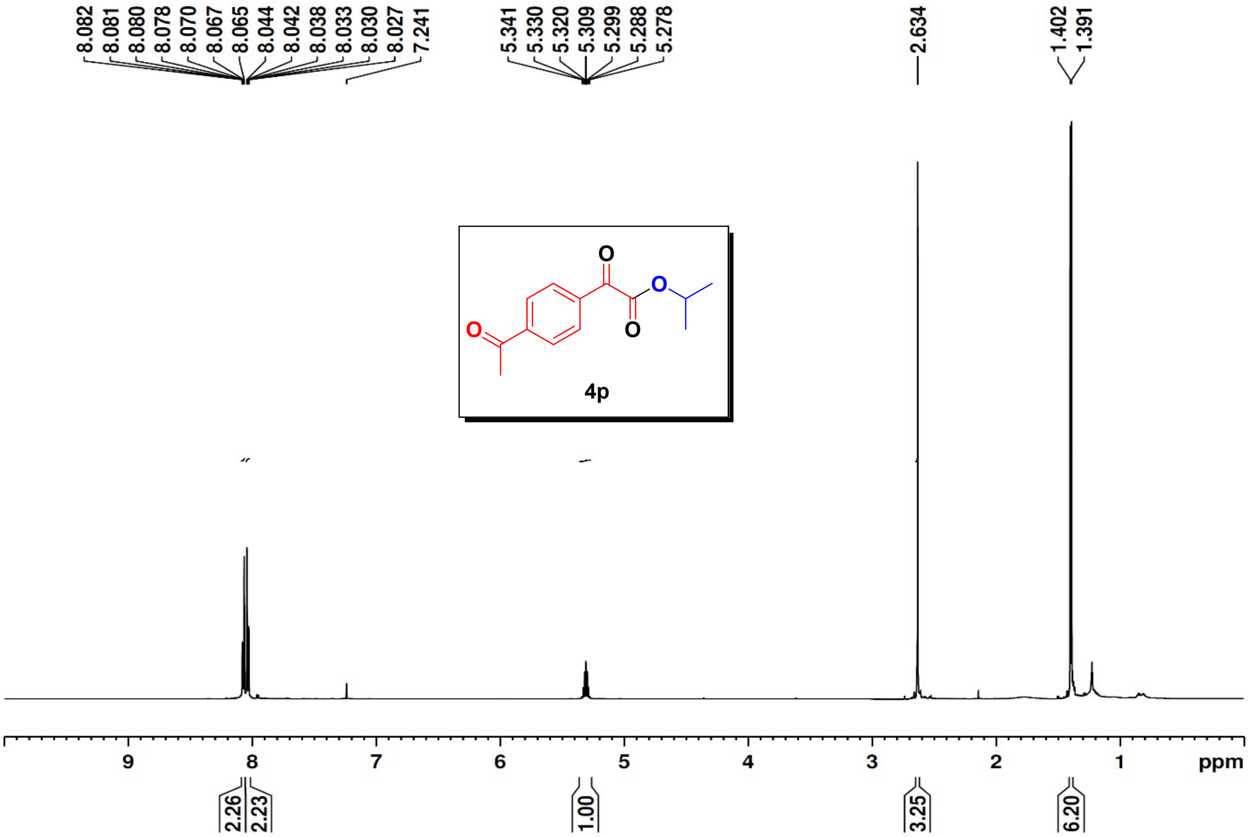
8.082
8.081
8.080
8.078
8.070
8.067
8.065
8.044
8.042
8.038
8.033
8.030
8.027
7.241
5.341
5.330
5.320
5.309
5.299
5.288
5.278
2.634
1.402
1.391
O
O
O
O
4p
9
8
7
6
5
4
3
2
1
ppm
2.26
2.23
1.00
3.25
6.20

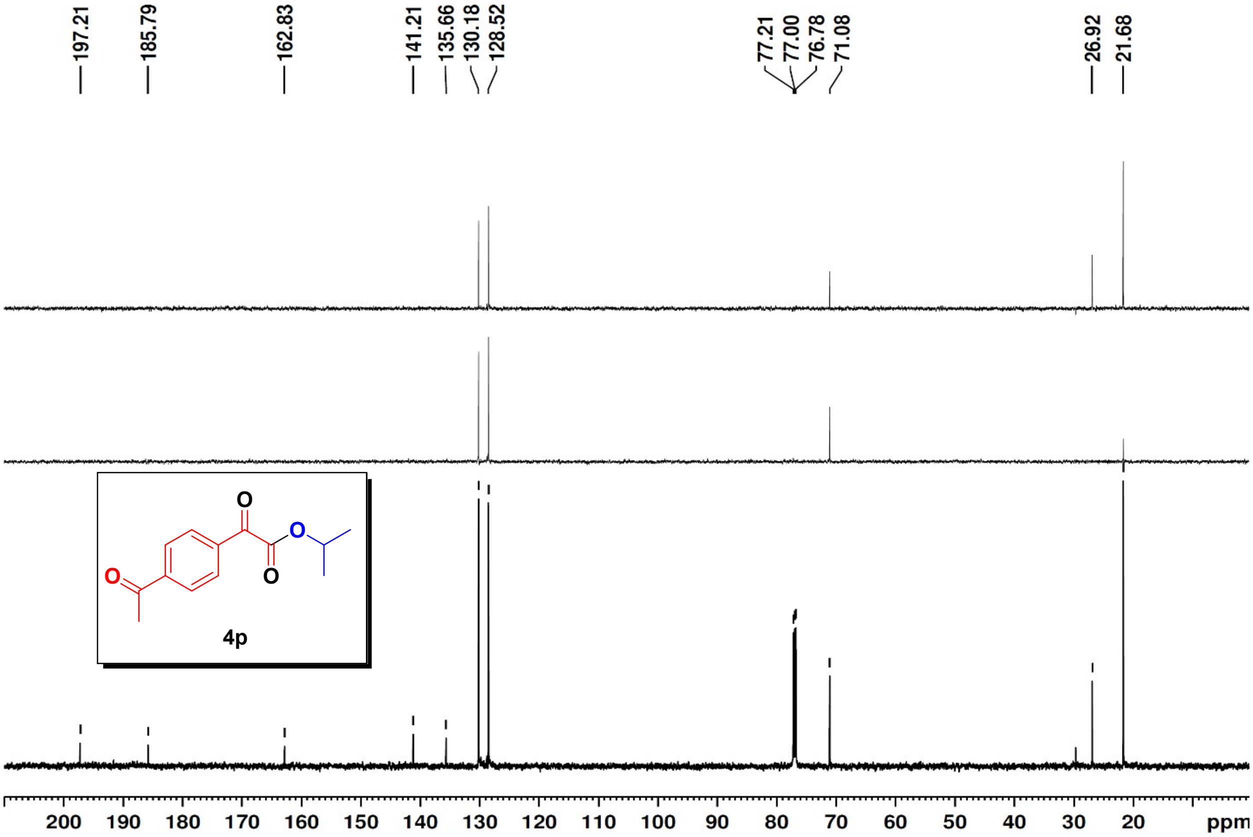
197.21
185.79
162.83
141.21
135.66
130.18
128.52
77.21
77.00
76.78
71.08
26.92
21.68
O
O
O
O
4p
200
190
180
170
160
150
140
130
120
110
100
90
80
70
60
50
40
30
20
ppm

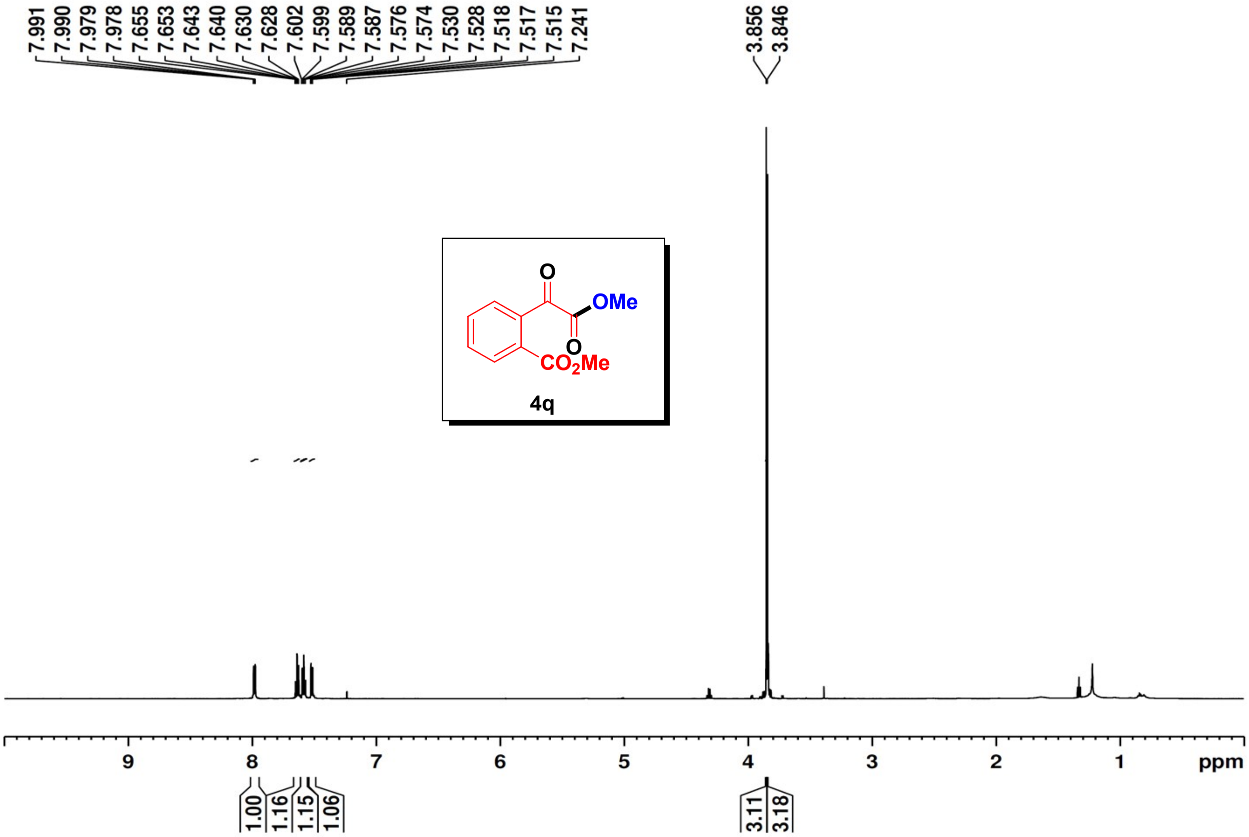
7.991
7.990
7.979
7.978
7.655
7.653
7.643
7.640
7.630
7.628
7.602
7.599
7.589
7.587
7.576
7.574
7.530
7.528
7.518
7.517
7.515
7.241
3.856
3.846
OMe
CO2Me
4q
9
8
7
6
5
4
3
2
1
ppm
1.00
1.16
1.15
1.06
3.11
3.18

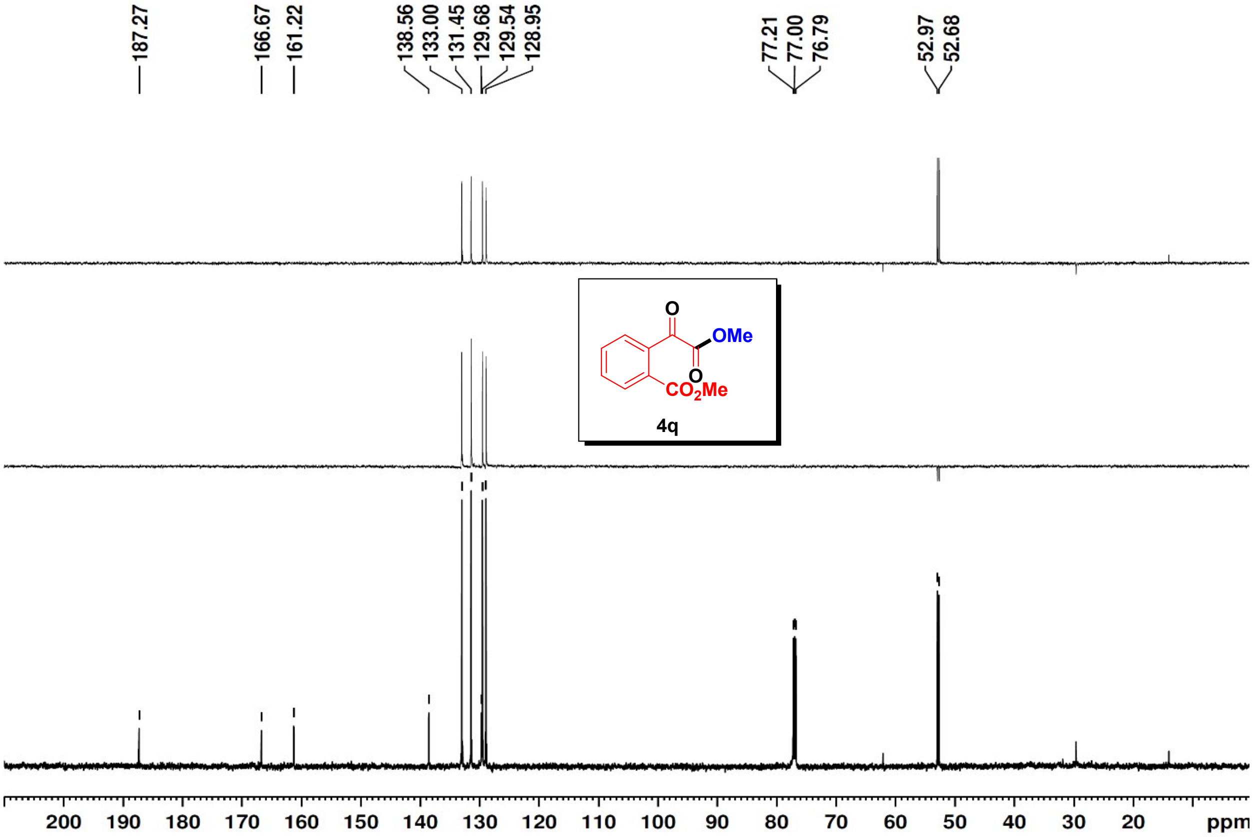
187.27
166.67
161.22
138.56
133.00
131.45
129.68
129.54
128.95
77.21
77.00
76.79
52.97
52.68
OMe
CO2Me
4q
200
190
180
170
160
150
140
130
120
110
100
90
80
70
60
50
40
30
20
ppm

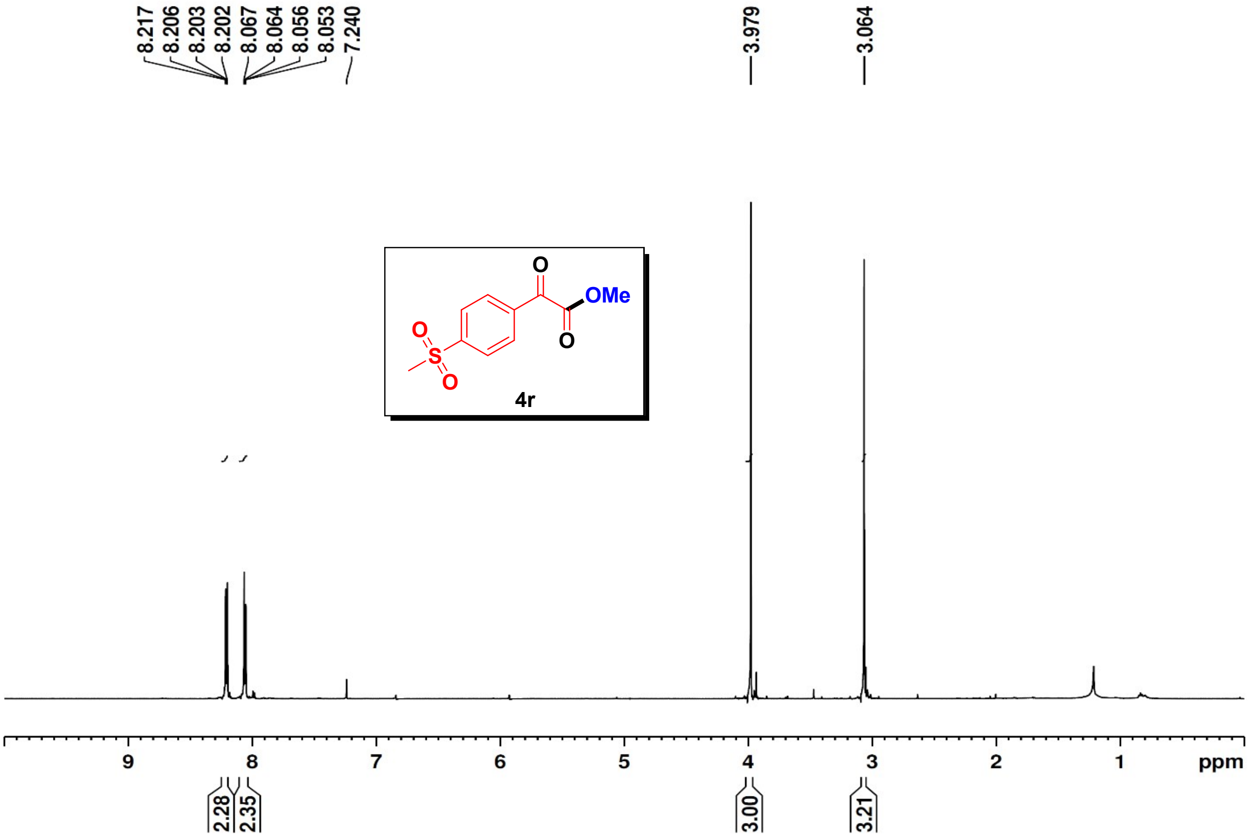
8.217
8.206
8.203
8.202
8.067
8.064
8.056
8.053
7.240
3.979
3.064
O
OMe
O
O
S
O
4r
9
8
7
6
5
4
3
2
1
ppm
2.28
2.35
3.00
3.21

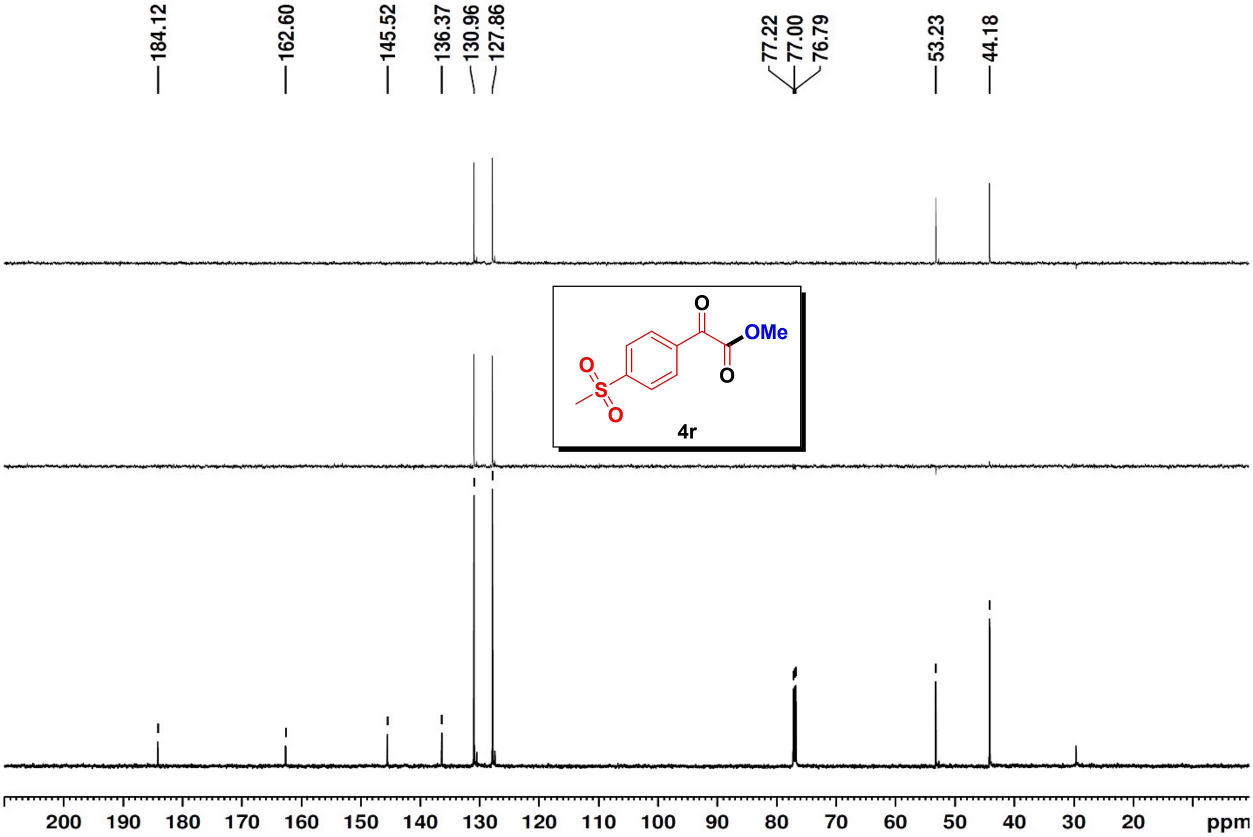
184.12
162.60
145.52
136.37
130.96
127.86
77.22
77.00
76.79
53.23
44.18
O
OMe
O
O
S
O
4r
200
190
180
170
160
150
140
130
120
110
100
90
80
70
60
50
40
30
20
ppm

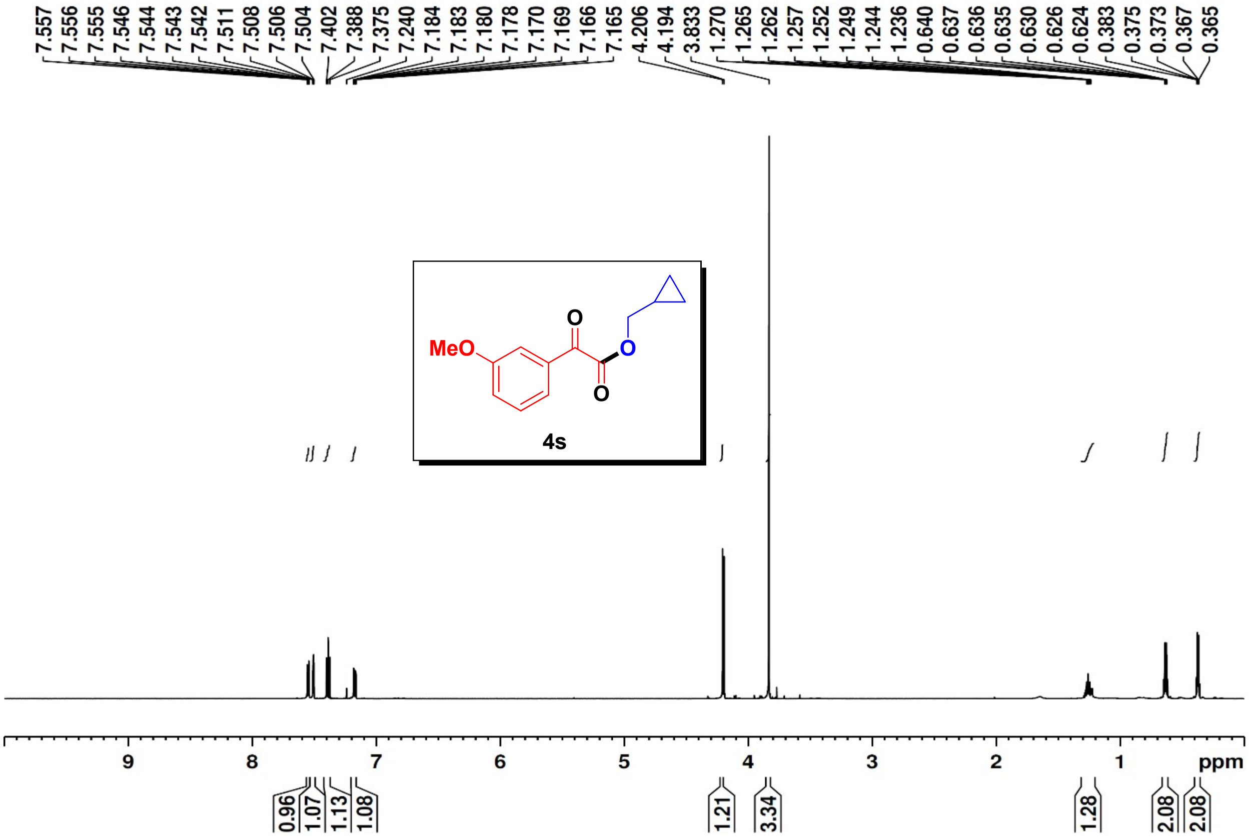
7.557
7.556
7.555
7.546
7.544
7.543
7.542
7.511
7.508
7.506
7.504
7.402
7.388
7.375
7.240
7.184
7.183
7.180
7.178
7.170
7.169
7.166
7.165
4.206
4.194
3.833
1.270
1.265
1.262
1.257
1.252
1.249
1.244
1.236
0.640
0.637
0.636
0.635
0.630
0.626
0.624
0.383
0.375
0.373
0.367
0.365
MeO
O
O
O
4s
9
8
7
6
5
4
3
2
1
ppm
0.96
1.07
1.13
1.08
1.21
3.34
1.28
2.08
2.08

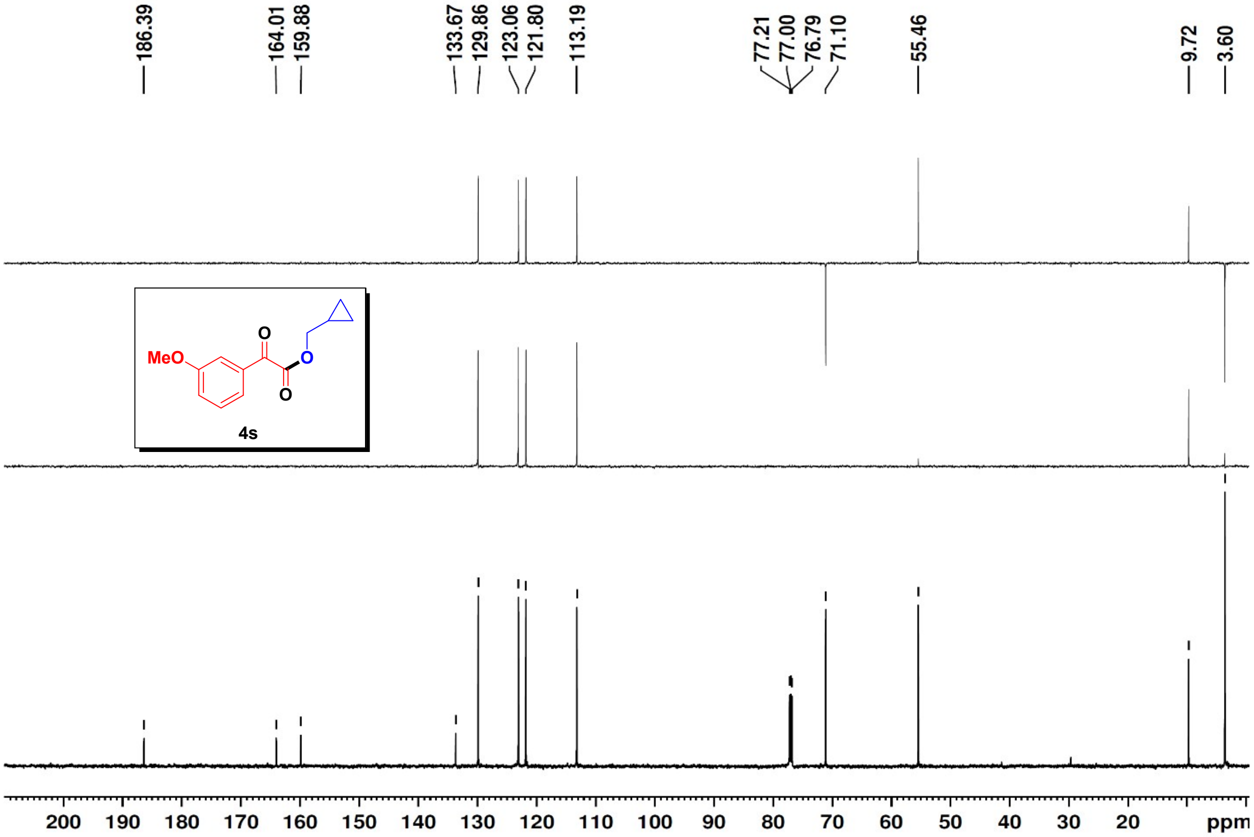
186.39
164.01
159.88
133.67
129.86
123.06
121.80
113.19
77.21
77.00
76.79
71.10
55.46
9.72
3.60
MeO
O
O
O
4s
200
190
180
170
160
150
140
130
120
110
100
90
80
70
60
50
40
30
20
ppm

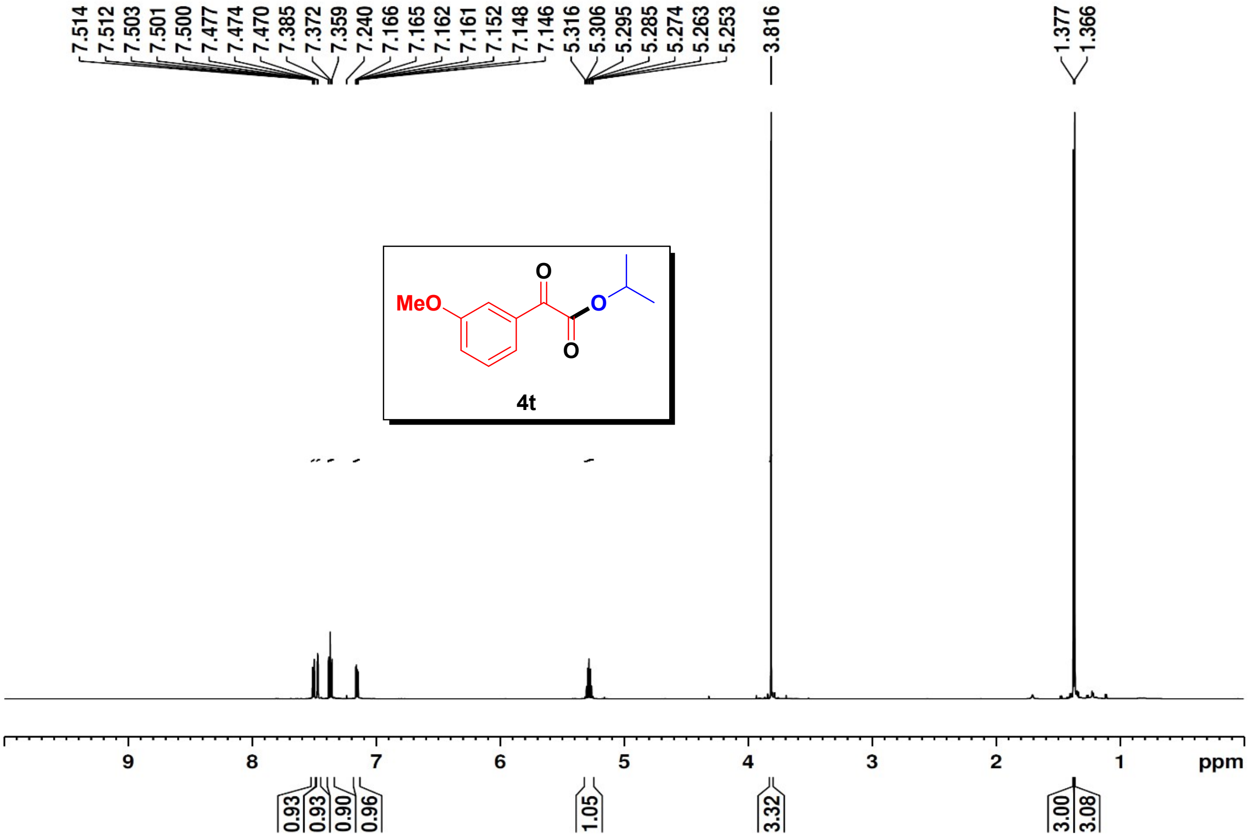
7.514 7.512 7.503 7.501 7.500 7.477 7.474 7.470 7.385 7.372 7.359 7.240 7.166 7.165 7.162 7.161 7.152 7.148 7.146 5.316 5.306 5.295 5.285 5.274 5.263 5.253 3.816 1.377 1.366
MeO
O
O
O
4t
9 8 7 6 5 4 3 2 1 ppm
0.93 0.93 0.90 0.96 1.05 3.32 3.00 3.08

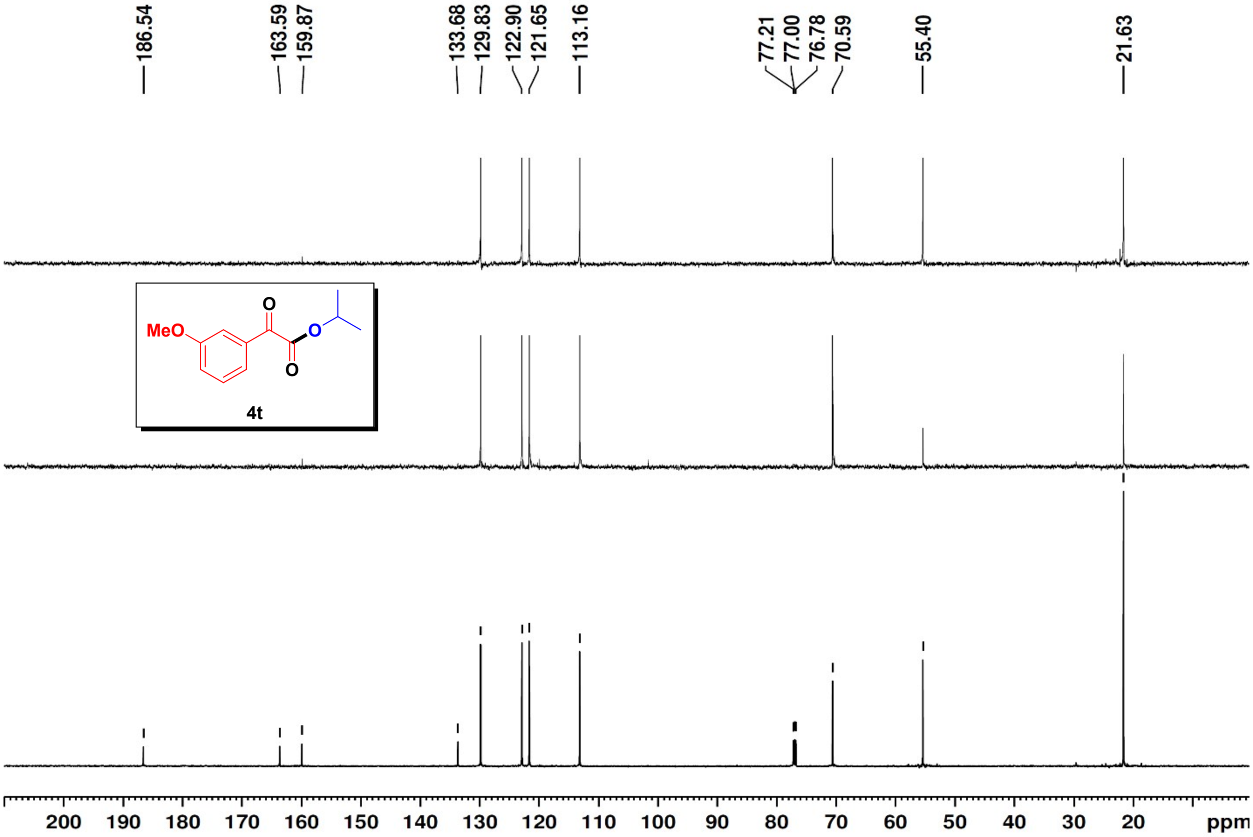
186.54 163.59 159.87 133.68 129.83 122.90 121.65 113.16 77.21 77.00 76.78 70.59 55.40 21.63
MeO
O
O
O
4t
200 190 180 170 160 150 140 130 120 110 100 90 80 70 60 50 40 30 20 ppm

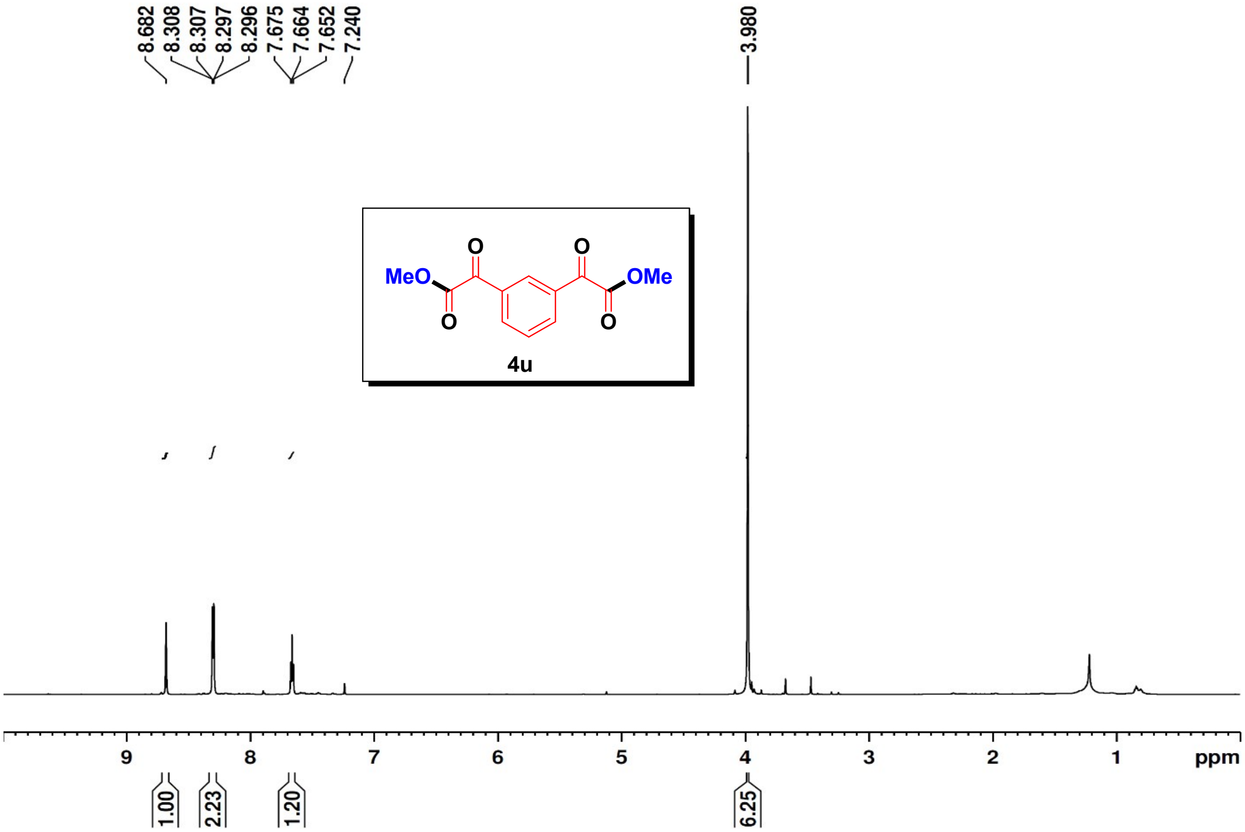
8.682
8.308
8.307
8.297
8.296
7.675
7.664
7.652
7.240
3.980
MeO
O
O
O
O
OMe
4u
9
8
7
6
5
4
3
2
1
ppm
1.00
2.23
1.20
6.25

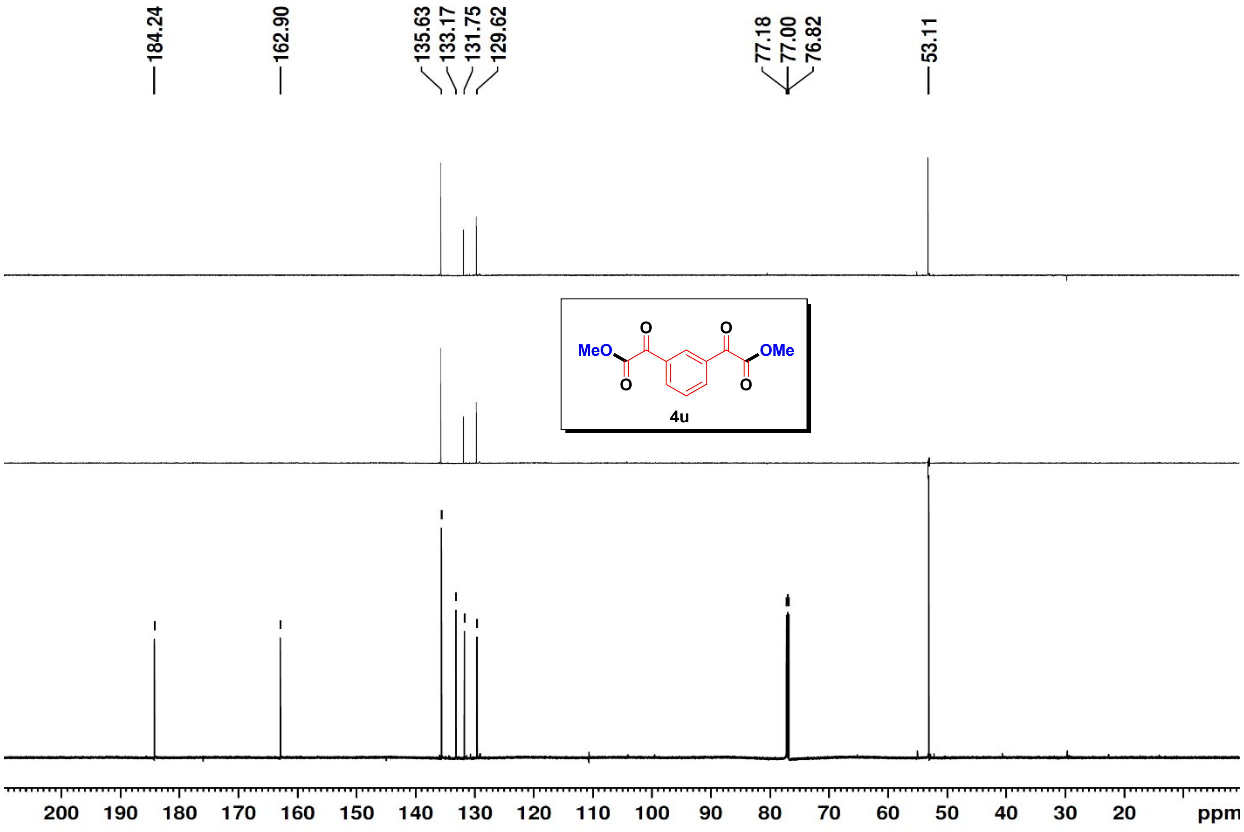
184.24
162.90
135.63
133.17
131.75
129.62
77.18
77.00
76.82
53.11
MeO
O
O
O
O
OMe
4u
200
190
180
170
160
150
140
130
120
110
100
90
80
70
60
50
40
30
20
ppm

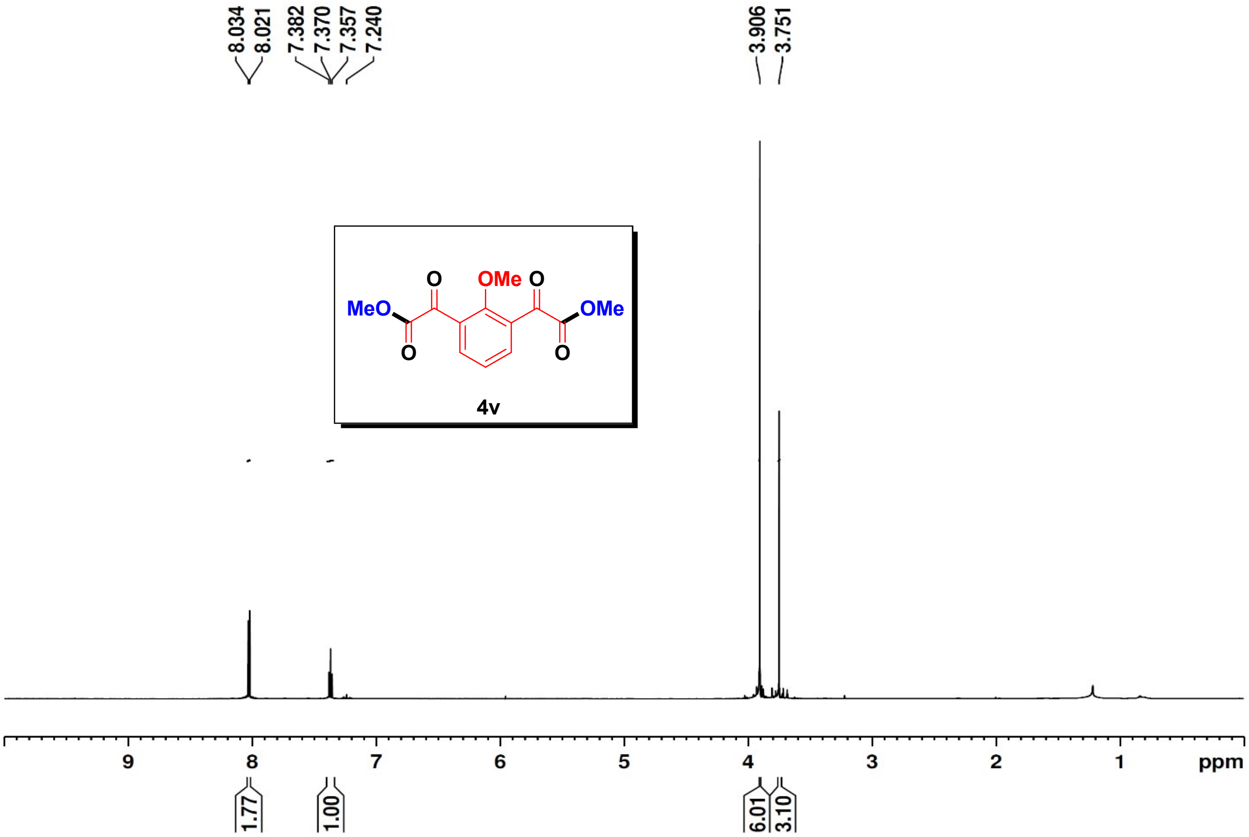

8.034
8.021
7.382
7.370
7.357
7.240
3.906
3.751
MeO
OMe
OMe
4v
1.77
1.00
6.01
3.10
9
8
7
6
5
4
3
2
1
ppm

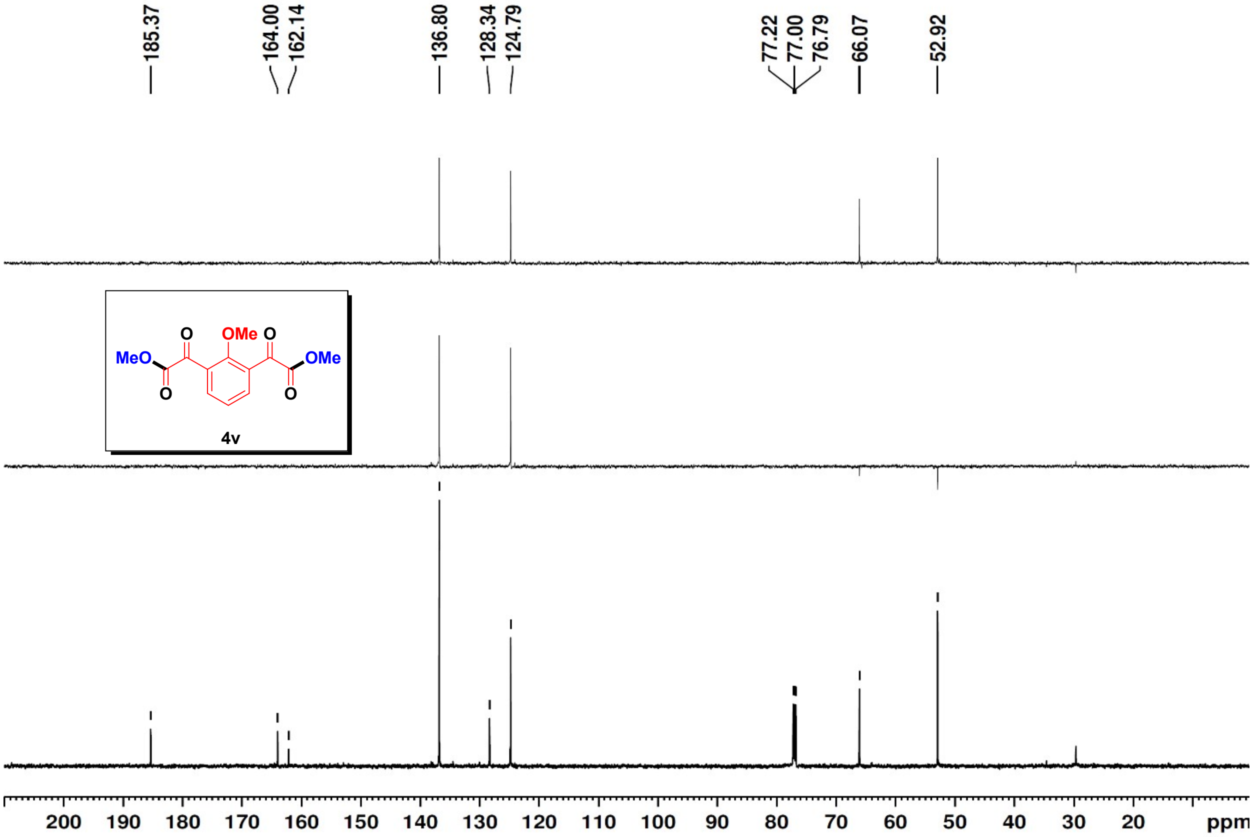

185.37
164.00
162.14
136.80
128.34
124.79
77.22
77.00
76.79
66.07
52.92
MeO
OMe
OMe
4v
200
190
180
170
160
150
140
130
120
110
100
90
80
70
60
50
40
30
20
ppm

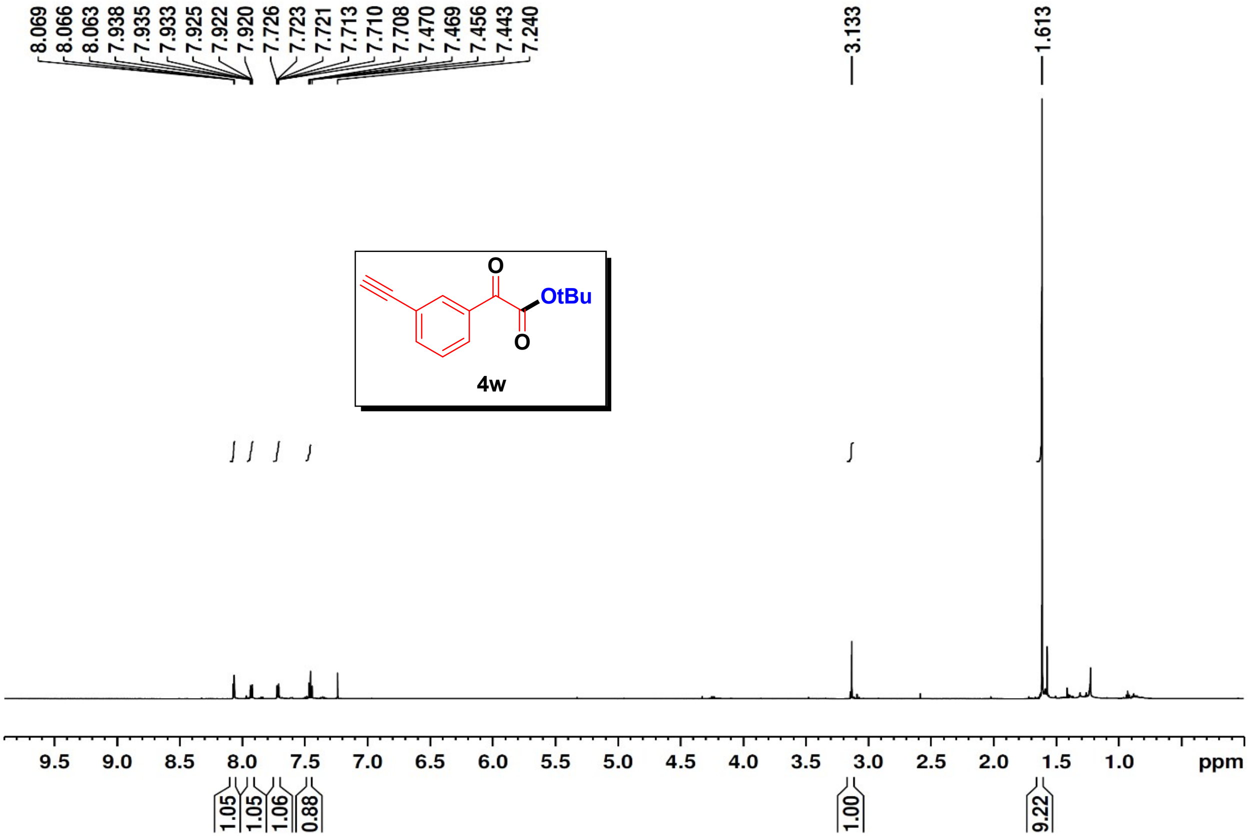
8.069
8.066
8.063
7.938
7.935
7.933
7.925
7.922
7.920
7.726
7.723
7.721
7.713
7.710
7.708
7.470
7.469
7.456
7.443
7.240
3.133
1.613
OtBu
O
O
4w
9.5 9.0 8.5 8.0 7.5 7.0 6.5 6.0 5.5 5.0 4.5 4.0 3.5 3.0 2.5 2.0 1.5 1.0 ppm
1.05
1.05
1.06
0.88
1.00
9.22

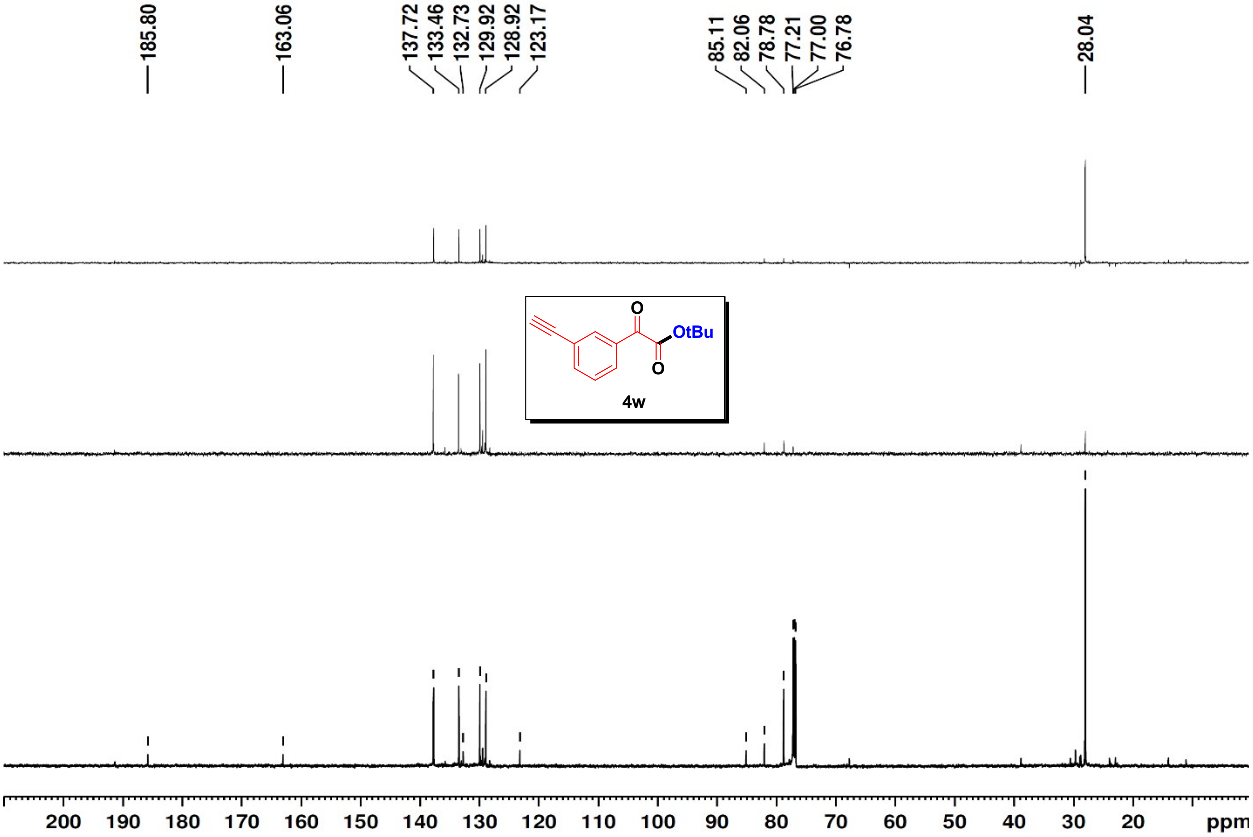
185.80
163.06
137.72
133.46
132.73
129.92
128.92
123.17
85.11
82.06
78.78
77.21
77.00
76.78
28.04
OtBu
O
O
4w
200 190 180 170 160 150 140 130 120 110 100 90 80 70 60 50 40 30 20 ppm

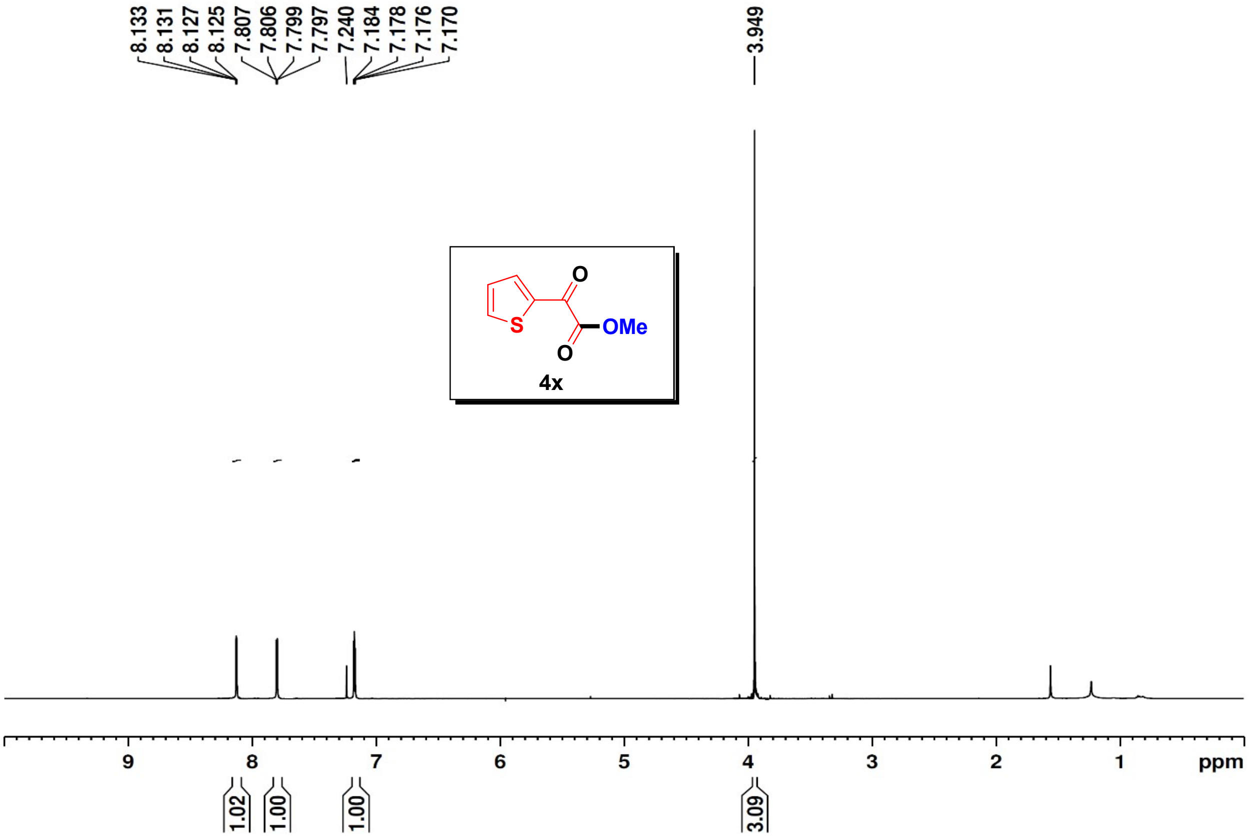
8.133
8.131
8.127
8.125
7.807
7.806
7.799
7.797
7.240
7.184
7.178
7.176
7.170
3.949
OMe
4x
1.02
1.00
1.00
3.09
ppm

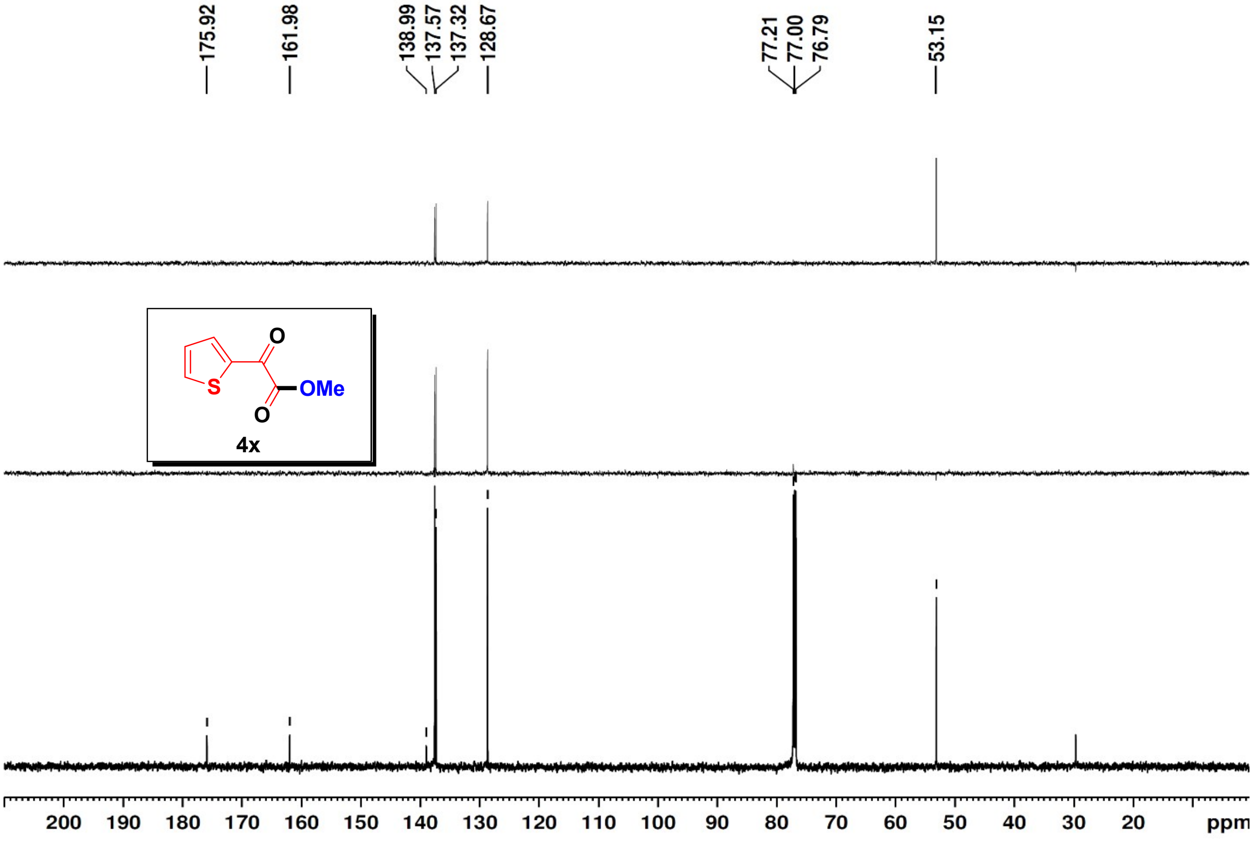
175.92
161.98
138.99
137.57
137.32
128.67
77.21
77.00
76.79
53.15
OMe
4x
ppm

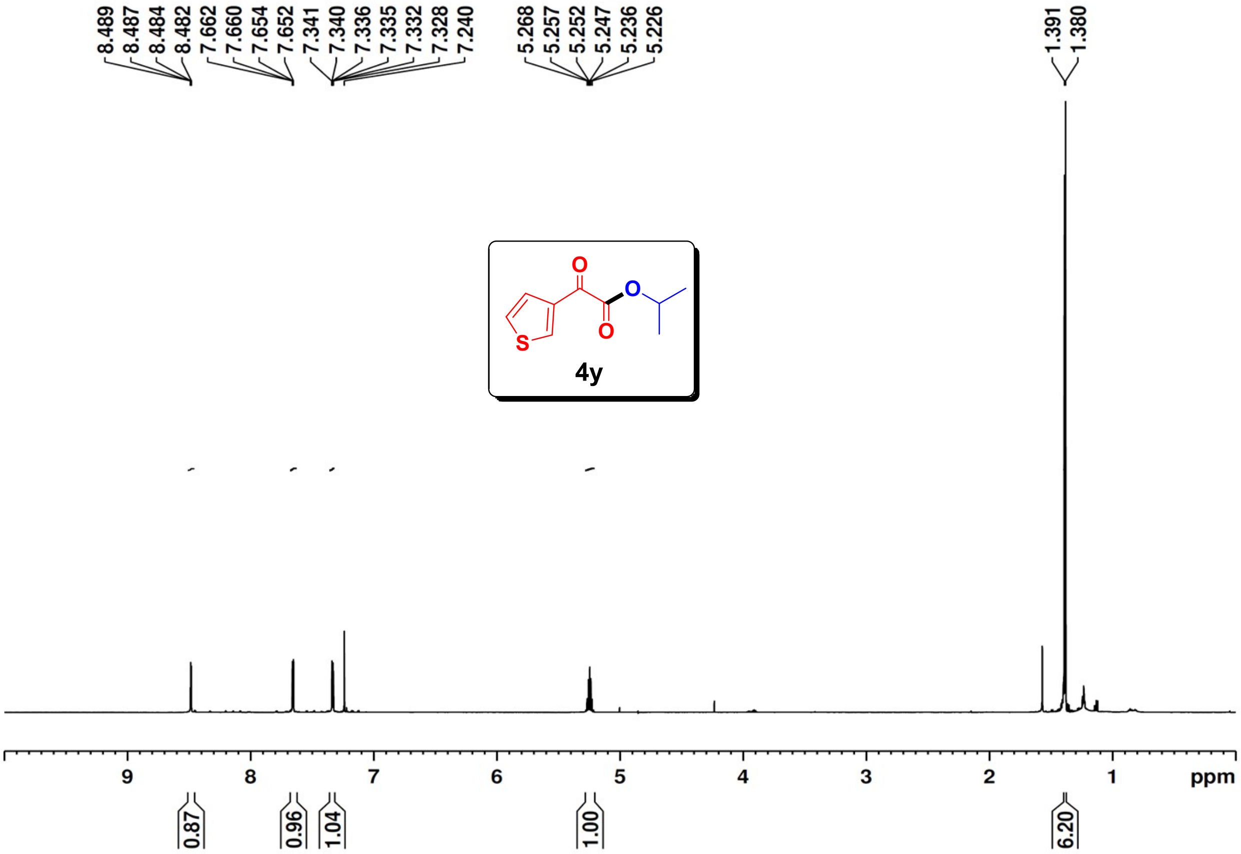
8.489
8.487
8.484
8.482
7.662
7.660
7.654
7.652
7.341
7.340
7.336
7.335
7.332
7.328
7.240
5.268
5.257
5.252
5.247
5.236
5.226
1.391
1.380
4y
0.87
0.96
1.04
1.00
6.20
ppm

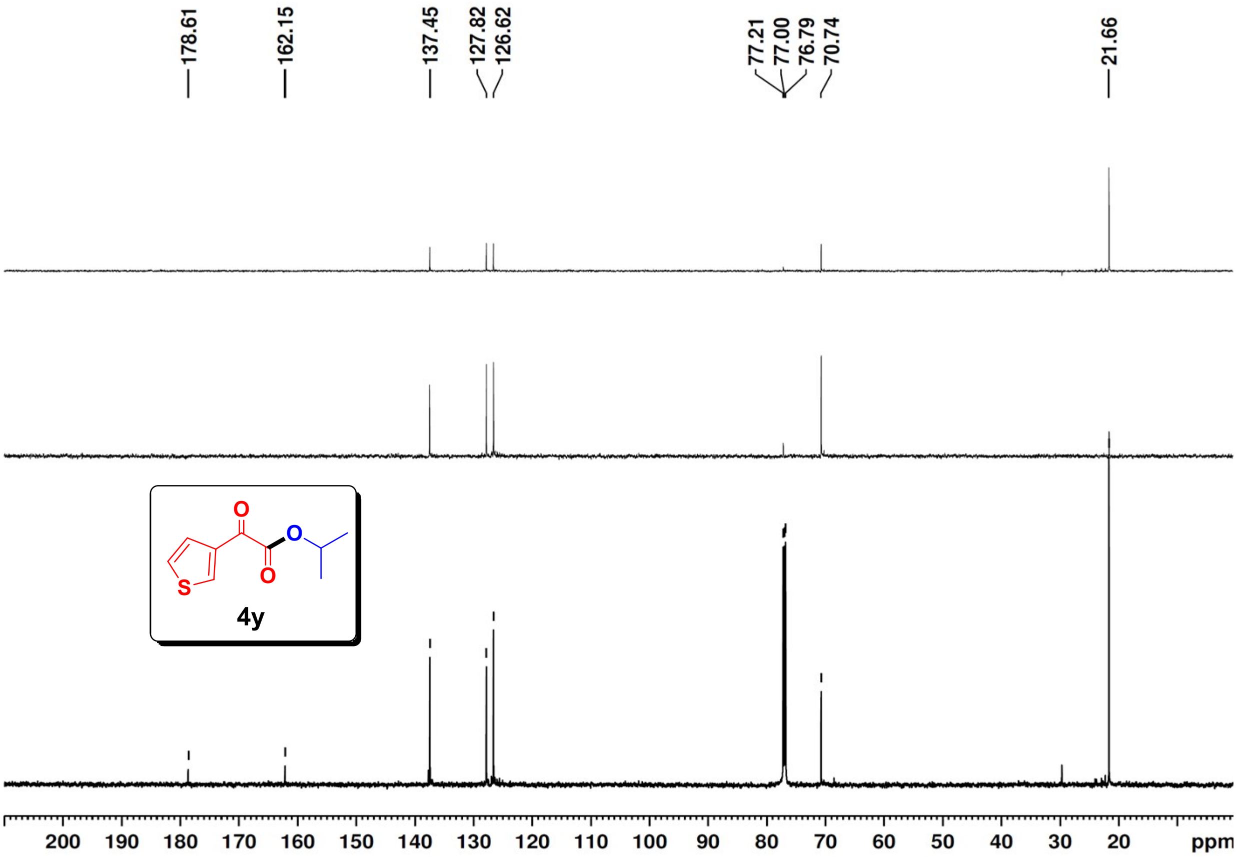
178.61
162.15
137.45
127.82
126.62
77.21
77.00
76.79
70.74
21.66
4y
ppm

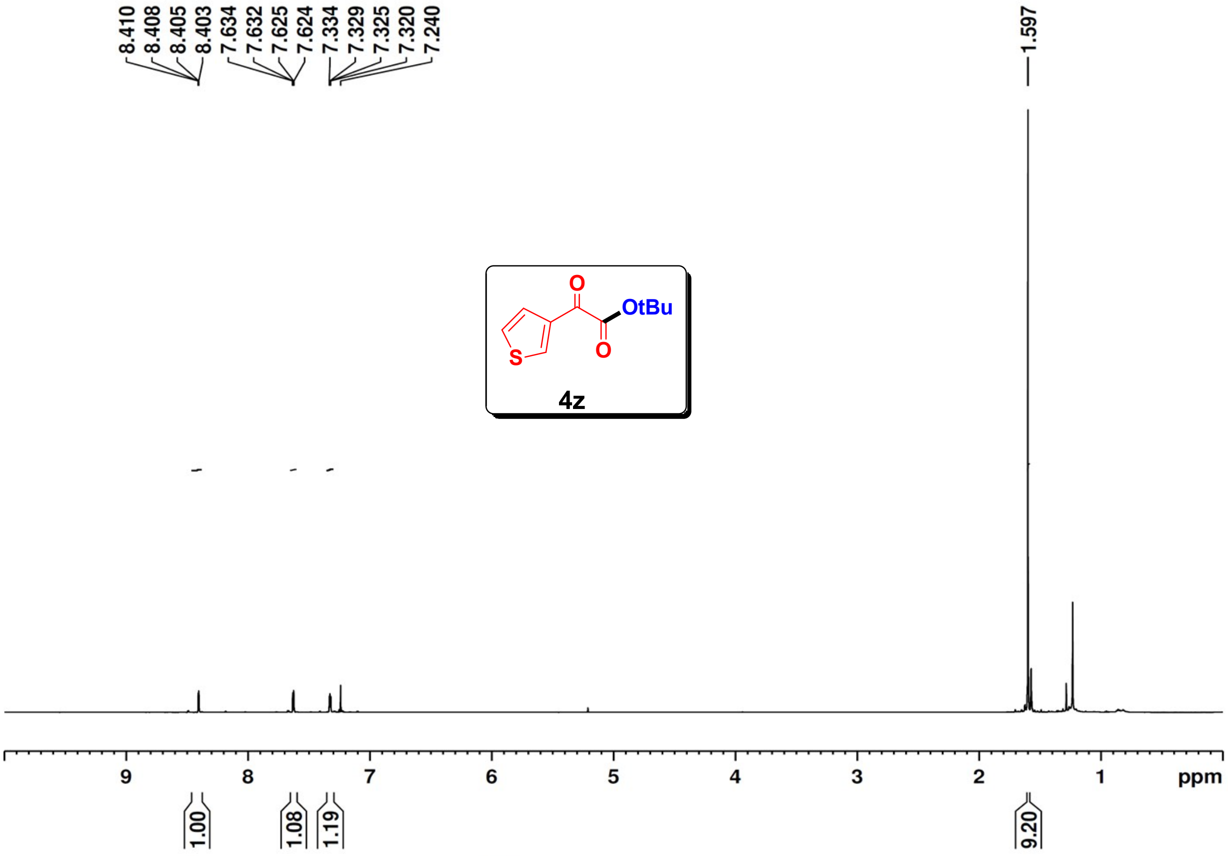
8.410
8.408
8.405
8.403
7.634
7.632
7.625
7.624
7.334
7.329
7.325
7.320
7.240
1.597
OtBu
O
O
S
4z
9
8
7
6
5
4
3
2
1
ppm
1.00
1.08
1.19
9.20

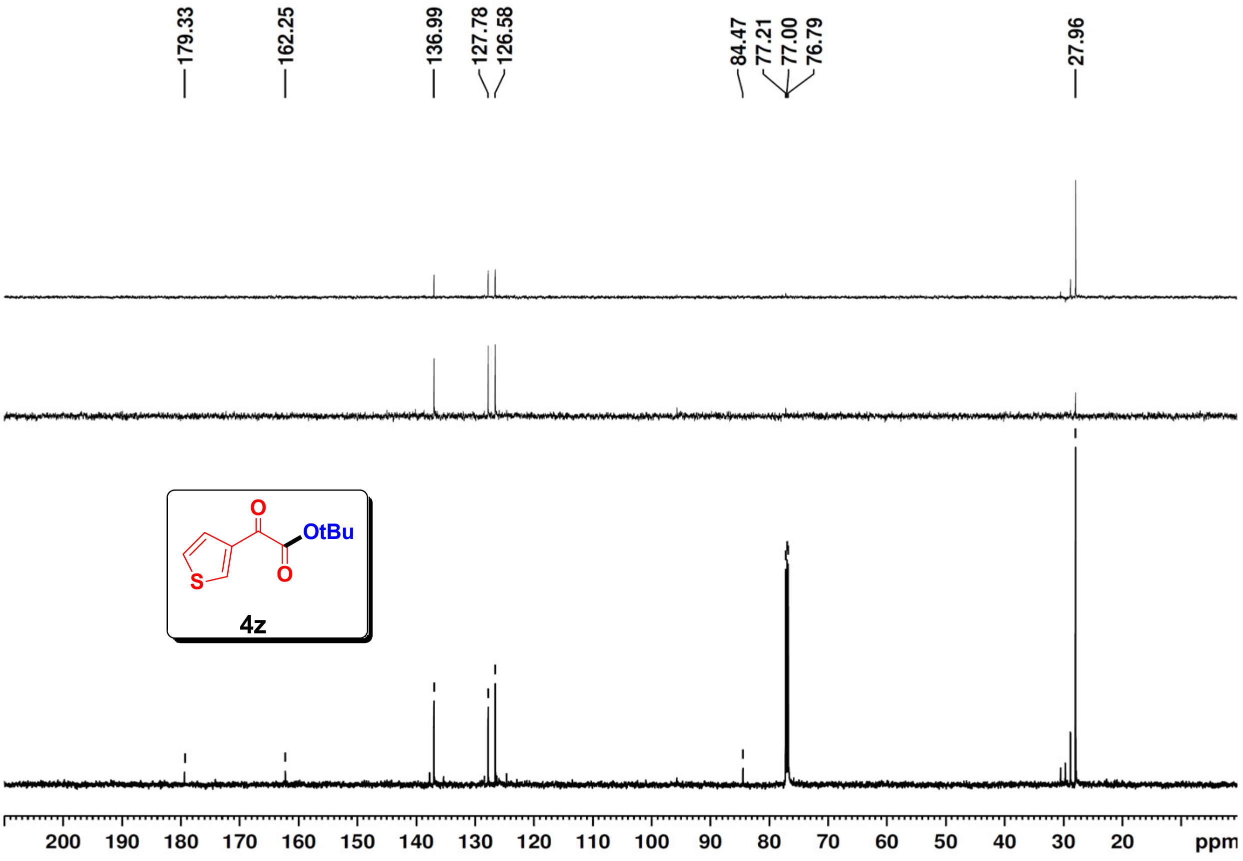
179.33
162.25
136.99
127.78
126.58
84.47
77.21
77.00
76.79
27.96
OtBu
O
O
S
4z
200
190
180
170
160
150
140
130
120
110
100
90
80
70
60
50
40
30
20
ppm

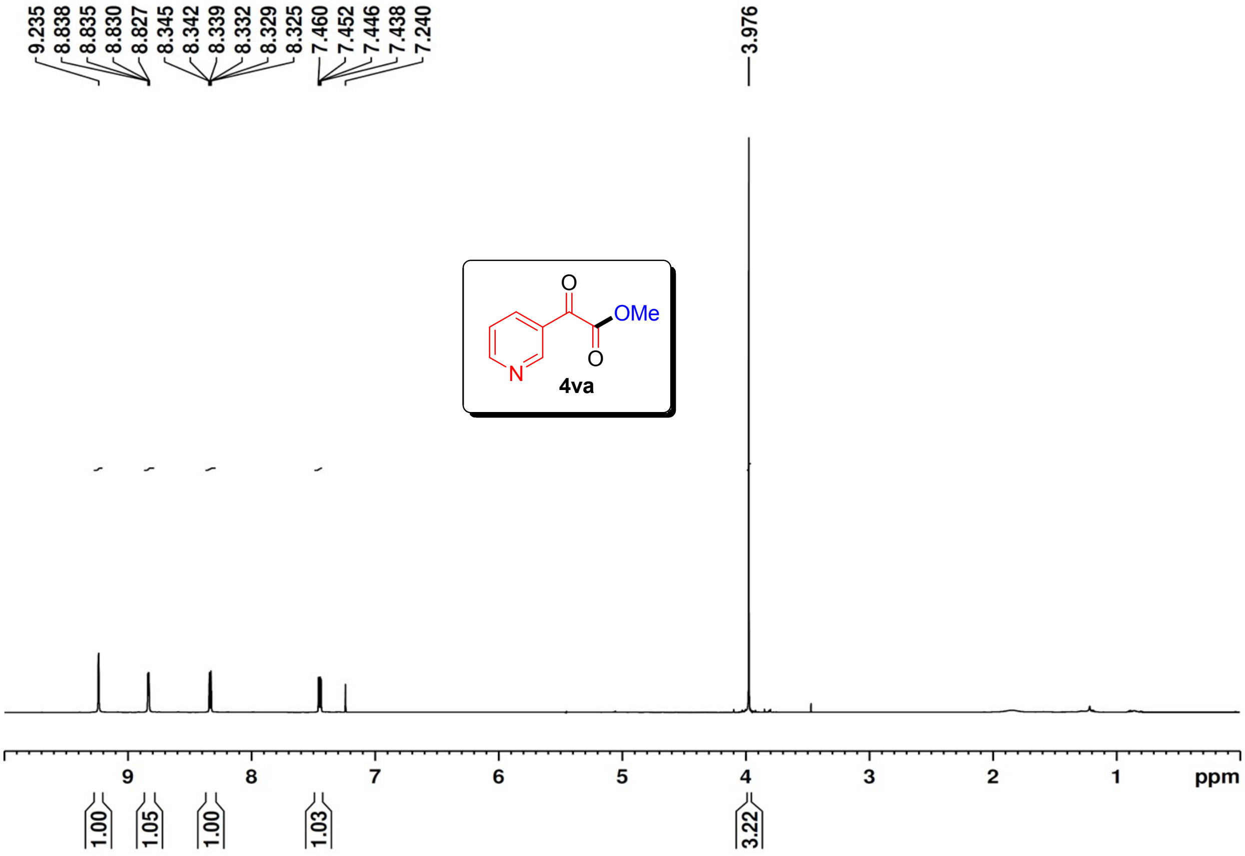
9.235
8.838
8.835
8.830
8.827
8.345
8.342
8.339
8.332
8.329
8.325
7.460
7.452
7.446
7.438
7.240
3.976
O
OMe
N
O
4va
9
8
7
6
5
4
3
2
1
ppm
1.00
1.05
1.00
1.03
3.22

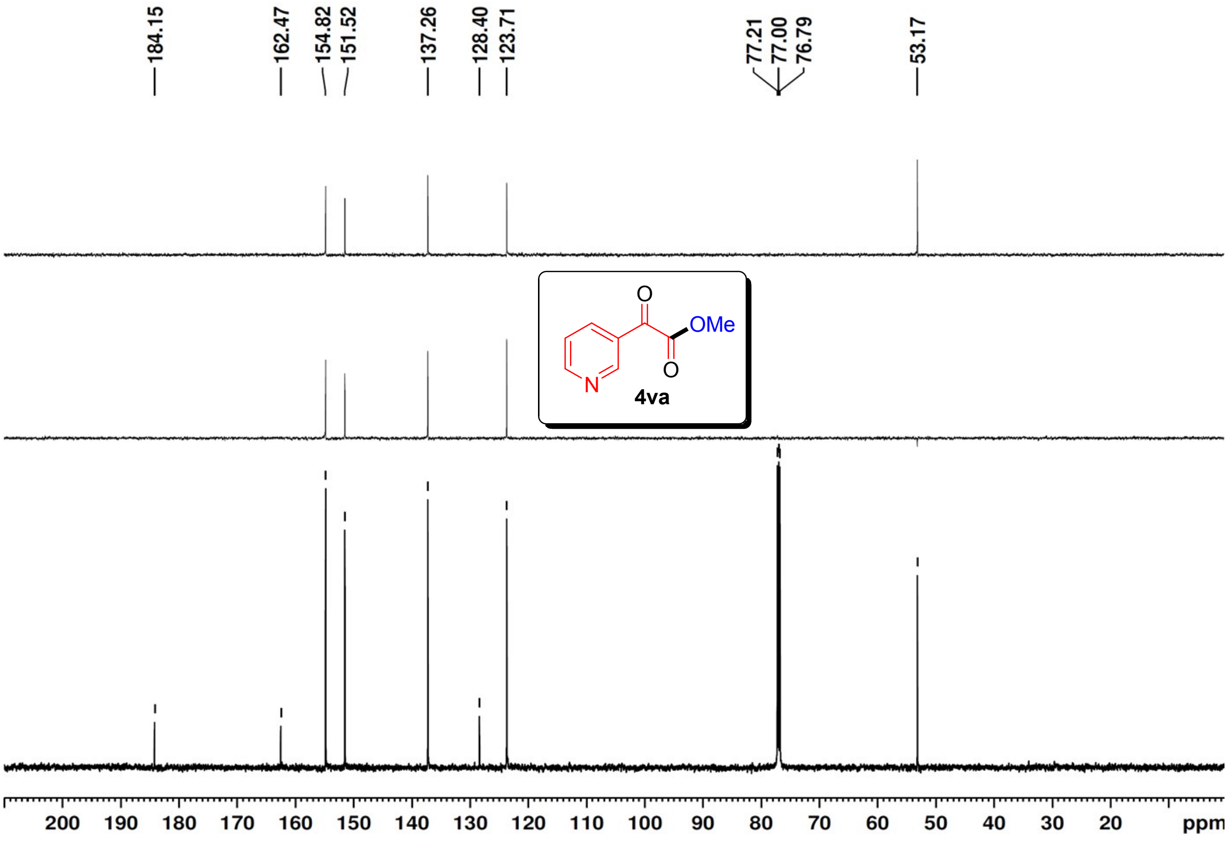
184.15
162.47
154.82
151.52
137.26
128.40
123.71
77.21
77.00
76.79
53.17
O
OMe
N
O
4va
200
190
180
170
160
150
140
130
120
110
100
90
80
70
60
50
40
30
20
ppm

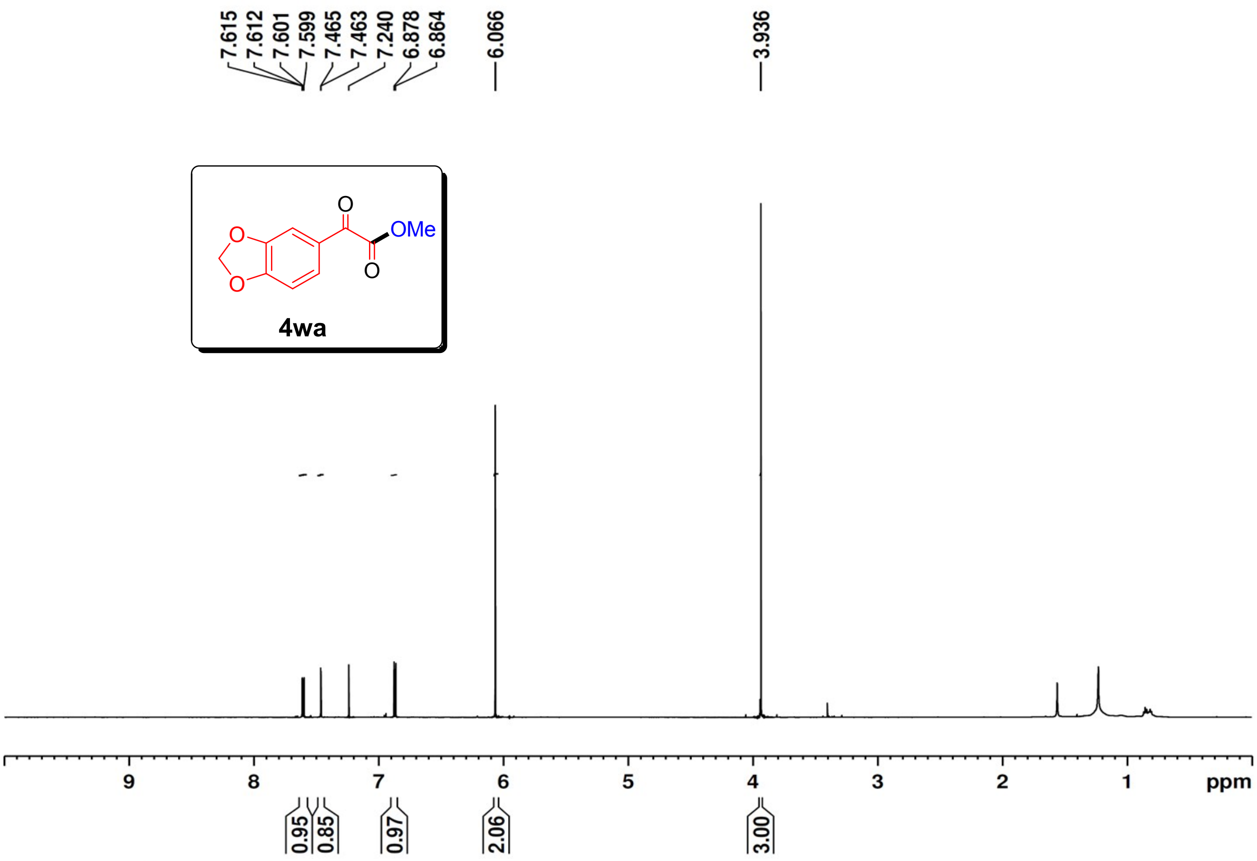
7.615
7.612
7.601
7.599
7.465
7.463
7.240
6.878
6.864
6.066
3.936
OMe
4wa
0.95
0.85
0.97
2.06
3.00
ppm

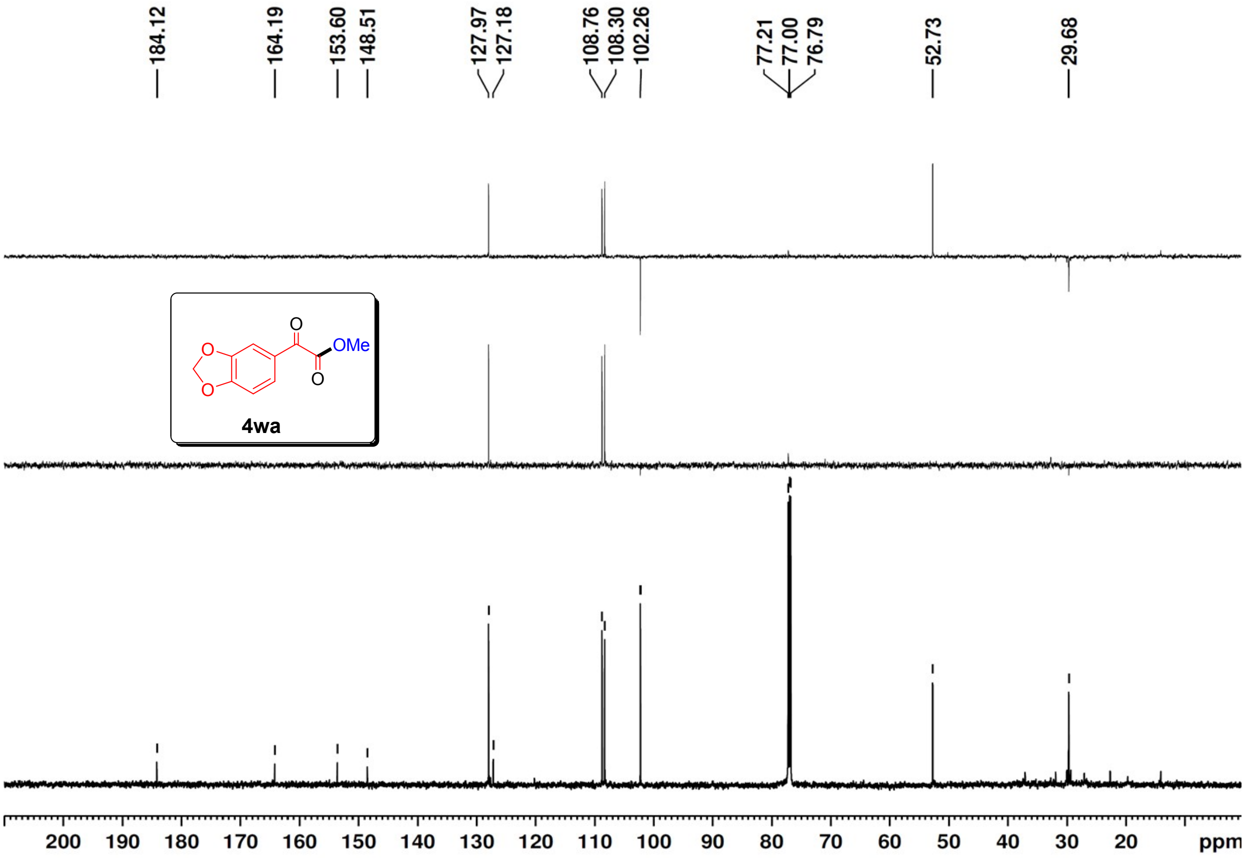
184.12
164.19
153.60
148.51
127.97
127.18
108.76
108.30
102.26
77.21
77.00
76.79
52.73
29.68
OMe
4wa
ppm

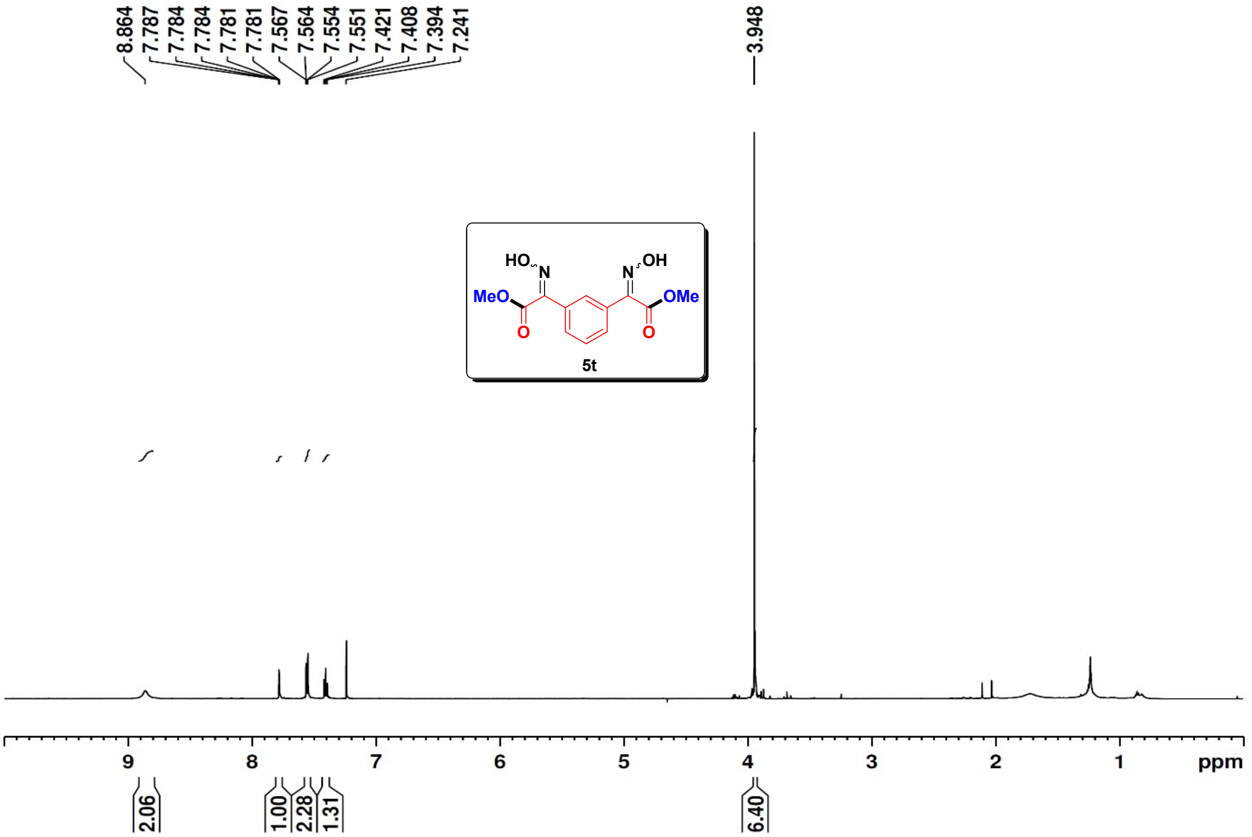
8.864
7.787
7.784
7.784
7.781
7.781
7.567
7.564
7.554
7.551
7.421
7.408
7.394
7.241
3.948
HO
N
N
OH
MeO
OMe
O
O
5t
9
8
7
6
5
4
3
2
1
ppm
2.06
1.00
2.28
1.31
6.40

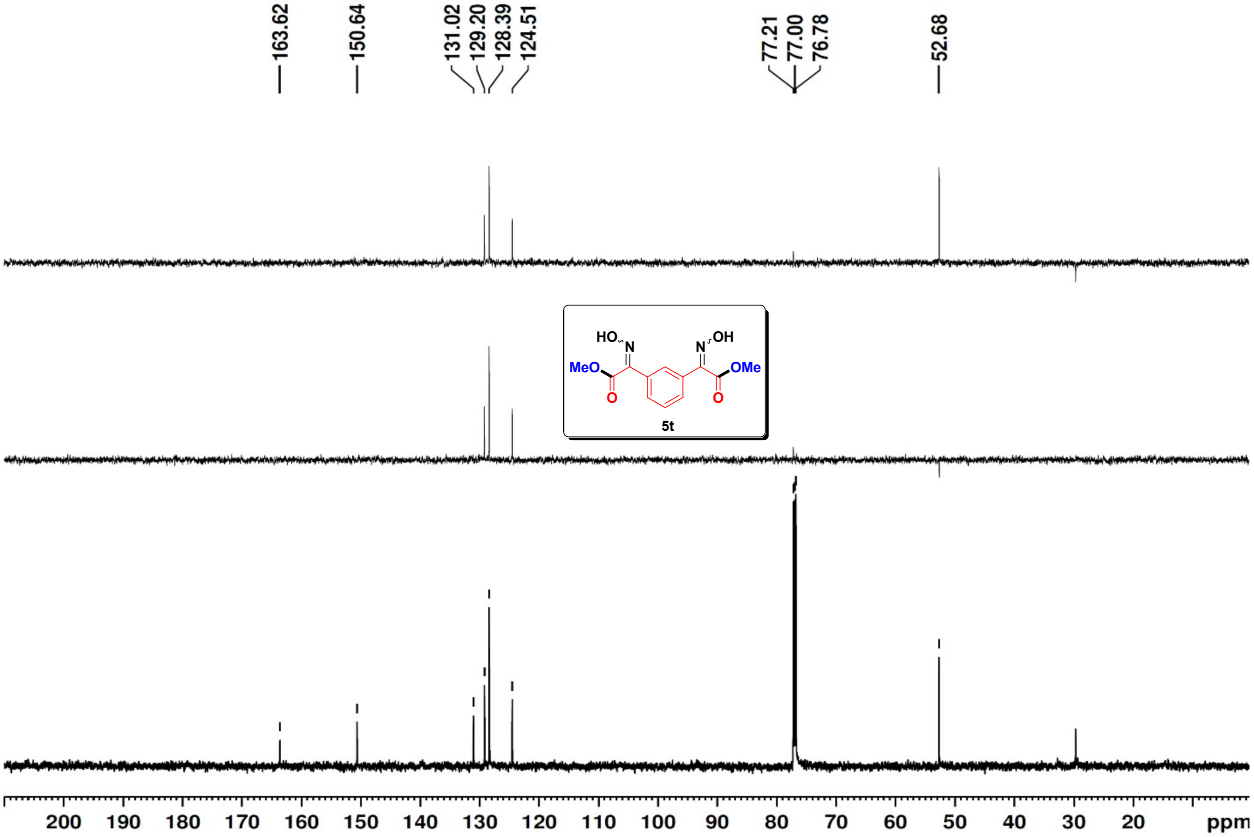
163.62
150.64
131.02
129.20
128.39
124.51
77.21
77.00
76.78
52.68
HO
N
N
OH
MeO
OMe
O
O
5t
200
190
180
170
160
150
140
130
120
110
100
90
80
70
60
50
40
30
20
ppm

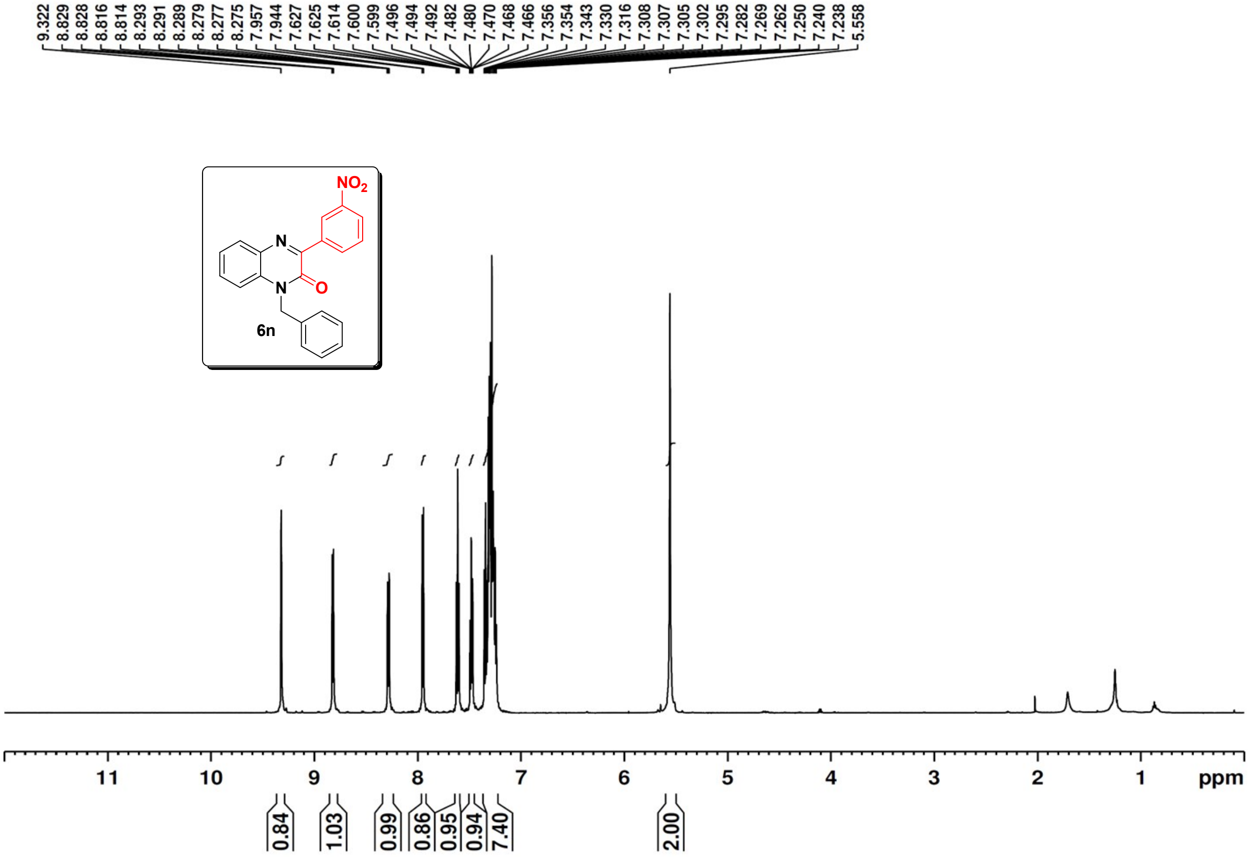
9.322
8.829
8.828
8.816
8.814
8.293
8.291
8.289
8.279
8.277
8.275
7.957
7.944
7.627
7.625
7.614
7.600
7.599
7.496
7.494
7.492
7.482
7.480
7.470
7.468
7.466
7.356
7.354
7.343
7.330
7.316
7.308
7.307
7.305
7.302
7.295
7.282
7.269
7.262
7.250
7.240
7.238
5.558
NO2
N
N
O
6n
0.84
1.03
0.99
0.86
0.95
0.94
7.40
2.00
11 10 9 8 7 6 5 4 3 2 1 ppm

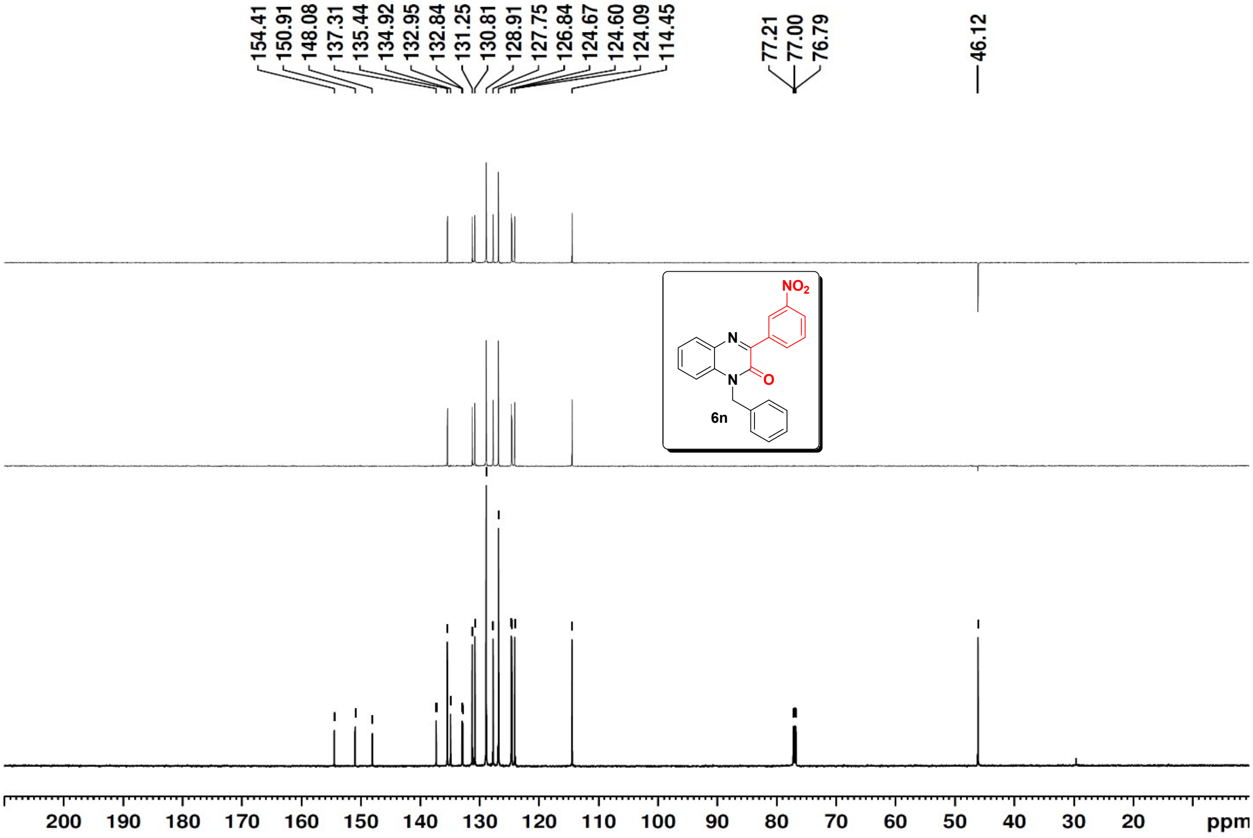
154.41
150.91
148.08
137.31
135.44
134.92
132.95
132.84
131.25
130.81
128.91
127.75
126.84
124.67
124.60
124.09
114.45
77.21
77.00
76.79
46.12
NO2
N
N
O
6n
200 190 180 170 160 150 140 130 120 110 100 90 80 70 60 50 40 30 20 ppm

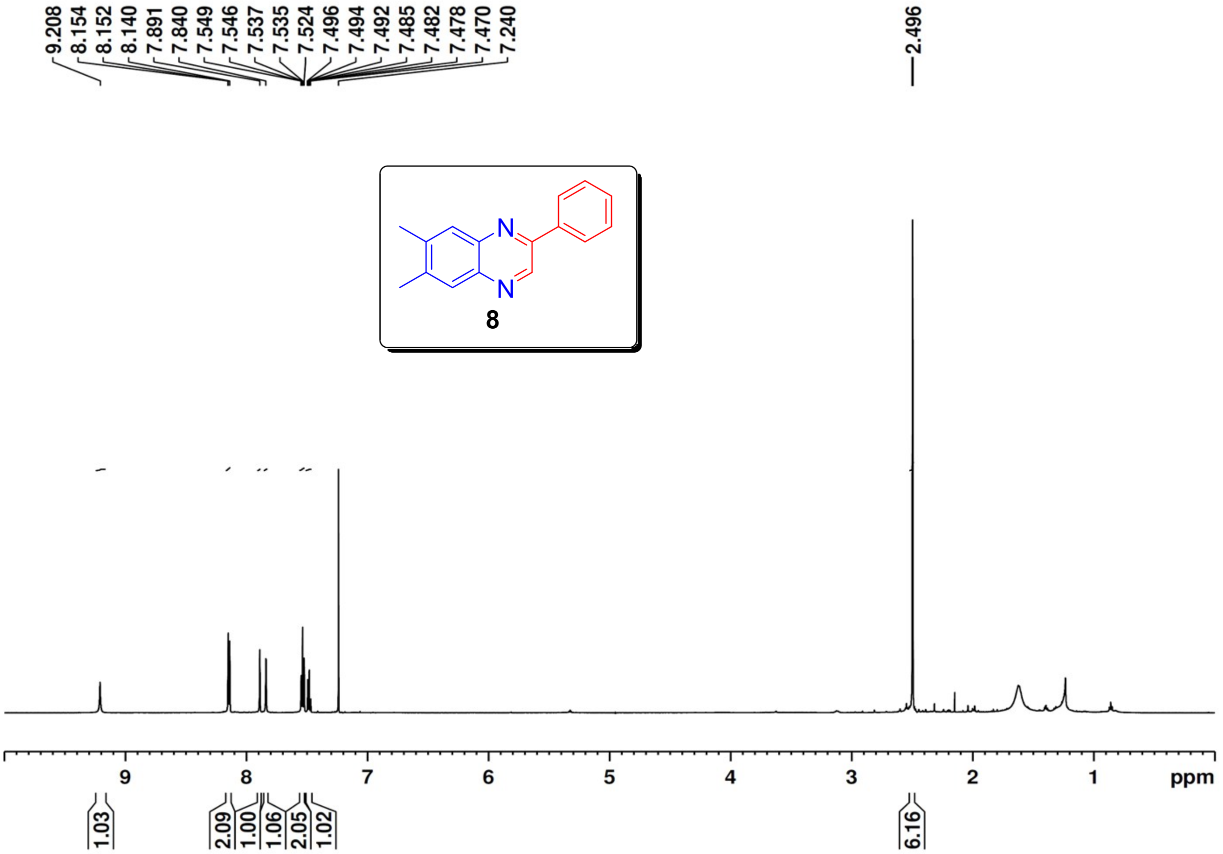
9.208
8.154
8.152
8.140
7.891
7.840
7.549
7.546
7.537
7.535
7.524
7.496
7.494
7.492
7.485
7.482
7.478
7.470
7.240
2.496
N
N
8
9
8
7
6
5
4
3
2
1
ppm
1.03
2.09
1.00
1.06
2.05
1.02
6.16

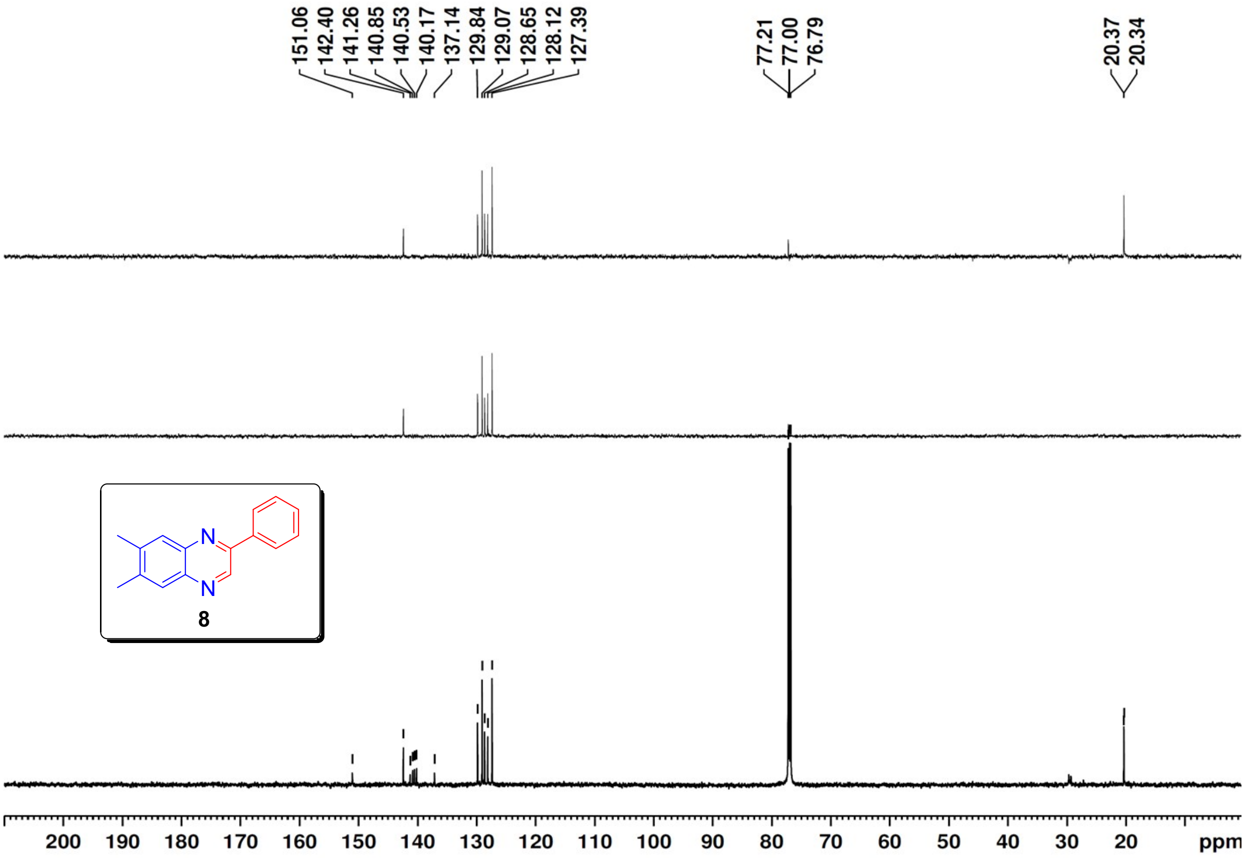
151.06
142.40
141.26
140.85
140.53
140.17
137.14
129.84
129.07
128.65
128.12
127.39
77.21
77.00
76.79
20.37
20.34
N
N
8
200
190
180
170
160
150
140
130
120
110
100
90
80
70
60
50
40
30
20
ppm

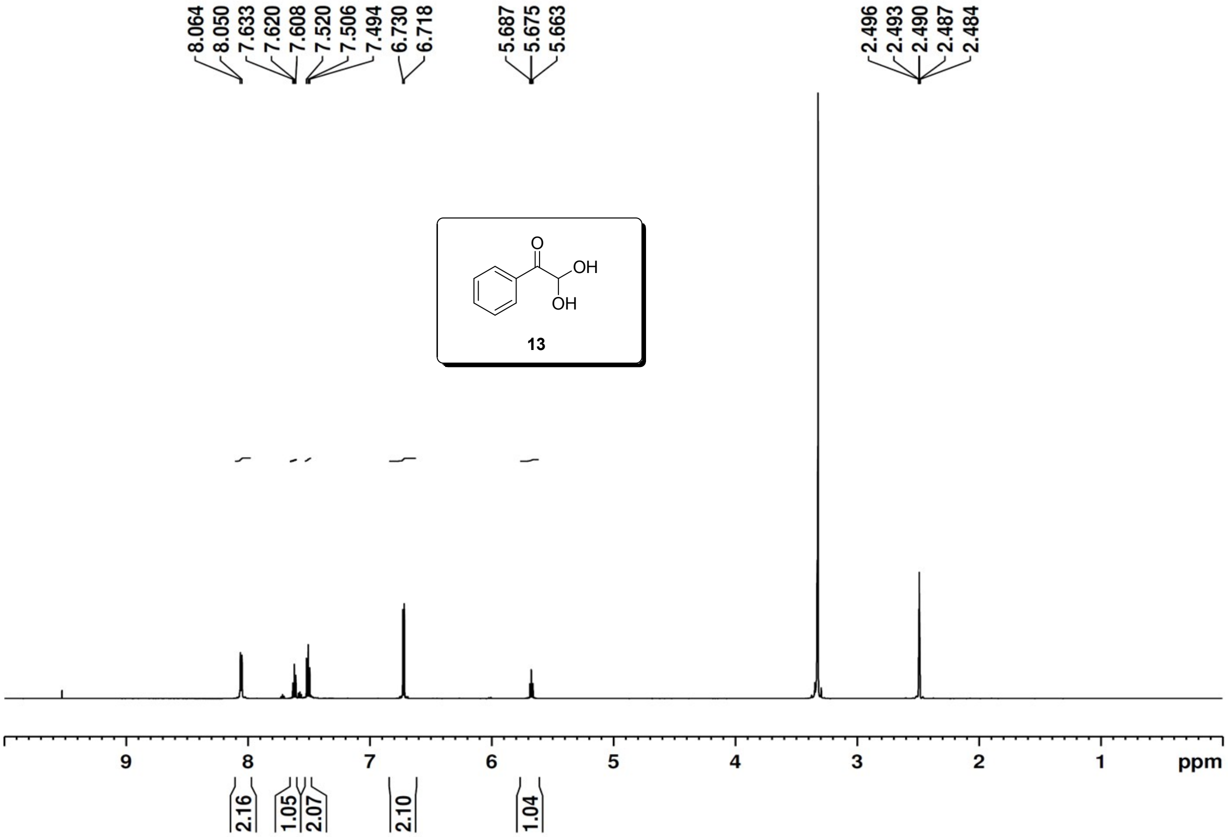
8.064
8.050
7.633
7.620
7.608
7.520
7.506
7.494
6.730
6.718
5.687
5.675
5.663
2.496
2.493
2.490
2.487
2.484
O
OH
OH
13
9
8
7
6
5
4
3
2
1
ppm
2.16
1.05
2.07
2.10
1.04

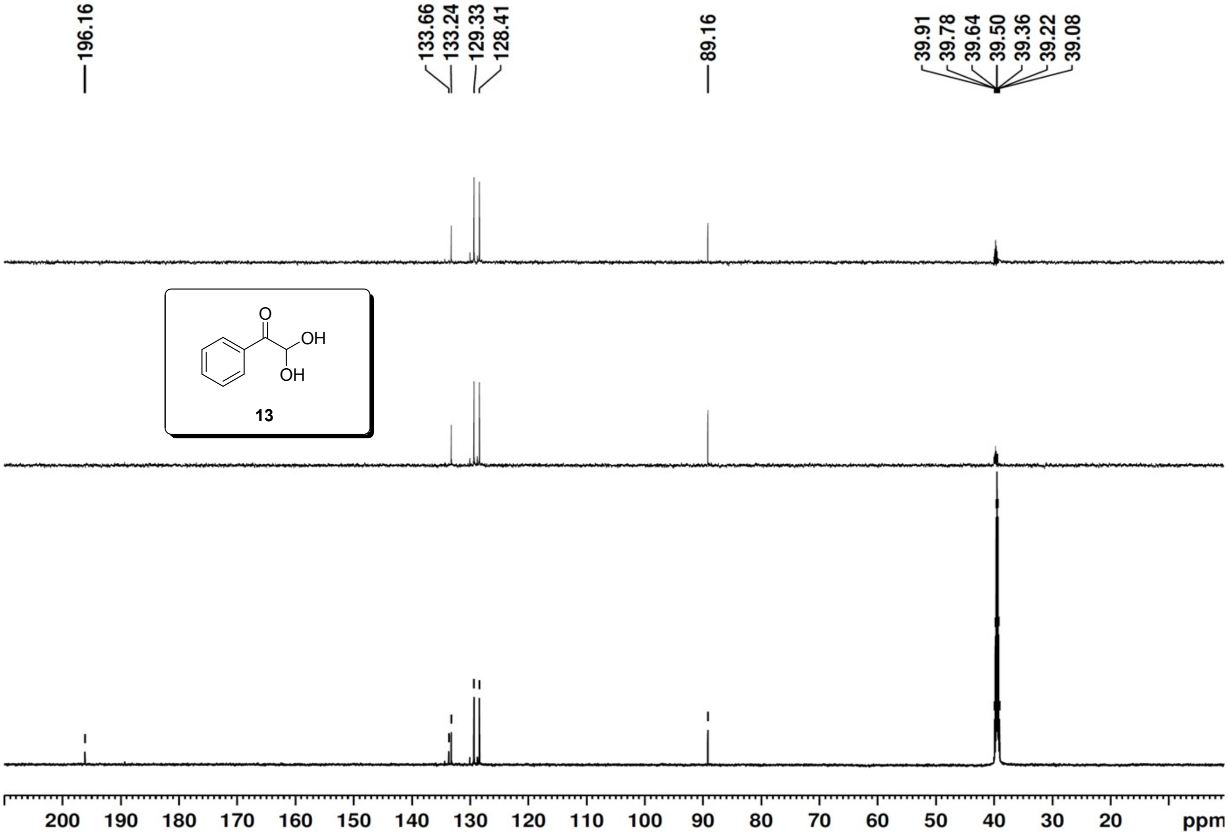
196.16
133.66
133.24
129.33
128.41
89.16
39.91
39.78
39.64
39.50
39.36
39.22
39.08
O
OH
OH
13
200
190
180
170
160
150
140
130
120
110
100
90
80
70
60
50
40
30
20
ppm

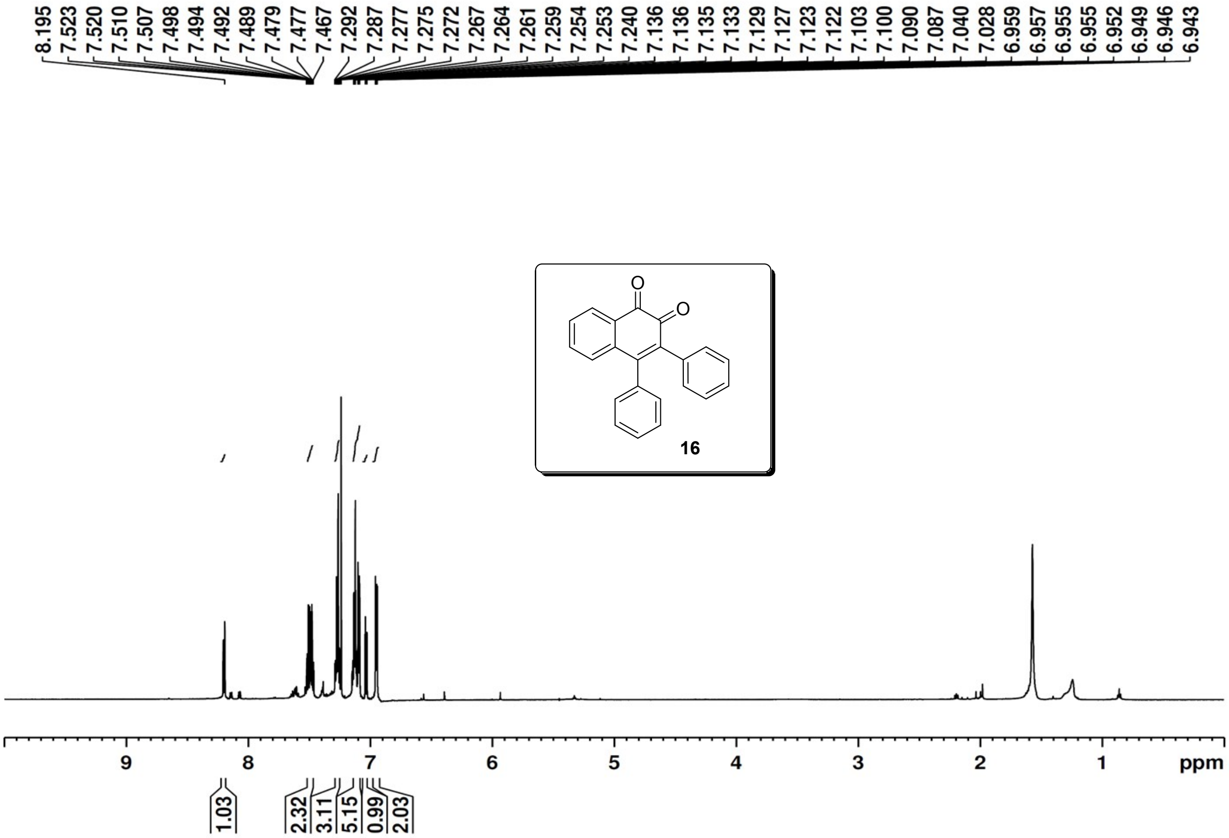

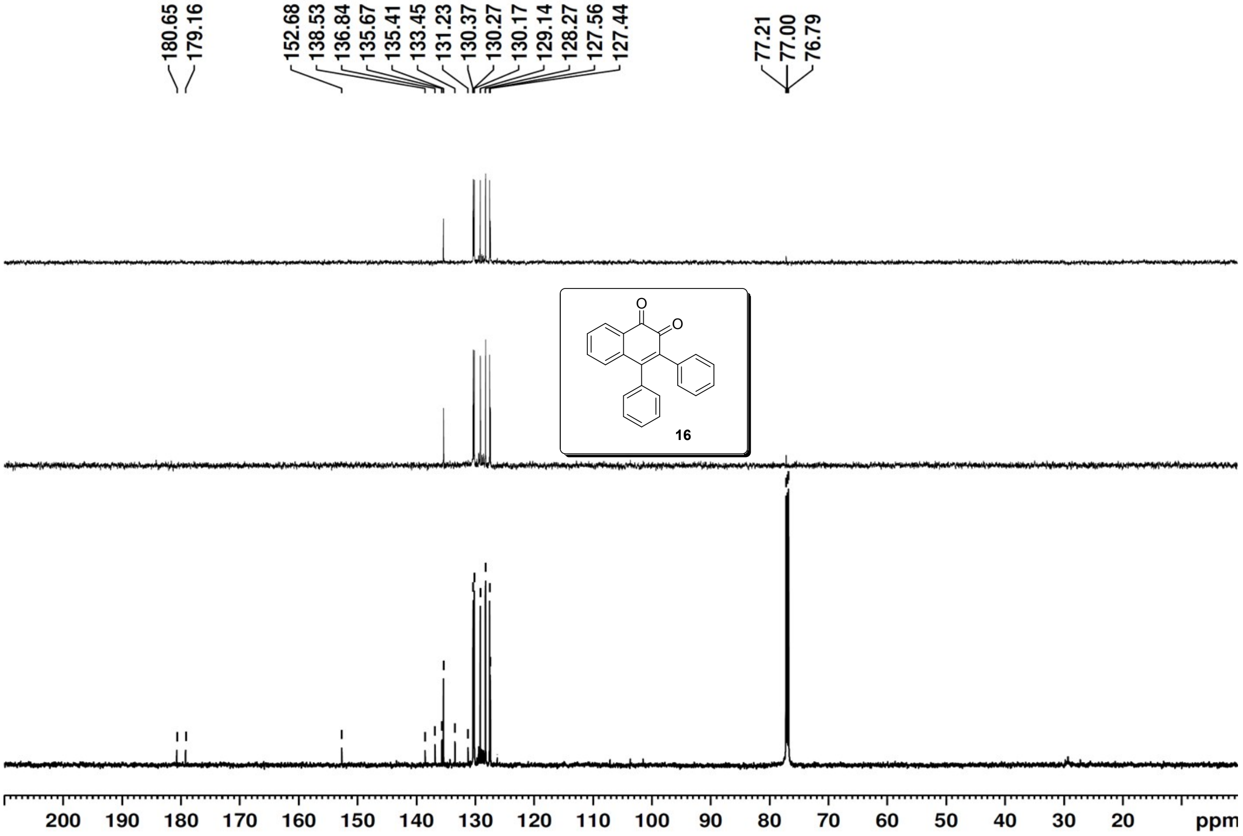

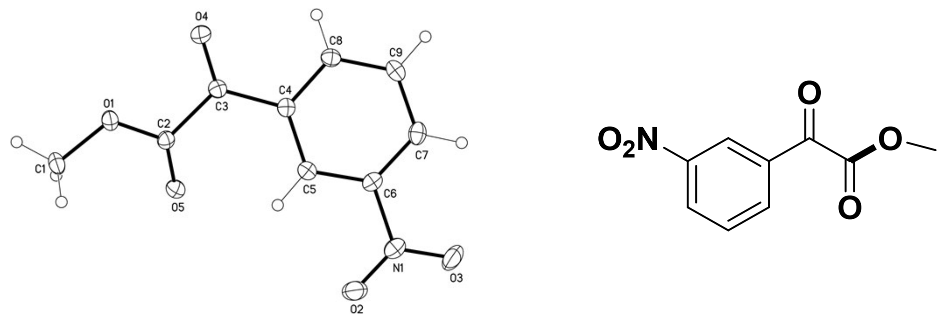

Figure S7. ORTEP diagram of compound **4n (CCDC :** 1584501**)**

Table S1. Crystal data and structure refinement for 170731LT_0M.

Identification code	170731LT_0m	
Empirical formula	C9 H7 N O5	
Formula weight	209.16	
Temperature	100(2) K	
Wavelength	0.71073 Å	
Crystal system	Triclinic	
Space group	P -1	
Unit cell dimensions	a = 7.2306(4) Å	α= 106.573(3)°.
	b = 7.3257(4) Å	β= 100.859(3)°.
	c = 8.8664(5) Å	γ = 94.501(3)°.
Volume	437.69(4) $Å^3$	
Z	2	
Density (calculated)	1.587 Mg/m^3	
Absorption coefficient	0.132 mm^{-1}	
F(000)	216	
Crystal size	0.15 x 0.10 x 0.10 mm^3	
Theta range for data collection	2.457 to 26.400°.	
Index ranges	-9<=h<=7, -9<=k<=9, -11<=l<=10	
Reflections collected	6864	
Independent reflections	1777 [R(int) = 0.0210]	
Completeness to theta = 25.242°	99.5 %	
Absorption correction	Semi-empirical from equivalents	
Max. and min. transmission	0.9485 and 0.8928	
Refinement method	Full-matrix least-squares on F^2	
Data / restraints / parameters	1777 / 0 / 137	
Goodness-of-fit on F^2	1.055	

Final R indices [I>2sigma(I)]	R1 = 0.0283, wR2 = 0.0767
R indices (all data)	R1 = 0.0302, wR2 = 0.0786
Extinction coefficient	n/a
Largest diff. peak and hole	0.327 and -0.233 e.Å^{-3}

Table S2. Atomic coordinates (x 10^4) and equivalent isotropic displacement parameters ($Å^2$x 10^3)for 170731LT_0M. U(eq) is defined as one third of the trace of the orthogonalized U^{ij} tensor.

	x	y	z	U(eq)
O(1)	70(1)	3951(1)	6444(1)	18(1)
O(2)	451(1)	-2545(1)	9729(1)	26(1)
O(3)	2771(1)	-4211(1)	10010(1)	28(1)
O(4)	3493(1)	3076(1)	6309(1)	22(1)
O(5)	-877(1)	948(1)	6437(1)	18(1)
C(1)	-1905(2)	4294(2)	6192(1)	20(1)
C(2)	347(1)	2221(1)	6544(1)	14(1)
C(3)	2481(1)	2011(1)	6727(1)	15(1)
C(4)	3205(1)	443(1)	7338(1)	14(1)
C(5)	2197(1)	-560(1)	8116(1)	15(1)
C(6)	3036(2)	-1970(1)	8645(1)	16(1)
N(1)	2005(1)	-2987(1)	9519(1)	19(1)
C(8)	5020(1)	29(1)	7136(1)	16(1)
C(9)	5806(2)	-1395(2)	7678(1)	18(1)
C(7)	4815(2)	-2427(1)	8438(1)	18(1)

Table S3. Bond lengths [Å] and angles [°] for 170731LT_0M.

O(1)-C(2)	1.3235(12)
O(1)-C(1)	1.4563(12)
O(2)-N(1)	1.2261(13)
O(3)-N(1)	1.2291(12)
O(4)-C(3)	1.2079(13)
O(5)-C(2)	1.2045(13)
C(1)-H(1)	0.9800
C(1)-H(7)	0.9800
C(1)-H(6)	0.9800
C(2)-C(3)	1.5453(14)
C(3)-C(4)	1.4931(14)
C(4)-C(5)	1.3935(14)
C(4)-C(8)	1.4021(14)
C(5)-C(6)	1.3868(14)
C(5)-H(2)	0.9500
C(6)-C(7)	1.3861(15)
C(6)-N(1)	1.4731(13)
C(8)-C(9)	1.3850(15)
C(8)-H(5)	0.9500
C(9)-C(7)	1.3884(15)
C(9)-H(4)	0.9500
C(7)-H(3)	0.9500
C(2)-O(1)-C(1)	115.33(8)
O(1)-C(1)-H(1)	109.5
O(1)-C(1)-H(7)	109.5
H(1)-C(1)-H(7)	109.5
O(1)-C(1)-H(6)	109.5
H(1)-C(1)-H(6)	109.5
H(7)-C(1)-H(6)	109.5
O(5)-C(2)-O(1)	125.74(9)
O(5)-C(2)-C(3)	124.04(9)
O(1)-C(2)-C(3)	110.15(8)
O(4)-C(3)-C(4)	122.15(9)

O(4)-C(3)-C(2)	118.72(9)
C(4)-C(3)-C(2)	119.06(9)
C(5)-C(4)-C(8)	119.80(9)
C(5)-C(4)-C(3)	123.46(9)
C(8)-C(4)-C(3)	116.72(9)
C(6)-C(5)-C(4)	117.91(9)
C(6)-C(5)-H(2)	121.0
C(4)-C(5)-H(2)	121.0
C(7)-C(6)-C(5)	123.36(10)
C(7)-C(6)-N(1)	118.53(9)
C(5)-C(6)-N(1)	118.08(9)
O(2)-N(1)-O(3)	124.09(9)
O(2)-N(1)-C(6)	118.13(9)
O(3)-N(1)-C(6)	117.77(9)
C(9)-C(8)-C(4)	120.54(10)
C(9)-C(8)-H(5)	119.7
C(4)-C(8)-H(5)	119.7
C(8)-C(9)-C(7)	120.50(10)
C(8)-C(9)-H(4)	119.7
C(7)-C(9)-H(4)	119.7
C(6)-C(7)-C(9)	117.87(9)
C(6)-C(7)-H(3)	121.1
C(9)-C(7)-H(3)	121.1

Symmetry transformations used to generate equivalent atoms:

Table S4. Anisotropic displacement parameters ($Å^2$x 10^3) for 170731LT_0M. The anisotropic
displacement factor exponent takes the form: -2□^{2}[h^2 $a^{*2}U^{11}$ + ... + 2 h k a* b* U^{12}]

	U^{11}	U^{22}	U^{33}	U^{23}	U^{13}	U^{12}
O(1)	16(1)	17(1)	24(1)	9(1)	4(1)	5(1)
O(2)	28(1)	25(1)	31(1)	11(1)	14(1)	3(1)
O(3)	33(1)	26(1)	30(1)	19(1)	3(1)	4(1)
O(4)	17(1)	22(1)	31(1)	15(1)	7(1)	2(1)
O(5)	14(1)	19(1)	23(1)	8(1)	4(1)	2(1)
C(1)	17(1)	22(1)	24(1)	10(1)	5(1)	9(1)
C(2)	16(1)	16(1)	12(1)	5(1)	3(1)	4(1)
C(3)	14(1)	14(1)	14(1)	4(1)	2(1)	1(1)
C(4)	14(1)	14(1)	12(1)	3(1)	1(1)	1(1)
C(5)	14(1)	16(1)	14(1)	3(1)	3(1)	2(1)
C(6)	18(1)	14(1)	12(1)	4(1)	2(1)	-1(1)
N(1)	23(1)	16(1)	16(1)	5(1)	3(1)	-1(1)
C(8)	14(1)	17(1)	15(1)	4(1)	3(1)	0(1)
C(9)	14(1)	19(1)	19(1)	3(1)	3(1)	4(1)
C(7)	20(1)	14(1)	16(1)	4(1)	0(1)	3(1)

Table S5. Hydrogen coordinates (x 10^4) and isotropic displacement parameters ($Å^2$x 10^3) for 170731LT_0M.

	x	y	z	U(eq)
H(1)	-2679	3236	5294	30
H(7)	-1995	5507	5940	30
H(6)	-2371	4371	7175	30
H(2)	974	-286	8279	18
H(5)	5715	731	6624	19
H(4)	7034	-1667	7528	21
H(3)	5338	-3414	8803	21

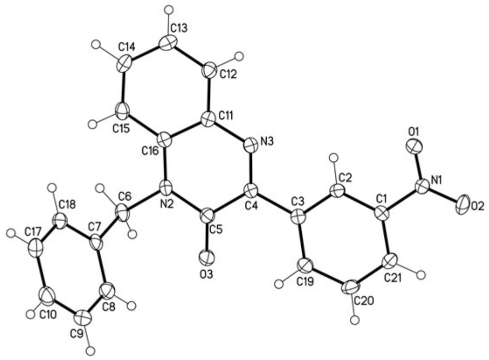

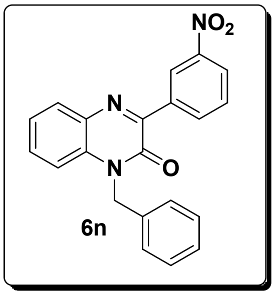

Figure S8. ORTEP diagram of compound **6n (CCDC no:** 1584500)

Table S6. Crystal data and structure refinement for 170916LT.

Identification code	170916LT	
Empirical formula	C21 H15 N3 O3	
Formula weight	357.36	
Temperature	100(2) K	
Wavelength	0.71073 Å	
Crystal system	Monoclinic	
Space group	P 21/c	
Unit cell dimensions	a = 10.2072(5) Å	α= 90°.
	b = 22.8173(11) Å	β= 90.0090(10)°.
	c = 7.0823(3) Å	γ = 90°.
Volume	1649.47(13) Å^3	
Z	4	
Density (calculated)	1.439 Mg/m^3	
Absorption coefficient	0.099 mm^{-1}	
F(000)	744	
Crystal size	0.20 x 0.15 x 0.05 mm^3	
Theta range for data collection	1.785 to 26.330°.	
Index ranges	-12<=h<=12, -28<=k<=28, -8<=l<=4	
Reflections collected	10011	
Independent reflections	3358 [R(int) = 0.0370]	
Completeness to theta = 25.242°	99.8 %	
Absorption correction	Semi-empirical from equivalents	
Max. and min. transmission	0.9485 and 0.8737	
Refinement method	Full-matrix least-squares on F^2	
Data / restraints / parameters	3358 / 0 / 244	

Goodness-of-fit on F^2	1.264
Final R indices [I>2sigma(I)]	R1 = 0.0591, wR2 = 0.1678
R indices (all data)	R1 = 0.0691, wR2 = 0.1728
Extinction coefficient	n/a
Largest diff. peak and hole	0.357 and -0.374 e.$Å^{-3}$

Table S7. Atomic coordinates (x 10^4) and equivalent isotropic displacement parameters (Å^{2}x 10^3) for 170916LT. U(eq) is defined as one third of the trace of the orthogonalized U^{ij} tensor.

	x	y	z	U(eq)
O(1)	1230(2)	7453(1)	1773(3)	28(1)
O(2)	2995(2)	7737(1)	362(3)	32(1)
O(3)	2729(2)	4435(1)	1265(3)	26(1)
N(1)	2283(2)	7350(1)	994(3)	19(1)
N(2)	683(2)	4237(1)	2301(3)	16(1)
N(3)	138(2)	5431(1)	2258(3)	17(1)
C(1)	2709(3)	6737(1)	840(4)	18(1)
C(2)	1852(2)	6297(1)	1342(3)	16(1)
C(3)	2268(2)	5715(1)	1257(3)	16(1)
C(4)	1295(2)	5250(1)	1781(3)	16(1)
C(5)	1663(3)	4620(1)	1754(3)	18(1)
C(6)	1045(3)	3603(1)	2234(4)	20(1)
C(7)	1763(3)	3408(1)	3998(4)	18(1)
C(8)	3121(3)	3425(1)	4096(4)	24(1)
C(9)	3773(3)	3241(1)	5716(5)	29(1)
C(10)	3063(3)	3038(1)	7253(4)	28(1)
C(11)	-802(2)	5032(1)	2815(3)	16(1)
C(12)	-2042(3)	5246(1)	3333(4)	20(1)
C(13)	-3007(3)	4869(1)	3929(4)	23(1)
C(14)	-2746(3)	4263(1)	3997(4)	22(1)
C(15)	-1547(3)	4042(1)	3492(4)	20(1)
C(16)	-548(3)	4422(1)	2877(3)	16(1)
C(17)	1712(3)	3014(1)	7159(4)	23(1)
C(18)	1062(3)	3199(1)	5552(4)	19(1)
C(19)	3562(3)	5601(1)	708(4)	21(1)
C(20)	4400(3)	6057(1)	198(4)	25(1)
C(21)	3977(3)	6631(1)	225(4)	22(1)

Table S8. Bond lengths [Å] and angles [°] for 170916LT.

O(1)-N(1)	1.231(3)
O(2)-N(1)	1.229(3)
O(3)-C(5)	1.217(3)
N(1)-C(1)	1.468(3)
N(2)-C(5)	1.384(3)
N(2)-C(16)	1.388(3)
N(2)-C(6)	1.492(3)
N(3)-C(4)	1.296(3)
N(3)-C(11)	1.379(3)
C(1)-C(2)	1.378(4)
C(1)-C(21)	1.387(4)
C(2)-C(3)	1.397(4)
C(2)-H(15)	0.9500
C(3)-C(19)	1.401(4)
C(3)-C(4)	1.500(4)
C(4)-C(5)	1.486(4)
C(6)-C(7)	1.516(4)
C(6)-H(10)	0.9900
C(6)-H(9)	0.9900
C(7)-C(8)	1.388(4)
C(7)-C(18)	1.397(4)
C(8)-C(9)	1.391(4)
C(8)-H(8)	0.9500
C(9)-C(10)	1.387(4)
C(9)-H(7)	0.9500
C(10)-C(17)	1.382(4)
C(10)-H(1)	0.9500
C(11)-C(12)	1.406(4)
C(11)-C(16)	1.418(4)
C(12)-C(13)	1.375(4)
C(12)-H(11)	0.9500
C(13)-C(14)	1.407(4)
C(13)-H(2)	0.9500
C(14)-C(15)	1.372(4)

C(14)-H(3)	0.9500
C(15)-C(16)	1.406(4)
C(15)-H(4)	0.9500
C(17)-C(18)	1.383(4)
C(17)-H(6)	0.9500
C(18)-H(5)	0.9500
C(19)-C(20)	1.394(4)
C(19)-H(12)	0.9500
C(20)-C(21)	1.379(4)
C(20)-H(14)	0.9500
C(21)-H(13)	0.9500

O(2)-N(1)-O(1)	122.8(2)
O(2)-N(1)-C(1)	118.9(2)
O(1)-N(1)-C(1)	118.4(2)
C(5)-N(2)-C(16)	123.0(2)
C(5)-N(2)-C(6)	115.1(2)
C(16)-N(2)-C(6)	121.9(2)
C(4)-N(3)-C(11)	119.9(2)
C(2)-C(1)-C(21)	123.1(2)
C(2)-C(1)-N(1)	119.1(2)
C(21)-C(1)-N(1)	117.8(2)
C(1)-C(2)-C(3)	119.2(2)
C(1)-C(2)-H(15)	120.4
C(3)-C(2)-H(15)	120.4
C(2)-C(3)-C(19)	118.3(2)
C(2)-C(3)-C(4)	117.4(2)
C(19)-C(3)-C(4)	124.2(2)
N(3)-C(4)-C(5)	122.8(2)
N(3)-C(4)-C(3)	116.3(2)
C(5)-C(4)-C(3)	120.9(2)
O(3)-C(5)-N(2)	120.4(2)
O(3)-C(5)-C(4)	124.5(2)
N(2)-C(5)-C(4)	115.1(2)
N(2)-C(6)-C(7)	112.3(2)
N(2)-C(6)-H(10)	109.1

C(7)-C(6)-H(10)	109.1
N(2)-C(6)-H(9)	109.1
C(7)-C(6)-H(9)	109.1
H(10)-C(6)-H(9)	107.9
C(8)-C(7)-C(18)	118.8(3)
C(8)-C(7)-C(6)	121.0(2)
C(18)-C(7)-C(6)	120.2(2)
C(7)-C(8)-C(9)	120.6(3)
C(7)-C(8)-H(8)	119.7
C(9)-C(8)-H(8)	119.7
C(10)-C(9)-C(8)	119.9(3)
C(10)-C(9)-H(7)	120.1
C(8)-C(9)-H(7)	120.1
C(17)-C(10)-C(9)	119.8(3)
C(17)-C(10)-H(1)	120.1
C(9)-C(10)-H(1)	120.1
N(3)-C(11)-C(12)	118.1(2)
N(3)-C(11)-C(16)	122.0(2)
C(12)-C(11)-C(16)	119.9(2)
C(13)-C(12)-C(11)	120.5(3)
C(13)-C(12)-H(11)	119.8
C(11)-C(12)-H(11)	119.8
C(12)-C(13)-C(14)	119.4(3)
C(12)-C(13)-H(2)	120.3
C(14)-C(13)-H(2)	120.3
C(15)-C(14)-C(13)	121.4(3)
C(15)-C(14)-H(3)	119.3
C(13)-C(14)-H(3)	119.3
C(14)-C(15)-C(16)	120.1(3)
C(14)-C(15)-H(4)	119.9
C(16)-C(15)-H(4)	119.9
N(2)-C(16)-C(15)	124.1(2)
N(2)-C(16)-C(11)	117.1(2)
C(15)-C(16)-C(11)	118.8(2)
C(10)-C(17)-C(18)	120.4(3)
C(10)-C(17)-H(6)	119.8

C(18)-C(17)-H(6)	119.8
C(17)-C(18)-C(7)	120.4(3)
C(17)-C(18)-H(5)	119.8
C(7)-C(18)-H(5)	119.8
C(20)-C(19)-C(3)	120.8(3)
C(20)-C(19)-H(12)	119.6
C(3)-C(19)-H(12)	119.6
C(21)-C(20)-C(19)	120.8(3)
C(21)-C(20)-H(14)	119.6
C(19)-C(20)-H(14)	119.6
C(20)-C(21)-C(1)	117.6(2)
C(20)-C(21)-H(13)	121.2
C(1)-C(21)-H(13)	121.2

Symmetry transformations used to generate equivalent atoms:

Table S9. Anisotropic displacement parameters ($Å^2 x 10^3$) for 170916LT. The anisotropic displacement factor exponent takes the form: -2□^{2}[$h^2 a^{*2}U^{11}$ + ... + 2 h k a* b* U^{12}]

	U^{11}	U^{22}	U^{33}	U^{23}	U^{13}	U^{12}
O(1)	21(1)	19(1)	43(1)	2(1)	8(1)	5(1)
O(2)	29(1)	17(1)	50(1)	4(1)	9(1)	-6(1)
O(3)	24(1)	19(1)	35(1)	4(1)	11(1)	5(1)
N(1)	17(1)	17(1)	24(1)	1(1)	-1(1)	-2(1)
N(2)	21(1)	12(1)	16(1)	1(1)	1(1)	0(1)
N(3)	19(1)	16(1)	16(1)	-1(1)	0(1)	0(1)
C(1)	18(1)	16(1)	18(1)	1(1)	-1(1)	2(1)
C(2)	14(1)	20(1)	15(1)	0(1)	0(1)	0(1)
C(3)	19(1)	18(1)	13(1)	-1(1)	1(1)	0(1)
C(4)	18(1)	15(1)	14(1)	0(1)	0(1)	0(1)
C(5)	19(1)	19(1)	15(1)	2(1)	2(1)	1(1)
C(6)	27(1)	14(1)	18(1)	-1(1)	2(1)	1(1)
C(7)	23(1)	10(1)	22(1)	-2(1)	2(1)	2(1)
C(8)	24(1)	19(1)	30(1)	1(1)	7(1)	2(1)
C(9)	20(1)	26(2)	42(2)	6(1)	-2(1)	2(1)
C(10)	29(2)	24(2)	32(2)	8(1)	-9(1)	-2(1)
C(11)	19(1)	18(1)	13(1)	-1(1)	-1(1)	-2(1)
C(12)	21(1)	18(1)	20(1)	-2(1)	2(1)	-1(1)
C(13)	17(1)	27(2)	23(1)	-2(1)	3(1)	-2(1)
C(14)	22(1)	24(1)	20(1)	2(1)	0(1)	-8(1)
C(15)	25(1)	18(1)	16(1)	3(1)	-3(1)	-3(1)
C(16)	19(1)	18(1)	11(1)	0(1)	-2(1)	0(1)
C(17)	28(2)	20(1)	22(1)	3(1)	-2(1)	-4(1)
C(18)	21(1)	15(1)	22(1)	-2(1)	-1(1)	-1(1)
C(19)	20(1)	17(1)	27(1)	-2(1)	3(1)	2(1)
C(20)	16(1)	26(2)	33(2)	-2(1)	6(1)	0(1)
C(21)	18(1)	21(1)	25(1)	1(1)	3(1)	-4(1)

Table S10. Hydrogen coordinates (x 10^4) and isotropic displacement parameters ($Å^2$x 10^3) for 170916LT.

	x	y	z	U(eq)
H(15)	987	6389	1741	20
H(10)	1609	3532	1120	24
H(9)	240	3366	2085	24
H(8)	3610	3563	3045	29
H(7)	4702	3255	5770	35
H(1)	3505	2916	8366	34
H(11)	-2215	5655	3270	23
H(2)	-3841	5015	4292	27
H(3)	-3415	4002	4400	26
H(4)	-1390	3633	3558	24
H(6)	1227	2871	8205	28
H(5)	132	3183	5505	23
H(12)	3873	5209	683	26
H(14)	5274	5971	-174	30
H(13)	4533	6942	-162	26

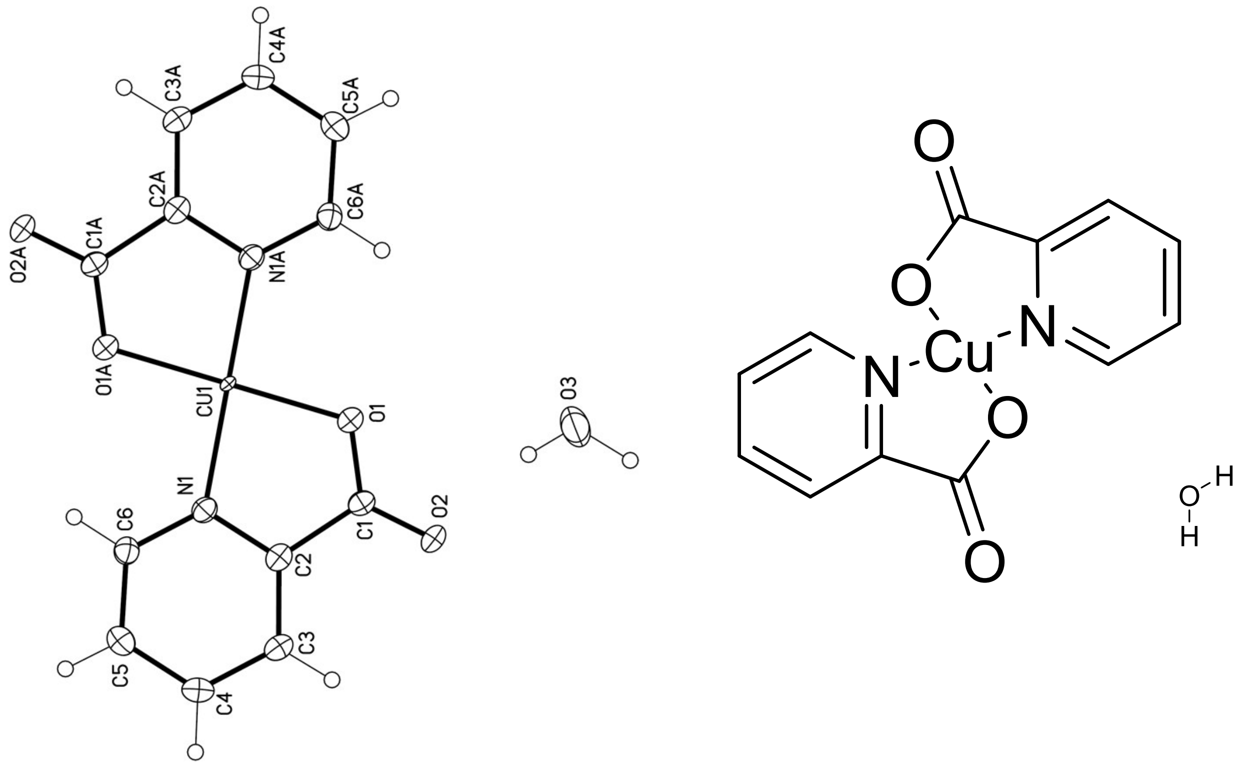

Figure S9. ORTEP diagram of compound bis picolinate Cu(II) complex obtained as a blue precipitate Cu(II)L_2 **(CCDC :** 1847226**)** crystallized in ethanol and water mixture.

Table S11. Crystal data and structure refinement for mo_170824lt_0m_a.

Identification code	mo_170824lt_0m_a	
Empirical formula	C12 H12 Cu N2 O6	
Formula weight	343.78	
Temperature	99(2) K	
Wavelength	0.71073 Å	
Crystal system	Triclinic	
Space group	P -1	
Unit cell dimensions	a = 5.0866(4) Å	α= 76.038(2)°.
	b = 7.5068(5) Å	β= 85.125(2)°.
	c = 9.0684(6) Å	γ = 72.2290(10)°.
Volume	319.98(4) $Å^3$	
Z	1	
Density (calculated)	1.784 Mg/m^3	
Absorption coefficient	1.738 mm^{-1}	
F(000)	175	
Crystal size	0.15 x 0.12 x 0.03 mm^3	
Theta range for data collection	2.926 to 26.702°.	
Index ranges	-6<=h<=4, -9<=k<=9, -11<=l<=11	
Reflections collected	5170	
Independent reflections	1351 [R(int) = 0.0132]	
Completeness to theta = 25.242°	99.4 %	
Absorption correction	Semi-empirical from equivalents	
Max. and min. transmission	0.95 and 0.78	
Refinement method	Full-matrix least-squares on F^2	
Data / restraints / parameters	1351 / 0 / 98	
Goodness-of-fit on F^2	1.160	
Final R indices [I>2sigma(I)]	R1 = 0.0172, wR2 = 0.0442	
R indices (all data)	R1 = 0.0172, wR2 = 0.0442	
Extinction coefficient	n/a	
Largest diff. peak and hole	0.394 and -0.290 e.$Å^{-3}$	

Table S12. Atomic coordinates (x 10^4) and equivalent isotropic displacement parameters (Å^{2}x 10^3) for mo_170824lt_0m_a. U(eq) is defined as one third of the trace of the orthogonalized U^{ij} tensor.

	x	y	z	U(eq)
Cu(1)	10000	0	5000	9(1)
N(1)	7819(2)	2291(2)	3591(1)	9(1)
O(1)	6691(2)	424(1)	6270(1)	11(1)
O(2)	2250(2)	2162(1)	6165(1)	13(1)
O(3)	2312(3)	579(2)	9415(1)	29(1)
C(1)	4634(3)	1798(2)	5644(2)	10(1)
C(2)	5270(3)	3005(2)	4142(2)	9(1)
C(3)	3443(3)	4700(2)	3397(2)	12(1)
C(4)	4307(3)	5698(2)	2041(2)	14(1)
C(5)	6918(3)	4945(2)	1465(2)	14(1)
C(6)	8625(3)	3221(2)	2260(2)	12(1)

Table S13. Bond lengths [Å] and angles [°] for mo_170824lt_0m_a.

Cu(1)-O(1)	1.9446(9)
Cu(1)-O(1)#1	1.9446(9)
Cu(1)-N(1)#1	1.9589(11)
Cu(1)-N(1)	1.9589(11)
N(1)-C(6)	1.3400(18)
N(1)-C(2)	1.3469(17)
O(1)-C(1)	1.2809(16)
O(2)-C(1)	1.2369(16)
O(3)-H(7)	0.8383
O(3)-H(8)	0.7852
O(3)-H(8')	0.8081
C(1)-C(2)	1.5127(18)
C(2)-C(3)	1.3819(19)
C(3)-C(4)	1.390(2)
C(3)-H(3)	0.9500
C(4)-C(5)	1.387(2)
C(4)-H(4)	0.9500
C(5)-C(6)	1.385(2)
C(5)-H(5)	0.9500
C(6)-H(6)	0.9500
O(1)-Cu(1)-O(1)#1	180.0
O(1)-Cu(1)-N(1)#1	96.11(4)
O(1)#1-Cu(1)-N(1)#1	83.89(4)
O(1)-Cu(1)-N(1)	83.89(4)
O(1)#1-Cu(1)-N(1)	96.11(4)
N(1)#1-Cu(1)-N(1)	180.0
C(6)-N(1)-C(2)	119.62(12)
C(6)-N(1)-Cu(1)	128.11(9)
C(2)-N(1)-Cu(1)	112.14(9)
C(1)-O(1)-Cu(1)	114.33(8)
H(7)-O(3)-H(8)	118.5
H(7)-O(3)-H(8')	106.4
O(2)-C(1)-O(1)	125.04(12)

O(2)-C(1)-C(2)	119.79(12)
O(1)-C(1)-C(2)	115.16(11)
N(1)-C(2)-C(3)	122.27(13)
N(1)-C(2)-C(1)	113.86(11)
C(3)-C(2)-C(1)	123.86(12)
C(2)-C(3)-C(4)	118.19(13)
C(2)-C(3)-H(3)	120.9
C(4)-C(3)-H(3)	120.9
C(5)-C(4)-C(3)	119.37(13)
C(5)-C(4)-H(4)	120.3
C(3)-C(4)-H(4)	120.3
C(6)-C(5)-C(4)	119.34(13)
C(6)-C(5)-H(5)	120.3
C(4)-C(5)-H(5)	120.3
N(1)-C(6)-C(5)	121.16(13)
N(1)-C(6)-H(6)	119.4
C(5)-C(6)-H(6)	119.4

Symmetry transformations used to generate equivalent atoms:

#1 -x+2,-y,-z+1

Table S14. Anisotropic displacement parameters ($Å^2$x 10^3) for mo_170824lt_0m_a. The anisotropic displacement factor exponent takes the form: $-2\pi^2[h^2 a^{*2}U^{11} + ... + 2 h k a^* b^* U^{12}]$

	U^{11}	U^{22}	U^{33}	U^{23}	U^{13}	U^{12}
Cu(1)	5(1)	9(1)	10(1)	0(1)	1(1)	0(1)
N(1)	7(1)	10(1)	11(1)	-3(1)	0(1)	-2(1)
O(1)	8(1)	11(1)	11(1)	-1(1)	1(1)	-1(1)
O(2)	7(1)	15(1)	14(1)	-4(1)	3(1)	-1(1)
O(3)	26(1)	43(1)	16(1)	4(1)	2(1)	-15(1)
C(1)	10(1)	9(1)	12(1)	-5(1)	0(1)	-2(1)
C(2)	8(1)	11(1)	10(1)	-5(1)	0(1)	-3(1)
C(3)	9(1)	11(1)	14(1)	-6(1)	-2(1)	-1(1)
C(4)	15(1)	10(1)	15(1)	-2(1)	-4(1)	-1(1)
C(5)	16(1)	15(1)	11(1)	0(1)	-1(1)	-6(1)
C(6)	11(1)	14(1)	12(1)	-3(1)	1(1)	-4(1)

Table S15. Hydrogen coordinates (x 10^4) and isotropic displacement parameters ($Å^2$x 10^3) for mo_170824lt_0m_a.

	x	y	z	U(eq)
H(3)	1646	5170	3800	14
H(4)	3120	6886	1513	16
H(5)	7530	5604	534	17
H(6)	10399	2689	1856	14
H(7)	2408	891	8464	50
H(8)	1087	190	9790	50
H(8')	3881	208	9714	50

CheckCIF file for **4n (CCDC:**1584501**)**

checkCIF/PLATON report

Structure factors have been supplied for datablock(s) 170731LT_0m

THIS REPORT IS FOR GUIDANCE ONLY. IF USED AS PART OF A REVIEW PROCEDURE FOR PUBLICATION, IT SHOULD NOT REPLACE THE EXPERTISE OF AN EXPERIENCED CRYSTALLOGRAPHIC REFEREE.

No syntax errors found. CIF dictionary Interpreting this report

Datablock: 170731LT_0m

```
Bond precision:   C-C = 0.0015 A          Wavelength=0.71073

Cell:          a=7.2306(4)        b=7.3257(4)       c=8.8664(5)
               alpha=106.573(3)   beta=100.859(3)   gamma=94.501(3)
Temperature:   100 K

                 Calculated                  Reported
Volume           437.69(4)                   437.69(4)
Space group      P -1                        P -1
Hall group       -P 1                        -P 1
Moiety formula   C9 H7 N O5                  ?
Sum formula      C9 H7 N O5                  C9 H7 N O5
Mr               209.16                      209.16
Dx,g cm-3        1.587                       1.587
Z                2                           2
Mu (mm-1)        0.132                       0.132
F000             216.0                       216.0
F000'            216.15
h,k,lmax         9,9,11                      9,9,11
Nref             1791                        1777
Tmin,Tmax        0.984,0.987                 0.893,0.948
Tmin'            0.980

Correction method= # Reported T Limits: Tmin=0.893 Tmax=0.948
AbsCorr = MULTI-SCAN

Data completeness= 0.992           Theta(max)= 26.400

R(reflections)= 0.0283( 1657)      wR2(reflections)= 0.0786( 1777)

S = 1.055                  Npar= 137
```

The following ALERTS were generated. Each ALERT has the format
test-name_ALERT_alert-type_alert-level.
Click on the hyperlinks for more details of the test.

Alert level C

```
PLAT369_ALERT_2_C Long   C(sp2)-C(sp2) Bond  C2       - C3       .        1.55 Ang.
PLAT911_ALERT_3_C Missing FCF Refl Between Thmin & STh/L=    0.600          8 Report
```

Alert level G

```
PLAT154_ALERT_1_G The s.u.'s on the Cell Angles are Equal ..(Note)      0.003 Degree
PLAT432_ALERT_2_G Short Inter X...Y Contact  O5       ..C3              2.97 Ang.
PLAT912_ALERT_4_G Missing # of FCF Reflections Above STh/L=  0.600         6 Note
PLAT913_ALERT_3_G Missing # of Very Strong Reflections in FCF ....         2 Note
PLAT978_ALERT_2_G Number C-C Bonds with Positive Residual Density.        13 Info
```

```
   0 ALERT level A = Most likely a serious problem - resolve or explain
   0 ALERT level B = A potentially serious problem, consider carefully
   2 ALERT level C = Check. Ensure it is not caused by an omission or oversight
   5 ALERT level G = General information/check it is not something unexpected

   1 ALERT type 1 CIF construction/syntax error, inconsistent or missing data
   3 ALERT type 2 Indicator that the structure model may be wrong or deficient
   2 ALERT type 3 Indicator that the structure quality may be low
   1 ALERT type 4 Improvement, methodology, query or suggestion
   0 ALERT type 5 Informative message, check
```

It is advisable to attempt to resolve as many as possible of the alerts in all categories. Often the minor alerts point to easily fixed oversights, errors and omissions in your CIF or refinement strategy, so attention to these fine details can be worthwhile. In order to resolve some of the more serious problems it may be necessary to carry out additional measurements or structure refinements. However, the purpose of your study may justify the reported deviations and the more serious of these should normally be commented upon in the discussion or experimental section of a paper or in the "special_details" fields of the CIF. checkCIF was carefully designed to identify outliers and unusual parameters, but every test has its limitations and alerts that are not important in a particular case may appear. Conversely, the absence of alerts does not guarantee there are no aspects of the results needing attention. It is up to the individual to critically assess their own results and, if necessary, seek expert advice.

Publication of your CIF in IUCr journals

A basic structural check has been run on your CIF. These basic checks will be run on all CIFs submitted for publication in IUCr journals (*Acta Crystallographica*, *Journal of Applied Crystallography*, *Journal of Synchrotron Radiation*); however, if you intend to submit to *Acta Crystallographica Section C* or *E* or *IUCrData*, you should make sure that full publication checks are run on the final version of your CIF prior to submission.

Publication of your CIF in other journals

Please refer to the *Notes for Authors* of the relevant journal for any special instructions relating to CIF submission.

PLATON version of 13/12/2017; check.def file version of 12/12/2017

Datablock 170731LT_0m - ellipsoid plot

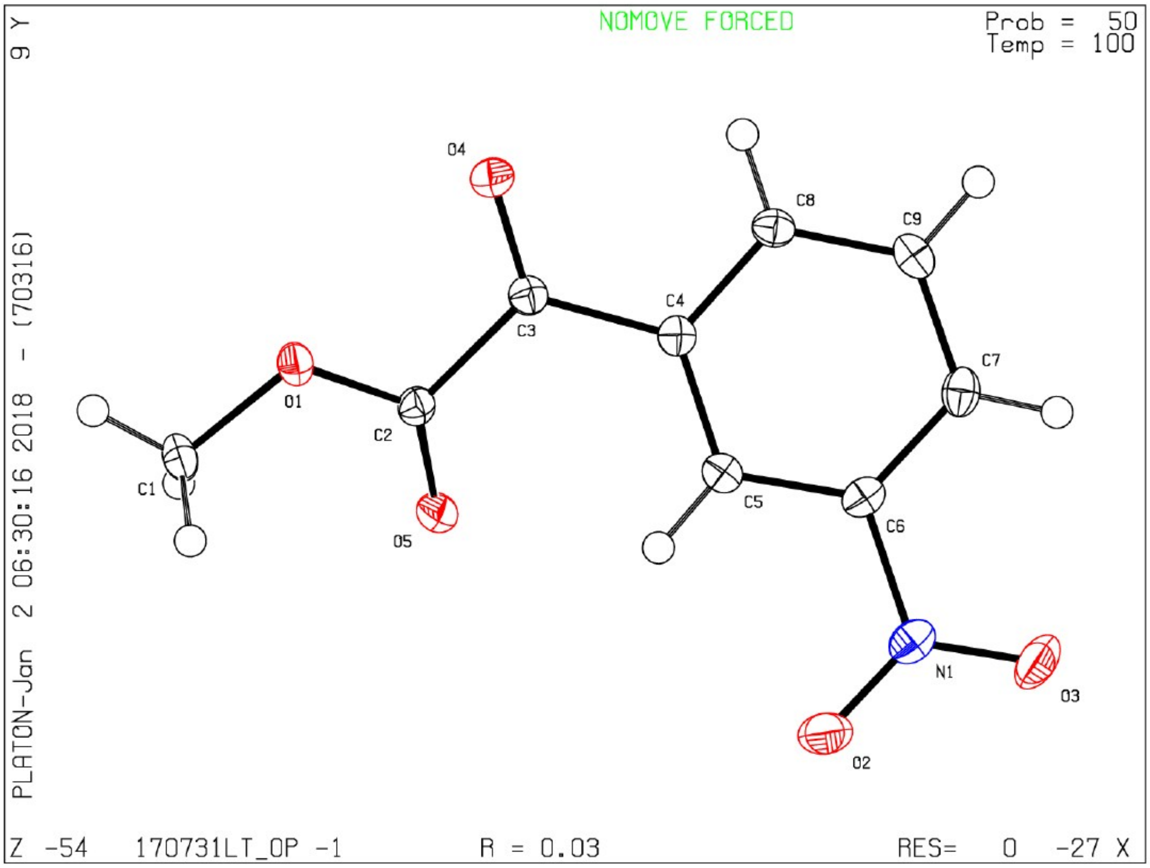

CheckCIF file for (**6n) CCDC :** 1584500

checkCIF/PLATON report

Structure factors have been supplied for datablock(s) 170916LT

THIS REPORT IS FOR GUIDANCE ONLY. IF USED AS PART OF A REVIEW PROCEDURE FOR PUBLICATION, IT SHOULD NOT REPLACE THE EXPERTISE OF AN EXPERIENCED CRYSTALLOGRAPHIC REFEREE.

No syntax errors found. CIF dictionary Interpreting this report

Datablock: 170916LT

Bond precision:	C-C = 0.0040 A		Wavelength=0.71073
Cell:	a=10.2072(5)	b=22.8173(11)	c=7.0823(3)
	alpha=90	beta=90.009(1)	gamma=90
Temperature:	100 K		

	Calculated	Reported
Volume	1649.47(13)	1649.47(13)
Space group	P 21/c	P 21/c
Hall group	-P 2ybc	-P 2ybc
Moiety formula	C21 H15 N3 O3	?
Sum formula	C21 H15 N3 O3	C21 H15 N3 O3
Mr	357.36	357.36
Dx,g cm-3	1.439	1.439
Z	4	4
Mu (mm-1)	0.099	0.099
F000	744.0	744.0
F000'	744.34	
h,k,lmax	12,28,8	12,28,8
Nref	3362	3358
Tmin,Tmax	0.982,0.995	0.874,0.948
Tmin'	0.980	

Correction method= # Reported T Limits: Tmin=0.874 Tmax=0.948
AbsCorr = MULTI-SCAN

Data completeness= 0.999 Theta(max)= 26.330

R(reflections)= 0.0591(2802) wR2(reflections)= 0.1728(3358)

S = 1.264 Npar= 244

The following ALERTS were generated. Each ALERT has the format
test-name_ALERT_alert-type_alert-level.
Click on the hyperlinks for more details of the test.

```
Alert level B
PLAT919_ALERT_3_B Reflection # Likely Affected by the Beamstop ...          2 Check
PLAT930_ALERT_2_B Check Twin Law ( 1 0  0  )[ 1 0  0  ] Estimated BASF       0.03
PLAT934_ALERT_3_B Number of (Iobs-Icalc)/SigmaW > 10 Outliers ....          4 Check
```

```
Alert level C
PLAT906_ALERT_3_C Large K Value in the Analysis of Variance ......     10.903 Check
PLAT911_ALERT_3_C Missing FCF Refl Between Thmin & STh/L=    0.600          5 Report
PLAT918_ALERT_3_C Reflection(s) with I(obs) much Smaller I(calc) .          1 Check
```

```
Alert level G
PLAT870_ALERT_4_G ALERTS Related to Twinning Effects Suppressed ..          ! Info
PLAT913_ALERT_3_G Missing # of Very Strong Reflections in FCF ....          2 Note
PLAT931_ALERT_5_G Found Twin Law ( 1 0  0  )[         ] Est.  BASF       0.03 Check
```

```
   0 ALERT level A = Most likely a serious problem - resolve or explain
   3 ALERT level B = A potentially serious problem, consider carefully
   3 ALERT level C = Check. Ensure it is not caused by an omission or oversight
   3 ALERT level G = General information/check it is not something unexpected

   0 ALERT type 1 CIF construction/syntax error, inconsistent or missing data
   1 ALERT type 2 Indicator that the structure model may be wrong or deficient
   6 ALERT type 3 Indicator that the structure quality may be low
   1 ALERT type 4 Improvement, methodology, query or suggestion
   1 ALERT type 5 Informative message, check
```

It is advisable to attempt to resolve as many as possible of the alerts in all categories. Often the minor alerts point to easily fixed oversights, errors and omissions in your CIF or refinement strategy, so attention to these fine details can be worthwhile. In order to resolve some of the more serious problems it may be necessary to carry out additional measurements or structure refinements. However, the purpose of your study may justify the reported deviations and the more serious of these should normally be commented upon in the discussion or experimental section of a paper or in the "special_details" fields of the CIF. checkCIF was carefully designed to identify outliers and unusual parameters, but every test has its limitations and alerts that are not important in a particular case may appear. Conversely, the absence of alerts does not guarantee there are no aspects of the results needing attention. It is up to the individual to critically assess their own results and, if necessary, seek expert advice.

Publication of your CIF in IUCr journals

A basic structural check has been run on your CIF. These basic checks will be run on all CIFs submitted for publication in IUCr journals (*Acta Crystallographica*, *Journal of Applied Crystallography*, *Journal of Synchrotron Radiation*); however, if you intend to submit to *Acta Crystallographica Section C* or *E* or *IUCrData*, you should make sure that full publication checks are run on the final version of your CIF prior to submission.

Publication of your CIF in other journals

Please refer to the *Notes for Authors* of the relevant journal for any special instructions relating to CIF submission.

PLATON version of 13/12/2017; check.def file version of 12/12/2017

Datablock 170916LT - ellipsoid plot

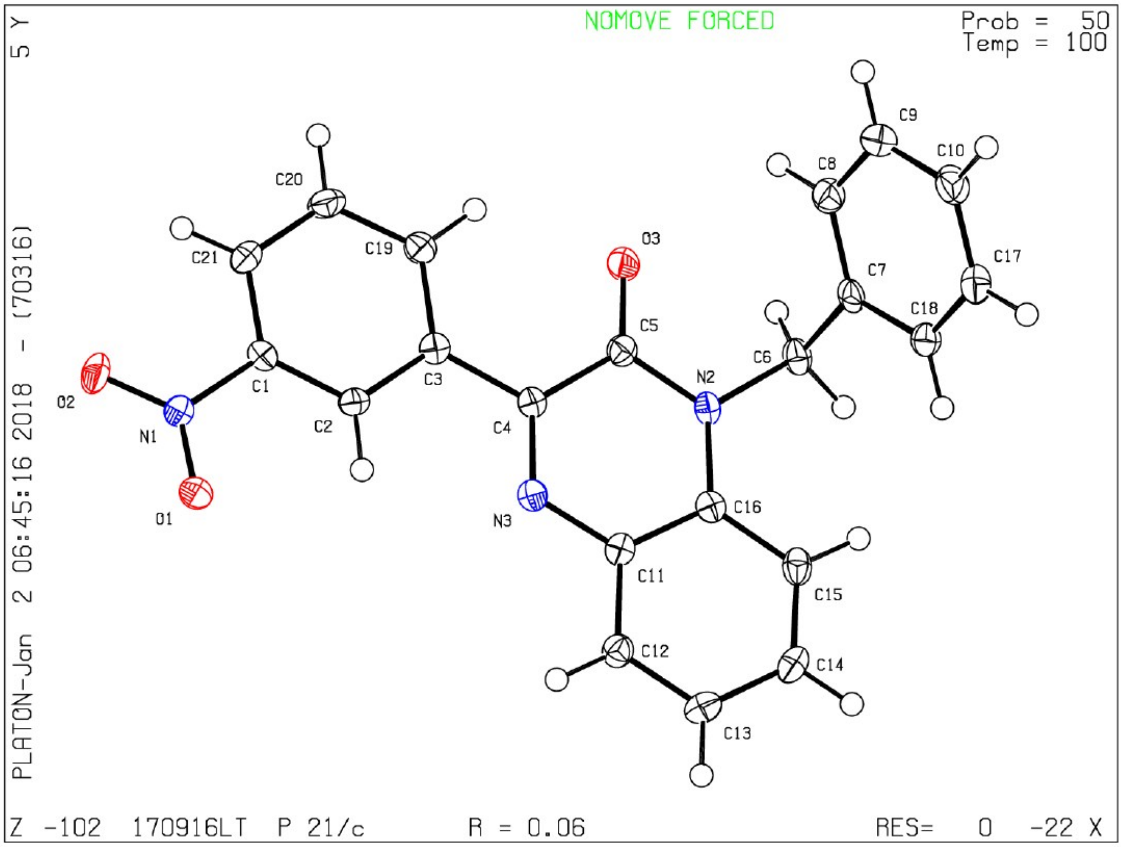

CheckCIF file for Cu(II) picolinate complex **CCDC :** 1847226

checkCIF/PLATON report

Structure factors have been supplied for datablock(s) mo_170824lt_0m_a

THIS REPORT IS FOR GUIDANCE ONLY. IF USED AS PART OF A REVIEW PROCEDURE FOR PUBLICATION, IT SHOULD NOT REPLACE THE EXPERTISE OF AN EXPERIENCED CRYSTALLOGRAPHIC REFEREE.

No syntax errors found. CIF dictionary Interpreting this report

Datablock: mo_170824lt_0m_a

```
Bond precision:   C-C = 0.0020 A          Wavelength=0.71073

Cell:           a=5.0866(4)       b=7.5068(5)      c=9.0684(6)
                alpha=76.038(2)   beta=85.125(2)   gamma=72.229(1)
Temperature:    99 K

                 Calculated                  Reported
Volume           319.98(4)                   319.98(4)
Space group      P -1                        P -1
Hall group       -P 1                        -P 1
Moiety formula   C12 H8 Cu N2 O4, 2(H2 O)    ?
Sum formula      C12 H12 Cu N2 O6            C12 H12 Cu N2 O6
Mr               343.79                      343.78
Dx,g cm-3        1.784                       1.784
Z                1                           1
Mu (mm-1)        1.738                       1.738
F000             175.0                       175.0
F000'            175.40
h,k,lmax         6,9,11                      6,9,11
Nref             1365                        1351
Tmin,Tmax        0.779,0.949                 0.780,0.950
Tmin'            0.771

Correction method= # Reported T Limits: Tmin=0.780 Tmax=0.950
AbsCorr = MULTI-SCAN

Data completeness= 0.990          Theta(max)= 26.702

R(reflections)= 0.0172( 1349)     wR2(reflections)= 0.0442( 1351)

S = 1.160                 Npar= 98
```

The following ALERTS were generated. Each ALERT has the format
test-name_ALERT_alert-type_alert-level.
Click on the hyperlinks for more details of the test.

```
 Alert level C
PLAT911_ALERT_3_C Missing FCF Refl Between Thmin & STh/L=    0.600          6 Report
PLAT913_ALERT_3_C Missing # of Very Strong Reflections in FCF ....          6 Note
```

```
 Alert level G
PLAT004_ALERT_5_G Polymeric Structure Found with Maximum Dimension          1 Info
PLAT007_ALERT_5_G Number of Unrefined Donor-H Atoms ..............          3 Report
PLAT066_ALERT_1_G Predicted and Reported Tmin&Tmax Range Identical          ? Check
PLAT169_ALERT_4_G The CIF-Embedded .res File Contains AFIX 1 Recds          1 Report
PLAT232_ALERT_2_G Hirshfeld Test Diff (M-X) Cu1      --N1       .         7.2 s.u.
PLAT300_ALERT_4_G Atom Site Occupancy of H8        Constrained at         0.5 Check
PLAT300_ALERT_4_G Atom Site Occupancy of H8'       Constrained at         0.5 Check
PLAT910_ALERT_3_G Missing # of FCF Reflection(s) Below Theta(Min).          1 Note
PLAT912_ALERT_4_G Missing # of FCF Reflections Above STh/L=  0.600          7 Note
PLAT961_ALERT_5_G Dataset Contains no Negative Intensities .......     Please Check
PLAT978_ALERT_2_G Number C-C Bonds with Positive Residual Density.          4 Info
```

```
   0 ALERT level A = Most likely a serious problem - resolve or explain
   0 ALERT level B = A potentially serious problem, consider carefully
   2 ALERT level C = Check. Ensure it is not caused by an omission or oversight
  11 ALERT level G = General information/check it is not something unexpected

   1 ALERT type 1 CIF construction/syntax error, inconsistent or missing data
   2 ALERT type 2 Indicator that the structure model may be wrong or deficient
   3 ALERT type 3 Indicator that the structure quality may be low
   4 ALERT type 4 Improvement, methodology, query or suggestion
   3 ALERT type 5 Informative message, check
```

It is advisable to attempt to resolve as many as possible of the alerts in all categories. Often the minor alerts point to easily fixed oversights, errors and omissions in your CIF or refinement strategy, so attention to these fine details can be worthwhile. In order to resolve some of the more serious problems it may be necessary to carry out additional measurements or structure refinements. However, the purpose of your study may justify the reported deviations and the more serious of these should normally be commented upon in the discussion or experimental section of a paper or in the "special_details" fields of the CIF. checkCIF was carefully designed to identify outliers and unusual parameters, but every test has its limitations and alerts that are not important in a particular case may appear. Conversely, the absence of alerts does not guarantee there are no aspects of the results needing attention. It is up to the individual to critically assess their own results and, if necessary, seek expert advice.

Publication of your CIF in IUCr journals

A basic structural check has been run on your CIF. These basic checks will be run on all CIFs submitted for publication in IUCr journals (*Acta Crystallographica*, *Journal of Applied Crystallography*, *Journal of Synchrotron Radiation*); however, if you intend to submit to *Acta Crystallographica Section C* or *E* or *IUCrData*, you should make sure that full publication checks are run on the final version of your CIF prior to submission.

Publication of your CIF in other journals

Please refer to the *Notes for Authors* of the relevant journal for any special instructions relating to CIF submission.

PLATON version of 23/04/2018; check.def file version of 23/04/2018

Datablock mo_170824lt_0m_a - ellipsoid plot

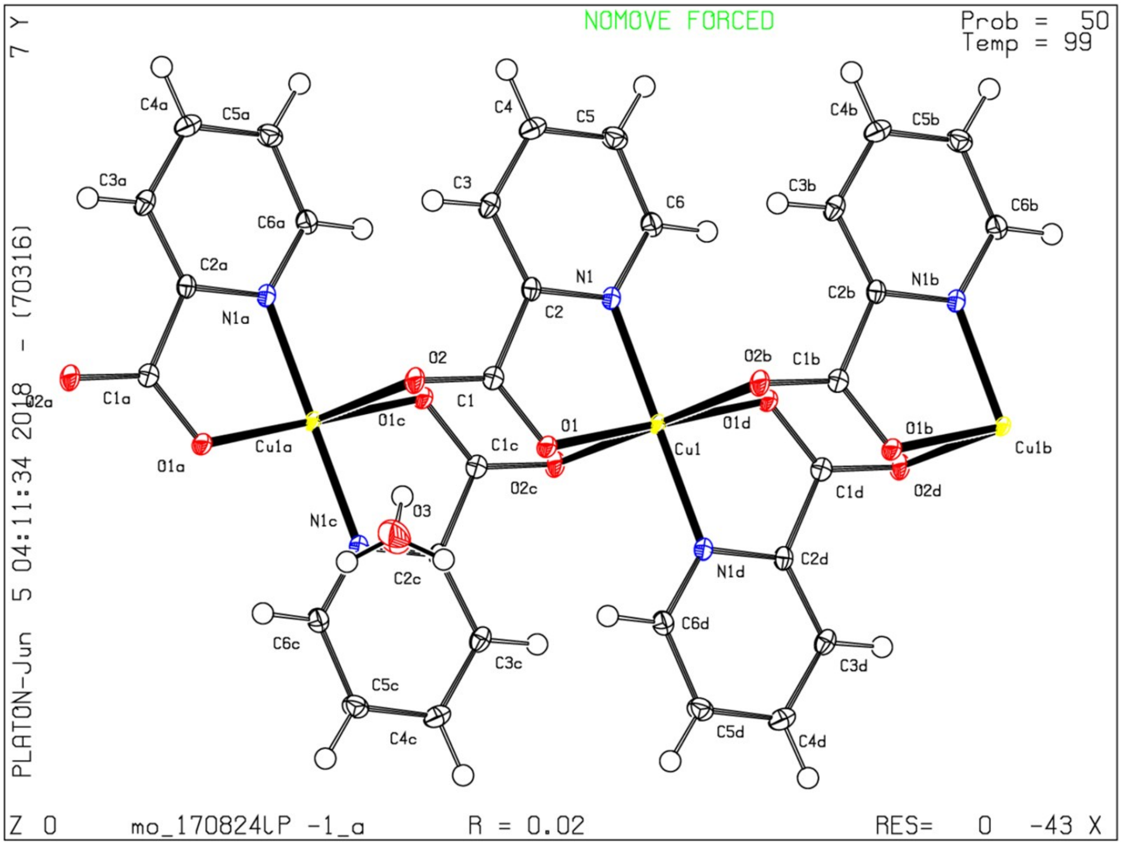

Supplement: Supplementary file 1 [file SC-009-C8SC03447H-s001.pdf]
